# Supplementary figures and images for: Analysis of the RNA virome of basal hexapods
Source: PeerJ. 2020 Jan 9;8:e8336. doi: 10.7717/peerj.8336 (PMC6955108; doi:10.7717/peerj.8336)

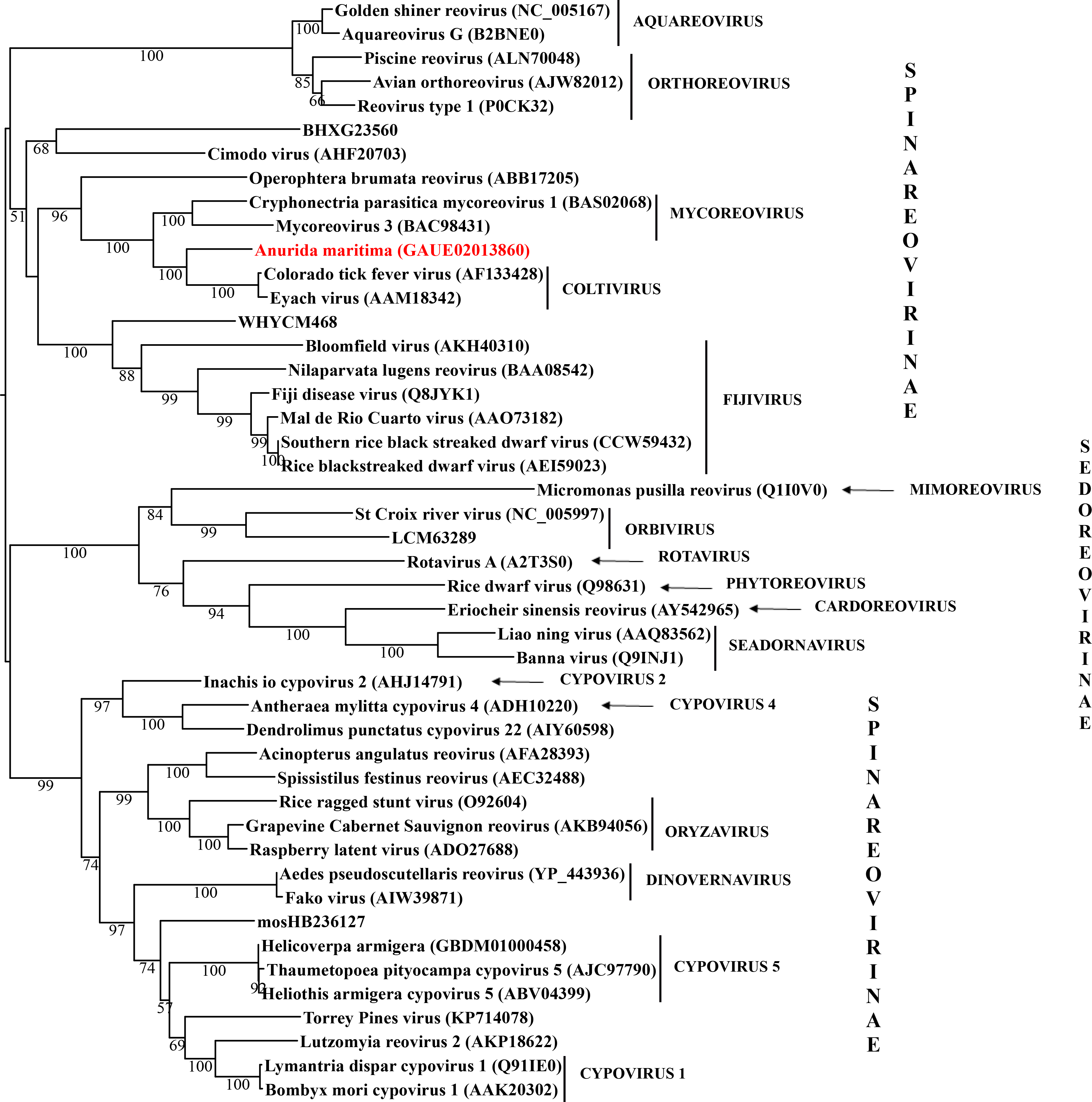

Supplement: Figure S1 — Each phylogeny provided in this and the other supplementary figures is based on the analysis of the viral RdRP domain. The names of the viruses are marked with different colours based on their host taxonomy; springtails (Collembola) are red, Diplura are violet, Monocondylia are green, and Zygentoma are blue. The star symbol denotes host taxa that contain endogenous virus copies (EVEs). The best fit model of amino acid substitution for this data set was determined as Blosum62+I+G4 according to the Bayesian information criterion. Most sequences were obtained from the GenBank; species names and accession numbers are included. Sequences from the Shi et al. (2016) have the same unique accession numbers as in the original publication. [file peerj-08-8336-s001.png]

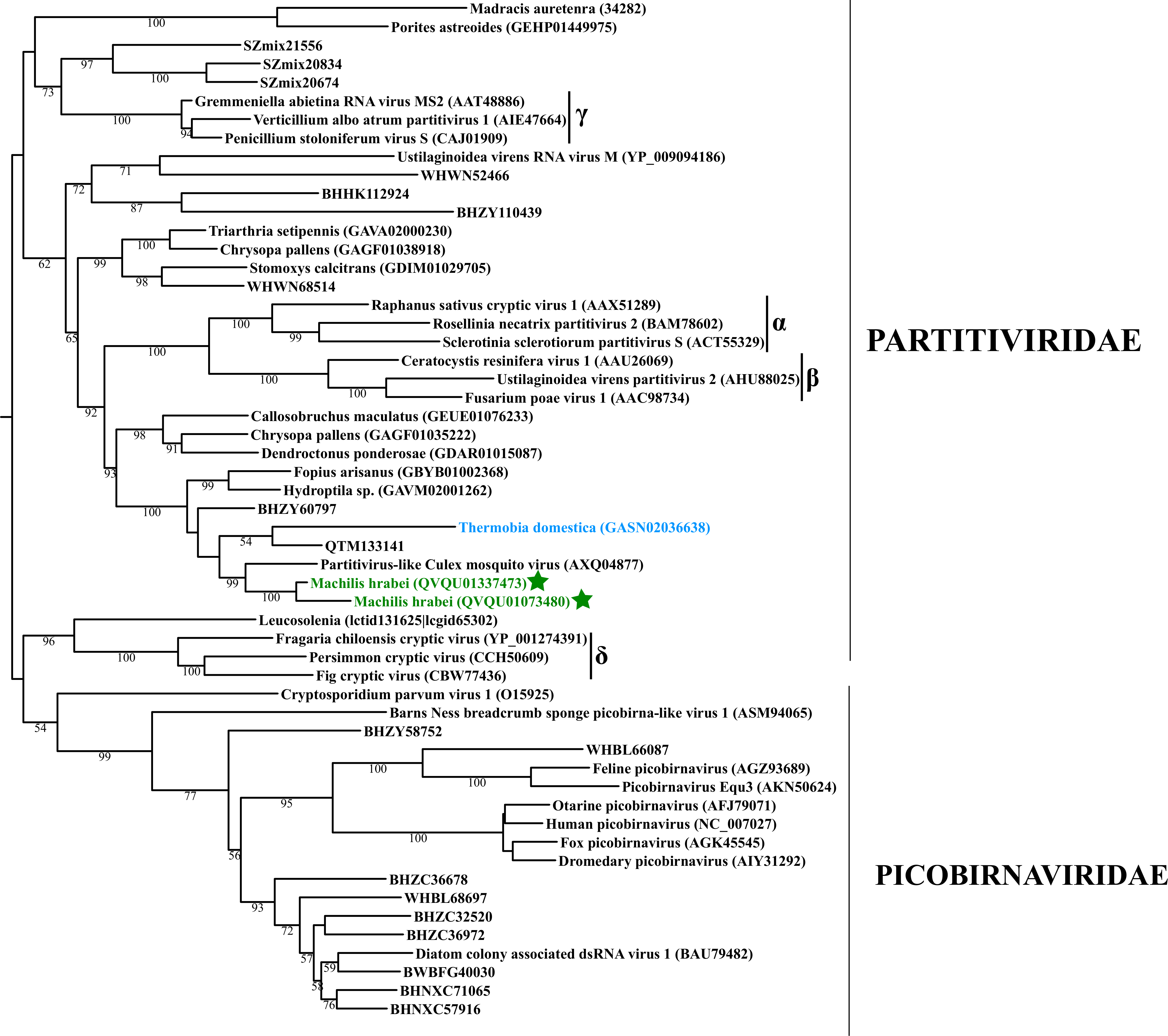

Supplement: Figure S2 — Each phylogeny provided in this and the other supplementary figures is based on the analysis of the viral RdRP domain. The names of the viruses are marked with different colours based on their host taxonomy; springtails (Collembola) are red, Diplura are violet, Monocondylia are green, and Zygentoma are blue. The star symbol denotes host taxa that contain endogenous virus copies (EVEs). The best fit model of amino acid substitution for this data set was determined as Blosum62+I+G4 according to the Bayesian information criterion. Most sequences were obtained from the GenBank; species names and accession numbers are included. Sequences from the Shi et al. (2016) have the same unique accession numbers as in the original publication. The best fit model of amino acid substitution for this data set was determined as Blosum62+I+G4 according to the Bayesian information criterion. [file peerj-08-8336-s002.png]

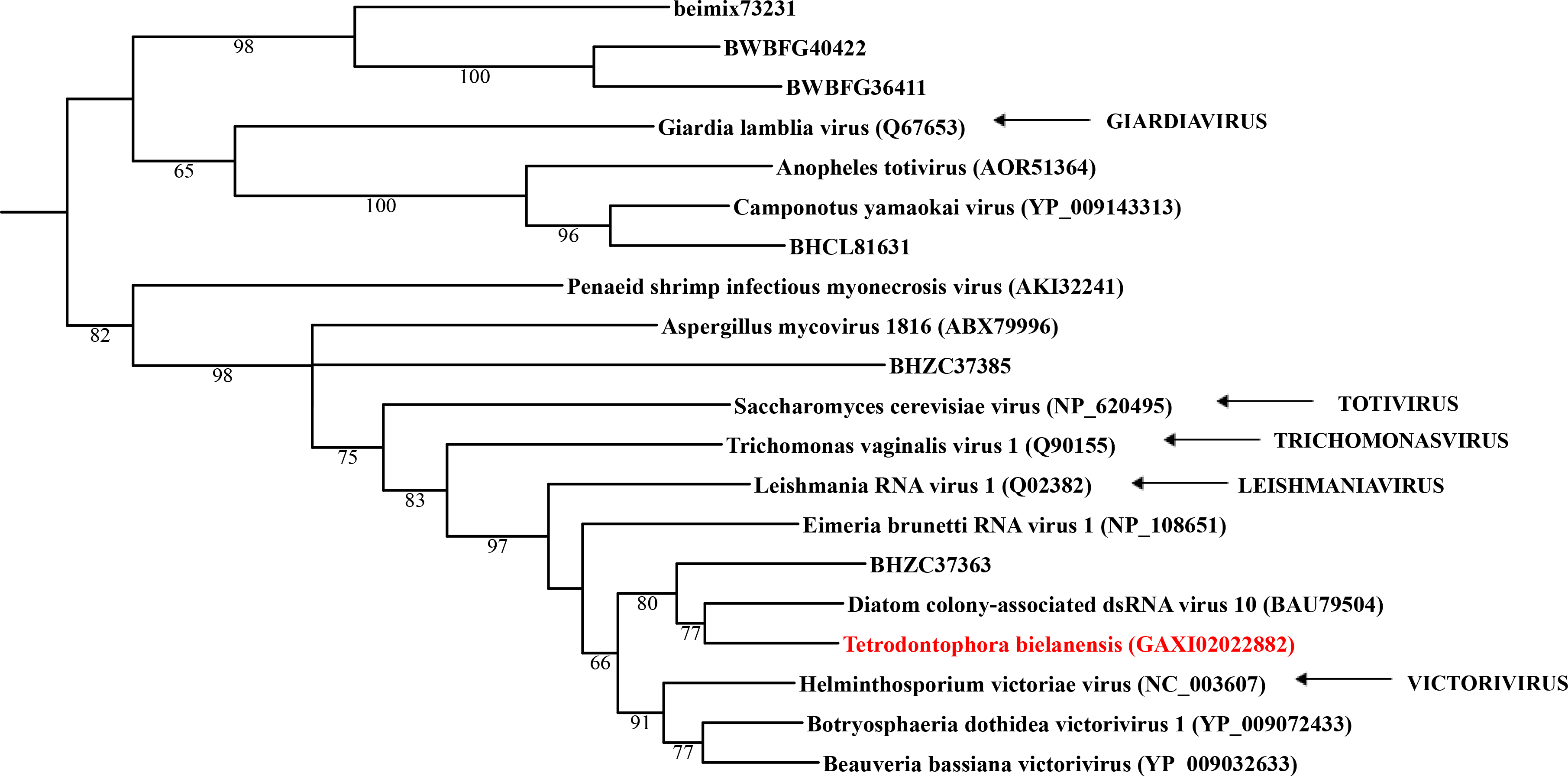

Supplement: Figure S3 — Each phylogeny provided in this and the other supplementary figures is based on the analysis of the viral RdRP domain. The names of the viruses are marked with different colours based on their host taxonomy; springtails (Collembola) are red, Diplura are violet, Monocondylia are green, and Zygentoma are blue. The star symbol denotes host taxa that contain endogenous virus copies (EVEs). The best fit model of amino acid substitution for this data set was determined as Blosum62+I+G4 according to the Bayesian information criterion. Most sequences were obtained from the GenBank; species names and accession numbers are included. Sequences from the Shi et al. (2016) have the same unique accession numbers as in the original publication. The best fit model of amino acid substitution for this data set was determined as PMB+I+G4 according to the Bayesian information criterion. [file peerj-08-8336-s003.png]

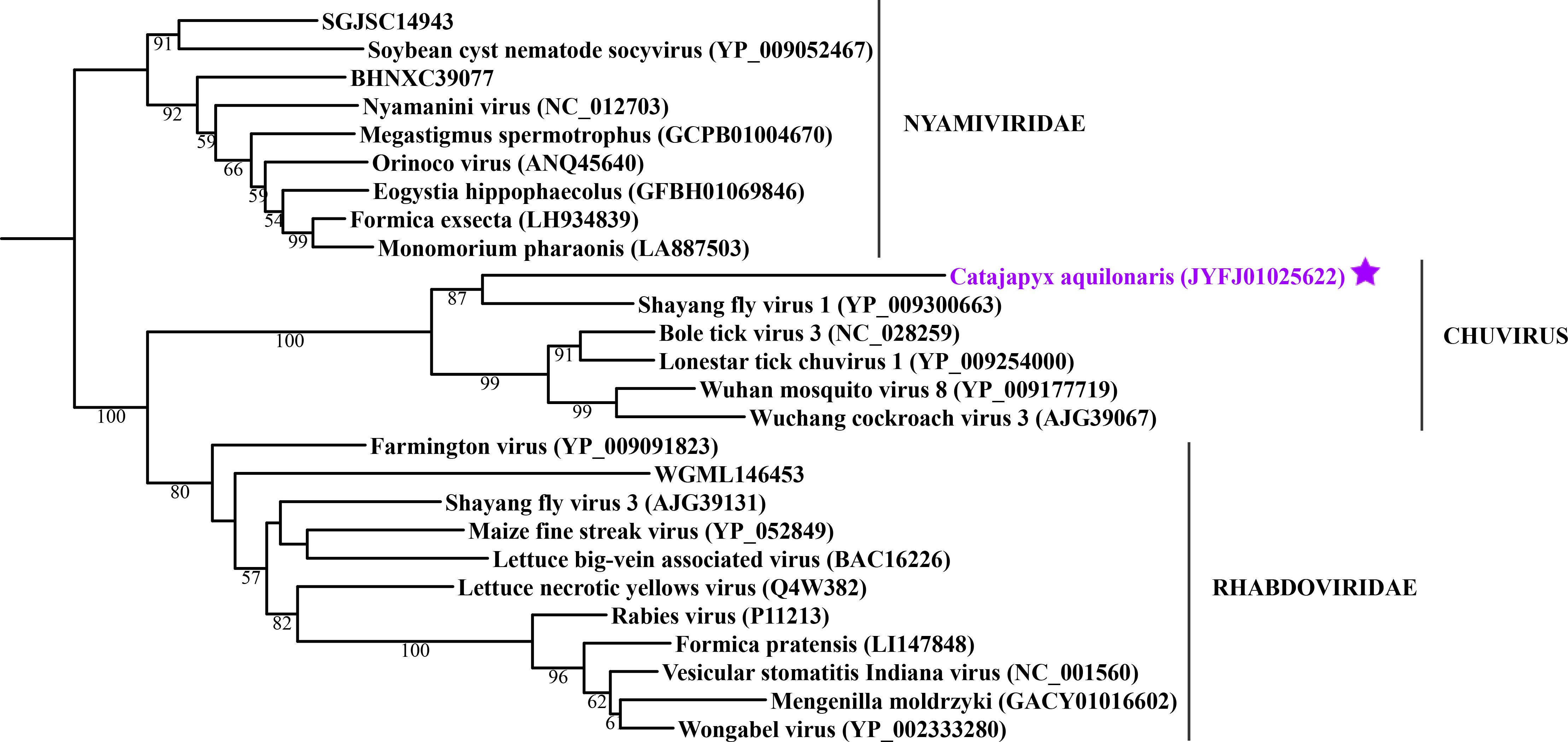

Supplement: Figure S4 — Each phylogeny provided in this and the other supplementary figures is based on the analysis of the viral RdRP domain. The names of the viruses are marked with different colours based on their host taxonomy; springtails (Collembola) are red, Diplura are violet, Monocondylia are green, and Zygentoma are blue. The star symbol denotes host taxa that contain endogenous virus copies (EVEs). The best fit model of amino acid substitution for this data set was determined as Blosum62+I+G4 according to the Bayesian information criterion. Most sequences were obtained from the GenBank; species names and accession numbers are included. Sequences from the Shi et al. (2016) have the same unique accession numbers as in the original publication. The best fit model of amino acid substitution for this data set was determined as LG+I+G4 according to the Bayesian information criterion. [file peerj-08-8336-s004.png]

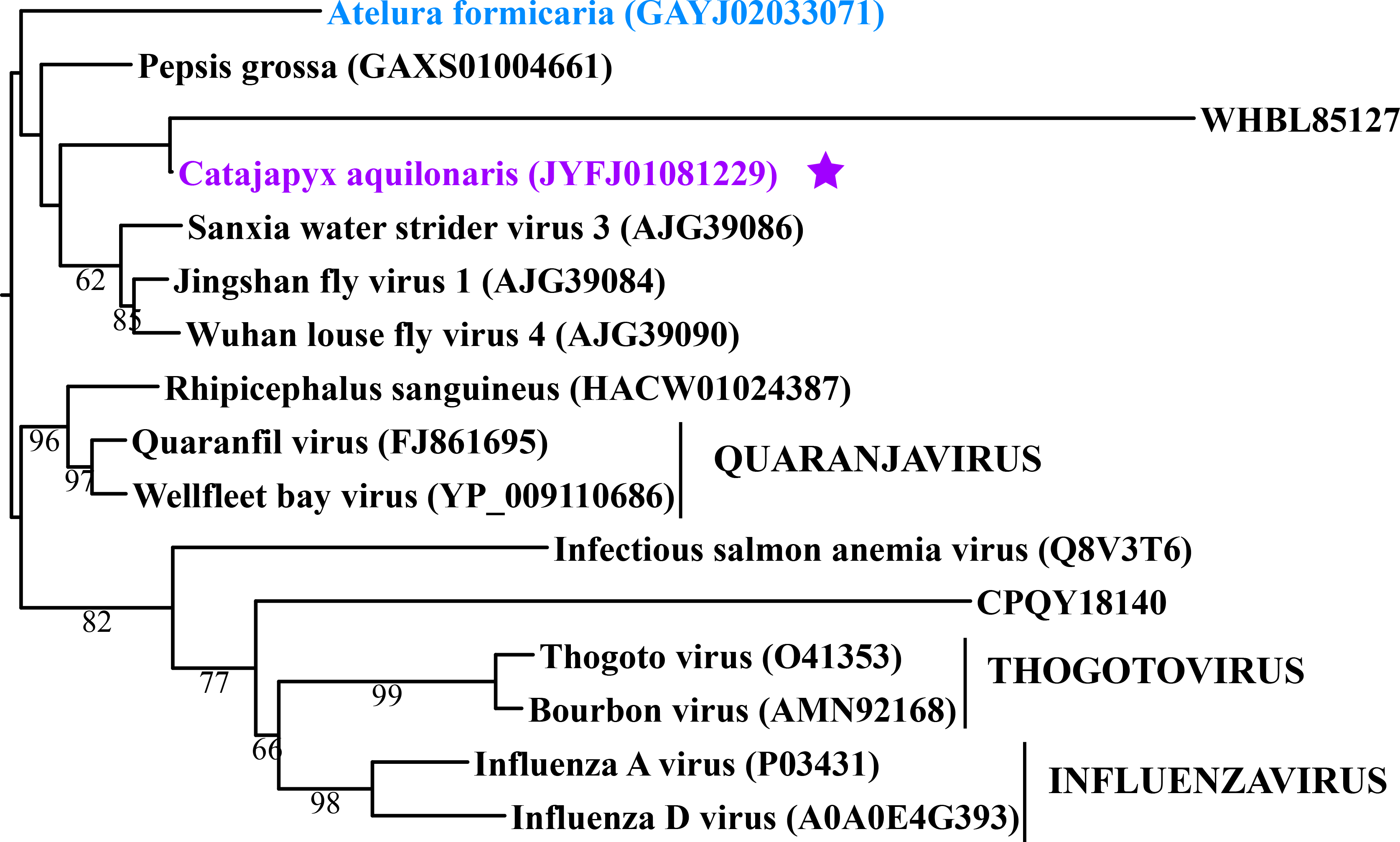

Supplement: Figure S5 — Each phylogeny provided in this and the other supplementary figures is based on the analysis of the viral RdRP domain. The names of the viruses are marked with different colours based on their host taxonomy; springtails (Collembola) are red, Diplura are violet, Monocondylia are green, and Zygentoma are blue. The star symbol denotes host taxa that contain endogenous virus copies (EVEs). The best fit model of amino acid substitution for this data set was determined as Blosum62+I+G4 according to the Bayesian information criterion. Most sequences were obtained from the GenBank; species names and accession numbers are included. Sequences from the Shi et al. (2016) have the same unique accession numbers as in the original publication. The best fit model of amino acid substitution for this data set was determined as LG+I+G4 according to the Bayesian information criterion. [file peerj-08-8336-s005.png]

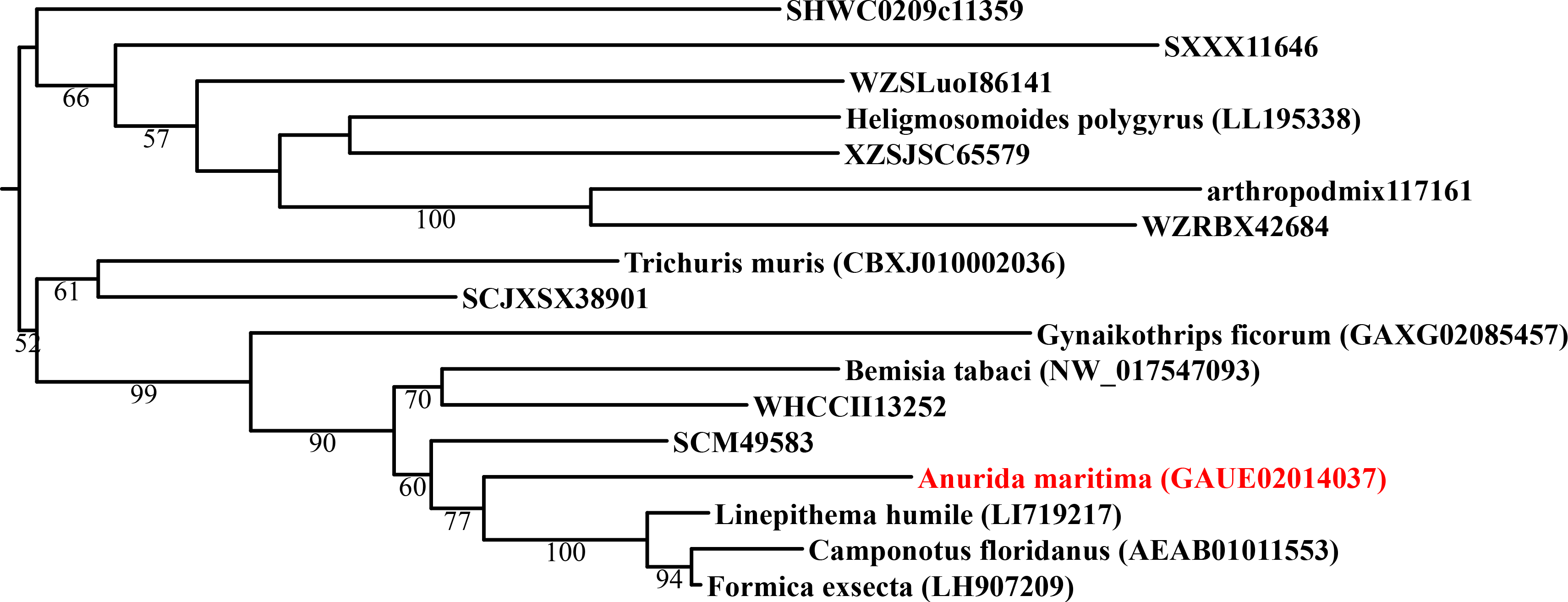

Supplement: Figure S6 — Each phylogeny provided in this and the other supplementary figures is based on the analysis of the viral RdRP domain. The names of the viruses are marked with different colours based on their host taxonomy; springtails (Collembola) are red, Diplura are violet, Monocondylia are green, and Zygentoma are blue. The star symbol denotes host taxa that contain endogenous virus copies (EVEs). The best fit model of amino acid substitution for this data set was determined as Blosum62+I+G4 according to the Bayesian information criterion. Most sequences were obtained from the GenBank; species names and accession numbers are included. Sequences from the Shi et al. (2016) have the same unique accession numbers as in the original publication. The best fit model of amino acid substitution for this data set was determined as LG+I+G4 according to the Bayesian information criterion. [file peerj-08-8336-s006.png]

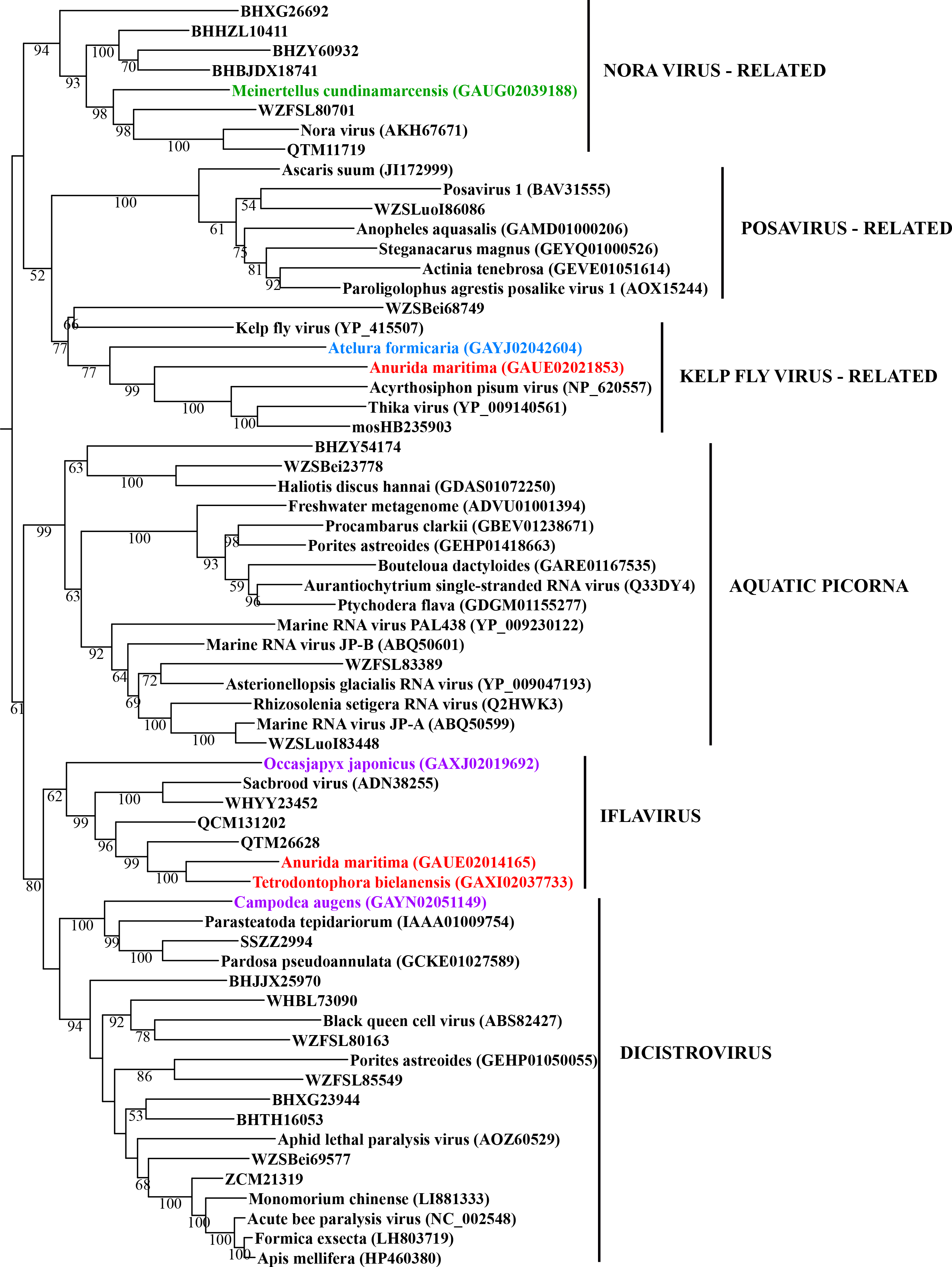

Supplement: Figure S7 — Each phylogeny provided in this and the other supplementary figures is based on the analysis of the viral RdRP domain. The names of the viruses are marked with different colours based on their host taxonomy; springtails (Collembola) are red, Diplura are violet, Monocondylia are green, and Zygentoma are blue. The star symbol denotes host taxa that contain endogenous virus copies (EVEs). The best fit model of amino acid substitution for this data set was determined as Blosum62+I+G4 according to the Bayesian information criterion. Most sequences were obtained from the GenBank; species names and accession numbers are included. Sequences from the Shi et al. (2016) have the same unique accession numbers as in the original publication. The best fit model of amino acid substitution for this data set was determined as Blosum62+I+G4 according to the Bayesian information criterion. [file peerj-08-8336-s007.png]

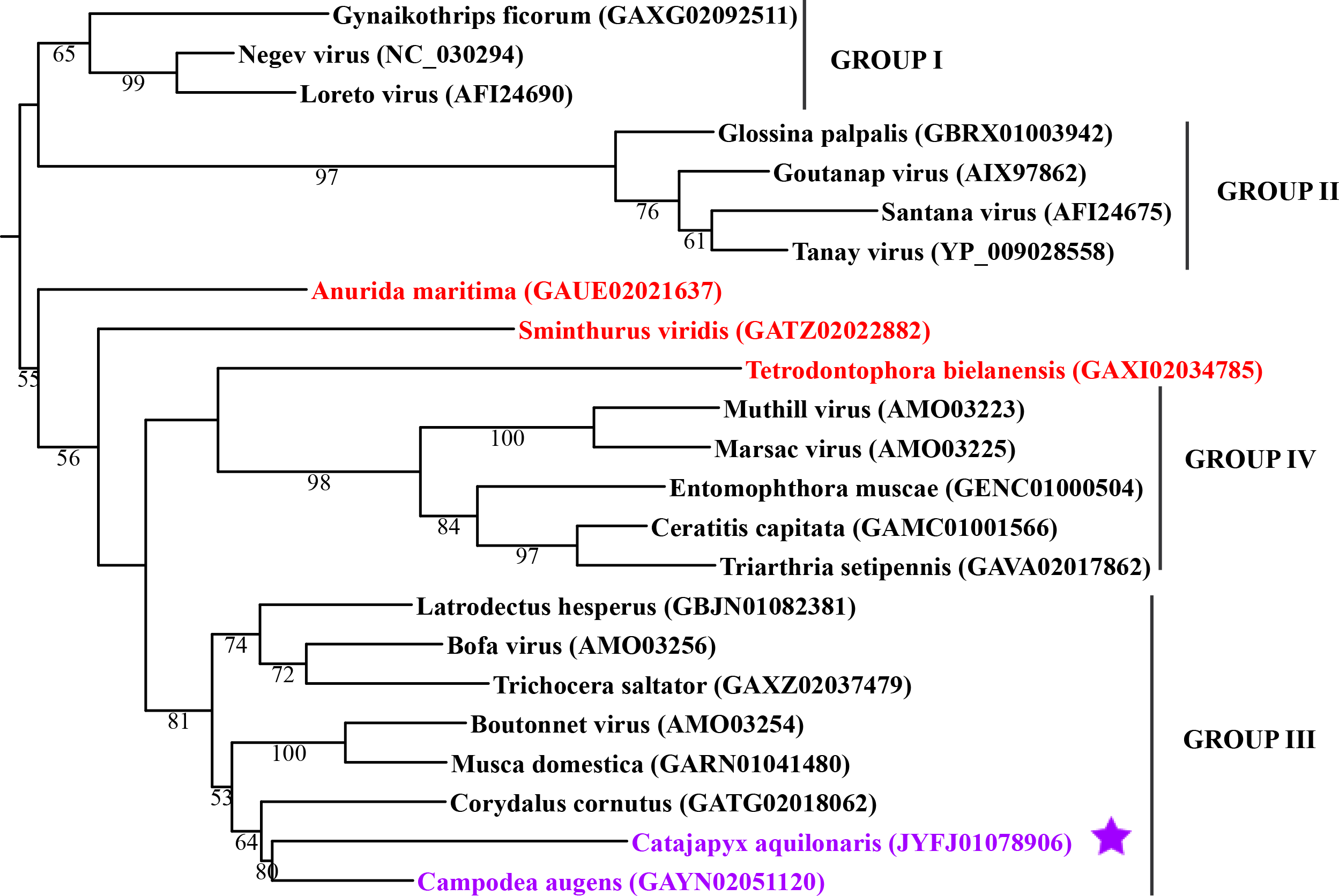

Supplement: Figure S8 — Each phylogeny provided in this and the other supplementary figures is based on the analysis of the viral RdRP domain. The names of the viruses are marked with different colours based on their host taxonomy; springtails (Collembola) are red, Diplura are violet, Monocondylia are green, and Zygentoma are blue. The star symbol denotes host taxa that contain endogenous virus copies (EVEs). The best fit model of amino acid substitution for this data set was determined as Blosum62+I+G4 according to the Bayesian information criterion. Most sequences were obtained from the GenBank; species names and accession numbers are included. Sequences from the Shi et al. (2016) have the same unique accession numbers as in the original publication. The best fit model of amino acid substitution for this data set was determined as LG+F+I+G4 according to the Bayesian information criterion. [file peerj-08-8336-s008.png]

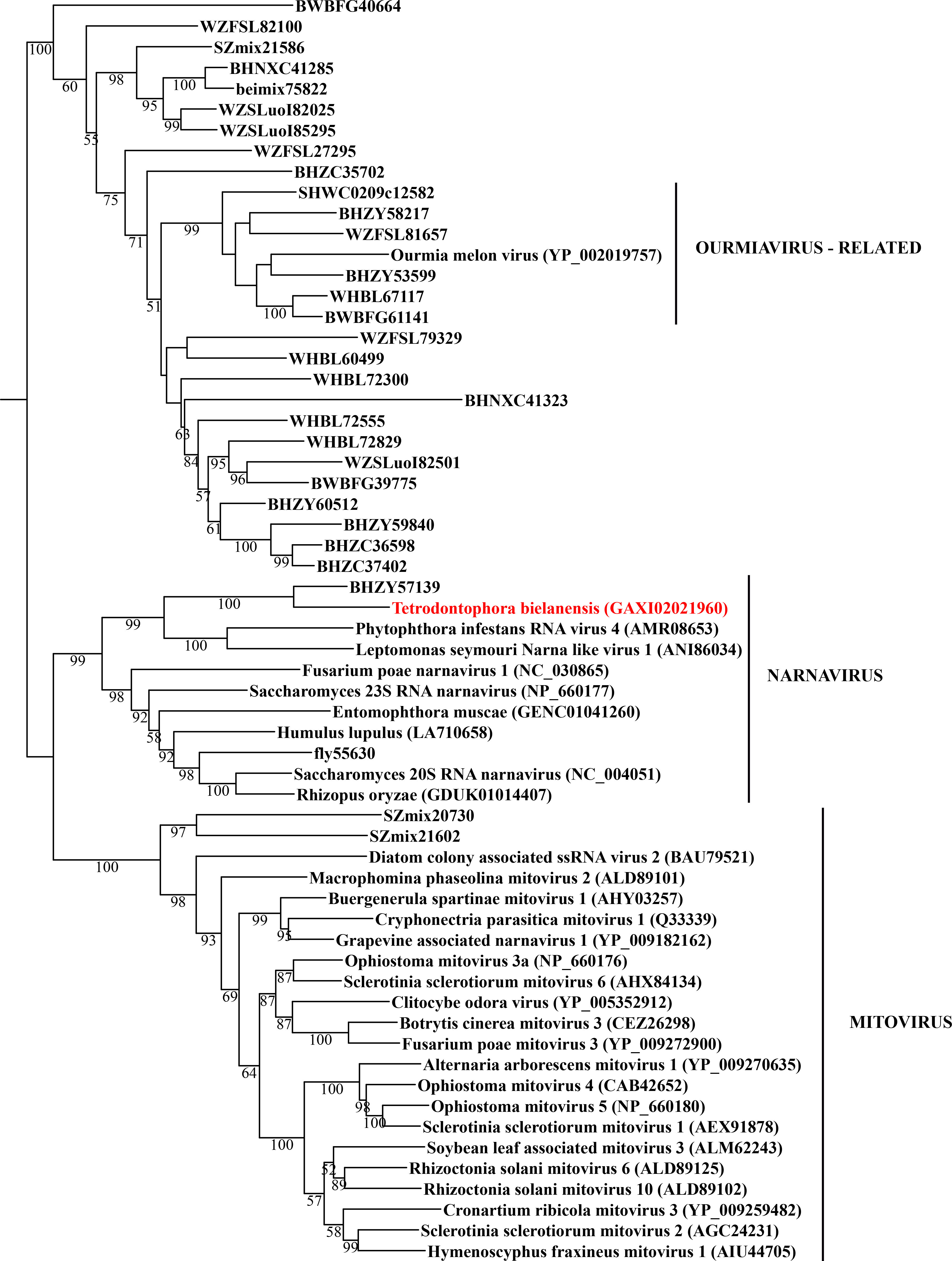

Supplement: Figure S9 — Each phylogeny provided in this and the other supplementary figures is based on the analysis of the viral RdRP domain. The names of the viruses are marked with different colours based on their host taxonomy; springtails (Collembola) are red, Diplura are violet, Monocondylia are green, and Zygentoma are blue. The star symbol denotes host taxa that contain endogenous virus copies (EVEs). The best fit model of amino acid substitution for this data set was determined as Blosum62+I+G4 according to the Bayesian information criterion. Most sequences were obtained from the GenBank; species names and accession numbers are included. Sequences from the Shi et al. (2016) have the same unique accession numbers as in the original publication. The best fit model of amino acid substitution for this data set was determined as VT+F+I+G4 according to the Bayesian information criterion. [file peerj-08-8336-s009.png]

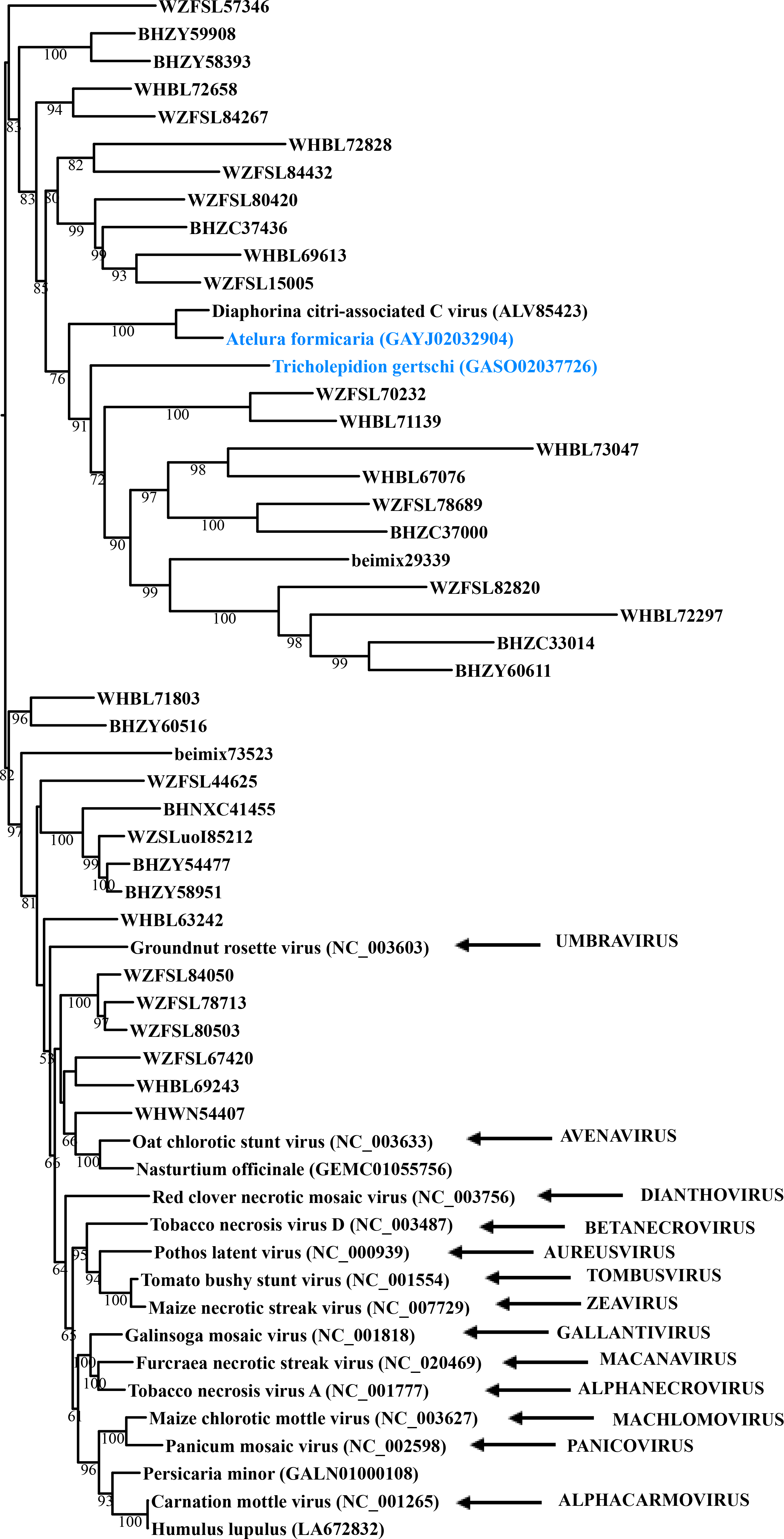

Supplement: Figure S10 — Each phylogeny provided in this and the other supplementary figures is based on the analysis of the viral RdRP domain. The names of the viruses are marked with different colours based on their host taxonomy; springtails (Collembola) are red, Diplura are violet, Monocondylia are green, and Zygentoma are blue. The star symbol denotes host taxa that contain endogenous virus copies (EVEs). The best fit model of amino acid substitution for this data set was determined as Blosum62+I+G4 according to the Bayesian information criterion. Most sequences were obtained from the GenBank; species names and accession numbers are included. Sequences from the Shi et al. (2016) have the same unique accession numbers as in the original publication. The best fit model of amino acid substitution for this data set was determined as LG+F+I+G4 according to the Bayesian information criterion. [file peerj-08-8336-s010.png]

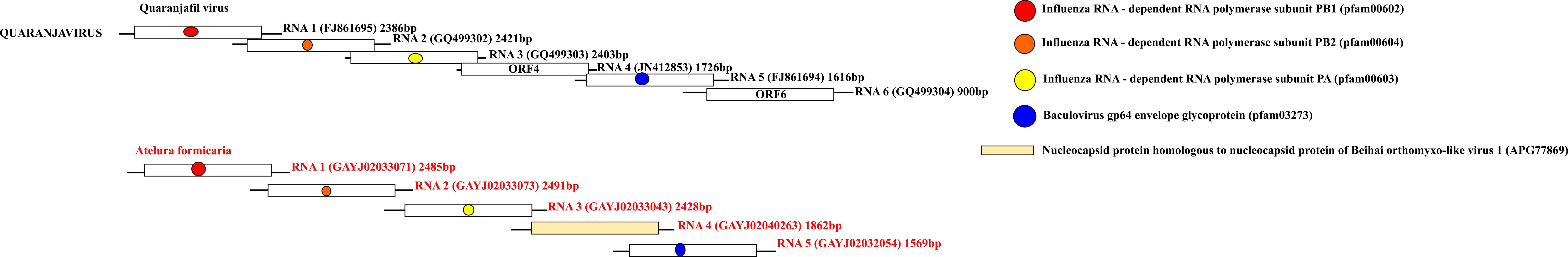

Supplement: Figure S11 — Within each genome, the outer boxes define the ORF boundaries, while the inner boxes define regions with blast matches to a viral protein or a protein domain whose detailed information is provided in the upper right corner of the figure. Each major homologous protein/domain among different genomes is marked with a different colour. [file peerj-08-8336-s011.png]

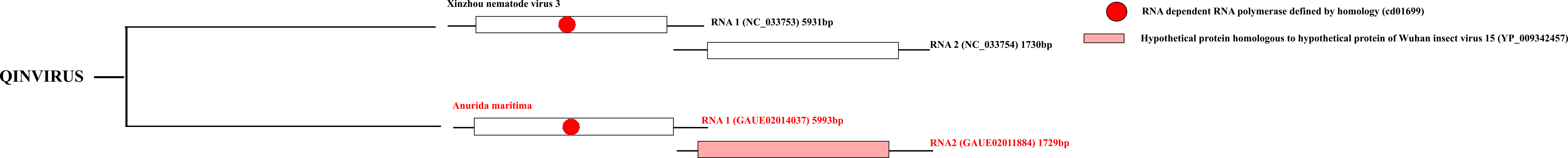

Supplement: Figure S12 — Within each genome, the outer boxes define the ORF boundaries, while the inner boxes define regions with blast matches to a viral protein or a protein domain whose detailed information is provided in the upper right corner of the figure. Each major homologous protein/domain among different genomes is marked with a different colour. [file peerj-08-8336-s012.png]

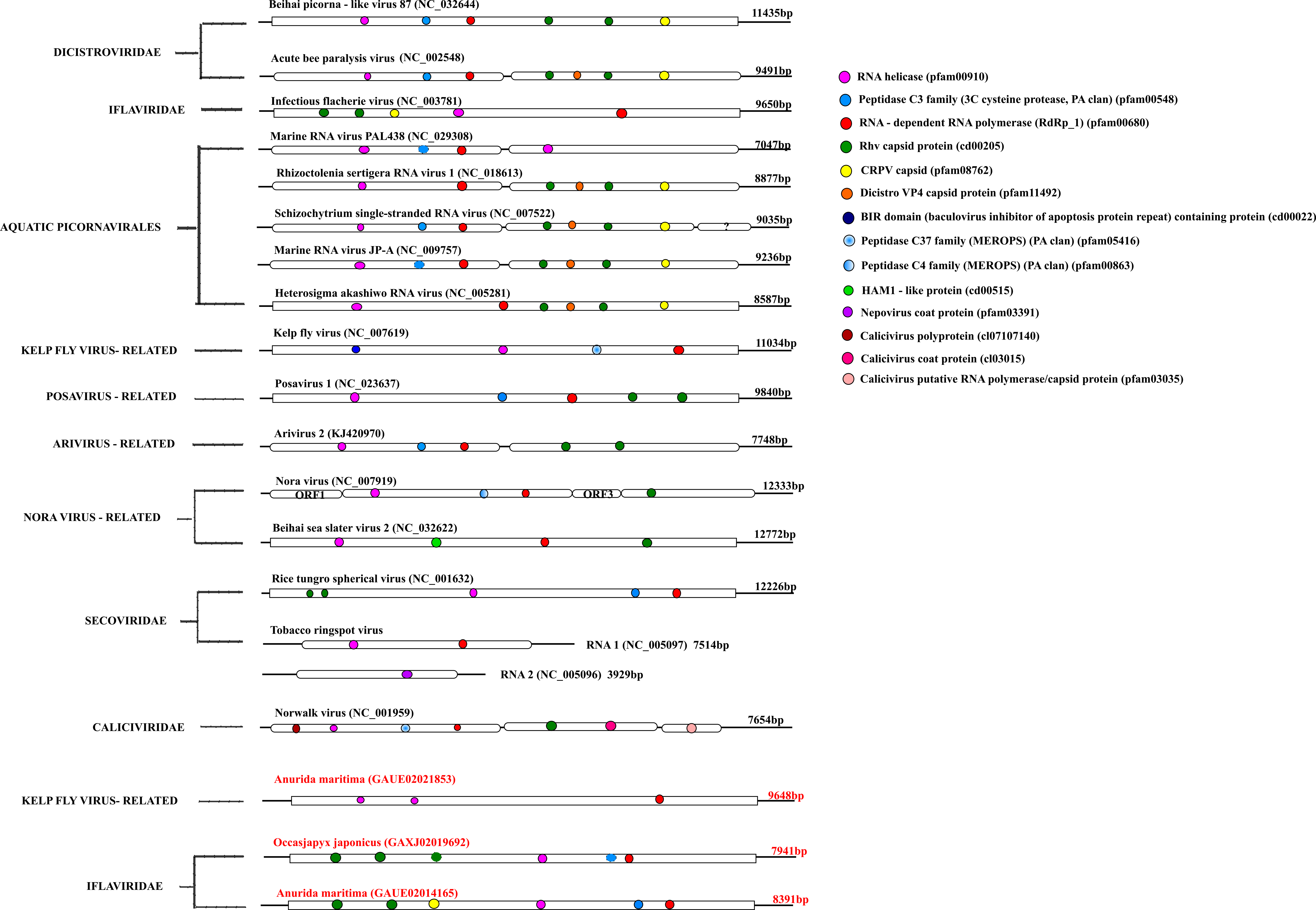

Supplement: Figure S13 — Within each genome, the outer boxes define the ORF boundaries, while the inner boxes define regions with blast matches to a viral protein or a protein domain whose detailed information is provided in the upper right corner of the figure. Each major homologous protein/domain among different genomes is marked with a different colour. [file peerj-08-8336-s013.png]

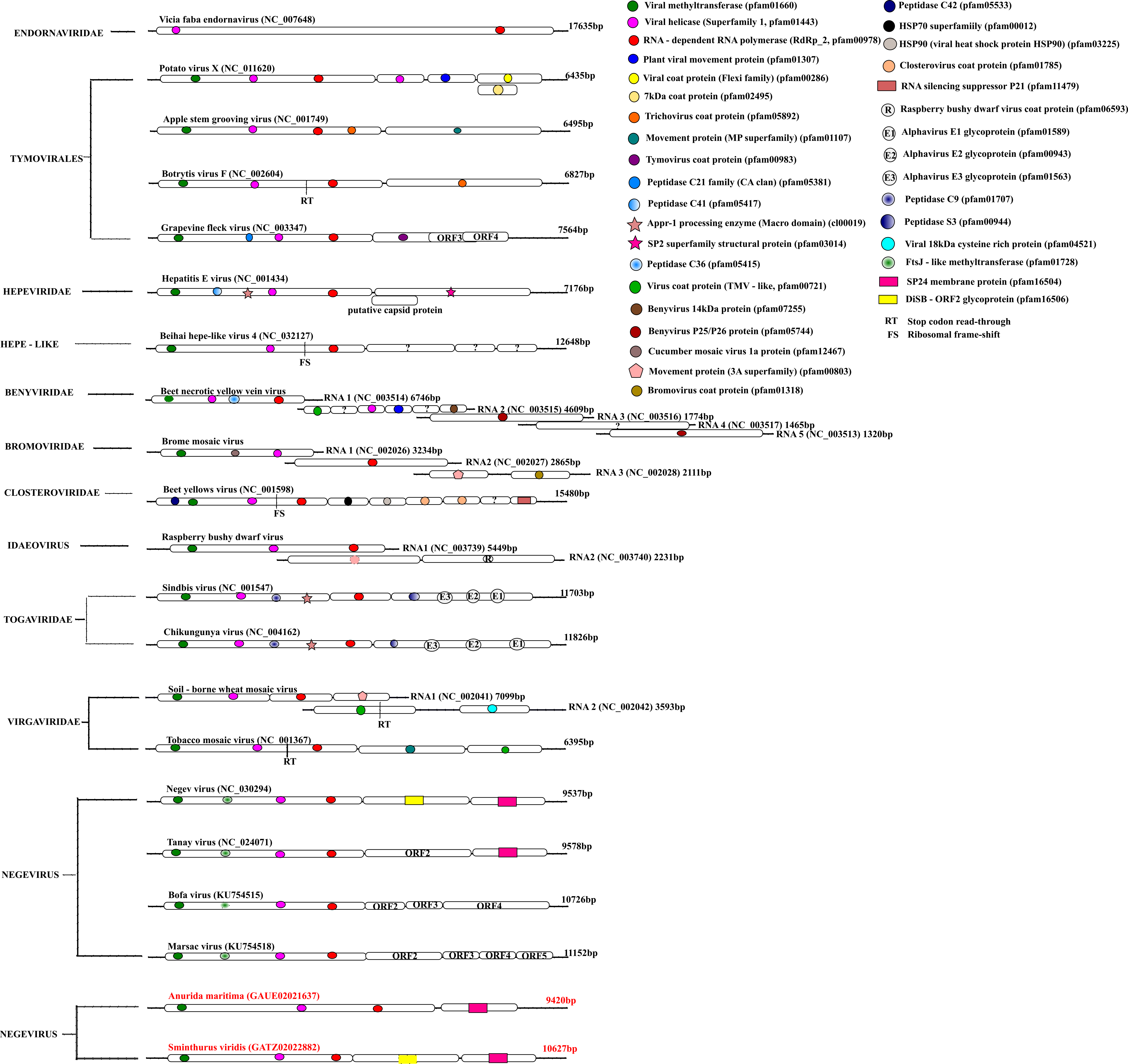

Supplement: Figure S14 — Within each genome, the outer boxes define the ORF boundaries, while the inner boxes define regions with blast matches to a viral protein or a protein domain whose detailed information is provided in the upper right corner of the figure. Each major homologous protein/domain among different genomes is marked with a different colour. [file peerj-08-8336-s014.png]

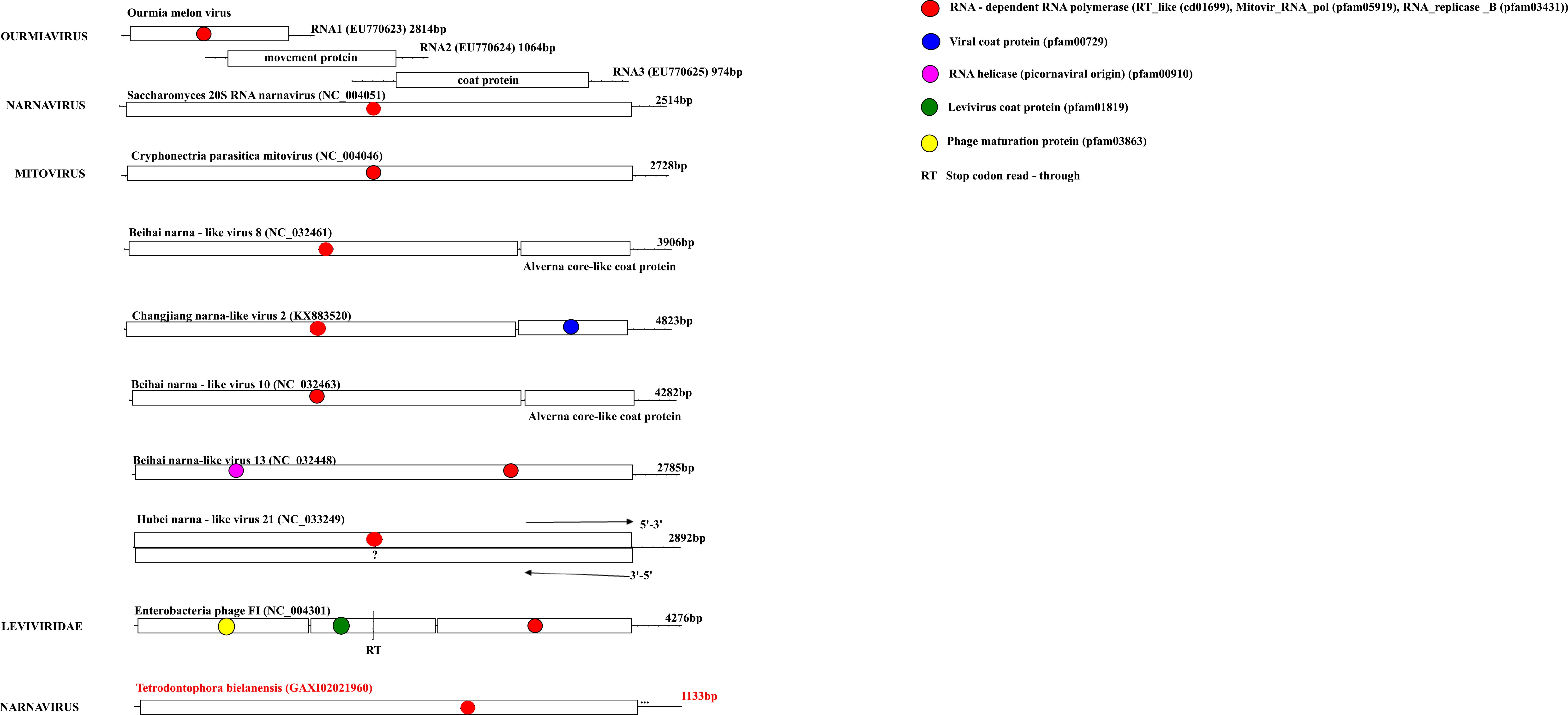

Supplement: Figure S15 — Within each genome, the outer boxes define the ORF boundaries, while the inner boxes define regions with blast matches to a viral protein or a protein domain whose detailed information is provided in the upper right corner of the figure. Each major homologous protein/domain among different genomes is marked with a different colour. [file peerj-08-8336-s015.png]

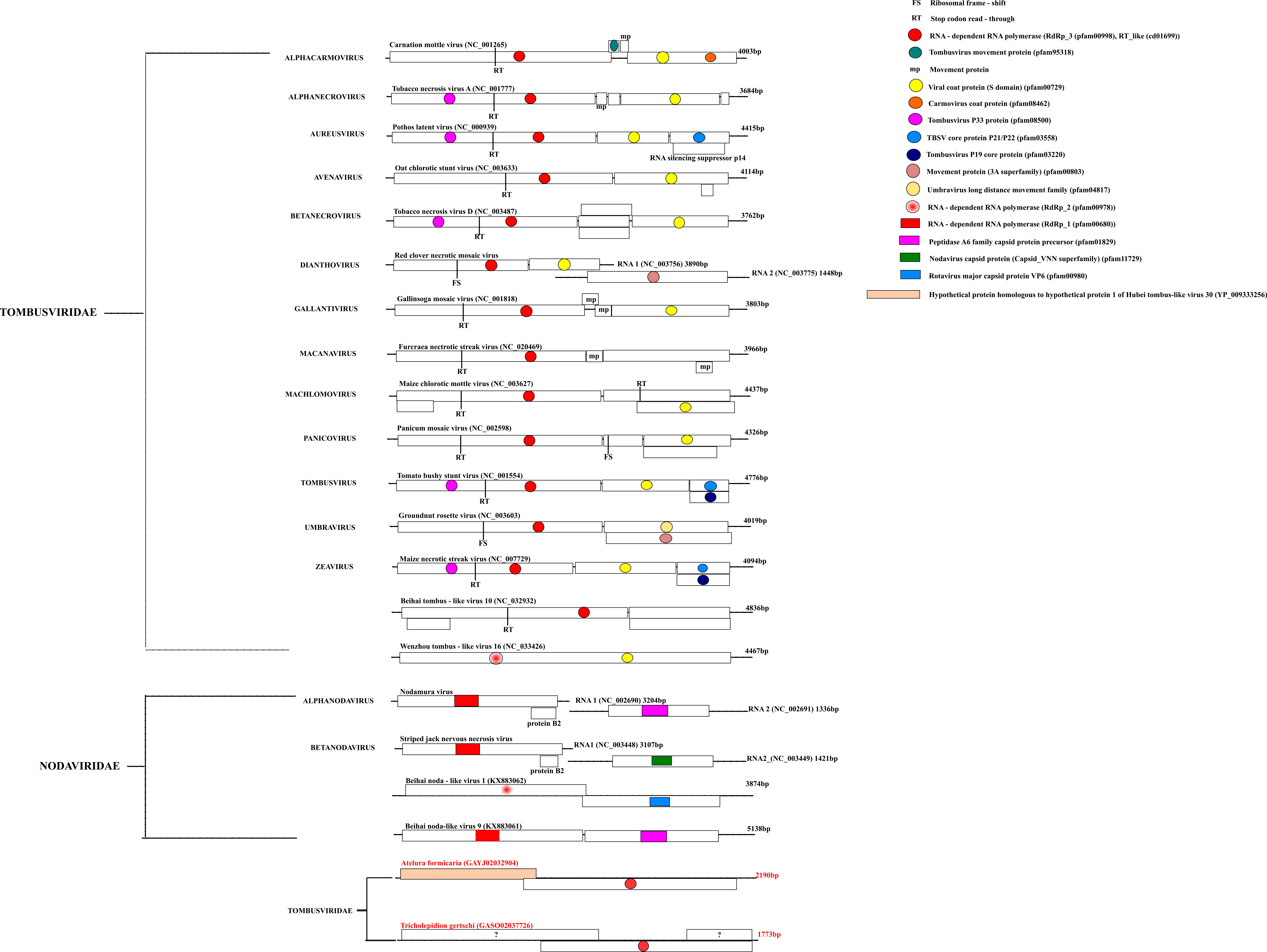

Supplement: Figure S16 — Within each genome, the outer boxes define the ORF boundaries, while the inner boxes define regions with blast matches to a viral protein or a protein domain whose detailed information is provided in the upper right corner of the figure. Each major homologous protein/domain among different genomes is marked with a different colour. [file peerj-08-8336-s016.png]
