# Supplementary material for: Analysis of the RNA virome of basal hexapods
Source: PeerJ. 2020 Jan 9;8:e8336. doi: 10.7717/peerj.8336 (PMC6955108; doi:10.7717/peerj.8336)
Supplement: Data S1 [file peerj-08-8336-s017.docx]

>GAUE02014037.1 TSA: Anurida maritima C99386_a_54_0_l_5993 transcribed RNA sequence QINVIRUS RNA1 segment

CGAACACCAAGAAAATTGGAAAATGTTACGTAATACATGAAAGAGGCAAATTTATTTTAATTTATTCCTA

CTTTTTAACCTTATTTTTCTTTGTCTTCATCTGATGCAGGTTTTTATATACAGAGAAAATGGAAAATATT

TACAAATCGATTATCTCACTGACACAAGTTAATCAAGGTTAAGCATCAATATGCAGCCCACCTCCAGTGT

CAGTAATTAGTTCAGCTCTTTGATCTCTAATTGATTCTAGATCAAAGATGTAGTCTTCTTCACTGTAGTC

AGAGATGTCAATCTTGTCATATGGACATTCAACCACACCATCCAAGGCCTTCTGAATTAGATCAAAATCT

AGTGAAAGTATGATGTCAGCCAGCCACTTGGCTTTCTTCTTATCATTGATCGTTACTTCATCAGGAACTG

CATCTCTAATTTCCATCTTGAACTCTGTGAGTTCTTCTTCTTGTAGGCCTAGAGGATCTAAAATACCAGG

AGTTGTTTTCTCAGGGCTCATCAGTCGCTCCTTTGGTAGCATAGGAGAGATAAGCTTGACAGAGAAATCG

GCTATAATCTCAGTAAGAACATCCATGATTGACACACTAACAGTTGGTATATTATTTACACTTAGGTAAT

CATGTATTTCCTTCCATTCATCATGCCTTTGTTCAGAGTTCCTAAGCCCACTTACCGTGAATTTTGGAAG

TTTTGATGTTAACTCATTTCTTGGAACTTTCTTCTTCTTTTTATGAGGTGGAAGATAAGCCTTTGACTCA

GTTGTGTTACCATGCATAGCTCTTTGTGTAGACATAAGAATTGCAGCATTCATAGCATTGCACAAATCTT

TGTAACTTGTTGCAGACAATACGCATTGTTTGGCTTTGTCATAGAGGTGCCAGGAATAAAGATACGTCAG

AATTGTAGAATCAGGTGGTATGAACTCTTTGTTTCTGCTTCCACAATGTTCTGCTAATTCCCTACTGAAC

CTGACTTGTTGCCTCTTTGCAGCAAACGACCAAGCACGCTCAATCATCTTTGGATCAGCCCGAGCATTAC

GCATATAGATGTCATGATCTTGGAATTTACCAAGGACTGCATATAGCAAGGTAACAAAAGCTTGTCTCTT

GGTCCACTCTGATGCATACTTGGGATTATGGACAAGCTTTACAACATCTTTAGCATATGTTCCAAGAGTT

GCTAATACGATGTCATCTTTTAGCCTTATTTCTTTGAAAACTTCTGTCATAGCATTCCTTATGCCGCCTA

TTGCGTTATCCAAGGACTGCTCCCAGTTAGGCAGGTACCTATGTATATTCTCAATAAGATACCTAGGTGA

CTTAACATATATGTCCTCATCCAAAGTAGAAGCTCGAGGGATAGAGTCTTGTAGAAGTTCAATCAGGCAT

CGCAAAGCAGCTGAAGTCTTGTCATATCTGTCACTGTAATTGTGAAGCTTAGTGACTGAAGTCTTTCGAT

GTGGGTCTCTAGGTATGTCATAATTCCCTTTGGGTATTCCAGTCTGTTGCTTCTTCTCAAGAGTTAGACC

AAAAGTGGCATGAATAAGGTCCTCAACATTTTCATAATAGAAATGTGAAACATCATCACGCAACTTGTTC

CAAGCAGACTCTCTTTCTTGTGCGTGAGAGTGTGTATACTCAGCAGCTAGAATGACATCACCATCCACCA

TGTCTGTTATTCCTAAGTCTCTATATGGCAATAGATGTTTTGCCATTATGTCAGATAGAGCAGTTCTCTG

GATATTGTAGCTGACTTGGCTAATCCTGTGCAACCTAACCCTGTCAAGTATTTGTAATGGGTCACCAACT

GGGCTGTACATGCTTCCTGGGACTACAGAATAATACAGAAAACCTCTGTAGCCTATTGCTCCTTCGAGGA

GGCCAACTACCTTTGCAGCGGTTATTAATGACATTACATTTCCTGAGCTCGCATAATACTCATACATGTC

AATTAGACTTCTGCCCATAACAACTGCACACCTGTGTGAGTTAGGAAAAGCTGATATGGGGTGTGTTAAG

CTACTTGCCTTCTTCAAGAACCTTTTGAAGCTTACTCCAGGTTTGATTGCAACAGTAGGTGTCTTCATGG

TCGTATCAATAGGTAAGTTCCACATAACACAAAAGATCTTCCATAGTGCGAGTGCAACATTGTTGCAATC

TTCCTTTTTTTCAGCATCTATGCCCTTGTTTGACAGATGTGCAACCACCTGAGTGGCTCGTAATAAGCAT

TTGGCTACAACTGGCAGATTCTTTGCAGTCTGTGGTATGGCACCATACAAAGTTGCAGTGGGTTTGCATG

TTCTACTGAGATCTGGTGAAATACCATCATAAAGATTTGCATATCTTTTGCTATGAGGTCTATCTTTGCA

CTTATTCAGAGGGTTCTTAATCTCAACAATAAGACCAATCTTAGGCTCCAATGTCCTTCTTGATAACATC

GTTCCAGGGTCCACAACTGTGCAGTTAGGAACTTTATAACCAGACAAGGCATGGTATGATGCTCTATAAC

TTATCACCTCTTCAAGATAATTCTCGAATGTCAGATCAATATATGGAATACTTCTAGATGTTGTTATTTC

AATTATCTTGTCCAGGATTTTAGCATTTGAGCCCTTAACTCTGCTCACAAGATCTCTCCTTATTGAGAAT

GGGAATAGAGTGAACAGCAGTTCCCCCTTGTGTGCCTTGTCAACTAATCCTCTTATGCACTGCTCAGGAA

ATGATGCACTCATGGCTTCTAATATACCGCCTGGTACAGAAACAGAAGACAAGAAAGCATGAAGGAGAGG

CTCAGCTCTTAGAGAGTCAGACATGTCAAAAGCTTCAGAGATTGCTGGAGACTTGCATTTTAAGCGCATT

CTAGCCTTAAGTGTCCTGTATACGTTATCATCAGGGCTTATGCGGTCAGCAGCATAAACAGCCATTGGGT

CTTTGAAGACAACTGTTGCTGTAGGTGGCTCCAATTTCTGGTTCACATAAGCAGAAAATGAGTAAATCAA

ACTATTTCTTATGTGAACATTGCTAATGAGATTTTTCAGTCTAACGTGAACACCAAAGAACATTGCTAAT

GCATCAACCTTCTCTTTTGTCATAAAGGCAACAATATGAGGCATTCCAAGTCCGTTAAGCGATCGTGGTG

CAAAGGCAGCAGTTACTAGGTATCCTAGGTTTGGTATTATCTCCTTTATGATTGCGTTAGACTGAATCAT

AAGATCAAATGACCTAAAGAAATACCAGTAGTAACACAGCATAGGGTCTGAGCCTTTCATAGCGGATGAG

CGATACCCTCCCATAACAGTGTCTGAGGCTTGAAAGAATGTTGACAAGCGTCTGTTGTAATCTCTGTCTA

CCTTAGAAAAAGTCTTCATATCAACAAGAACCTCACTACCTTCGCAGAAAAATCTGTTTAAGTATATAAA

CTTAATGGATGAGTAGATAGTCTTTGACTCACTGAGTGATGCACCTAGCTTTCCCCAAGTCTCTCTGGTT

GTCTCATAATGCTTATTGGCAGCATTCTGTGCAAAAGCTTTTGTTGTTCCATCTGGTAAAGAGAATGATT

GTACAGCATCATCTATAAGCCCAGCAGTTGTAGCTCTGGTTTGTTCAGGTAGATATTTGGCTTCTTTTCC

CTTCCTAACACAGTAGGTGCTAATACTTACATTAAAAAGGGAGTCAGCAGTGCCTGTCCAACCCTGAAAT

AATCCACCCTTAAGCTCATCATGTGCCAAAAATCCTCTCTTAGCCATCCCAGCATGTATACCATCTAGAA

GCTTGACAAGTTCGAAGCCATCACCCAAGCTAGTTAGAGAAACTTTGTAGTCATGATGCTGAGCCCATGC

TTCTCTAGGTGCACTAGGACTCCATCCAGAAGTGTCATTAGATATTATGATGTTGTAGTTCTTGGCAACA

CAACTTCTGTTTACCTTGTCAAATATCCCATCAACATGAACATTGCTTTTCCTAGATGTTACACCATCCA

CCATATGTGACAATCCAATTAAGCTGTGGTCATAGTCTGTTAGAAGTTCCCGAGTTATGGCATCAGCTGA

CCATGTCTCTCGCATCTTCGGGCCATATTTGGTATTTTCAGACTTTACAGACATGTCTGCGATCTTTGGA

AGATTTAAACTGCCATTGCGAACACACTCAAGCACTAGCTCTGGGCTCCAACCTGGCTCAATGTCTGGTG

CATGCATGAGAGTGTAAAGTAACTCGTTCACCTCCATTGGAACCTGTGAAGATTTCTCAACTAGGCTGTC

ATATTTAGATATGTCAGGAACAACTCTTGTAACATCAGCAGAATTGAGATACCAAGTATGTATAAGTAAA

TTCACAGGTACCACATTTTTGATCCAACACAGACCCCATTCATGTTTAGGTGGTAACATGAGCTTCCCTT

GTGCACACGACTTGTACCATTTCTTCACAGATGGATCATATCCATCTTTACAGTGGAGTTTTACCTTTCC

ATGACTCTTGGTGACAAACATGGAGACATCTGTTGCTTTGCAATAGTTCAAGAATGTTGCAAACTCCTCA

ACATCTTCCTTGTTGGAGTCATTTAGCTGTGTGACAACTCTTTCATAGACCTCTTTTGGAGGAGCATCAG

GAGAAGGAAGAATGTGGTACAAATATGCCAAGTTAACTTTGTCTCTTTCAGGGATGTCTAGATCAGACAA

GAAGCTGTACCATTCTATCTTTAGGTCTGCAATGTCTTTTTGCTCTTTCCATAACATGTCATTCCGGTTT

CCCAGGCCCATTTCCAGCTTTTCTGCGTGAGAATGGAAGTTGTTCTGTAGTATTGCCAGTCCATGTTTCA

TACTCTTTGCAAGAACTCGTTTGTCTTTGATAACTGACATGGCATTGATAAGCCAGCTAATAGAACCTCT

GTATGCTTCTTCAAACATCTTGGTCCTGTTGTTATCATCACCAAGTAGCCTGAAATTCATAGCATACTTT

TCCAGACCCTCCCATGTACGCATGACAGCCCGAACCATCTCCAAATGTGCAGAATCAAGTATGAATAGAT

CCGATCCATGTTTAAGGAAAGTGAACATTGCAACTTTAAAGATCTTTTCGAAACCCAGATTCATTTCTGC

ATAGTTGTTCTTAACTCGGAAAAGCTTGTAGTGTAGGTAGGAAGAATGCAAATCCTCATGAGCCTCATGC

ACATCTCTTGTACCACCACTAAGTCTAAGCTTTTCCCAAGCTTCAGCAGCATTAGCAGACATCCTGTAAT

CAAATGTCCCTTTTTCATAGCCACGGATATTCCCAATGCTTTGCTTTAGCACTTGTTTACCAATGTTACA

TACAGCACATCCAGCCCAAGTCAGATTTTCAGCTGCAAGTTCAGACATCCTCCACCTCTCAATTGCTTTG

TCAGAAGCTTTAGCCTTGATTTCATACTTAAGATACCTGAGGAGTATCATAAAGACAGCTTCAAAATCAG

TGTAGACATGTACTGGGTTTAAATTCCAAAAGATGTTCTGTATAACATATCCATACCTAGCCAAATTGAG

AAGTTTCTCAGTTGTCTTAACTCTGGTGCCATATGCATAACAATATCTTATGTGCTCGTTCACAAAGTCT

TCCTCATAAGCTGACTGATGGTCGTCATAGCTAGGGATTTGCACATTGCTATGTCCTGCAAGGAATGCAA

TTGTAGAATCTGCGTCAGCTACTATGCTACTCATTATAAGCTTCAGCCTTCTATGTTTAAGCTCAGATAA

AACTTGATCGTCATCCAGTTTCATCTCAGAAGCTAGACCACTGAGCATAACTATAGAATGAGCAGTTTGA

TTTTCGTAAGTTGGTGTTTCGAAGACTCTGGCCATAGTTATATCAGTCAAGAGTAGTGGTACGACAAGAG

AGATGAGCAACAAGCAAGAATAAATATAGATTTGATTTACCAGATGATGAAAAATAATTAGTAAATCTTT

ATCTATATTTATATTCACGTTTAACGCTCACTTAGGAGGTTCG

>GAUE02011884.1 TSA: Anurida maritima C90438_a_59_0_l_1729 transcribed RNA sequence// QINVIRUS RNA2 segment

CACGAACCTCCGAATAAGAGCGTTGTTAGTGAAAACGAACTTTCTATACTTAACGTAATTGCATTTAAAT

CTTGCCTTATTTTACTGTTATATACTTAGAAAGTTCGTAACAGGTCGTTCTGCTCAACGATGGATGCCAT

CTTTACCAACTTGCCAGACATCAATGGATCCATCACTGTTGGTCTTGCTGCAGCTGCTGAGGGGAAGATT

ATTCAGGAGAAATTCCTGCGAATCCTGAAGCTGGACAAGAAAAGGCTAAAAGAGATTCTCAAGGACGAAC

ACGCGCATCAAGCAGCTATTAAAGAAGTTGCTTCTGTTGCATGTGCCTACTTGGCCACTTCTGGTTTTTA

TCTGACCAGGGATCTTCTCATTTTGGCATGCTCAGCTGCAACAGTGGCAATCCAGTGTGGTCTTGTGTGC

TATGAGCTTGGCGACACGCAGGAGCCTGATGGTGACTGGATGTTTGTTGACGAAGGACCAAGTGCTGAGG

ATTTGAAGAAAATTTCTTCTGAAGCAAAAACATGGATCCTCGAGTCTTGGTCTGTAACAAACAGACAGAG

AGCTGCAACTCTCATCGCAGCAACTAAAATCAATTGGTGGAAAACCAATCACCATACAGGACAGGGTGCT

ATCGCAGGCTATGCGTCCAAAGTCTACAAGCAGTTCTTCGGTAACTCTACTGCATTTGTGGACGTGATGC

ACACTATGGGCCATTGGTGTTCTACCAAGCAAATTTTATCTATGCTTGGGATCAAAGGCATCTTGCCGGT

CATTCAAGTTGTGCCAACTACCAACACAGTCATAGCTGCTGATGATGCTAAGATGCGCGTTAAGTCTTCA

CCAGCTGGCACTGCAAAGCTTTTTGTTGCACAGGGACTTGCAAAGCGCATGCTTGGATCAGTTGTTGCTG

TATTTTGTCCTAGTATTTTGGATTACGCAAAGATACCAGGACAAGTAAATGCAGTTATGTACAGCCCAGC

TAAGTATCATGTTGGTTCATTCTACTTGACTGGTGAAGAGAGGGCCATCATTGAGGCAGACCTCTATCTT

GGTAGAGTTGGATCTTTTGGCCAGGTCTTCTTTAAGAACAGCACATTGATGGCAGCTCCATGTGTGGCTA

ATGGTGTCTACAAGAATTATGAAGACTATGACACCGGCTTTGAAGCTATTATGAACTCCTTCAAGGCAAA

ATCTGTTGTTGCTACTGAGTTGGACCCGATTCTCCTTAAGAATGTTTCCACTGGTGCTGGTCTGCAAGGT

GCTGCTAGTGCCTTTGGTGTTGAGGTTAATGCCACTGCTGTTGAGTATATTAAGAAGCTCAAGGAAGAAG

CAGAAGAGGCTGCAAAGAAGGCTGGCAAACCTACTGTTGAGGAGAAACCCCAGCCACCACCGAAACCAAA

GGAGAAGCCTGTGGCAGAGCCTGAAGCAGCTGGACCATCAGCTGATCTGGATCTTTAAGAAGGAAACTTA

AAGAATAATGTAAATAAAGTTCAGATCTCATATGCTAATCAATTTTGTATGAGTGTCTTCTTCAACATTT

ATACCACTTTTCTGGTTCTTTCACTCTATATCTTCTTATTTCACCTTCCTGTCCTGTGCATAAAAACCTG

CATACATGAAAAATCGAAGTAATAATTAGTTGCAAAAGATGTAAGAATAAAAGAAGTCCGTTGTTTTTAA

AGGTACCACCAAGTTAACTTCCGTATAGTTGGGGTCGTTGTTGTTGCTG

>GAXI02022882.1 TSA: Tetrodontophora bielanensis C172417_a_5_0_l_1303 transcribed RNA sequence//Totivirus

CGGGTATGTTGTATACACAACATGTGCTGTGCTACGACCTCCGAGATGGCAGGCATCTGCGTATGGTATG

TAGCCATCAGCTAAAATCGGACGTGACGACGTTCCCCAACATTGTAGGTAGACGTCGCCCCATGGATTCT

CACCAATATATCGGACGAGCTCACGTAAATCACATAATGCTAAAGATTTCTTAAGCATGTGTATGAGTGT

ATACGGGGCCAACAACCCTCTGACTGGTTCAATACTTTGCAGGTCTTCCAGCGACTGGGAAGTCACCACG

TGCATAATGCGCTCAGGCGCCGCGCGTATCACCAATGGATTGGCAGAGTCTACGTTGTACTCCGGTATCG

ACTTTCTAAAACTAGCCAATAGCATTGCACTTTTAACGCCTGCTCCAACCATATCCATGGCTAAACGTTC

GACGTCTGTCACACTTGTACGTAAGTAGACATCTGTCGCAAGCGATGACAGCTTGTATTTCTCCAGCATG

AAGCCGATTTCGCCGTCATTGGCCTTATCTTTTTCGCCTGGCTCTATGGTCCATCTGTCGTATTGGCGCA

TTCTGCCACGAACAGGACCGTCGTTGACTGACGTGCGGCACGATAGTATCTGCTCCGCCCTCGACTTGAC

AATGCCAGTCATCCTGCTGAATGAGCGTACGAGCAACCTGTAAATGTTATCATTGTCACAGCGGTTCATT

AGCGTCCACGCGGATTGTATCATAGTGTGCAGTGCTTCTGCCGGCTTCTGCTTTATCTCGGTGATCCAGT

TGCCAGAAACAACGCTACTTATGGACCTGCACGCGTAACCCCACGCACCCATACGGCCAATTGCTATTCG

CAAGAACTCAGCAGTGTAATACCCTGTACTCTGCTTCATCGGGTTCATGCGCAATGGGCTAGCGCGTACT

GCTGCGAGTAAAGTTGCCGCTGACTGCATTGATGGTGCAGACATGTAGACATCATCGCCGACATGCACAC

TCAACAACGAGTCATAAATGTGCGGTGCGTACACCCTAATGTATGCTGCGTTGAGTATACTATTAAAGAA

ACTCGTACCCCTGTCGCCGGACATCAACGTGCCACGCAACTTACCCATCTTGACACCGCCGTATATCACT

GACCTGCGTTGGAAGCTGCGTAGCAGTCTGTTGCTGAGCGCGATATCATAGCCGGTCCGTTCGCAAACCA

CCCGTGTCACTGTAGCCATGCTAGCGAGCGAGTGCTGCGAGTTGAAGTCATCGTAGTCCAGCATTATGTT

AATAGAGCCTGCTGCTTGCAGTTTTCTTATCCGCCGAACCATG

>GAMM01008132.1 TSA: Orchesella cincta OC8141 transcribed RNA sequence// Totivirus capsid

GGTTGGTACCTCACGTCGCCCAAGATTCAGTAGAAGCTTTGCAAGACGTGGGTACCGATGTAAGTGTTTC

ATTTCTCTTCAGCCTCTGTTCGTTGAACGAGAGGCTTACTAGGGTTGGCAGTGGCCGGAGTGGTACGGTT

GACAGGTGGACCGCGATGTGCATCTGCAGCAAAAACTGGCTCTATAGATTCGCCAGGGACAGTACCAGAA

TTGGACGCGTGTGTACTGTATCCAGGGGCGATCTGCTCACCAGAGTTAGGTTGTAAAGCATCCTCACCAT

GCACATGATCAAGTATGATAGGATGTGTCATATTAGCAGTAGTGAAGGGCACATCAGGCGAGCCTCTTGG

AATACGTTCACGTCCATAATGACGTTTCGGGACATCAATCAAATCAGGAGAGTCGAGTTGCATAGCTATA

TGAGCTGCTTTCATACTAGCATTCGTTCGTGAACGGTATACTTGTGGCGTTTCAACACCGGGTGGCAATA

GACCACAATATTGTGGGATGCTGACACTGATCTCGACTGTACCATTTATAATTTCATCAGTTGTGGGCAT

ATTCCAGGACCGAACGACGTTCCGTTGTGGTGTGAAGTGGTGGAACGCAAGAGTGATGGCTTCACCGACG

ACGAGGGCTTCACCAGGCATGAGTAGACCGTTTGCACTGTGCTGCCACAATAGCTGATGCATGTTGACAG

GGCTGCGTCGTCCAGGATTGACTAATTGATCAACACTCATTTCTTGACAAGCGATGTTGACAAGGCCGTC

ATTTGGGGCATGTAACAAGTGCAGCAGCATAGGGTTGTTTCTAGCGCTAGTATACTGAAGATTGACAACA

GTAGTGAACGTGCCGTTAACCATAACACGCGGGCGCTCGAAACAAGGCATGGTCATATTCTTGTTTGGAA

AAGCGAGAGGGCCGAAACCATGTATGGTAGCAAGATCAGAAGACATTGACTGATGGATGAGTGAAGTGGG

CTCGATCCAGAACCATGGGTTAAGGGTTGGAAAATTGAGATGGCGTGTAGGATGTAGTTGAAGAGTTTCC

GCAGCAACACAGAAGTGTTTAGCACTACCCGCACCATCACCATTAGTTCCAAACAGAAGAGCGAGGTTAC

GCATGTAGTTAGCGGCAAATGCTGCGACAGTTTCCTTAAGAAGATTTGCATTAGAGTACTGTAATCCGAC

GCCGTCGTCAAATGTATTTTCAGAAAGTGTAGTAGGATACATCTCACCATCGACGTCAGTGAGAGGGTCA

GATATTGCAACTGCAGCGGCAGTGCCGAGCGCAATTGAATCAACTGTGTGTCTGAAGTCGCTTGAGGCAA

GTGACTGGGGCATAGGGAAGCCATTGAAAGTAGCATTACCTGCGTCGATACCACCCCAAGGGGGCGAAAA

GCGGCGACGGCGTAATATATCACGCGTGATGCCACCTTCATGTGTGTGAGCGACGACAGTGGTTATCTTA

TGTATACCTCGTGTAACAGCATATGCAAAGTCGGCTCCATAACCGTGACTATCGTAATTTGCCCCCGGCC

GTAA

>GAYJ02033071.1 TSA: Atelura formicaria C264193_a_39_0_l_2485 transcribed RNA sequence//Orthomyxoviridae PB1

CTGGCAGCATTGCTAGACCCAACACGCATTTTGGAGCCGAAGATGAGAAAAAGCTTAAACAGGATATCCA

ACCAATACCAGTATGTGAACCCACCGCCCAGGGCATACGGGTCACCAGTGTTAAGAGTTGGGGAGAGCAT

AACAAGAGCTGAAGGGTTCGACAGGAACAATGCCAAACTGAGGTTCCGAGTAAACGAGGTTCCTTTTCAG

TCTACGGTGCAGCGTCCCGGGGAGATGGGCATCACCCCAAGCCTTGACACTAGTGCCAACTGGAACATAG

GATCCACGTTTGAAGATGCTGCAAAGTATCTTGAGGTTCACGCTGAGGTTATTGACAGGGTAGCGAGGGA

AACTGTTGATGCCCTCAAAAACACTCCCGTGTCTGATCTCATGAAAGGACGGCAGACTTGGGACTCACTC

AAGTACCGCAGTGTTCCAGCTCCAGAGGCTGTCCTTTCGTACATGGACTTCCTTGCTACCATTGGGGAGA

CTCCAGACACCATGTGGGAGGCATGGCAAAGCCTCGTCAGGTCGTTTGACAAAAAGAGGTTCGTAGCCCC

ATATCAGGAGGAGAGTGTTGTGAAGACCAAAACCAGGGAGAGGCTGAACTGGAAGAAGATGACAGACAAG

GACTCCTGCAAGTTTGTTTCACACCTCAAAAACAAAACATGGGAGGGTGACGCTGCTTTCGTTAAGATGG

TGGAGATCAACACTGCTTACGGCAGCTCCCTGAAGACAGGAGAGAGGGGGAAGGGGGATCATGAAAGACG

GTCCATAGCGTCGGGGTCGGCTCAGATGAGGATTGCCATGTACGGTCCTGAGACATTTCATCTCAAGCTA

TGCAAGGCTGTGCCCAATGCCACCATTTCAGATGGTGGTGAGAGGAAAAAGCACCAGATAGCTTCGGAGA

TGGCATCGACTCACTCTGGTATTCTGAGTGGGGACTTGGGCTTCCACACGACCGTAATTGGGAGTTTGGA

TGACACAAAGTGGAATGAGTGCATGCCCCATGAAGCGATGGCTTATGTGATGGACGTGTGGTTCCTCAAC

GATCTCGTGAGGGACGCTAGAGGGATTCAGCGCGCTACTGCTGAAGTGAAGGAGCTGGCAATGATGTGTG

AGGTTTCTTACATGATGACCTGCATGAAGAGGATATGGGTTGGATCCGAGATCAGGTCCCCGTTGGGAGA

TAGGAGGGTCGGACGTGTCCCGATGTGGGTCCACGAAAAGTACAACGCTGAGTGGAGGGAAGTGATGCGC

AAGGCGGCAGGTATCAGGCGTATTTTCTCTCTCACACAAGGAGATCCTGACCCTCACAACGTGTACCCGG

ATTTTCCCCTTATCCCTGGAGGGATTGTACCCACCCCTGCAAATAGAGCAGACTTGCAGCTTGCAATGAA

CCGTCATTACTTACCGGCCCCATGTGGGATGCTCATGGGAATCAGCAACGCCGGCTCAACCACCATGGGG

ATAATTCATGTTCCCCGGGCCACCATTCCCAGCGTGGGGATGGCATACCTGAGAAGCAGCGATGACAGCA

TGATCAAGATAGAGGCACCGAGCCAGCAGGCACTCGATGCTCAGTGGGCCCTAGTCTACAACCGCCTCCG

AAACAGAGGAATCAACATGTCTCTGACAAAGAGCTACTTCACTACAGAGACAGGCGAATACACATCTTGG

TACATCCAAGCCCAGTTCGCGGCAAACTTCGGACTGGAGACTAGTGTTGTGAGGCCTCAGGGGAAGAACC

CACAGGATGACTTTAACTCAATCGCTAAGAGTACGGCCACGTCCCTGAGAGAGCAGACCCTCAACGCAAT

GGGTGCCACCGCCAAGATTAGAGTCGGGGTCGGGAACGTCCGCAGGCTGTACAAGACCAAGCCATCATCC

GGGAAGTTCGGTGTTCACCCCAAACACCGGCTTCTGGCGTCCGGCGGAAGCAACCCTTGGGACTTCTCAA

ACTGCAACCTGGAGGAGAGCACTATCAAGGAAGTGGGTGCTAGCAGGAGGGCACGGTCTTACCTGGCTGC

AGTGAGGCACCCCCAATCCCCTTTCTCGGTGCCACCATCTGAGGGGTACGTCTTCGACAAGGACTCAGGG

ACCATGGGTCTGTCAGTGGTGGATGAGCCTAGGAATGTGTACTGTACTAGTAGAAAGTCTAACAGAACCA

TGAGGAAGAAGGAGCAACTCGGGCGGCTAGAAAGTGAAAGAGCATGTGCTGGTTTGGTGAGGTCTGCGAA

GGCGATTGACCCGGCTCTCAACTTCTACGTTCCGGGGGAGAGGGTCTCTGTGTTGGATTACCTGATTGCT

CAGGTTGTGGTTGAGTCCCGTGGCGCTGTCACTTTCCGTGAGGTGGCTGAAAGCCTCCAGAGGGGTGGTG

ATGAAGCTGGTCTGGAGGATGACGTGATATAACCCAACAGGAGAATCATTGCATCGGACCATTTGTGTGG

AGGAGCAAACTGGTGTGTGGATCGTGAAAAGAGAG

>GAYJ02032054.1 TSA: Atelura formicaria C259857_a_55_0_l_1569 transcribed RNA sequence // Orthomyxoviridae Envelope glycoprotein= hemagglutinin

CTCTCTTTTCAACCACCTACAAGGAGGCCGGTCACCCTCTACACAATGCTATGAAAGACATACCAAACGC

AATCCACCCAATCATTCCACTAATTCTTTGAAAATACTGCCACCCCCAAAAGTCCCTGAACCCGTCACTG

ATTATTGAGCCCAAAACACCACCACCATGAGTTACATCCCTGTTTGCGAACGCCACAGCCTTGAGGACCC

CATATTGCCGGTCCGCCAGCCAAGTCCACCCTTGCCAGGTTGAGGATGCGCCTTGGGGAGGGGGGATGTC

CAGCTCAGACAGTGAGAGGTTGTATTCGCTGAACAAACCTACGTCAGCCACCGCACTCAGATGGTAGCTA

GTGCAGGCCTCCGACTCTTCCTTTGCCCTAAACCTCCCCTGGTCATATATGAAAGTCCCAGCACAGTTGG

ATGTGGAGGCCATTACAGGCATGTAGCATGGACACTGGAAAAACACTTCTTTGTTGAACCATTTCGTCCT

CCCATGGTTCCCAAGAATGTTCCCAATAAGTTCGTCGTCTACCCTCCCCACACTTAGAACTAGGCTCCGA

AGCATTCGTTGCATCTCATGAAGAGATTGTAGAACCTTGATGTGATTGTAGATGCTCTCACTGTTCAGGT

ACAGTACGGAGTTGATGACCGGTTTCAAATCATCCACGGAGCTGGCCGCTCCGAGATCAGCTGCTGTTGA

TTTGATCAGGTGGACGTCTCGCTTCTCTCGCCGATGCACGTATATTGGGTCTGTGACCTCCAACCCGACG

TTCCTCATCCTCCCTCTCCCGGTCGTGAAATTTACCTGAATGATCGTTTTAGACAGGTGGCCAAGGGACT

CGGGCAGCTGGCACAGCGCCGATTCAGCATCTCCACTGGCGAAGCACTTGACCTGAGCGGTGTACGATCG

CATGGGCAGAAGGCCTTCCTTGAAAACGTAGATTATTGAGTTCTCGTCCAGCATGAACCCATCCTTGCTG

AATATCTGATCACTGTCGAGGAGTTCACCTCTTTTCCCTAGGATGGTTCTGTCCCAGGACTGTGTCTCGT

CCTTCCATCTAAGATAGACGGGATAGGGTGCAACTGTAAACCCACACATGTAGTCAGCGTTGTAAGTGTG

GTATGGGAACGACTGCCACTTGTTCCCGTAGCAGGTGAAGTCCTGAGAGTCGACCCAAATAGAATACTGC

TTTCTTTCCTTGTTCCATGACGTCGCTGTACTACCCCACACGAATTCACTACATGCCCTCTTCCCCACTG

CACACTTTTTCTTTGCTATCCATTTGTGCGCTTCCTCTAGGGAGGGAGGATCACCCCAATGATCTTGTGC

GCAACCAGTGTTCCCATCCGCAGGTCCCCCATCATAACAATAAGCGGAGTAACCCGCCCGCATCCCCAGG

AAGCCGTTTATCTCCTCCTGGGCCAGGGTTATCCTCGCCTGGTGTGATATCCATGAGACGTTTAGCACTG

GGACGGGCGGTAACTCTATCTTGAATGGCCCCGCGCAGTTCTTCTCAATGCAGGGGGAGCTGATGCACTG

ACCGGCTCCTGCAGCTAGCACCACAAGTA

>GAYJ02040263.1 TSA: Atelura formicaria s16139_L_41387_0_a_39_5_l_1862 transcribed RNA sequence// Orthomyxoviridae Nucleoprotein

AACACGGAGAAAGATCACACTCAAAAAATAGTGGCAGTCCAATCCCAGGAGAACGGTGCCGGACAGCACA

AACCGGCTTGGCTTGCTCAAGTCTCCATGTCGGTTGAGGTGGCCGCTGCCCCAGGAAGGGATGACTTGAT

CCCGCTCATCATCACGCTGGGGACACTTGAATCAGCCCTAGCAGTGGGGAGGGACTTCTCACCCGAGAAG

TAGAAAGCCACTCCAGATTTGGGTTTCTGCACGTCATCTGCCAGGTTCTCGGCTGACCACTTGGACTCGG

CAACATTGTAGGCCAAAGTAGAAGCGTTGTACCATGGGATATCCTGATACAAGGACAGGACGTTGCCTCC

TGTTTCTATTGCGTTGACGAACCCATCGACTTTCCCCTGGAGGTAGCTAATTACCTCAACAGCCGAGGTA

AAGTGAGAGCCGGTTTCCTCTCCCAGCTTCTTGACCATCTTGAGAAACTTCCCTGAAAACACCTGGGTCC

TTGCTCCCGCAAAAACACCGTGTGAAGGCGTCTCTAGAGTGTCAACGGTCTCCAGGGATAGCCCGAGTGC

CGAGGCCAGCTTGCTAGCTCTGTCTATCTTGGGAAGGGTCATGAAGATGCTGGCCTTCGTGCCCATGGAG

TCCGTGAACAAGTCACCCAAATCTGCCCTCGTGTGCCAAACCTCTCTTCCAGTCATGAACTGGAGCACCT

CCCTGCTCTCCAGATGGGACCCCCAAACTGCATGGAATATTATCTCTTCCATTATAGAGTTGCTCATCCC

ACCAAGCCGTGGCATGTGCCATTTGACTGAAGCACATTTGTTCCACAGCCACACTGCCCCCTTTCCCGAG

AAGTCCATCCTAGCAATGTCCTCCCCGAGGAGAGATACAGCCCTGGCCAGGAAAGCAAGGGGGAAATGGG

CCCTTCTTTGCTCCCTTGAGGTTGAAACCATGGCAATCCTGCACAACAGGGATGCGATCCTCTGCTTGTC

AGCTGGAACGGATTCTTCAAAAACCTTGACGATGGAGTCTATACCCCCCACGTGGCCGAAAGTCCTCTTT

ACAGCGGTTTCCCACTTGTTAAGGTACCGTTTGTCTGTGCCTGTCCCCAACATCAGGAGTTGAGTGCCTG

GACCCATCGACTGAGCCAGCGAGCTTCTCACCGAGGGAGACTGTCTGATGCCTGAAAGTAAAGGGAAGAA

CTCCCTAGCTATCCCCGCTGAAGTGACCTTGATCATGTCGCCCTTGCCATTGGGTGTCTCATCCATCCCC

AGCCTTATCTCCTTAATGCGGCCGGAGAACCCCTGAAAAAGAGCAAGCAGGGGGTTCATGGTTCCAATCC

AATGGTCCTTGGATCCCTTAGATGGGGTTAGGGGGAGTCCTCGCATGGGGAGCACCGTCTTCCACAGCGA

GATTATCGCACTGTTTTCCATCTCGACAGAACTTCTGTTCCAGGTGCCATCCTCTCCTTTCTTCCAGAAG

TGGAAGGTCCCCTTGTTGTTCTCGACCTTCGTTCTAAGGCTGGTTGGTGTGTCAGGGTTGTTCAGCGACT

GTCGCAGAGCCTGGAATGCGCTGTACATCACCGAACAAACATTTGAGCTCAGCAGGAGTTGTCTGTCCAG

GGAACTGGCTATGGCGCCCGCTCTTCCTCCTCCTATCTCATCAAGGATTGACACGTACACGGAGTTTATC

GCCAGGAGGCAGTTCTTCTTCTGGGACGGATCAACTGTAATATCCACTCGCCTCATTTTCGTCACCTCCT

CCCCGCTGTAGGCGTCAAGCTTCCTCTTCCTAGTTGTCTCTGCCTGTTCTGCCATATTGAATGGAGTGTC

TGATCACAAATTATCCGTTATGGAGCGAGAAGGTTGTTGCTG

>GAYJ02033043.1 TSA: Atelura formicaria C264075_a_56_0_l_2428 transcribed RNA sequence// Orthomyxoviridae PA protein

CCTCTCTTTTCATCCACAAGTCAATTGCGTACCCAACAAAGAATTTGCAAAGCTGCTATCTCATTCAGGG

GTAAGCAGTGCCATGGCCTGACGCAATGACTCATACAGCCTGACTGCCAACAGAGAGTCAATGACCTGCT

CCCTGGCTTTGGAACAAAACTCCTCTATGTTGCAATGGAAAACTCTCCTACCATTTGCCCACTCCCTCAA

CCAAACCACCATCTTCCGCCGGTATGGGAAGAATCCTTCTTCCTGAGTTCGACCAATCAAGGTCATGAGC

ACGTTGTCCACAACCCGATCTGTGAAGTACCCAAAGGACCTTGCCATCGTTGAGGCCGCCACTGCTCCAA

AGTCCTCCGCCTCCACTGGGACTGTGGAGCCAGTCAACAAATCTTCTACTAAAGCTCCAAACAGGTTGGA

AAGTGGAACTAGCATGCCGTACGCAAACGAGAAATAAGCCAGATCCCCTTTTGCAATGGAGTTCCTCCTG

GCCACTATGAGTTTCCCTCCATCCGTCTCACCCACTATTACCCAGTTGCTTGGGAGACGTTGGTTGATCA

TCATGCTACTAAGACTTGTTACTTGGACAACAAGTATGGGTATCCTGTCAGACTCATTCACTGTGTGGTC

AGGCCCACGCAACAAAATTCCATACACTTCTCTGTTGTCTCCCCCGGTGGTCGTCTTACTATAAATGGGC

CAGCACATCAACCTCTCCCTCCTGGCATGCCCTGACTCCATCCCCAGGGCCAGCCGGGTCGATATTCCCA

CTAGGCATGCAATGTCCCTCATCGCCTTGACTTTGAGCACTTCAGAGAAGAACTTGTCAGTGGCACTCTC

GCACTCTTTCGCCAGTGGGTGAACTGGATCACATTCCGACGTTTCCAGCTGGCTGTAAACTGGGTATTCT

TCGATCTTCGAGTACTCCTTGAAAAGGTCGTCAAACCACTCAGGGTATGACCTCCTGACTCTTGGTTGCA

TTTCTGGCTGGACTGCATCAATGTCAACTAGCCTGTCCCTCCTCCCCACCCCGAAAACAGGAAATACTCC

AGGGTAGTTCTTGAACTGAGCCACAGGGCCCAATATGGTGGTCTCCGCACCAAGGAGGATTGGGAGCAAA

GACATTATGGCCAGGGCCTCCTCACTGTCTCCATGTATTGTGCACCCTGCTAGCCATTTCCTTGTCATCA

AATGGTCCTCCGCATCATCAGTGTTGACAAACTCGGCCCAAGGTGTGGGAAGGAACTTGCCGTTCCAAGT

GGCCCCGTCTCCCTCGCACTCTGGAAGGAGAGACAGAACCTCTTCGGGAGTTACCTGCCTGAAATTCGGG

ACAGGGTATTGTGACTCATTCCAATCACCCTCTTCCTTGATCCTACCCATCCATTGTGAAGTCAGTGACC

ATGCAGTGGTCCTTTGAAGGTCAGCGGCCCAATCTTCGTCGTAAGCGCTCTCATCCTGTTCCGAGGGCAT

GGCGTTTTGTATCCACAGGATCCGTCTTCTGTTGAGGAAATTGAGTGCCTTTGCAAAACCTGGAACAGTG

GAGTCAGCGTTAATCACTTCGGTTTTGGTGCCGTCATCACTGAAAGCGAGAAGTCCGAACCTCCTGGGAG

CAGCACTTCTTGCCACTCTCACATTGAACCTGCTGACCTCGCGAGCTATGTTCTTGACTACCTTGACTTC

AATGAACACCTCCTTCACTCTGTCAACCACGTCCCACAGATCAGGTAGATTCGGCATGGCCCATTTGTTG

CAGTAAAAGTTCTGAATGTACACTGCTGACAAATTGCCTTCAAGGGGAACAAACCTGTCATGAGACCTGT

CCCCCAACAAGATGTCCTCTTCATCTCTCCCTCCACCCATCAGGTCATCACCTTCCCCCTCATCCTCTTC

CATGGAATCGTCGGCATCGTCCTCACTGAGCTCTAGCACTCCAGCCCCTGATGGACCGGGTTGATCCAGG

TCCTCGATTACCAAGGCAGTGCCGGGCCTTGATCTCTTCGGGACGCCGTGAAGAGATGCGTCTAGGTAAT

CCACTGGATCGATCCCTCTTCTCTCCATGTCAGAGAGAGTCAGGGCGACGGCTATTTCCTCCCCATAGAT

TGCCCGTAACCTGTGCTCAGTCTGGACCAAATCTTCAGCTGGGGTCATCTTTCTCTTTCTTGAGGAGAGA

TCCAGCTTCACAGCTGCAGCTGTGCTGTCGACAACCTCCTGGAGTGTCGGCTCAGAGAGCGTGCTGTTGC

AGTACAGGAGGGTAACTGTCTCATGTCGCAAGAACTTCTCCTTCCATCGCGGGGTCTCATTCCGATGCTT

TGAAGTTCTCTCTACCAACCTCACCATATCTCTGGAGTAACACCCAGATGTCTGGATGAAGTCCTCATAT

GGGTTTGTGGGCCGTTCCATCATTACCTGGTGTGCAAAAAGAGAAGGC

>GAYJ02033073.1 TSA: Atelura formicaria C264205_a_26_0_l_2491 transcribed RNA sequence // Orthomyxoviridae PB2

CTCTCTTTTCCACCAAACACCACGGGAGAATCATACCCAACCTGCCAGGAAGTCTCACCCGACTTCAAAT

CAGGTTGAAAAGTGCGACAACTGTCTCGCTATTGATGCAGCACTGGCAGGTCCGATGCTGCTGTCTCTCG

TCAGCTTGAACATCCGCCCACCCTCACTCATGTAGGAGCTCTCACCCTCCTTCAAGCTCCTCTTGTTCTT

CCAAGACCTAAACTCGGAGAGTCTGAAGACCCTTGTCCCTTCGGGCACCTCGTCTACTTGTGTGATGGCA

AAATCGTTGAACGCATTTGTCCCCTTCACCAGTGTGCCTAAGTGAAGCTTTTGGATTGATCTCTCCAAGT

CCTGCTGCATCTTCAGTTCTCCATTAATTGGGTCTCTCCACAAGATTGTTCCCATTCTCAAGTTAAAGTC

AACGTCCATCCCTCCATACTCGAAGATGGTCAATGGCTCGGCCTTCTTCCTGGGTGGTCCCCCTACGAAC

ATAAATCCCAGTATCCTTTGACCAGACATGAGAGTTTCAGTGACTGGATCGGCAAGGACCGCCAGGGCTC

CAGCATGAACTCGATGTAAGGCCTGAGTCGTCACATGGGGCTTTAGGTACCTGAAATGAGGAGTTGACAT

CTTTGCTTTGTTCTTGAGCCCCCGGTAAGTGGCCTCATAAGTGTCTAAGGCCTTGGACACCCAAGTCCAA

TGGAAGCCGAAACACCCCATGGGGTCCTTGTGGGCATCCACCACCGGGAAAGTGGAAGTCACACTAAGTG

AGATTGGGTCATTGGAGTCACCAGGGTCAGGCCTGTCGCCGGCAGCCACCCCCCTCCATGACAGTGACCC

ATCCGCCGTGTACTGCATCGACTCACTAGCCAGATCAGGTTCCTCTATATGAGAAAGGACCGTAACGACA

CCAAAGTCCACAGGTGTCCCCCCTCCAAAAGCGCTCCCCAGTGACAATAGCCTCCTCTTCCCCGGTGTTG

CACTCAGCAACGTGTCAGTTAGGAGCCTCTTCAACAGAGGGTAATTGAGCTCAGAAGTCCTGAGAAACTT

CCATGGCTCCCTCCTGACGTTTTGCTTTATGGTGTTCCTCCACCCACCACCAGATGATGTTGTCTCTTGA

AACCCCCATCTGATGTAAACTGCGACATCAGCTATCAGTTCAATGAAATCGCCTGGAGCGCAGGGCTCAG

TCACTATTGAAATTATCTCAGACCCATCATGAATAAATGTTGACTTAAACTCATTTCCCATGACGAACAC

TGACTCCCTCCCATCATAAACAGTGGTGACAGCGCCCCCCTTGTTCCTGATCCTCCTGCCCGGAGCCAGG

CCCTTCCCTCCATACGGAGTGAACTTTCGGGTTCCCTCACAGCTAAATAGCGACGTGGGGATCTCGAAAA

TCGACTTGAACAGGCGGACCCAATAGTCTTCAGTTGTGTCAGGCCTGATTAGCACCTCCTTTATGGGCAC

CCCGTCCAGGCTAGTTTGGGATGCAAAGTCCCTCCAGCTGTGGGGGTCCACCCAGTCAAGCATGAGGTTC

CCAACAGCTGCAAGTAATCCTCTCAACCTTGATCGACTTTCTGGGACTTCCAGGATGTCGTAGTTGGTTA

AGTGCCAGTTTTCACCGCTCAAAGCTTGCCTGATTGGGCCTAACTCGGTTGGGAATTGAAGGGGGACTGG

GATCAACCGAGTTTGAGGCTGCAGCATTCTCACGATAACCTCTGATTGACTGACAATGCTGAGGCCGGGA

TAAGACGTTACACTGATCATGTCTCTCACCGTCTTCAATGTGGGATCATCTGCTGGGAGCCGATGGATTA

CCCTGTCAGGAAGGACACACTGCAGCAGGGCCTGACTAACTCTTGATGGATCAACCTTCTTGTCAGTTGC

TTTGGTAGGTACTGTTTTCCGAGTAGTGGACACCTGCTTGAACACGCACCTTGTTGCCCGCCAGTCCAGC

TTGTTGTACATGTCCCTTAGCTGCCGAGCCGGGCCGTACATGACGGAAACTGCCTCTTCTGCCTCCACTA

GGGTTGCGTCGTCTTCAAATTCGACCATTCGTTTGGTTATCCAGTCGTCAATGGCAGACATGGAGCAAAT

TCCGAATCCGTACAAGTGGTAGTCATCGCCTCTCTTTATAGGGTCATGAGGGTCAACATCCAACCCGAAT

GCGGCCTTCCTCTTGACCATTATGGTGTCTTTCATCTTCGGCAGGTGCTTCTTTGGTATGTCTTTCCACA

TTCTCTTGTTGTACACGATTGGATGTGGGTCCTGCATCCTCACGAGCTGGTGAAGCAATGGATTCTGGTC

TCTAGTCGTTCTGGAAAGACGCTCCAGAGTCTTGAGGTCACAGACTCTATACTTTGAGGCGAGGGCGACC

AGCTCCGGAGACAGTGATTTGAGCTCCTCCCATTTTTGCTTCCATCCCACCAGCTCCTGTTCGGATTGCT

TTATCTCACTCATCGGAGTGATGGAAAAGAGAAGGTTTTTG

>GAUE02021853.1 TSA: Anurida maritima s8018_L_22455_0_a_66_9_l_9648 transcribed RNA sequence/picornavirus

TTTTTTTTTTTTTTTTTTTTTTTTTTTTGAACGTTAAATTGAAAGAGCCAATTAAACCTAATTTTTCTGG

ATTGAATCTATATTGGGTCTACAGAACTAGGCACCAGGTCAGGTGACTAGAACTAGTGTACTCAATAAGG

GAATACGTTTTAAAATCCCCTATCTAACTACTTATGCCTCACTCTCTGTGTGAGCAGGCATACTAAATCC

TACGGATAGTGCGCGTGATGGTACTGACATAGACTTCACCTGCATCTGTTGGTCGTGTGCTTGTTTAGCC

AACGACGCGTTGTTGAGATACTGGTTAGCCTGATACTCCATATCATGCTGTTGCCCTCTGCGCTGCATAT

CGGATTGCTGACGGAATTGCTTCCCCAACACATCCATTTGATGCGCCTGTTGATCTTCGCGGTTAAGAAT

GCCGAAGGCTTGCATATCTTCCCTGTTCTTGTCCATCCACTTGAACTGATTCTTCTGCATCCCCTTCTGA

TGTTGCATCTGGAAGAAGCTGCCTAAGCTAGAGCCGAGACCTTGAATTGCGCCAGATGCTGCCATTGCGG

CTGCAGCTGCGTTGAACTTAAGGTTCGCCACGTCATACACTTTCTGGTTGTCATCCGGCCAAGCCACAGT

TTTGACGACTTTGGACCCAAATCTGGGTAGCCATCCGGTTAAAGTGGTCGGTGCTGGAGCTGTTGTTTCC

TGCACGACCCTAAATTCTGTGACCTTTAAGGAATCTACATAGACTGGTGCTGTGTAGTGGTTGCCGATTG

TTGTTGGTGAATAAATTTTGAACATGCGTTCTGTAGGATTGTAGCGAACTGTCGCTAAGGTTCCTACTAT

CTCTTCGAGTTTAAACTCAAGCTCTTGCCCAAGCTGGACATTGCGATCTACTGCTTCTTGGTCAAACACG

CGAAGGAGCTGTTGAATGGATGACATTGTTTCAACTCCTGTTGCAGTCTGGCCTACGAACGTTGGTTCCG

TGAGCGTGAATTTAAATGTTTTGAACCCTGATGGTAAAAGGTTCGGTTTGACGTACGTTGTTTGAAGGTC

AGAGCCGGGATAAGGATACGCCTTTCCTTTCTTCCAAGCTACCTTTGAGATCCAGTTGTAGGGATCTTCT

GTTAGTGCCTCTATTTCGAAGACACACATCGTTCCAAATGAGAAGACTTGCTTCACGAACTTGGTCACAA

CTATCTTTACGGTGGTGGGATCTGTTGCAAAGAAATTGCTCATCTCCGTCACATCGTAGACAGTTGGATC

AACCACGATGGAAAGTGGTGTGTGGTCCGCAAGCATCGAATTCTCCTGGTAATATTCCGTTGGGTTGGTG

TTGAACGGAAATGGTGGCAATGCTCTCTTGAGCAGCTGTTCCTCTCCTTTGATGATCATATCGGAACTCC

AGCCGTACTGAATATTCCTCTTGTCTACTCCAGGGTCTGGATGACACTGGACTACGTCATACTGCGCCAC

AGACGTGTACATCTCTTTCATGCTATCGTACAAAATTGCTTCTGGACGCGCAATCACAGAGTGTGTGTAA

GCGACGACAGATGTCTTCTCTCCCCTCACTCTTCCGTCGGTTGCAGCATACATGATCGGGTTGACCGCTG

CTGTTTCAGGGATCAGATCTACAATCTTCTTTCCCTCGATGGTGTCTACTTTTGGCATTATCATCCTCTC

CAGAAGTGCTGGTGTTGGATACATCGTTTGAGGTTCTACAACGTAAAACCCTTGGGCTGCATCTTCAGCT

GGTGGTAAAAGTCTGGATCGGATATTGAATACCACTGCCGCGTCTCCTTCAGGGAACTGATTGACAATAG

GTACAAAGGACGCTATCACCAATCCTGGTCTGTCCGTCAATCTCACATCTTGCACATCAGGAAAAGCACT

GTTGAGTGGATTTCCATTGCTCTTGTTGAACTCATCCCATACACTTCTGTAGAAGGTCTGAAGACGAGCA

TCACCAAGTGTGAAGAGATGTGAACCTCCTCCTGTCACGTCATATTCCTGCCATGCGATCTTATAAAGCT

CGTTAATGTCGATAGATGCCGCTGTTGGTGGTGGGCAATTGCGAACGATTCCTGCCAAGATTTTGCCTCT

TAGCATTGGCACTGCTGTCATGTCCAATTCATACTGGATGGCTCCAGTGTACCTGAGATGATCTTTCGCC

CATCTTTGTGCAAAGGGGTTCATCTCTTGTACCCCTACTGCGTATGGAAGGACCTTTAAAATCTTTCCAC

GTGATTCTCCACCATTTACGGTGAAGGAACCGCCGTCGAAAAAGTCAAAGGCATAGTCGATCGTGGATCG

GATGTTTAAATTCCTATATAATAGATTTTCTACAGTTTGACCTGCAGTTAATCCGAGATTCTGTGCCGAC

ATGTCTGATGATGCGACTACCTGCTGCGAATCGGGCTTGTTGGGAGGCCCTGCAGACGAGGCAGTCAAAT

TGGTCGTTCCAAAGGGTGTTCCGGTCGCTGCTCCTGTGGCATTTCCTGCTGCTGCAGGTGCCGCTCCCAT

GACTTGTTGGACCGGTTGTTCGGGTCCATTGTTGAAGGACAACTCACTAAGTGAGGATATAAACGCGTCT

TCATCGATTAAGTTACGCAAGCGCACCGTAGCCTCATACACGTCTGATCCTGCAGCCTTAAACCTTTTGA

TACCACGCGCGGCTTTTGCAATGATGCGGCCTGGCTTGGTTTCGGAGATTTGTGGTGCCTCCATATAAGC

AGAGAGTTCCTGTTCTTGTCGGGACCCTCTAAATCGTGACTCTTCACAAGTATCGTTTCCTTTACTTGTG

CCGGGTATGTACCGTGGGAATTTCTCTTGGATGATTTCATGCAAGACTTTGTAGCCAGCTTGTTCAGCTT

CCTTCTTTGTCTTGCAAGACGACGTTGTAATGTCGTAATGCTCTCCAATACTCCCTTGTAGGTTGACACT

CCAATAGTTGGTGTTGTTCTCGAAGCGTCGGTTGAAAGTTACCGTGGGTTGAACTTCCATGGGATGTCTG

TCGAAGAACTCTTTGACGAGTCCGATGTAGTTGACTTTTGGTTGTTCGGCCATTGTAATGTTGGTTGTTT

GTTGTTGTTGTTGTTCTTTTGAATTAAAACGCAAATCACTATTAATACTCACTTTTCCAGTCTCATTCGA

AACTGGAATATCTAGTAAGCTAGGAGCTGGTAAGCCTGTTGGCCACTTGCCTCCTTTCATGTACGAGAGA

AACTGTTTTCTGAAAACTTTGTATTCTGGGAACACAAGTGTTTTCTCTAACGTATCCCTGCACAGTCCTG

GCATCTTCTTGATCTGCACTCTAATGTCATGTACGATGTTTTCGTACTCTGTCTGTCCTGCTATCAAATT

TTCTGTCAGAAGGGATTGCATTTGACATGCCACTGCTGAAGGTTCAAATGATTGAAACCAAAACAGTGTA

CGTGTTCGAGATTCTTTTTTCAAAATAGGCCACACCACTCCGTCTTCCTTACAAAGCTCCAAGCTTCGTG

ATAGAAAGATGTTTGTAGTGAGTCCATCTTGTTTGTCTGCATGGGTCATTTCCATTCCCATTTCCCTAGC

ATACTGAAAGAATTCTTCAGCTTCAAACTTCAATGTAGTTTTAAGCTTTAGGTCATCTCCCAGACAAAAT

GCTGTGAAAAGGTCATGTAATTCGAAGATGGTTGGGAACGAGTCATATTTCTTTGCGTGCACTCTAATCA

AACAGTACACGAGTGTCCTTAAGTTTGCATAGTCGTCTAAAATAGTGGTCAAAAATGCTCCTGACAAAAC

ACCTCTTGGTGATGAAAAGAGCGTTCCTTCAGCTACCGTGATGGCATGTGGTGCTACTCTACTTTCCTCT

CTGTACATCTTTGCTACTCCTTTGGGATCTTCATGCATGAGACATATCGTTTTGGCAAAGCCTACACGAT

TAGCCTTTCTGACACGTTTGTCAAATCTGGAATAGTCGAAGTCGTACTCCGATCCTAATACAGCGTCTAG

GCGTCTCCTTAAGTCTGTGAACTCTATGAAGGAATTTCCTCCAATCTGAGACGCCGCATTTCCTCGGTTC

TTGACGCATGATGCGAACAAATACCCGTACATGACACGTTCTACTAGCACATCTGAGATGCACACGTTAA

TGAAACACCTCGTCTTTCCTATAGCTACTTTCTCTTTTGGTAAGAGTTCAGTCTTCTTTGAAGCTCTAGC

TGTTGGTATGATGGGATTACCTGTGCGTAAACCCTCTAGTTTATCAAAGACCATTCTCTTGAGATCTAAT

CCAGCTTCTGTGTCTGCGAAGTCATAATGTGGTTTCCCTCCTGTAGTGACGATGTTCTTGAAAAGTTCGC

CCTTAGTCTTGATATTCCACTTCGTTTCGAAGTAGAATCCAGCTGACGCGTCCATTTGCATTTGTCCTAA

TAATCCGTGTAGAGGATCTCCACGCTTCACTCCGTTGATGGCTTCGTCTATAGACAGCAAGCGCATTGCA

CCATAATGCTTCTTGTAATAATCAACAAGGAAGAGCTCGGCGAGATTTTCTGCATTCGGCTCAGGTGTTT

CCTTGAAGTCGTCACAATAGTGCATGATTTGTGACCAGAGAATGTTGTGTTTCCCTGTCTTTAGATTGAG

GTACAATCGCGATTTGTCTGTTACTTGATCCAAGGTCTGTGCTGCTGGCATAGAATTGTTCGTATTTCTA

CAGCCCTTAGAGTCCAACATTTGAAATGCTGGTCGTTTGGGTGGGTATTCCGCAGCTTTCTTATGGAACC

CAAAGACTTGCAGTCCTGGTCCATAGAATCCATGATCTTGTGCCGTCTTAATTAAGCTATCTACGTATGG

GTCTACTCTGCATTTTTCACCATACACTTCTATTTCTGGGTAACTCATTGAGTTATACTTGATGCCTTCT

TTGTTAAATGAGGTGATGTTCTCCCTCGTTGTAATGATGTAGGCTGCAAAGTGCTCAGACAATCCATTGT

GGAGTCCAGCAATTTTGACACCAGTTGGTGTGCGAACTAATAAAGGTGTTCCACAATCTCCTTGTTTGAG

GAAATCATGGACTACAGCTATACCAGTAGGGACTACCAGCATAACATTCTCAGCTCGGTAATTGTCTGAT

GCCAGTGCTTTTGTGAGGACGTCAATCACCGCTGGTGAATACGATAACACATCCTGATCATACCTTACTA

TTTGACCATAGTCAAAAAAGGCGACATCTTCTTGAAGTGCAAAATGTTTGTCGATGTTGGGGAATCTATA

TTTTGGTGGTAATTGTGACACACAGAAGTCTCTTTCTGCATTTGAGAAAATAACCTTACAGGCCACTTTC

ACAGCAGAGCCTTGCCAATCAAACACAGCATTAATAATGCCGTCATCTGCGACATGGGCTACTGTGTAAA

TTTTGTCTCCTGATGCCACTCCATAACATTTGGTGGAATTGTCTATTACAACAGCGTTTTTGTGAACTAC

TGCTGCAATGATTTCATAGTCTCTTTTGTTAGAGCACACTGGTGGAACATTGCTAATAGCATTTGGTTTG

ATGCCAAGTATTCTAAAGGCCTTACGTACTGTTGGATTGTTCTCGTTGGCTTGGTAATACCGCGTCAATG

CTGTTGGTGTCTTTCGCAATTGGCTAACCATAAGCTTGGCTGTAGCCAAAGCTATATCGTCCTTACCATT

GTAAGCCCATCCTCCGTCCATGTCACCTTCCCAGTCATCTTGATCAGACCATTTAGGTCCTTTGATGTCG

TATTCTTTGTCTAACTTCTTTGCGTAATTGTAACGCCTAGTGGCTTCTCTTTCCTGTTGTTCTGCTTCGT

CGTCACGGTCTCTTTCTCGATCCATGTCTTCTTTCATTCGTCTGAGTTCATGTAATGAGTACTCACCTTT

CCATGATAGCGAACCATCAGGTTTAAGTCCCATATTTCCAGGTAGTTTTCCATGTTGTCTTTCCAATTCG

CGAAGCATCTTCTTGTATGCCTCGTATTGTGCTGATGTCATTCCTGCGTTGGTTTCCATAGTAGCTATAT

CTTGTGCTAGCTTCTTGGTAATATATTCGTGAGTTTTACTGCCTATTGAAGCTTGCGTATTGACTTTAAC

TATTTTTGGAGTTTCAGGTTGTGCCTTTCTTCTTCCACATATCATTGAAATTGTGGCAACTCCTGCTACT

ACTGCACCTAAGCCTGCAAGTACTGCTCCCGCTATGATGAGACCTTTGTGATCTCTCCATGTTTGGTAGA

GCCTTACTTTCAGAGTTGGGAATTCTAGCTCTTGCCGAGATGGTTGTGATAGAGCCTTTAGTTGTGGATG

CGTCAAAGCTGTTATTTGAATTGCTTCCACCATCATGACTCTTTTACATGCTGGAATATCCCTTGTGTCG

TGACTTGCGCAGAATCTGGCAATTTGTAAATCGTTGAAAGTATGAGATTCATGTCCGTTGGTGACAATGT

AAGTCTTAGTGGTGTCGTTATAAGCACATTCCCATTCACTGTTGGGAGTGGATACATACAAAACCCCTCT

GTAATACTGGTAGAAAAAGTCTGATCCTGTTGCTGAGAACGAAATATCAGGACTAATTCTCCTGAGCGCA

AGGATATAGCGCTTAAGTACTGGTTCCGGGTCTTCTGTGATATTGGGGCTAACGATCCAATCACTAACTT

GCAATTTTCCTTTGATCGACTCTAGAAATCTATCTGAGAATCGAATCTTGTTTTTCGTGACCAAGTTGAT

TGCTGATGTCTTGTTGGTAATGGCTTTCTTGAGGTCTTCTTTGTCGTCCATGAGTAGAGTTACGTCATAC

TCCACATTGTAGGGACATCTTACATGCCTTACTACTATGCGCTTCTGAGTTTCCAGGTACTTCTGATATT

TGAGATCAAGAAAATCGAAGAGTGTGTCGTTCGTGAATGTTGCACCTTCAACTTTCAATTCAGCTGTGTA

TCCTACATCAACTATGGTCCCATGATCTCCAAGTGTTTCATATTCCATGCCGTTCCATACCATACCCGAT

GCACCTGATCTTCTGGTGTAAGCCTGATTAGAACCTTGAGCATTGATATGCCAACAAGGTCCGTATGGTT

CTAAGGCTCCTACAGCATCTGCTGCAGTCTTAGACATCTTTGACGCAACTGAGTAAGTAGCCGTATGTAC

GACAGTTTTGCATAAGTCTGTTTTCCTGCGATATTTTTGCAGTACTTCATTTGTGGCAATGATGACTATA

GATCCTTGTGCGATGTTGTTCACTAAATCTATGTAAGTCTTATACTCTCCTGTGGTGAAGAAGTCATCAA

AGACATAGATGGCCGGGTGAATATAATGTGGGAGTAATGCTGGTCCTATAAGGGGATTATTTGAGAACAC

TTTCAATTCAGGATAAAGTGTAACAAATTGTCTAGCCAGCGTCTCTGCTAGTTCTGTTTTTCCAGTGCCA

GTTTTGCCTTGAAGTCTTACTACATAAAACTCTTTAGCAGCACTAGCAAAAGCCAGTGGTTGTGGTGGTG

CCGGATTGTGTATCCTTACCCACGCATTTCCTAGGTGGTCAGGATTGGCAACTTTTCTGGCTAAATAGGC

TTGCTTGTGTTGTTCCAAGCCTTCTGTCAATGCTGGTGTGGGATTGTAATAGTTAATCATTCGATCAACA

TAATCCACTTCCTGATGTGCCATTCGGTGTGCAAGTCCTTCAATCAAAGTTGGGAGTGTGATGTTTTCTG

CTCCCAACAAGTGTCCTTGTTCATAAACTCTATATGAGAGTTGGAGATGACTAAAATCCGCACGTCTGTG

TGGATAAGCATCTACTGGCGCATTACGACCCATGATTTGTGGGTCCTCTACATCAATCCAAATAATCCGA

TCCCAAAGGGCATCTACACTCTGGGCTAGTTGTTGTGCCTGAAGCTGTAAATTATTAGGATCTTCTATAT

TGGATGTTAAGAAGATGGCGCCAAAGTCAATCCATTGTTTCTTTTCCGGTAATGCTGGTCCTGGTAAATT

GTATGGGTCGCATGAAACTAGTGCATTAAAAATTTGTAGCACTGGGTCATTTCCTTTGAATTGTAGAAAT

TCGTCCTTCATGGCAAACTTCTCTCCTGCATAGGTGTTGAAATGCTTTCCTCCTGGTACTGTGGCCAAAT

TGAAGCGTTTGTTTGGGTAACCAAGTTTTTCACTTAGTTGCTTCTGGATGTAATTGACCATAGTTGTCTT

GCCAACTCCTTTCCTTCCTGTCAACATAATAACTGTTGGAACTGGTCGTGTTGGTTTGGTTATTATTAGA

GCCAAATCTAAGATGCGTGCATTGATGTTAGACATCTGCATCACTAATTGTTGACTGAGTGCTCTTGTTC

CTGGGTCCACTTCTTTTTGTCGTTGAATACTCGCTAATGCGTTGGTAACGCGAGTGTTGAGTGTATTAAG

TGCATCGATCTTCTCATAGTCACTAATAATATCTTTAATAGGCATTGTAAGCAACTGGTTCACTTCATCG

GCAAGCCTCTGAAGCAGAACCTTATTCAAGGTGGGGTCCGAAGTTGATTCTGTAATGGCTTTAGCTAACA

ATTCAACTGTTGAAGAAGCGTTGCTAGCCATGGTGGTAGTGCCATTCATTAGTCCTACGACTGATGCTGC

GTCTTTTACACTCGTACACGTTCCTGTGAGGAGTGCAATGACTGTTCCTGCTAATCCTGAAAACAAGATC

TTTACCCAAGACATATGAGATGAAATCCACTCAGAACAAGAAGTAAGAACCTCCTTGAATCCGTTGAATT

GCAATCCCTCTGGAGCTTGGTGTTCAGCTCTAATCTCTGCCAAATTCACATTTTCTGCTATCTGGGTAGC

CACATCTCTTATGCTCGCCATAGCTGCTGATGGATACAGCTTAATGTTGAGCTTCATTAACAAGACTATA

AGTTGTTGGCATATTCCTGTTAATGTCGCTATAATACCTGTCACAGGTATTGCTCTCTCATTCGTCGTTG

CAACCGAAACCACATGGAGCATCAAATTGATGACATGCACGGCTAAGGTCGTTCCAAAGTTTGTCCAGTC

TTCTGCTAGCTGGTAGGCTAACAGTGTGAGTCCATTACGAACGGTGTCTGCAAACGCATTCGCTAAATCC

TTGACATTCTGTAGCAGACTGTCTGATAATGTTGCCGGTTGATCTGGGGGTCCGTCAGTAGGGTTTTGGG

GTGTTTCAGGAGTTGGTTGTGGTTCTAGGTCAGGTGGTTTGTCCGAGTTGAATCTCAACTGAAGATTATC

AGCTGAAAAGTTCCTTCCGCCCCCAAGATTATGAAGGCTCCTTACACCCTTGAGATTACAAGAGTTATCG

TCGACGTGACTCCCAAGATTATGAGAGTTTTGTTGGCTCCCGAGATTACGAGAGTAGATGTTGGTTTGGC

TCCCGAGATTACGAGAGTAGATGGTGTTGACATTTCCGAAATTACGGAGATGTTGTTTGTGCGCGTCCAA

AATTATGGATGCGTAGTTTACAGGTTTCGAAATTACGATTCCTGAGTTGTTGTTGTTTTTGTTCGTTCGA

AAATTATCGAAGAACATAATTCCTCCATTGTATACAATGGGTGAACGTTTGGTACCAATTGGTACGCTGA

TCATAATAATTTCCATTTTGGGAACTACACACGAAATAAGAAAAATACAGGATAGTTA

>GAYJ02042604.1 TSA: Atelura formicaria s18246_L_58722_0_a_56_6_l_5717 transcribed RNA sequence/picornavirus

CATCGTGTGCATTCTGCTTCGTGTCTTGTATTTTCGTCTCAAGGTGTTCGTGAAATAGTTGGTAAGGTAG

AATTAGCCAATCATTGTGATCTTCCGCTTCAAGCTCCTGGTATTGTATTTTCTAAGAAAGCTTGGACTCT

TACTTCTTTTAATGCTCCGAGTTTCCAGACTAGGCCTGGAGATTGCGGGTCACCTTATGTGGCTCATAGT

AATAAGTTGGGTGCTTCTAGGATTTTTGGGATACATATGATGAAATTGTATGGTAATTCCGTAGCCACTC

TTGTAACACGAGAAATTATTGAACAGTTAATTCAGTGTCAATATCAAGTTGATGAAGAGTGTGAATCTGA

AGGTCTTGAATATTCTCCGCGCCTTTCCACTGCTGAAGTAGATTCATTTTTAGATCCATGGGTAGCAAGT

GTGATGGAAGAAGCTGAAGGAGATCAAAGTAAGCTGCCTGTCTTCAGAAATGAAGTTAGGAGAGAGAATA

TTGATGTGATTGGAAGAAGTAAACAGATTCGATCGGCAAGACCAAAGAGTGGTTCAAAGATTATGTATCC

TTGGGTAGAAAAATTGGAAAATAAAACTAATCTTGTAATTCCTCCTATGATAGCTAGTGAAGTTAAGGAT

CCATCAAATTTAATTAAATTAGATGATGGATCGCGTCAATTATTGTTGACACAGGTTTATAAATATAAAA

TAGATCCTTGTCTTGAGGAAACTAAGTTATTGTTAAAGGAAGCCAGAGAAGAACTTAAACCAATTCTTAT

TGACCTGTATGGCAAGCAGAAGCATCGTATTTTATCTAAGTCCGAAGCTATAAACGGGATTTACGAACCA

GGTAAGTCTTATTATGGTGGATTGGAGCGGATGGATATGACTACTTCTGTTGGTCCATACTTAAAAGCGC

GGCACGGCGTGTCAACTAAGAAAGAAGTGTTTCGTGTTGTGAAAACCACAAAAACAGGCTTGCATCTTTA

TGGTGTTGATACCACAAAGGATGCAGGGCAAACACTTCAAGCTAGATGGAATCTTGCTTATGGTTTAGCT

ACCACAGCACAACCTGAAGGTCGAAAACCTGCCAGGCTTGTCATGGTGACAGCTGATAATCCTAAGATGG

AACCGCGTCCAGCTGAGAAAGCAGCAATTGGAAATATTCGCTTGTTTAACAATATGGAAGTTAATTCTGT

ATTGTTGGAACGGCAGTTTTTCGGAACCATTGGTGCTGCAATGAAATGTGAACATCTTAATTCTTTTGCA

CAGATTGGAGTTAATCCTTATGATGACTTTCTTGTGCTTTTTAAAAGATTGACTAAAACTGGATATGTTG

GTGTTGATGCTGATCTTGAAAGATGGGATAAACGGCAGCTTCCTGAAGTTATAGATTTAGTATTTGATTT

GTTTGGAGATATATTTTCTTCCAATGCATCAGATGCTGAAGCTGCCGTCTTTAAGCGCGCTTTGCCTGTG

CTTAAAGCAAATTTTCGAGATACTTTGCATTGTTGTGATGGTGTGGTTTATTGTAAACATTCTGGTAATC

CTTCTGGTGCTTTTCTTACATCTATTTATAATAATATAGCCAATTGGGTGTTGGGTTTGATTTGTGTAAA

ACTTGTTGTGTCCAATAGTATAGGCCGTAGTATTAGTTTATTGCATGTTTATAGAAATGTGGATATGTTA

ACAGGAGGAGATGATCAGGTGATGGTGTTTTCACCTGAGTTTGCCCAGTATGTAACATTTGAGAAACTCG

CAGAGGTGTATAAGTATTATTTCCAAATTGGATATACTGCCCCAGCCAAGGATGGCTCTACTTATACTTT

AAAACCTGTGTGGGAGTTGGATTTTCTTTCAAGGAGGATGGTGAGAGATACAGAAATTGAATCCCTGTAT

TATCCTGCCCTTGCGAAAACTAGTCTTGTGAAAATGCTGTCTTTTTCAGTTGATGCTGATGATCTTGATC

AATTGGTATCACAGATAGAGACAGTGCAGAAGGAAATTATATTCCACCCGCAAGATGTGTATATGAGTGT

GACTCGTGATATAATGTGGATTAAACAGAATATCCTTACACCTACCCACTGTCGGAGACTTGTTGTGGGT

ACGTATAGGAGAATGCGTGAGCAGTTTCTGGCTGAACATTATGGAGTCAGTGTTGGTAAAATCCCCAACA

TGGATCAGGGCCAGGTTATTTCTAACAGTGTTTTCCAGCGATTGAATTATCAATCAACCGATATGTCGAC

TGCAGAGACAATAAATAATGCAGATACCCTCCCCGAGCTGGTCAGCCGTACGGTTACACAGTCGGAGGAA

GGGAGTGCGTCCGTGCTCGCGAAGGATACAACCACCAGACGAGCAAGTAAGAAAAGCGAACGAGCAATTC

GTTACGGCCAAAGACGCGCGCGCCGTAACAATCACGCTTCCCGTGTCCCGAAATCAGCAGAGTCTGAAAT

GGGGGGCACGGCTAAGCAGGCGAACCAGGAGAATACCCGCAGACAAGTGTATGCGCACATTGGTAAAGCG

CTTCTCTCCGCACACATGGATGTGTGCCAGAGAGTTTTTGAGCAGAAGTCCAGTAACCAGACGGTATACG

TTGCCATAACACCTCGGCATCCGGTATACTCCGAATTAAAAGAAGCTAAGCAACAACTTGGTGCCAATTG

TCTTATTAGAGGAAATGACATTTATGTCAGATCCACTTCCCTGGAAGGTTTCTATAATTTTATGCAAACC

GACCGGATGTGTGGAACAGATTCTAAGAAGACACAATTGAAATTTGGATCCGCTTCAATGGTTTATCCTC

CTGTGACGGTGAACTCAGATATGACAATATTGGGACAGAAGGCTATAATGGCATTTCACTCTTTTGTAAA

GAGTAAGTGCACAAGCCTACCCCCAAATACTGTTGTGATGGGTCGCCGCCCCCAGATTATAAAACAAAGA

ATGCAATCCCAACCCGTTGATCATGTGAGTGGTGTGCGACTGAATATTGATAGCTCAAATAATGCTTGGA

TTGAGAATTCGAGCATCCAAAGTGCAGTGATGTCCTTTGGTGATGTAGCTTTTAATATTGTGAAACAGGC

CATGCAAGATATCTGCGTTGAGAAGTATGGTATGTTGACTTTAAAGCGAAACGATGCTTTGGTTCCCCTC

GATCCAGTGGTGGATGTGGGTGATGTAAAACCAAAATTCCGTATTGCTCCAAGTCCGCATGCTGGTGCTT

CACAGATGGGTGACACACCACCGTTGCCCACTCCTGGCCCTACTGAAGGGCCACTTTCCGCAGATCCTGT

ATTTGGAATGGATACGACCACCATGCCAGCTGCCTCAATTGGATTTCAACAAGGCTTTGTCCCACCCCAG

GAGATGTTCATGGGAGATGCTGTGCAGAATATGCTCAGCCTTGCCTATGACCATTTTGTGTGGTTGGATC

AGGTTGTTACTGTCTCTCAGGATGCTTTGCCCGGTACAGTGTTTGGTGTCTGGGAATTTGGTGAAGACCT

TTTCTCAGGCCCCATGCGCTGGTGGGCAAACTCGCATCAGAGATGGACAGGAGGCATTGATTTGAAGTTG

CATGTCGTAGCTCCTCAGCCACTTGTGGCTCAGTTGAGCTTTGGATTTGTGCCACAATATGATCCCGCAA

AAACCTATACTCTTCAGGACCTTAACGTATACAAGGATTGGAACGCTCAGGCCGTGCAGATGGCAGGAAA

TCTTGAATGGACCCTTGCGTGGTATGATCGCGCCGACAGATGGTGGGAGAAAGGAAAAACCCCAGCGTAT

CGTCCAGCTGTAGTAGGAATTATTACAGTTGGTCTTCAGAATCCCTTTGATAACCCTGCAGTTAACATTG

TTATTCGGTCCCAAACCCGTTTTTCTAAAGTGATCCCCTTTACAGCTATCATGCCAGATCTTTCTGTGAT

ACCATCTATGGAAGCTTTGTCAACAAATACTCCAGCAGAACTTGCATATCTTTCAGGTAGAACCTTTGGC

AACTTGGTCGATAGATTGAATGTAGATCCATTCTATCGCACTCAAGTTGCCTTGGCTCTTGACGGAAATA

TGCAAGAAACAATCCTGATGAATATACCAACCTATGATACCACTGCCGATATGTTGCAGATGCAGTATGA

TAGGAGTACTCCAACGGACTTGTCATTTCGACTTTTGAGGTCGGCTATAGGAAATAGCGGAGATGAAACT

CCAGACCGTCCGTGGATGATACCTAAGCGTGCTGTTATTAACGCACATCGGTTTGAGAAAGGTAGTGATA

ATACAGGATTTGCCATAGGAGGCCTTACTGGATCTTGGCCAAAGTATGCGTCGAGGTATTCCTTTCCGTA

TACTCCAGTCCAAGTTCAAGAAGGTCCAGCCTATGAAGAGAGTTATTTGCATTTTGAGTGGGCTGAATAT

CCGTTCTTTGATGAAACTACAACCACAGCAACCATGATCCCTTTGTATTATGCAGGGAGATGGAATGGCG

AAATTGATGTGGTGAATCCCCGTGTTAACACCTGGGTAGAATCTGATATTGAAGTAGGACCGGATATGTA

TGCCGCTGAATGTGCCGTTGTTGATGGAAAAATTGATGCGAGTGTTGGAACTGTTTTGCAAACGAATCGA

GCCGATTTTACCCAGATAGGTGGTAATTGGGTTGGAGTTGTTTTCCAGAACCCTGCTACGATCTTTGCCC

TTAAAGCAGATCCGATGGGGTTACCTGCTGGACATTACGACTTCATGTACCATTTTACTAACTATTTGGA

TGGGAAAATCCCAAATGGAATGGCATGGCAGTTTGCCCTCTACAACCCTAGTACTGGCGTGACAGTAGGT

CAGTTTCGGTACTTGCCAGAGTTGAAGAGATTTGTTACCCTAGCTTTTGATCAGGGGGCACCTACCAAGG

ACTTATATCGTGTATTTGGAGGGACAGACATCTCTGAGCTAGTGATAGCTCAGAGCTCGATTGTTCCTGT

GGCAACAACTATGCCACCAACCGATGTAACGTCCTGGGAGAAGCGTCTTCCTGAATCATTGCTAGTTAGC

AATGTGACTACCAAGTTTTCCATTGAAGATTTTCGTAATCGTGTGAAACGTGTTGATCAAGCAGTGAGAA

TGAATGCGCCAAAGATTGCACCCTTTGCTGGGGAAGCCCAAGCAGGCTGGGCGGCGCTCCTTGGAGGAGG

CGCCTTGTCTGGTATTGGGAATGCCCTAGGGAGTTATGCCAACTACTCCATGCAAAGTAAGCTGATGGAT

CGGCAATTTGGCAATCAGTCAAAGCTTCAAAAACAAGGCTTTGACTCCTGGCTATCTAATCAGAAGCAAT

TGCAGAGCTATTCGATGACATTGCGAGGGTTGTCCACCCCGGCAGCTCAACGCGCAATGTCAGCGGGTCG

TTCTGAACCCAGTGTAAATTCTCCGCCCGCTCCTGCGACCCCCGTTGCCCCAGCGTCTCCACCAGCCCCA

GCTCCAGTTGAATCTGTAGTTGGATCTGGTGGCGGCTCTGTTGCACAAAGCTTCGCACCTACTGGCGTTG

CCAGCCTGTCTAGCAGTAACAGTCCTGCAGTTAGCTCCTTGGAAGGAGCTACTGCCACCCACTCCCGAGC

TTGAATGTTTTTGTTGTGTTGTTTAGGCTGGAGGTGTCTTTAGTGAGATGCCTCGCACCTGTTAGAGCAG

GTATAATTATGCTCACATGAATGGTGAATAGCACCATCTCCTGTGGG

>JYFJ01025622.1 Catajapyx aquilonaris Contig25622_fixed, whole genome shotgun sequence//Chuvirus

CAAAGTCCCTGTTACAGGGTGAAAGATGTGCAAACTGTGACAGGAGTTATGAACCACTCCTTTTCTGGTG

TCAGACACCCACTATCAACCTACTGTCCCGTATTCTCTCCAGCCGCAGCAGCCAGCAGGACTACAACAGG

CTCCGCGTCCTCCAACGGATCAACTGTGCCCGGATGCCATGCCACATCGCCCGACTGAACGCTGCCTTGT

TCCGACGTACGATTCCATGATGTATGGTCGACGACTCAGCACGTCAGTGCCGATCACGGTGCTTGCCTTT

TGGGCGCCCTTTTGCCTTAAATAGAAGAGCAGGAACAGGATGACATTAACCTGTGCTACTGGCGATAAAC

TCAAAACGTCTGGTTGTTACTGGTTCAATCTGACAATAGGTAGTCATAAACTACAACACAACTTTTGGGT

CTCTGACATTAACATTGGTACAGATATTGATGGTATTATTGGAGTCGATTTCTTAGACTATTATCACGTC

AATATTGTGTTATCAAAGAAAGCAATAGACATATCTGGAGACTTAATTCCGTTAGTAGGAGAGAAGATTC

CTCAGAGTACTAGTATAGTAAGGTTGGATGGCAAATTTTCCCAGCATTTTTTTTTACAAGGTTAGGTTCA

CCATTTTAACCTAAGTTTTGGACCAATGCCATTTTTCAAATATCCTTCACCGTAAGTAAATAATATATTA

TCCTTTTCACGAGCATGCCAAAAATTACATGTCATTACGACTGCATACATTAAAATTTTGCATGGGTGTG

CAATCCCTTGAGGTTTCTGCTCTTACTATAAATCGACACACAGTCTTCTAAAACATATAATGAATTCTCT

CTGGCAAAATTTGTACTGAACATTCGTACAAGATACTAAATGGTCTAACAGGAACTGGATTGGATCACAA

TCGAATAATCCTCTGAGAAATCGTCTGGATTTCAATCTAGATCCCAATTTAATAGCCGCTTCTAGTTCTT

TATTCTTAACCAGGTGAATCAATTGTTTTCTCAGCCGGTTCTGAATCATACCATCCCCTGTTTGGTCTTT

ATAAAGAGCTACGCGGTAGGGATTGATTGGAAGTTTCTCACCGGGGCGATCTCTTCTGATATTACTACTA

GCCTTCGAAAGGAGAGCTTGGGACAGGTCCGGATCAACTGGGATCAAGAACATGTAGAGTGATAACCATA

CTGTCAGAAGATCATTTTCTCCCTTTGTTAGGTAGGATTGGTATTCCTCCAAACTCTTGAGGGTCCAGCA

ACATTAACAACAGAGATCTAGTTCTCAATTGCCTTATCTCGGGATGTGACAATTGGCAGTAATGAAGACA

TGACATAAATGAGACGATGGTGGGTTCCATATCCACACACAGACACACATTCAGCAAATACTCCCTTGAT

CATTTCGAGAGGTGTCGGAATGAGCAGGTTTGACAATCCGTGCATTTTTGCCCCTTTCTTTAATGTTATT

GCCACCCATGCTCCGTCTAGAAGATACACTTTCCCGAACCCCAGGAATCTTCGAGTCCAGTAAGTCTCTG

TCAATCTGACCTTAAATCCAAATTGATCATGTAAGTGGTTGTTGAAAGTTTCTAAGTATGCCTTTGGATC

ATCTTGGATGTCATGTTTCTGCACACTGACAACAAGTCTTGAGATCATCTCCATTTGCCATCAGAAAAGC

AGTAACTCCAGCCTGGTCACAGACATAACGAGCAACTGATTCATAAATCCAAGTCCAAAATTTCTGGTTC

AATCCCTCTATGCCTCCCTGTTGACCATCCCAAGAGTACATAGATGAAAAATCAGACAAGAAAACCAGTG

ACCTGTTAAATACATGCATTATATGCTGGAATTGTTATTGATCAAAAATTCTGTCAAATATTAATCTTCC

AACAGGCTCACATAATTCATTCCGGAAACAGTTGTTCCAGGCATCAATGTCAATTGATGTAAAAAAAAAA

AACACACGAAATAGTCATCTGATCTGTTTATCAGGCTATAATAAGCGTTCATCTTCTCAAACTTGCTTAA

CTCATTGGTGGTCATTGCTTGTGATGGGTTGTACAAGTCCATAAGGGTAGACATGATCTAATATGCATCT

GCGGGCTCTCTCAACGGCTGGTGATGATCCAAACATTCGTCCTTCCTGCTTTAATTATAATTCTTTTTGA

GTCAATATAATCACAAAATAATCAATTGCTTCAGGATCTTCCAAGTGCAATTTGCTCAAAATACTTCCTG

ACGGCTTGATGAGGATATTTGGTCGTTAAAAATCAGGATTCTCTTGATCATATGCCTCCAACAGCAGCGT

GTGTAAGACGGCAGCCGTCACTTGGTGTGCCTCTCTTGCCTTTTTAACGTGTGTCACCTGCAATGAAAGG

CTCTTTTAGCGGCGCCGTAACCACACTTCAAAATAATAATTACTAAACCTAAAGGAACACTCACCTTGAG

TAAGGCATCCGCTGCGCCACTCGTTGTGATTCCTGCGTTGACCACAATTGATGTCCATCCACAACCCTCC

ATATAACTACCCAGGACGCGAAAAAAATTCATTTCAATATGCAGAGGCCCAAGCAGGAACATAAATTGAA

TCGAGCAAAAGAAGAACAGCACATGTCACCAAATGCAACTTTTTCAGGAGAGACAAAAAAACTGATTACA

GAGCTTACCGTTCACGACTGACAGTCCATACTTTCGGCTATATTAGCAGACAGGCATACGCTTCCAGAAA

TGTAATTTGGTTCTCAATACATTAATTAACTATTGAGATAATGTAAGTTAAAATGTGGACTTGTGCTGTT

CTTCCTTAGGTTTTCCTTGAATTTTATTAACAAAATTAGAACAATGTGTCAGAATTAGAACACATTTATT

GTAAATCAAAACATGTGGTCAGGAAAAGACTCTGGTATAATTTATATTTTATAACATTCATTGGATCAAA

GGATATTGAAAGAAGAATATGGTAGCTATATGCTTGATGAAATGTGAACTCCTACTGGAAAAGTTGTTTA

TTTTCCTGACTAAGAAAATATCATTACAGTATTTCAGTACCCTTGGACATTATAAACTTGAACATTCTCT

TATTTAGAATGCTTCCATTTTTTTAGTTGTGTAGGCCTATAGTTTATTTTTAACTTGACTTATTAAAGAC

ATTGTACCACCGACTTCCTGTACCATACAGCTAATTCATCCCAGATATGTACAGAGCAATTTGCCTGCAG

AATCTACAAATAGTAGTGAATCCACTTTTCGTTTTACTGATAATAAAGCAGATAACTGGATTGTCATTGA

CTGCAAATTAATGTAATGGACAGAACTCACTACATGCTTTGTTGGGCAAGTTATACCAATTTATAGACCA

TCTCTACACATTATAGACAACATGTACATTGTAGTATAGCCTACATACACATTCCGTCTTCATGGCATTA

TATCCCATGACAGGGTGGCCAGCGATGACCATCAGGTATCACACCTGGCCATATCACATGATGGCAATAC

AATGAATCCACTGCTGTCTGCTCAGGTACACACATAATAGGTATGCATTAAGGCCATTTCACAAAAAAAT

GTCGCGCGGCAAACGGCGCGCGTTGCGGCAAAGTTATTATTTATATTTATTATTGCCAACAATTGGCAAC

AATAAATATAAATAAAAAATTTGCCGCAACGCGCGCCGTTTGCCGCGCGACATTTTTTTGTGAAATGGCC

TTAAGTCACGTTTCGAGTGTCGTGTAAGTCACGAAAGAAATGATTTATTTCTTGTAAAATTAATAACATA

ACACCAAGTTCACAATAATATTTTAATTTGGGTTTTTGAAAATATCCGCACCCCAATAACACTAACATTC

AAGGCATAAATGTGAAGAATTTTACATTCATATCTGAAGCCTCTTTTACCAGTGGCTTTCTTCTCGTGTA

AGTCACAGATTACTTTATACATTTATATAACTCAAATGAAATGTGCAACTGTTAAAATCAAAACATTAAC

ACTTAGATACAGGCTGCATATTTATGTTTAAACACCATTTCCTTAAATGCTACGTTTGTGCTTTAAAAAA

TTAAAAGTTAGGCATAAACTGAGTGACATGTCGTGTAAGTCACACGTGGAATTAGCCAAATATAATCTAT

TCTCCTTCATTCACGCCAACTTTCCTCTGTAGAGGTCAATGAGCTCTAGACATTATTTTGGCTGCGATAC

GAAAAAAATGCTCTCCTGAAAAATGTGTCACCTTGCAACTGGGCCCTTTTGCATATCCATGCTTCGATCT

TTTTCGTTGTTTTGTTACCCTCCCTCGCGACCCCAAATATTATGATCGCGACCCACAGTTTGGGAAGCCC

TGCCCTAAAGAAATACCTTACCTCACAAATCATCCGGTCGAATTCTTTCTGAATCACTGCCACTCCCTGC

GGAATTTCCTCCGTTTGAATAATAGCACTATGTAACACACTTTTTGTGTTTGGCAAGTGAGTGTTATGTC

CTGCTTTCTTATGCCTAAAAACTTGAGATAATGGCCATTATACCTGCCAAAGTTTGCCTTTGTGCTCAGG

AGAATGACTTATATATTTTGCATATCCCCATATACGTACTTGGGTAACAAATGTCAATTTTTATAAACAG

ACAATATTTAGAAATGTGTGGTTTTTCTAGATTTACGATTGTACTGCACATCACTTTCCCGAATTAGTCC

CCATATGTAATCTCCCATCATGCTACCATTGTACTGTCCTTGGTATCGACGTTCAAAGGTCATCATATCT

TGATGGAAGCGTTCGCCGTGCTCCTCTGAGTAATCTCCCATGTTCCGCTTGAAGTGATCCAAGTGAGCAT

CAAGGATATGGAGTTTGAGAGACATCCTGCAACCCATCTTGCCATAGTTCCTAACCAGAGTTTCAACCAG

CTTCACATTGTTGTGGGCCTTATGATTCCCAAGGAAACCTTTCACTATTGCCACAAAGCTGTTCCCCTGC

TATTTGTCGCGCGGCAAACGGCGCGCGTTGCGGCAAAGTTATTATTTATATTTATTATTGCCAACAATTG

GCAACAATAAATATAAATAAAAAATTTGCCGCAACGCGCGCCGTTTGCCGCGCGACAAATAGCAGGGGAA

CTTCGTAAAACCACCTTGGAGACCCATCAGGAATGCCACAATTTTAAAATCTCCTATGACCTCCCACCTG

TGCTCATCATACCTCAGTCGGTGCAGTAAAATCTTCACATTTTCATAGTCCTCTTTGAGATGCACTGACA

GTGAGTGCGCCAGGGGAATAGTAGGTAACGTGTTCCCATTATGAAGCAGGACAGCCTTGAGGCTCTTGCA

CGAACTGTCAATGAAAAGGCGCCACTCATTTTCACTGTATGTGATGCCGATTTCATCAAAAAGTCCTCTC

ACATCGCTGCAGAAACATAGTCCATCTTGACTGTTGAAGAAACTTGAAAAAACCTGGTGTCGCTTCCTCT

GGTCTGTTATATGTACACTTTCGTCCAAAAGGTTCCACTGCTTGAGCCTGGAGGTCAACAGCTCAGCATT

AGATTTTTTGAGTCCAAGATCTCTTATCAAATCATTCAGGTCTTGTTGATTCGGGAAATATGGCACTTGG

TCCGAAGCAGAGTCGGTGAAATTAGCATCTTCATCTCCGTCGCTGTCTGATTGGCCACTTTCATAGGAGG

ATAACTGTTCTTTTGCTGCTGGTGGAGTGGGCACTGGGTACTCTGTGCTGTGTGGCACTTGTGCGATTGA

TGAAGGAATGTCTGGATACACCGCTGCTTTTGCATTTTTACCAGATCGGCGTTTTGAAGGGTCCACCATG

CAGAAGTAACAGTTGCTTGTGTGGTCAGTAGGTTCCCTCCAAATTCGTGGAATTGCAAACCTCATGGCCC

TTTTCTCTCCTCTGTACCATGCTGCAGAGGAACAAAATTAAATGAATGTAAAACTAAGATATCTAGTGAG

CAACAACTACCTTAATTTTACCTTACCTTCTAGAGTTCTTATGCAGTTCACGCAGGTGAAATGAGGTGCC

CATGTTTTATCCTGATCTCCAACTGGCATTCCAAAATATGCCTTGTAGGCCTGACACATTCTCGATGATT

CTTGTACAGGGTACTTTTTCGCTCTTGTCTTGAGGAATTCACCACACACATAGCAAAATGAGTCCACTGG

ATGCTTGCAGCCTCTTGACGCCATGCTATGTACCTAGGTATACAGTATAGTGCGTTTGGTGTGCAGCATG

CTCCAGCAAAGCAAACCATACTGTCACTGTTGAGGGCGCTGTAGGAAACCGGAGCACTCTGAAATGATTG

GCAGCTCGAAGAGGGGAGACACGAGGGAAAAACTTATTTCGGGAACCACCTGCATACAAAAAATTGCTAG

GGTTGGGAATAAGAAATGATTGGAAGGTCAACGAGGGGACGAGATTCAGAGACGTGTGGTCACCCAAATC

ACATGGCCCCAACATATGGTGTGACTAGTGCAGTTGTGTTTATAAGTACAAGGGGGACATTTCCCGACAT

CTTCTGTGACGTGGGTCAAGCAGGTCAGTCTGATCTTCAATTTTAATGCATGCGGCTTTTTGTCGGCAAG

TGAGCGGGCGCCGAGCAGATCCGTCGGTGCGAAAAAAAAAGAGACGAAAATATGGGTACCCCTCAGGGAA

AAAGAGTCATTACCCAGCCCTAATTGATCGGATGGCCTTGGAAACCACTCTGAAATCTGATAAACAGCTA

AACGCAACTTTTTTGTGCATTTTCCAAAGGGAAATAATTTTAATTACTCTAATAAGTTAAAAAGAATTTA

GCCGGGTCCCCTTCTTCTGTGATCGCCCTGTATACATATGATTGTCCTGGTCACAAAAGCAAACTTTTTA

CACCATAATTGCCATTTTCTGTACTTTCTAGACACGGGGAAACTGAAACACACAATAAATGCCCAAACAC

AAACATAAAGTTAAAATTTGTTACATAGTGTAATCAACGAAATGCATAAGTGGCCTGAGAATATAGACCA

TAATAGCACTAACATGATGCGTAACTGAATGCATCCCTTGTATAATAGGTCGCAACACTATATCCTGCGG

CAACCCAGAAACTAACTTATGCAACTTAGGGATGCCATAAACCAGTGGAATTTGGAAATGCGTGAATTCC

AAAAACCTATTCACTAGCAAAAATGTGCAGAGTGTCCGGCAATTTTTTGGCTGGTCTTCCCCCAGTTTGG

AAGAGTCCAGAGCCACCGACGTCCAGCTCAAAAGTAGCCCCGTCGATCTTGAGTACATCTACTCGCAGTG

AGACACGATAGCCCCTATCTCTTGCAAGTGATAAGAACGGCATCAGCTTCCTCCGCCTTGCCCTTGTCAG

CTGGGAGAAGTCTTCACTTATGTGTATGTTAGTGTCCTTCAAATTTTTGCCGTTGAAAAGTATCGCTTGC

TTGTCAGGAAAACGTAAGAATTTTGCAATAACCGGTCTGGTGTACGGCCCAGTCGGTGGACCCTCTCAAA

TTCTATCTTCTTGGGGTCATCGTGCAGTTTGAATTTAGTGATGATGTTTCTAATTAGTGTCTCAGACTCC

GCCCAGGATTCTGTTGTTTTATCCTCTACACCGGCGTGAATGGTTCTGAAGGGTTGCCAACTGGTCGGTC

AAATCACGTACGCGGTGGTCCGTATCACTCAACTGGGACTCGATAGTGGTTACCTTCGTGGACACAATTG

AGAGGTCCTCCTGAATCCGGCCACAGCGGTCTTCAAGCTTCGATATCACCTGGTCCAGCCTGTCAACGAT

GCTTTGCTCCATCTTTGACAGGAGTTCCTGGATCTCGGTGATTGACGCCATTTTTTCGGAGTCGAATAGG

CAGAGCGAGACGAGAGGCGTGCGTCCAGGTCAGGAGCTAGGCGGCGTGTGATTTAGTTTACATTATTGTT

AACAGTGCTTACAATTTGGTTTGTGTTCGATTGCAATTTTTCCATTGTATTGTTCATAGTATTCAGAGTT

TTGTTGGTTTTTCATTGCTATTTATTGTTCTGCCTATGGCGCAGAGTTTGGTAGGTGCTTGGTCCGATTT

TGGTCGTACTTACTTTTGAGTACGCAACTATTTCTCTCTCTCTCTCTCCCTCCCCCTCCCTTCCCCCTCC

TCCACTCAATCTACTCCTCCTCCTTATCCTCAGCAGCCATCATCTCATCCTGAGCAGCCTTCTTCTTATT

TTTTTGTCATCAGCAGCCATCTTCTTGGCCAATAATATTCTATCCGGTTCCCGCAGAGCCAACACACTTA

TAGATGGAGAATCCGTAAGTTCCGGAAGCATGCAGTCAAAATAAAATTGCTCCAATTTGAGAATTATCTG

GGAAGTAAACTCCTCGTCTCGGAATATCTTGACCGTGACCATGTCACTGGGAAGCCAAACCACGAAGTCA

GTGAATTAGGTTGCAGCACAGTAATTGCATCTGGATTTGATAATAATATGAATGCTGCTTCGGCAAATAT

AACTTTCCCTCCTCTTCCACCTTTTTGGAGGGGAAAATGGCTGATGCTTTCCACTGCTTCCGCGATGCTA

ACACATCTCCTTGACGTCCATGGGCACTTTACCTCCAGGATGAAGGAATCATCTATGTTCTCTCCATGGA

CAATCCCGTCTGGTGTCGCTCCGAGGAAGGGGTATTCTTTATCCACGTAAAGCCCGTACTTACTGCCAGT

TTCCATTTCATATTGAGCTATTGCCTGTTCCTCATGGGCAAGGCCCCATGCCATGGCTTCTGTTTGCTTG

AGGGATGAGTACAAAATCCTCCGCACCAGACCTGCAGAGAAGGTAGTAGACTTCTGGCACGCCACCTGCT

TTGCGTACACTCTTAGTTGCGGAGGCCATGACGGTAAAATCCACATCAAAAGGCCGGGGATACAGAGACT

TGCTGACTGATTTGTGAAAGGGAAAACTCATAAGCCTGGCATTGAGTTGTTTCCGAGTGAAGATGTGCCT

TTCAGAAACAGCTACCCTAAGAAAAAGCATAATTTCTTTTGGTCCGAAGGCTTCTAACACAACATGCATG

GGGTCATACAGTAGGTCTTTTGTAATCGAAAAGTGCGCAATATCATTTAAAGACAGACCGACCATTTAAT

CCACACTCTTGAGACATGTGTCGCTTGTCTTTCGCGGACTTGCAGTTTTCTATGGCCTCTACTTGTCTTT

TCACTTCAGCATCTGTGCGCAGGTGGCATTGCCCATGATACGTGAGAGTGGCATAATCAGCACTACGTGC

TAAGCAGGACCTACAGGGTCGTAAGACATTTGGACTGAATGATTCCTTGAACCCTGCGATGTTGTGGCAT

GCCAAAGCATCGCCAGTGTGACAGGGATGGACACAAAGACGAACAAACGTGGGTTTCGCTTAAATGAATG

GTGTTTATTTTACCGTAGCTCAGAGGTACAAAAATAGGGGTAGGGAGAAATAGGAATATCAGAAGGATGG

GAGAAGGGAGTGACCAGTCCCCGGGATATTGTCCCAGGAACTGGCCACGAGCGAAACGGGTGACGGGTTT

TTATAGTGGGCAGAACAGGAGTGATGCATTGTGGGTACTAGGCATGTATTAGGCGAGAGTGTGGGTTGAA

TTATAATGGGTCGGAGAAGGGATCAGTACGGGGAGTGGTGCACTGTGGGTAGGAGGTCGAGTTTGATCGT

GTAAAAAGAGGCACGGGAGTGGTGCACTGTGGGTGGGATGTAAGTTTGGTCAAATAGGGGGTGTTCCAGA

AACAGCTCAATACTGTGATGGGTAATACTGTTACACCAGCATATGCCAGGAGGCACCCATGAAAACGTTC

CTCTCCCTCGCATGTCTTCAACATAACCCCCTCATCCATAGATAAATTATTTACAGTAGCCACAAAATCT

TGCAATAGTTCCTTTTTCATGCTGGCCAGTTTTAGGTCACTAGACTCTCCTACACCTAATAAATGAATAT

GGCACAGCTTAGATCGTAGAACAGGTGGGACATTCAGGAACACCACATAAAAGAAACACATTTTACCCCT

TCCGCCACGTTTCATCCCCAATGGATTGGCGAGTTCTACGTCATCATTGTAGAGGAGCATCTTAATCAGA

TTTCCCTGCTCGTAATACTTTTGTGCAATGGGATGTGTATGAAACTTACAACCATCTGTAAAGTCTTTCA

TCAACCCTGCTTCCCCTTGGTGTGATGATTGCCTAAAAATGGTAAGCATTTCCTTGCATTTCAGCAAATG

TTCTGATAGAGCCACAAGAGGGATTGCGTATGCTTTTCGACTACTAACAGAGAGCAAAACCTCACGTGGT

TCTACATACAAGCCCTGCTGACGATACAGTGCTTTCCTGCTTTTGTCCCTTTTCAACCGCTTCAGGTCAA

GCTCAATACCACTTATGTCAATGATGTTTGATACATGAGCCACCACAGAGTCAATTAGCACTTTTGAGGC

TCTGTGATGTCCCTCTAATTGCCTGCACATTTTCTCTACTTCTAACTGCACTTTTTCTTCTACTCGCACT

GAATAATCTTTTGATCCACCAATATTACTACCATTTGTCTTTCCGCTATGTCAGTTGCCTGAGTAGCACT

CGGCACTAAAAAGCATCTATCATGCCGATTATGTGCTTCATACCGTACAGGGTACTCAAAAGTAACCGCT

TATTTTGGCTGATACCGCTATAATGCATGCGCGCATCGTCTCCGCGCATCACGTGATATACAACAACGAA

TTCAAACGTTACCTGTAACATCTAGATAATATCTAAGCCTGCTTACTTAGACGTTACATGAGAGGTCTGC

AACGTCTCCGCCTTGGAGACGTTACAACGTAATATCTAACCCTGTATATATTCGACCAGACGTTCCCAGA

TACGTTTAAAACATGTAGTAACGTCTAACATTACACAATTCACAGCTTTTGTAACGACTAAAGTGTGACA

TGAACGTTACATGGACGTTACATGGACGTTACCAGTCTAAGAGTGCATGAACCGTTCAGCTGGGTTGCTG

GTCTTATGCTGAACCAAAGAGTCTGAGAGAGAAGAGAGCCGAATGACGGCGGATCCTATTATTTCAGCCA

ACATTCCCACTTTTTTTAACGTGTCCACTTGACTCTCGCTTCCGTCTTCTTTCTGGAGGCAATCATCTGC

ACAAGAGGTATGCTGGCCAAATACGTGTAAGGGCAAGTTCTTTAAATCCATTTTCAGTTTTTCTGCATCA

CCTTCATCGGTCACATTGTCTATCATCGACTTGTCTGTATACGTCAATGATCCCATATTGGTCTTCAGTA

ACTTTCTAGCGGCAAGTCCATCTGGGCCTGGATAGGAGCGAACGTTTGCGGCTGTCCTCTCCAGTTTGCT

ACGAAAGTTTCGCAGAGCATGATTTACGCACTCCACCTTCGTAACTGGGAGATGTCCTGCGTATTTGACG

CGTTCCCTATGAATCCCTATGAATCACCGATAAATCGCCGTCACCCACGATGCTGTTGCAAATTATGCCG

TGCTCTTCATAAAGCCTATTCAAACCGTCAACAATTATGTCAGATTCCATGGGCGTGGAAGACTCGGACC

AGTCGGCATTGCAAGCATTGTCAAGGGGAGTCCTTTGGCTTCTGCATATTTATATATTGCGCACCCCCGC

AAAGAGGACTTTCCCTGTGACCCTGTTTAGAGGCTACCATTTACGTGCAATCCGAACCAAAGTGCATTAT

TATGTCAACGATTAGGCCGTAAGCGGACTATGGCGGGAGCACATTTTTGTACACGTTTTCTCTATTATTT

TCATGCCAAGGCATCGGAGGATCATAACACCCTCTAATTGATATCTGGGGAATGATTTGGTGATTCCAAC

GATGTGAAAAAATCGCACATGTTTTTGTTTGACAGTATAGTAAATGGTGGGGGAGAACCCAGCTGTCCTT

CGTCCAGATGTCAATCACTTATTCCCTCGAAATTATCCACTCTGATTTAGATAACACTGATCTACAACGC

GCCTAGGGCAGGCAGGTAAACTCAAAAGGGAATAGGTAACATCGCACAAAAAGGGAGGGGGGGGGGAGGA

TGCTTGCATCATTCGGCCCCAGGTCCAGCGGGGTCCTCCTCCATCGGGCTGGCACCCAGCTGGGTGTCTT

CCACCGCCGTCGCAGTCACAGGAGCAACACCGCCGTCTGCCACTCCCACTGCTGCTGCTGCCCGCGGGTC

GCTGGTCCCCAGATGACCGGCCCGTCCCCTCCAGCCGGTTACCTCCGTCCGCAGGATGTCCAACTGCCAC

CTCAAGGCTCGGATCTCCTCCCCAACAACTGCGATCACTGGCGGACGATCTCTCTCAGTTGGTGCAGCCA

TCGAAAGCTTACGCACGCCTGAGGTATGTCCTGTGGTGGCAAGTCCACAGGGTTGTGGCCCGCATCGAGG

GTGATCCCCATCTCTGCAAGCAGCCTGGCCTGTGAAAGTGTCAACTTCTGGGCGGGGTACCACCCCAGTA

AGTTGACGTTCGCTTGCCACGTCTCTTGGGCACCGGCTACCGGTGCACCAAAGTGAGCGTGTCTCCACAC

GTCCAAGTTCCATTCCGGGTCGAATCCTGGATTCATGGTGGCCATCAGATCTGAGTACAGCCTCGCGACG

AGCACACCTGGTGTCATTACACCCATGTACATCGGCAGGGTATCGTCTGTCATGTTCCCCCACATTCCAG

GAATCCGCACGCCGCCATATCGTGCACTGTGCACTGGCACTCTGTCCACATTCCAGTACATCGTCTGGCC

TGATCGGGTATCCAGATCTCCTATCATCGATAGGGCGTAGTAAATAGGGAGTGGTGTTTCGGTCTTTCCC

AATTCCATCCCGTGGATAACATTGAAGTACCGTGGTGGTAGCTGTCCATGGTGCTCCAATAGGGCGGCTT

TCCGTGCCCATAGCAGCATTGAGCAAAAGAGCTCGAACTGCGTCAGACTCATCCTCTCTGCCCAGGCCGG

CGCGTTCCTCGCCATCTGACAGTATGTCTCTCTGACTATTGAGACGAAGCCTGAATCGTCGGTGGTATAG

TCGTTTCCACGTTGGGCGCCACTTTCGCGATGGATTAACTCGTAGGTCTTACCCAGGGCGCTGAAACCGA

AGTACACACTCACTGTTTCGGGACAATCTCTCCTTTTATTCCTCTAAGCGTCTATATACATGTCTAGTCT

CTTATGCTCTCCTGGGCTCTCTGCCACCTTTCCCCCCTTTCCCCACCTCCACGACCCACAACCCCCAACA

CGAGGGTGCGGCCGAGGAGGGGAGAGGGACCAGCGGCGGGCGGCGCAAACTCCCGCCGTTATAATCTGTC

AAAGGGGAACACCATCCCGACGCCTTTTGATGATCTTCTCTCTCAGTGCAACAGACAACGCTCTTCTCGC

CATGGTTCTTCGCTGTGATGAGTCATAGGCAGCGATTTATCATGCCTTTTTTAGCCAAGATGGGCTGATT

CCCCCCTTCCACCAATTCCAAGCACAGTATATAGTTTCGTAAAGCATAATGAAATTGATGATCATTCATT

GCACCTCTTCCTGTCCTGGTGTCTCCTCTTCAGACTGATGGCAGAGTACGTATCCCATACGACGTCTACG

CGGAGCACGCCCAGCTTTACACTGGCATCCCTGATGTGCTTCGCTAGGTAGGCAGCGTAGTCACTGAAGC

TGCGCATAGTGTCAGGAGGTTTGAAGCTGTGGACTAGTACTGCTCCATCAAACACGATGCCGTCTGCAAA

TTCAGGATTTGTGTTTGGTGCCAACTTTTACAGGACTTGGACTAAGTGTGACTTGTTGCCACTTCGCATC

ATTCCATTCACTGACACTGAAGGAGGATAAGCTTGATTTTCATATTCAAAGAATGAGTCTAAATTGAGTG

CCCTGGCAGTAGAGATTGTGAACAATCTAGAGTACAGCTGTACTTCAGATCTAAGTGTCCTTAAAGGCGA

CGCTCCGCTAAATATATGAGGAGTTGGATCGAAAAACTTGATAACCCTCACAGGCACACTTTTGAGAAAA

GTGCATTTTTGTATATTTTTGCATCGCACGAATCACAATCCTCTCACAAATGTGAAATCCGGCGTGAAGT

AGCAATGACCTTGGTCGTGTCCAAGGTCATGAATGCCAAGATTGTAAGCCCAAATTTTGCTCCTCATCCT

CTCCAAGTCGTCCATTTCAGACAACTTCGCGTCACAGATGAAGCATTTCATAGATGTTGTTGTCACCCAA

ACGGGTGTAAGGATACGACAGGGTTCAGCTTAAGGTATATAGTCTGGTGACCCGGGGTAGCTTGCCCCCT

GGGGTAACTGGTTGTTTACTGCTATCCTTTTACATATGTTGTCATGGTGTTTGCTTACCCGGGGTCATCA

GACTAGGGCCCTAAGTCCTCTTTTATTGGCGTGTCAAGGGTTAGGAAATGATATCAAGGTAAGGTATAGG

GTGGAATGACCAGCGGGTCCCCCATCAAGGAGGAGCACCGACTGGCCTCGAGTAGTATAATGCGACAGTA

TTTATACCAACGATATAATATGGCATGATGGGTAGATTTATCAAGGCGAGCGGTCAGGATAGCCAAAGTG

TATATCGGTTGATCAAGGACAAAGTGATGGACAGGAGGAGAGAGTAAAGTTGGTTCAGGGAAGCATTGAA

TCGGCGTCAAAAAGGACTTTTGGTCACATTGTGTTTGTCAACTCATTGCCAACCTGTCAAGAATGTAATA

ATAAAATTATGCCTCTAGATATCAACATAACTTGCGTGTATACGGAAGAATGCTAATAATACTGACCTTT

CCGTCCACCATTGTCATCATTAGGGAAAAATTGATGGTATCTGGTTCCACTTGAAGGGGCTGCAGGTTGG

CTATTTGGTTCTCTATGTACTCTTCTTTTTTAATGGTTTCCTTTGATTCTTGTATCCATTGCAAGTGGAT

GGGGCGGCAGAATGAGGACGGGCGAGGGTTTTCCCAATGGTGTTGTCCGGTGTTGTCATCAGTGAGACGG

AGAGTAACCAGGGCAGTAGTAAATAAACTTTCATCTGAAGAAGCAGTACTTCCTTATTTCCATTTCTGCT

TATATCGGGAGTGACCCGAGCTGCCATCAAATCCCCACTTGCAAGTACAGAGTGCAAGTCGTTGGAGACT

CAAAAGGAGGCAACATTTCCAAAATCCTGGTGGTAGTGTGATATAGTGGAGCATCGATTATCCGAACAGA

GTCTGCCAAGGGGGTGTTCGGATAACTGATTGTTCGGATAACCGATCATTTACGGAAAAAAATGTACCGG

TGTCATAGGAGCAATATGAAACTAAGAAACAGTGATATTAACCACAAAACTATATTTTCTGCACAGGCAG

GTCTTAAAAATACTGTACATATACCTTACTGTCCTTACATGTAGTCCTGCAGTTTAGGGGGGGAAACGGC

GTTTTTTTGTTTTTTGGGTGGTTTTGGTTGTTTTTTTGGTTTTTTGCCGACCCGAAAACGTTCGGTTAAC

CGATGGTTCGGTTGATCGGTGTTCGGATAATCGATGCTCCACTGTAGTATAAAACACGGCACTCGGGAGT

TAAGAAGTGAAAAAAAGCACACAAGGCCCGGTATGTTGGGAATCCAGTATAGTACCTTACGTCCGAGTCA

CTTTTGTGGAATCTCAGTAAGGTGAAGCTGGTCTTGTCACGCATGTGTAGCAAATCCACGTACTTTTTTC

GTATTTCCACATCACCACCCGAACTTTCACTTGATGATTGCCCGTCACTTGCCTTTTCAGAATCGGAATG

CACCACCTACTTCTCTCCCACACCGGCCCTCACCCCAGGGCACCTTTGACAAACAATCTAACCTCAGCGA

TTATCCTGCTCATGTCCCAGTTGCTAGACAACTGTTAGAACAACAATATTACCCAAGATGCCTCAGCCTG

GCTAATGGAAAAGGATAGGGAGTAGGTCCTCTAGGTATACATAGCAGAACTCTTTGAGCACAACACGTAC

CAAGCACATTTCCGCTGTCCCTCCATTTATCATCATGCTGAGTTCTGCCACGTAGCTGGCTACATTGGGA

GAAGTGTCATTCGAACAGGGCCTGCAAGAACAAATAGAAGTGCCTCGCTCTCGTTGAAACTCAACCCCTC

CAGCTGCCCGACGACTTGCTGCTCGCAGGGACACCCTCTGCGCCGGGATACCGGGGAGTTTATCCGTTTT

CACAAATTACTGTGGGTGATGGTAACCCGCTACTTGTGCGGCTTTAGTGTGCAAACAATGGAGAGGTTTT

TTTCTGCGGAGTTGGGCGAGTTCA

>GAXI02021960.1 TSA: Tetrodontophora bielanensis C169527_a_7_0_l_1133 transcribed RNA sequence//Narnavirus

GCAACGAGGCGGGCAGGTTGAAGAACTTGCACGCATTTGTAAGGAGGAAATCCTTACAACCGTCTTTGAT

TTAAAGACGATGAACCGGTGGAGTGTTACACCCCGACCGGTGAGGACGTCGCAAGACGTCCTCCAGTGGG

CCCTCTGCAGGGCCACTGATAACCGTCAGGATATCCTGAGGGTTAGGACTCACTGTGTAAGTGAGCCCTC

TAAAGCAAGGGTTATAACCGTTGCTACCATGCCTTATTTGATATTAACGCATGTGTTATCGCACCTGTGG

AAAACAGTTGCGAACTCCCCGCAAGCGCGGGGAGGGATGACGTCTACACGTCATCTATGGAGGTTCCTCT

GGAAGGATCTCCATCCCCAGAATGATATCTGGGGCTCACTCCGGGTATCCGGAGTGACCTTCTTCTCCAG

ACCGGAGGAAGAAGGAATGATCTGGGCTCTAAGCACAGATCTTGAAACGGCGACCGATTATGGAAACCGT

TCGGTCTCGAGGCAAATCCTCGGGGCCCTTCTGCGGAGATCCGCAGAAAATCCGGAGTTTCCCTCCGGAT

TCGCCCAGTTTGTTATTGGGCTGTATCAGTCGGCTAGGCCGATTGATAATTTACCAGGGGTTGAAACAAC

CCGTGGATGGTTTATGGGAGATCCATTGACCAAGCTAGTGTTAACACTAGCGCAACAATACTGTTATAAC

AGTATTGGAGCTGGCGTTGCCGCCAGCTTTGTGGGCGATGACTTAATCGCGCTTTCTCCAGATAGAAATC

TATTGAAGAATTACCTAGATAAACTCCAGGCAATGGATTTTAAGGTATCTGAACCAGATACCTACATAAG

TACAAAACTTATGTTTTACTGCGAGGAGGCCGCAAAGGTCCCCTACAAACCAACAAAGTCACTTGTGGTG

CAAATGCTCCGTGGAAAACGGACCATAGGGTATATTGATTACCCACGCATAAGACTATTGTTACCGCTAA

CAAGCGACAACTATAGGGAATCCTATACGAACCTTGGAAGAACAATCCTCCTAGGTCAAGAAGTGGTCTG

GTGTCACCAGACCAATCGCGGGATACTACCCGCGATGATAAGGGCTGTAGTGTATCAACACATAACAGTC

CCTCAGGACAGGG

>GAYN02051120.1 TSA: Campodea augens C259464_a_47_0_l_2383 transcribed RNA sequence//Negevirus

GACAAAGAGCTGATAGCCCTATGGTATAATGCACACGAGTGCACAACACTCGTTGATCGGGCCGGTGGTG

TTAGCAGTCGCGTGGTGTGGCAACGTAAGTCCGGTGATGCCTCAACGTATATCGGTAACACGATGGCGCT

AATGGGCGTCATCGCTGTTATCTTTGACTGTAAGAGCATGCTCTATGGAGCATTCTCGGGTGACGATTCT

TTGTTAGTGGGACCGGGAATGGAAGTCGACAGGACTGATTTGTGTGCTCGATTGTTTAACCTTGAGTCAA

AGTTTTTCTTCTCATTCGATTACTATTATTTCTGTTCAAAGTTTCTCATTCCTGTTAACGGGCGATGGGT

GTTCGTGCCGGACCCAGTCAAATTCATAACGAAGCTTGGCAGGTCTGACCTTGCTAATCACGATCATGTC

GAAGAATACAGAGTGTCGTTATGTGATTTGATAGTGGATTATAAGAATGCTGCTGTATACCCCGTTCTCT

CCGCAGCTGTGACAGAGAGGTACCGAACAGTGATAAGTGACCATTCATACGTTTTTTCCAGCCTGGTAAA

CATCATTATAAATAAGACGACATTTCATTCATTATGGTACTCTGAGGACCGCGACGTCCTGTGTCATGAT

CCGTCGCGGTCGAAATTGTGACAAGGCGTAAAGCCAGAAGTTAAGTGTCGTGTAGGTATTATGGAAGTGC

TCATTGTTGATTTGTGTAATTTATTGCTGAAGGTCGACATCACCCTATACTTCCATCGCACGAAGGTAAT

ATACATTGCCTCATTTATCTCTCACGCGCTCTTAACGGCTAAGGTAAAAACAGATTTGGCTGAGGAATTG

TTGAGTGTGGTTTACACTCCATTTGAATACTTGACAGACGCAGTTGTTGATCGTTACGTTGCTGCGTTGT

GCGGTATTAAAAGTCCATTGAGTGATTTAAGCGATCACTGCGATTTGTGTTATTTGCATCTTTGTATCTG

TAACGTTCGACGGTTGAACGTAGAAATGGCTGCAGGACGCAATCCACGCGGCGAAGTCGCACCCGGTTAT

AGGTATTGGGGGGAACAGTGTTTACAATTGGAATGCGGCGATTGGGTTCGCCTACAGGATTTAAGTATTG

CGGTAGCCACCCTCAAGAAGTTGAAGTTTCATCTGAACTCGCACAGAGAGAAGGTTATCCCAATTATGGA

CAATATACCCAGAGTGCAAGTCACAAGACGGTCTAGGACGCCTATGACCACAATCCTTGATCTCAATTGT

GAGATATACGCGACCATACTGGCTCGGCTATATACTGCCGTCGATTTTGGTGACAAAGCTGTGGAGAGGG

ACGTCGTTGCATCTGAGAAGTTTGCCTCAGTCAACGATGCTCGTCGGTCACTAGCGACGGCCCTCTCGGA

GTTGGGGTCATTATCCTCTACGGCCACTTCACCACGAGGTCGAGCTGCTATCGGGTTGTTTTCTAGGGAA

CAACTTGAACGCAAATTCGCGATTAGATGGGTAAACGACTTGCCTCTCGACCAGGAGGATGTGGAGGATG

AACCAGGCCAGGCAGCATTCGCAGAGCGGATGGGTCGGCTTCTCGGAGATCTCGATTTGGATGGTGCAGA

GGCTGGACCGGTGCAGGGCGCAGCAGCTTTGCCACAACCCCGCCAAGATGTAGATCAGGATGCTCCCGCG

GCCGGTGGGCGCGATGAGCGTCGTAATGCAATCTGAAACGAGGAAGGATTGCGTGGACGCGAGCGAGCGG

GTCGCGCAATCCGCTTCGTGGAAGGTGAGGAGGGGGCTGGAGTGATCGAAGTTAATGACCTGATACTGTT

TCCTGACCTACCTGTGCCTTTCCAGGAAGGTGCCCATGTGGCACTTGGGGCTGATGATGGTGAAGAGAAT

AGTGGTGATGAAGGGTCATGTAATTCTATGTGTGTTGATGAGGAGTTGAGTGGGAGTGAAAATGGCAGTT

CTACGTCAACCCCTGAGTTGCATTCGCCTTGCCAGGAAGGTGCAGACTTTGCACCTAGGGCCGATGACGA

CGATGGTGATGACGGAAGTGGTGGACTGTTATATTTCATGTGAGTGTAGCGGCATTTGGACCATTGACGC

GGTCGTCCTATGATCCAGTTAGGTTTGGAGTAGGTTGAGTCGGTAAAAGATGCTGCCGTAGTGTAAAGCG

TTCTGAGTGAACGAACACAATGTTGGTAATGAGAGTTCTTTTGCGGGGATTCTATTTAGTTTTTGGGGGT

TTTGTTTCTTGGTGATACCAAGTTTTCAGGGACCGGGCTTTCGAGGGCCCTATTAGGTTTAGTGTGTTTT

CCTGTACAGATTTATATATTAGGTTAAGTTTCTTTCATTATATTCATTAGATTAGTTAAAATAAAATAAA

AAT

>JYFJ02009787.1 (replaced seq JFFJ01078906) Catajapyx aquilonaris contig_9787, whole genome shotgun sequence/Negevirus

ACAAGTGGAGTATATTTGGTGACTTCATTGCCAGTGAGCTGCGACAATGTCCTTACATGGATCTACAGAT

GGAGGCTAAAATGGACATAAGTGACGTGCTTTGCAAGCTACGGAGGTCCATTCATGACATAACTATTTTC

AGACCACCAGCATCCCCACACTATCCCTCACCGACAACCTCTTCAACTCTGGACCCATTGCACATCTCAG

TATGTGTACAGGGTTAGAAGGTTTAACTGTTTGTGCAACAAGTGCGAGTGCCCTGATTGGTGCAATTGAA

AATTTACATAACCACCCCCGACTGGACCTGGCAGTGCGGCGAGAGAGTGGGGCAAATTGGAGTCCGCTGT

TTCCTAGGGAAGAAGTGGTCCACAATCTACGGGAAATAGATTTGGGTAGGGCCAAAGGCATTGACGACAG

ATGCATCTCCACTATTGCGCATAAGTGTCCCGAATTGACCAGGAGATCCTTGGTAAATGACAAGATTACT

GAAAATGGTCTAGACTCAAGAGGATTTTCTGAACAGAGTTGCCAAACTCAAGTATCTCTGGGTCATATTT

GACCCTCAGTCTGCCAAATTATGAAGAAAACTTGTTTATTTTCAGAGGCTCTCCCGGCGATTATGCGCCT

TGGAAAAGTCTAAGGAGACGGCGAAAAAGAACGACAAGATCGTGCAAGTGTTAAGGAAGGCATATATCAG

CAGCGAAGAGAGCGGGGACGAGGCGGGACACTGTCAGAGTGTTTGGTGTGAAGGAGCTACCATGGCAAAG

CCACAGGGTCACCAAATGGTTCCACAAATTGGATGAGTGCCACGCACTGGGCACCACAAAAAGAGAGGGA

GATCTGATGGCGAAGTGCCGTCCATCGGAGGTGTGCTCTCTGAGGCAACCTCTGATGAACACCTTCAAAA

GTTCTTCACGGAGCAAAGTAGGTTGTAATTCAGCCAATTCACGGAGCAAAGTAGGTTGTAATTCAGCCAA

TCAGGCACATAACAGCTTCCCTGATATTGTCACTTGGCCATGAAAACTCAGCAAGATATATACATATAGT

GCGTGTCATCCTTGCTGAGTAAGCCAAAATCTTTAGTAACATTGAGTTAGTCTTGCGAAACCTTTGATGT

AACATGTTAAACTATTTTCTTGTGCGTGGCAAAGGTACCTTTCAATTTTACCGTATCATCATTAAATAGG

AAATGCGTCATCTGATGTAGAGCAACATATGACGCATTTCAGCACATAAAAACTAACCGTATCCAATCAA

GTCGAGATCGGTAGTGTCAGGTGTCCCAATGTACGTCAATAGACATCGGTGTGCGTCATGGGACACCGTC

ATAAAGTAGTTAACACGTGGAAGTTAAGTATAAAAGACAAAGTTTGAGCAGTCTTGAGAGCAGAGATCTC

CAGGGCACAGCACTGCGATTACTCAGAAAATCGACGACATTCTCCCTCCGTGGACATCAGAATACAGTAA

GTGTTCACATTTTACTCCCCGTATATTTGGATGGAAGTTGTAATCATTTTTAGCCAGTGTCAACGACAAC

TTCTTGCAATAAATACAGTTTGTTACTCTTACTGGTGTACAAGAACTATTTTCTCACTTTGCTCCACAGA

AAACTATAAAGTCGACTGAACCTACCATTAAATAAATTAATGTCGATGACACATGCATGAAACAATGAGG

TGTAATGCCTGAATCATTTAGACATGAAGCTCTGAATGTACCAAGTCACGTAGCAAGTGTGTAGATTTCA

TCACCTAATATCAATATTATGTGGCTAGGTTTGAAGTGACCTTAAGTGCCACGCTCGGGCCAAGCATGCT

GTTCTGCTGAAGGCGCAAACGAGCACAGGTCAAGGAACCCACTATGTCCCTCCTCTGGCCAATGAAGAGG

CGCGCGCATAATGGCAGTAATAGGCAGCGTGATGTCAGTGGGGGGACCCAAAGGTGGCCACTCTTGGCCT

GTGTTGTAATGGAGGGAAAGCCAGTTACTTGGTTTGCACTCATTTTCAACAACAGCCGTATACCTCACTT

TGGAAAATATCACTGTGGCAGGCCTCAATCACGCATTCCTTTTTACTTACGTGTATAAATATGTCATACA

CTAACAAACTGATATGCACAGGTTTTTCTATGTGAAGTCCGCTGAATAATGAAAATCTTTTTAACTGGCT

GTGGTTCTCATGAAGACATATCCATAGATCTTATTAGCCAGCCTTTCAATTAATTATGTGGACTTTGCAT

TTTTCATTGTAATGGCACCACTCCAGTGGATATAACAGCTGCGTATTCCTGAAGCACTGGAAAAACCGCC

CCTTCAAGTACTACTGTATGTGCTGCAAAAAGTTGGAATTAGCCATATGGAGGTGTTGCCAACATCTTGC

AGCATATTGTACATAGGTTATTCTGAATTATCATATTCATCACTTTCCACACAGGTAGAGCCAGTTGAAG

AAGATGACGGCCACCGAGATGGGGATGATGATATGACCCCACATCAGGCTTCCACTCCACAACCGCAGGC

ATGGGGTCCAAGTTCTACAAGGGGAGCTGTGTGGTCCGCTGCGTTAGAGCAGGACCGGGACACTCATGAC

CGGTAGTATACTCTGAAAGAGGAGTACTACAAAAAACAACTGGCATTGGAGGAGAGGACGACAGTGGCCC

CGGAGGAGAAGGTGAAGTTGGCTAAAAGGAAGACAGTGGCACCTGAAAACCAGTCTGCTGCAAGTAAGCA

GTTCACGACAGAGGAAGCCAATAGTTCCCATTTGGTGACCAAGGTACGCTGGGTGGTAGAAGCAGTCAAT

GGCCACATCAAAAACTTCTGCGTGATACAGTACCCAGTGCCATGGTGGACCAAACTGGAGAGTTGGTAAG

GATTGTTTGCGCCTTGTGTAATGCCTTCCGCCCGGAATTCATCTCATCTAGTAAGGGAGATGAGCATGAA

GCTCTCGCTCGGAAGATGTTAGAGCAAGTTCGGAACATGAACCCACTCCAAGAGCTTGCGTCGAAGAAGG

CGTTGACAGCCGCGGGGGGAGTTGGGTCATTAGTGACCCGGCTGCTGTTCCTGACTTCCCCCGCCCGCCT

AACGGAGGAGGAGGTCAGAGGACTCACATTGGGAGTCTACCAAATGTGGCAAGCTAGGAGCTATGTGAGT

GAGCACATGAACAAAGATGGAGATTATGAGCTGCTCTGTCATAAGGCAGCGCCTGATCTCATCTGACGTT

GAGGAGGAGGCTGCAATCCACGATGAAGGGGCGTTTATCCACACGACAACAAGCTTCCAGCAAATGTTCA

TGCTCGGGTTCATGCAGCAAAAATAAGTAGCATTTTTCTGCATCATACTTGAATTTCTGGAGACACATTG

CCTTGCACTTTTGAAATAGGTTACCAAGGCAGATAAGGGCAGATAAGGAGTCTCCTCCTCCAGAGGGGTA

GATAAGGGAGGAGGGTGGCATTTCCTACAGAATAAACCACCCGGGGGAGCTCAAGCCATTGAAAGTGTGC

GGTCAGGTGCTAGAGGGCGGTATTCCGTTATCATTGATGAATGAACAGATATTTCTGGGGTTAAGGATTT

GGCTGTCGTGGTTAAATACTTCTGTGACTCGACAAACAAAACTGAGTCCAGGTTGCTCAATCTTGTACAT

GTGGAGGTGGCAACAGCAGAGGAATTAAGCCAAAAACTGGTAAATGAGCTAAAGAGTCACAATCTGGATA

TAAGTCATTGCATAGGGTTCTCTGCTGTTGCTGATACCTGCAATGTAATGATGGGATCAATCAGGAGGGC

TGCAGGGAGCTCAGGGGAAAATGTGGCATCAAAGCTGAAGGCACTTAATCCCCACATGATGATTATCAGG

TGTATATGTCATTCTGTGCACCTCTGTTCCAGCTATGCGTGTCAAAAACTACCACGACAGGTGGAACAGA

TGGTACATGACACATATAATCATTTTAATCATAGTTACCCGAAAAGGTCAACATTTGCAGAATTCCAGGA

GTTTTGCAAAGTTAAGCCATCTAAAATCCTGCTCCCTAGCCAAACCCAGTGGTTGTCCCTTCACCAATGT

GTCTGCAGAATTCTAGAACAGTGGGATGCATTAGGTTTGTATTTTAATTCATCATATTGGCAAGTTTTTT

TATAAATCCTGCATATGTACGCGCAACGGCAGCCCAGGACATCAACCCCCGAAACAGCAGTCAGCACTTA

CCCTTCCATCAGCAGTAACTGGGAAGTGCTGTTGCCATCTTAGTGTCAAAAATTAAGGACAAGCAAAGGT

TGCAGGATTTTTTTCTTTTCAGGTGCCAGCAATTCCTCATTGAAGTATCAGAACAAATACAAAACAGGAT

GCTGTGACCGATCACGTTTCAGTAAGACTGTACTCAACATACTTTTTTTTGGTATTGTGTGTACCATGTC

ATGTGACTTTTTCTAAATATTTAGAAGCACTGAAGAGGAAAGTAGTGAAAGAAGAGTCGGATGCTGAAAT

TGCTGGTTAGGAATGAAAAGGCAATTAGTCCATCGCGACGTCTACCTGTCAGAAGGCACAGCTTTCTACC

TTGTGACAGGTGTTTCGCAGTTATTGAAAAAATGAAACGCAAGAGGGAGAAGGTGGAGGTACCACAAGGT

TGGTATGGCATGGTTCCCAGAAGGGGTCACATCGCCCTCTCCTCATGCTGCCAACTCCTCCAGACCCCCA

GTGTTATTACATTTCTACGTGGTGGCTGTACACTCCTAGCCCTCTGCGAAGAGCACGTCCGCTCCATTGA

GTCGAAGGTGGGAGATGTGAAGGCAAATAAGAAGGAAGAAGCATGGCGGACGATATTCGAGACCTTCACC

GGCAAGCACGGACACAACCCTATCCGCACTTTGCGGCGGTTGAAGGAGCAGTACTGGCGCCTCAAAGGTC

AGGCCAAGAAGAATAATGTAAGAAGACGGGCGGTGGGCCTCCACCACCTGAATACTCGGAGGAGACGGCG

ATGTTGGTGGAGACTCTGCCATAAGGAGAGTTTGTAGTCCCAATGAAAACATGAGGTTCGTGTCATTTCG

TTCGTTTTGCATTTCAGATAAACGTGATGAAACTTACTCGGTGAACTTATGCGGGGTCTTGATAAATGGC

TGCCGCCACTTAGCTGGACATTAATTTTAATAACGTATTTCAAGTGCCACAGAAACCCCAAACACAACAC

TTTCAAATTAATTACAAACCATTAACTGTTGCATTAAATAGGTGCATGGCTCATTTCCTGAGAAATTATT

CTACACTTGCTATGCCACATAAGACAATGTAGGCTAATGTATGAGTCAGTTGTTGTCCGAGTGAAATTTT

CTGTGATAACAATGTTCATACTTAATTAATGCGAAGGTGTTTGAAATCCCTTCATTCAAAAATGCTCACA

AAACTTGGCATCTGGAAGCCATAATCACTTTTTTACTATGGTGGGAAATTTTTTGCAATAAATTCTCATG

TCTGCAAGTTACACCTGGGATAATTTAACCTTGGATGCGTCGAAGAGAGATAGGTTCAGAGTTGTGGAAT

ACGACAACCTAAGACCGGCCATTAGGAGCGGAATGCCCCACGAGGAATCAAAACCAAATTGAGACCCTCC

TGGCGATGGTAAAGCGAAACATGAATGTGCCCGAGATTGCGGACTTGATCAGCGAGAAGAGCCTTGCTAA

AAAGATGTGGGGAGAACTTTCTGGAGGGGATGGTCGAGCACAAAAGGCTACTAGAGTCCTTCTCGACCGA

CCTGGAGTTACAGATAGGAGTGAACTACCCGGATATCCTTGAATGGAGGGAGTCACAGGACAGATTCAAA

GCTGCCCTAGCCGCCAGTGATGAACCGATTTGGATGAGGGATCTGCAGTCCTATGAGTTCATGATTAAAA

CGAACGTCAAGCCAAGGTTGGATGGAACGGCAACGACAGAGTACGCTGCGCTCCAGACAATTGCATATCA

CGACAAGAGCATCAACTTGATATTTTGTCCCATCTTTCGCGAGATGAAGTTGAGGCTGAAAGCCGTCTTA

AAAGACAAGTTTCTGATAAACACCGACGTCTCACCAGAAGAGTTTGCCGAGGAGATCACGGCAAGATTTG

ATTGTGAGCGGCTGCAAAAGGCAGGCAAGCTGGAGATCGACGTGTGAAAATATGACAAGTCGCAGGGGGC

TCTTATCTTGGAGTTCGAGTGCCTGGCGATGGCGGCCTTTGGGGTGGAGCCGTGGTTGGTGGAGCTGTGG

CGTGGGTGACACGAATTCACTCGGCTTACGGACAGGAGAAATGGCGTCGGCGTGAGGGTCGCCCATCAGA

GGATAAGTGGGGACGCGTCGACCTACTTGGGAAATACGCTTGCCCTGATGGGGATGATCGCAACCCTTGT

GGATGTTGACTCTCTGGACTGCGGGGTTTTTTCCGGAGACGATTCGGTGCTCTTTGGGGAGCGACTGGAC

GCGGATTTGTCAGTGCTGTGCGCCTCCTTGTTCAATTTTGAGACTAAATTCCTCAGTACTTACAAGTATG

CTTATTTCTGCTCCAAATGTATTGTGGCGGTGGCCCAACCGGCAGAGAAAACGGAAGTGTAAAAATGAGA

GTGAACCGGGAACAACAGCCAAGGGGAAGTCAACAGGAGCCATAGCCTAGAGTAATGGTATGAATCCTGT

TGACTACCTACTGACCTGCAACCCGGAAAAGAAGGGAAAGCCACACGGTTTGGACCCACAAGGACCCCAA

ACCCAGAAAAATAGAAGAGAAACTAAAAGTATCAGGCACGATATAAGGAGCGAGAATCGGCCAGTTCGAG

TCGAAGAGTCGAACGTCGAGCGTCGGGTGTCTGAGAGGCGAGCGGCGAACAGCGAACGTGAAGTCGAACG

TCGAACGTCGAGCGTCGGATGTCGGAAGTTGAGCGTGCAAAGTCGAGCGTGCAACGTCGGAGGAACGGTC

CGGACAAGTCCAGAGGCACGGGAGAAGAGTCCCGAAGATATCCCTGCATCCCGAGAGACGCCGGCGCCTG

TACCAGCATCGTGAGTATGGGAATGTCAGTTCGGTGTACGCGTCACCAGTGAAGTCCTGGTCCCATGTCC

GGCCATAAGCAAGGGTGGTCATCCTATGAACAGTGTCTGTAATGCTTATAACCATTATGTCATGTCGTTC

AATAAATGTACACAAAAATATTACTTTATTTATGACAGCTGGATTTTCAGAAAATAGAAGTCATGGTACC

AGTGGCGCCATATTATTCCGGACACTTTTTATACTTAAAAAAGTGTCCAGAATAATATGGCGCCCATAGT

ACATTTCAATTACAAAGTCTGCTGTCCTGTGGACTTCATTGTATATTACTGTTATCGCCATTTTCCATCA

TGCCCATGTGGCACAGTGGTAATACCATCACTTCTCGTGTGTTTTTGTGTAGATTAAAGCATGTCGCGTA

TCAAGTTACTTGAGCTTTGACCAGTCGGTACCCGCCGCTACCTCTCAGCAGGAGTTCGGATGGGGAAGCT

GAATTTGAAGTGAGACAGTTATGTGCAATAAAATATCGGTATACCCGCGGTCAGGCCTGGTCAAATTAGG

CGGTCGAGATCTAGCAGTGACATTTGAAAGATTAGGGTACTTTCTTAACGACTTATAACAGTTTTTCCGG

GCGATCGCCGTGGAAGCCCGTCAAGCATGCGCAGTATTTCGAAGCGGTACAGGGTGTCCAGATCCAACGC

TTCGCCACAGCCCTTATGTGTGCCACTTTTAGGTTTTGCACGTTGTGACCACTCGCTAAAATTTCCACAG

TTGGGGCCTTTTCAAGACGCCTCCACTCCTTACAGGTGTTTCCAAGTCCATTTAGACACATAATAGTCCC

AAGTTTGTATGTTTCAGAAGACCAAAGAATACCACACAACAGATTTGATCTCACTCTTTGATGTGCCAAT

GCAATGGGAACTGGGCTATGGGACCAGTTAGGTCCTTAAAGCGTCCAGAAAAAACCAAATAGCTGGGCAG

GGTTGGGGTGCTCCATAGCAAATCCATTGGCCCGAGGTCCATGCACCTGTGCTCTCCTCCACTGACTTTC

AGTACTTGCAGCTCATTAAAATGTATGCTTTGGTGACACCTTGGACTTTGCAAAATTCACGTTCGATGCC

AATGGGCCAGGTCTATAGTGGGATTCCCTTGCACCTCCTTCAGGGCAAATGTCACTGGCTCCAACTCCTT

TCGAACCCGGTGCCTGTGTGAGATGGATTTAGAGGTGGGTTGGATTATTGGGTGTGTGAATCCAGGTCTG

GGGCAACTGACATCAAGGGACCGGACTGTAATTGTAACACCTTAGAGGCCATCCTACCAACCAACCACCA

CGTCGCGACTTCCAGGGAATTCAAACACATCGCAAGAATGGTTTAAAGCAGCTTCTGCCATTCATTGAGC

CCAAAGTTTTGTGCCAAGCACTCAAGGCCCCAGNCCCGGATTTGATCCCAGGACCTCCCGAGCGTCATGG

TCGATGGTCGAATTTGAGTCGTGGTTTTGTCCCCTTGCTTCATGAGCTTTGTGACATCCATAATAGTTCT

AGGACACATGGGTACTTCCAAGCCTTTCCTTTATGGGGGCCAGTTTTTGTGGTCTATTTTAGTGGTTGCG

GATGGCTTTTACATCCCTATGGATCACCCATGCATCGAATGCCAACCGGTATGCATTTCCAGGACATTGT

GACCCCTCCCTGGAACCATGCCCCATGTTTTTTCAGCAATGCCCTTTGAGTCCCATGTGCCTGTATATAA

CTCGTGACTTCTTTCTCCTTGGCATCACATCAGGTGGCACCCAGCACCCAGCAACCACTCGACTGAAAAG

TGTAGGCTACACTGGAGTACCAAAATTCAAGGAGTACCTGGGAGTACCACTTGTACACCTTAGTCAGATA

TTAACGGGGTTATTCCAGACCATGTAGGTCCTTAAACCCCGGGCCCCACATAGGGCCTATATCATCACTC

AAAGTCCCTTTCATCGTGGTCCGCACAGGGGCGGACCACCCGTTCGCTTCCGAATCGAACCCAAGCCGTC

ATAGGGTCGAGACTCGACCATCGGGACG

>GAUE02021637.1 TSA: Anurida maritima s7824_L_19464_0_a_69_1_l_9420 transcribed RNA sequence/Negevirus

TTTTAACTTTCACTTCCATTAAAAGCAAATTATAAACTCTCTCACGAATTAAATAATTCATGAACACAAC

TCAAAACTTCGACCTCGGTAGCCCCAATGGGGCTGTTGGGGTGGATGAGTTGGGGGATGTAACGCATCCA

GCCGATTTTTCCGACCCCGAAAACACCTTTGAGGCTAACGCAGCGCGTCAGTCTCATGTGACCAGCTTGG

TTGCCAAACTAACTGGTCGTTCCCTTGAAGAGTACGCGAACATTGTTCTGCAGAAAAGTCTTGAAACCAA

CGACGATCTAAAGAACTCTTTTTACACACATGTACAAAATGAAGTTCGCCGTGCTCTTGTACGCCCTAAG

GACAACCCAACTGTCATCATTCCGCAGGTCCTTACTGTCTCTGAGTACACAACTCTCATTGACTCCTTCC

CTGAACTTTCATTAAAGTTCACCAACAGCAACGCTGAAGGTCATGGTTTCGCGCACGCTCACAGAATCTG

TGAAACTGAATTCTTACTCAGACTTGCTCGTTACCACCCAGAAAGAATCACCAACCCAGGAACTTTCCAA

GTCAAAGACATTGGAGGTAACTGGTTGTACCATCTCGCGCACAACCGAGCTTCAGTACACAGTTGCTGCC

CAAACATGGACGCACGAGACGACGCACGAAAAACTGATCGCATACTTTCAATGGAACTAATGCGAAAGAA

CCCGTTACAAGAGCAAATTGTAAAGAATCTCTCTGAATTTACTTGTGACGAGGCAGGACAAAATTGTTCT

GTTAAGGCTCCGATCCTTATTTTTGTGCAATCAGTCTATGACATGACCCTTGAGAACATGGCCGACTCAA

TGTATCGCTCTTCTGCTCTTGTGGGTTATGGTTCATTCGTGTTTGCTCCAGAAGTGGTCTACAAATTGGA

TGGCCAAATCCCAGACATCAAGATGAATTTCAAACGCATCAAGCGATGGTTTCGAGAATACATCAGATTT

TCGTTCGATGATTCGTCGTTCATGTATGAACATAGGTATGACACCTACCTTTCGAAGATTGAGGCTTCTT

TCATAACAACATCATGTAAAAGTCAACATTATCTTTACGAGCTCATCGAACGTCGTAATGGAGTCCAATT

TTTCAAGATCACCAGGTTGAGTAACCCTAACATCCATCGTACTCGACTTGCCCATCACGTTCCCGTCCCC

GAACTCAAGAACTCTGTCATCATCAAAACCTGGCGTTGGAGTTATTCACATGTTGGAGCCGGTCCTGCCA

TCCATATGCGTCCAGTTACTCTTGTCGTACCTTCCGAACTATTTGGTTTGGTGTACAGTTACATCATTTC

AATGGGTGACGGACGCTTTACAGCCGGCAATGCAGTGATCTATGCTAACTCAATGAACAAACGTGTCATA

GTCAACGGAACCTCTGTTGCCTCGCCTAATTCAATTCCACCTGAGGACTGTTATCAAATGGCTTTGGCAA

TCTATCTTCTTTGTTATCGTGAGAGACATTCAACCAGTCTTATGATGAAGACGTTTATGGAAGAAGAAGA

AAGAATTCGGAAGCAGTCAACACAATCAGGCTTCCAAAGGTTCCTTGCAACTATCGCTCGTTGGTTTTAT

GGAGGTTATGCAAAACCAAAACCACACTCAAACAAACCAACGCAAATGCATGAAGATTGGCAAAGGGGAG

TCGATTGTTATGATGGTGAGGTCCATGCAATCTTTGCACTCAGCCATCGTTTCAACATTAGTGTTGAGAA

GTTCTGTTCTAGAATTGAGTTTTCTCAACTTCTAAAAGAGACTCTCCAAAGTGCTAAATGCGCTCCCGAA

ACACACTCTGACTTTCTTCAATGCATTGGTCCTGACGTCGATTTGACTAAAGAAACACTTGAATCATTCC

TAGGTGGTGAAAAACCATTCGCTCTTGGAATCGATGAATCAGTTGAATCTTTCTCTGTCGACTCTTCCGA

ACTGTTTGAACCAAAGAAGGGACCTAGAACGTTTCCAACTTCTCCTCCGACAGAAAGACCAAAGTCAAGT

CTCAGCGAAACACCATCTGAAGTACCACCAGCATCTGAGAAATCTTTCTATGATGACGCAAAATCTGTTC

CTGAAGACGAACCCAAAAACAATTGTTTCGATCCTTTCAGGAAGCACAAAAGGATGTTCATTAAATCCAT

CAATAATGAATGTCTTTATGCCGCGATTGCTGCCGCATGTTGCCCATCCGTCTCTGCATCTGAGTTAAAA

CAACAACTCTTGATCAGTCCCGCTTTGTTTACTTTCGAAGAAGTATTTCAAAAACATATTCGGGAAATGC

TGATGGGCAAGACTGTGATGGGAGATTACCAAATAATCGTCCTAGCTTCGCGACATTTCCAACACAATTT

TTGCGTTCACATTGATAGAGTCAATTACCACCATGTCTTTGCCGCTGCCTCACCTGATGCACCTGTTTCC

CATTTATGGTACTCAGGTGAACATTACGATCTAATGGAACTTGATTCAGATGAACCTGAAAGAATCTATT

ACAAAAACGTCAACTTCGTACCAACAGAAGAAATTCAGGATATTAAGTTTGGTGAAGTTCGTAAGAATCT

ATCCAGGGTTCTTAAGGCATTCTCCAAACTTACCAGAAGAGAAAAGACGGATTTGCAAAAGAAGTTCTTT

AGGAGTAGGCATTTCGCTAAACAAAGCGACCAAGTCATTTCGGATGCTGTGAAACGCTTTAATCTTGACA

AACTACCACCTAACACTGAGTTTGACCACATCGCAAAACTGTTTCGGTTGTCTAGAAAAGGGATTGCATA

TCAGACACTTGAAACTGACACTGACGTACTCTACTACTGTAAATTCACAATTGACCTCGATTTCCTTGAG

ATTTTGAGACATTTTGTTTTAAATTTCAACTCAGTACAGTTGTTCGTTAGTGGCAATGATAGTATTCTGT

CCAACCATGTTTACATCTATGCATCTCTACCCAGAGGAAATCTTTCCGGACTCTCACAGGAACTTGCACA

GGTTGTTTTTGACATTCTTAACACTCTTGTCATTTTCTTTGAGAATAGAATTCTCTTCTATCTTCCTCAT

CTTTGCGATGTTGAAGTTAGTGACGGCGAGATTAGTGAAGACTCTTTTCATACTACATCTGACAACGAAG

ATGATAGAGACGAAACTAAACCTCCGCAACAACCATCACAACCGCAACCGGAACCTCAACAAAAACAAAA

TTTCGGTGAGACAAACGACAAACAAAACAAGGAACCACCAAAAGAGGATAAGGACTCAAACAAACAAACA

CCCAAGGACGAAAAGGGTTCAAATAAACAACGTGAACCCGAAGAAAAACCTAAACCTGAAAGTCCTAAAC

AACCAGAACCTGAACAAAAACCAAACCCTGGACCTCAAAAACCGGACACACCGAAGAATGACAACAAAAG

AGACCATTGCAAGGATATCCCTAGACCTAAGACTAAGCCTAAATTCGACTGGCCACAACCAATCGGACTT

GTGCGCAACAAAGACCCAAGGGTTTGGATGCGTGAAAGCACGATTGTTGACTTCCATCTGGTCTACGAAT

TCAGGAACGAAACTAACCGCTATGACATTCTTGACATTGCAAGGATTGAATACGACAGCATCGTCGTTGC

AATGACCAAAAAGACAACATTGAGGAATGTCTTGGAAGTTTTGGCATTTATCCCTGACTCTGGTTATCTT

ATTCACATCGACAATTCCTTCTTCAACAGACCTGTCAGATTTTCTCCCGATGATCTTGTCAACAGTCTGA

GGTATGTGAAAAAGAAAAACAGTTTTGTGTTTATTCGTCCTGACCAAAAGGTCTCAACACTCTTGACAAA

CAAAGAGTGCTTTAAGACTAACGTCAATCTTCCAACTGACGATCGATACGATCCTGTTTATCTTCCTGAA

GACGGAACTGGTAACACCTACGCAAGAAATGCATTGATCGAGATTGTTGAAACTTGGCGCGTCACACATG

CTGCGAATCTTCGTAGTCACGCAGAATTCTACAATTTCGTAGCCAACGCATCTAGACACGAATTGATCAA

GAAGTGTACAAAGGCAAAATTCGCTGTTCTTGATGAACATCTCAGAACCACAGTGAAGTACAACGCAATG

CCTGAAGAATTTTCGTATGTTTATTGTGATGGAGGTTTCGTTAAAACCAAACTCACTTATAATCATGCAA

CGAAGGCTTTTGACACATCTGAGACCCTTCCTCATCGAAAGTTCTACCTTCTTTCTCAGTATTGTGTTAT

GCAACTTGAAAACGAGTTGTATAAGAGATTCGCACATTATGAAGTCGCAACACTCAGACTTCCTAAGTTC

GAACTTGTGCAGGGTGTTGCTGGTTGTGGAAAAACACACCACATCGTGACTAACCATAGACCACCAACTC

ACATCTCTAGGGGCGAAATAATCCTTTTACCAACACGTGCCTCTTGTGCTGAAGTAACTCAAAGAGTCCA

AGCTGTTGCTGGTTCAAATGTACCTGTGACAAATGTCATGACAATTCATTCTTACATGCTCAACCACACA

GCTAAGGACTATGAACACACAAACTTGTGGATTGATGAAGCTGTTAAACAACATCCAGGAACTATCGTTG

CTGCTGCTTTGATCAGTGGTTGCACGACAGTTAAATGCTTGGGTGACAAACATCAACTCAACTTTATCAA

TCGCAATCCAACTTTAGCAACTAGGTACGATCGTGTCCCTGAAATTTGTATTATGGCTGAACTGCACATG

TTATCTAGGCGTATCCCAACTGACGCCGCTTTTGCATGTTCCCCATTTTACGTTCAGGACAAGGAAATTG

GAAAAATTTGTGAGACGTTAAACTCAAAATTAAGATCTTTTGAGTTGAATCAAATCACTAACGTCACACA

AGTCCCTAGAATTGATTGTACTAAGGGTGATACTGTTTACTTGACCTTCACGCAAGCTGAGAAGGAAGAA

GTTTCAGTAGCAATCCCTAACAGTGTTGTCCACACGGTTGATGAGTTTCAGGGTGGTGAAAGAAAGAACG

TTATTGTCGTTCGCACGAACAAAATCAGAGACAACCCATTGTTCTCAGATGCGAAACAAGTCAATGTCGC

TCTAACTCGCCACCGCAACACCATGAGATATTTCACCGTGTACTCTGACGATACATTGTCACGTATAATC

TACAACACCATGAACTCGCCGACATCTGCGATCAGAAAACATCTTCGCACAAAAGGAGGACTGCACACAG

AATACCACCAAATCGTGGGCACGTCCCATCCTTCACAACAGATTACGTTCACGAATAAAACTGACAGTGA

GGGTATCTCGATTGTGAAAAGAACAAACAAACCACAGGGTTTCTTACCATCTGGTGACTACAATACGGGA

AAAACATTCAATTCCGATAAGATGGGACGTGACTTCGGGAAGTTCGTTAGACTCAACAAGATCGACGAAA

ACGTTATTCCTATGCGTAAATTAATTTATACGCTAAAGAAGTCAGATACAAAGGCTGTCATCACATCTAA

ATTCTTGTCATACGACGAAACTGTCCAAAAGACTTTGTCTAATGTTCAAGATTTTATGACAGTCAATGGA

CTCTTAGACACTCCGTGTCTCAGAGTAGTTGAAGATCCCGAATTCGAGGATGACTTCTTTGTCCCACCAC

ACATAGCACCACCTCCATTTGTTTCTGATCCGATTACAACCATCAGACATTGGTATGACACAATTAGGCC

AAATTTGACACCAAATAACACTTTTGACACTCATACCGTCCACCATCGCGATCTTGATTTATTTATCCAC

GACACTAAGTTCGATGTTTCCAAGTTCTCTTGGGAAGTTCCTACTTACGACAACATGCGTCCTGTCCTTA

TGACAGATATGCCCTCATTGCGACCAACATCAGGAATCGAGAGTATATTGGCTGCGAACAAACGCAACCT

TCATGCACCGTCATTGCGTGAACCTGGACCAACCGAGTTTCACGCTAAGTCTTGTGTTGATAAATTTGTC

GAAACATATATCGCAAAGGACAAGAAGTGGTTGTTAGAAGAGTTCAAGAAAAACCCGATCATGCCGAACG

TGACATCTTTGCTTGAATGGTTACAGACTCAACCGACAGAGGTAGCTGATATGATTGTCCCAGACGTGCC

TTTATCCGATGATGCTCTCAACGAGTACTCATTTGCGATCAAGAGAACTCCTAAGCCCAAACTTGAGTTA

AAAGGAGTAAATGAATATCTTGCACTTCAGACCATCGTTCATCATCCTAAACATATCAATGCTTTCTTCT

GCACGGTTTACCGTGAGATAAAACACAGGCTGCAGTCTGTATTGGACCCGAAATTTAACATCTTCACCGA

TTGTTCCCCAAAGGACTTTGCTAAGAGAATCAACAAGAAATTCCGACCTTCTAGACTTAAGAACTCAACT

AAATATAAGGTCGAATTTGATGTCTCTAAGTACGACAAGTCGAATGAAGAGTTACATCTCAACATCGATG

TTCTCTTGCAAGAACTTTTTGGGGTTCCAGAATGGATTCGTACGCTTTGGTACTTCTCACACACTGTCAC

CGTTGTGAGAGATACGAAGAACAAGATCAGATTCGTAATCAAGTTCCAACGTAAGTCAGGTGATGCATCC

ACTTTCCTTGGGAATACTTTAATTCTCATGGTTTTTGTTTGTGTTACAGTTGATATGGCGTTTGTTATCT

TTGCAATGTTTGCTGGAGACGATTCATTAATATTCTTCTCATGTGCTGGCAAGTTCTACAACATGGACTT

TGCTTCCTATTTCTTCAATTTTGAAGTCAAGAATTTTGACTATAAGTATTCGTACTTTTGCTCTAAGTTC

TTGATTGAGGTTAACGACTGTTGGTACTTTGTACCCGACCCTCTCAAATTTGTTACGAAACTTGGAAGAG

CAGACCTTGCCAATCCAGATCATGTCCATGAATATTTCGTTTCCTGCTCTGACTTGATGACTGACTTCCA

TGACGCAACAATTTATCCCGCACTCTCTGAGGCAGTCGCTGAGCGTTACAACACTCATTACGCTTCTGAT

GCGACTTTTGCTTCAATTTCTTCGCTTGTTTCTGATGAGGCACTGTTTAGTGAGTTGTACTACTCTCTTC

CCGGTGATTATCATTCGACTGCGAAGGACTTGGAGCTAAGGGATATTTAGTTTCTCGACATTACAATGAC

TTACTACGTTCCATCAAACTTTTCAGATTTGGAGATTTGCGAATTTGAACCTCTTTCATTTTCGCTTCCT

CTTCTTCTGTTCATTATCAACATCCCCACTCTCGCTACATCAAAACCGAAACAGACACTACAGACATCTC

CTTTCGATGACAACTCACATTTCGAACTCTCTAGGTACACTGACTCTGATCCCAGCTTCGTCCTCACAGA

ACAATGCCTTGACATCTACAACACCGGAGTCTTCAGTCCCACTGATTGCAGACTCTCTTATTTCTGTCGA

GAACCACAAAAGAAGGTTCTCAAATCCGGATTTCTTGGACTTGGCTCTGAACATATTCTCTGCTACTCTT

TCACTCCTATCCCTTTACCTCCTCTTTACCGCGGACTTACAATCTATAATATCGACAATCGCATTAATCA

CGAATTCTATCCTACTATTCTTCGTCCAACTTCTAACTTTGTCCTCGACCCTGACTCGTATTGTAAATTA

CTATCATATAGCTTCTTTTACCGTGACGGAACTTATTACTTACTTCACCCACATTGTAGACGTTACCTTG

ACTCCGTTAATACGACTGCTTTTCACGTTGACGATAAAGAAAAATTCTGTATTCCGCCAATTTATAGACA

CCAGTGTAAACCGATTTATTCTTCAAAACATTTCTACGAAATTATCTTCTATCTTAATCTTACTTATATT

TGTGACTCTTATGAATTCGACTTAGACAAATGTAAATCTGACTATGACCTACCTAATGACTGCCGCGTTC

CTAATCAACGCTTTCAAAATTTCTATTCCTATATTATGCGATCCATTATCACCTTTGTAATTAAGGAGGT

CATGTATTTCATCGAAGACATTTTCATCTTCTTCGAGGAAGTACTTATTGACCTATTACCGACAATCATT

GACGTTTTACTTCGCACTCTCTCAGTTCTTTTAGATTTAATCATCAACGCTCATCTGGTTTCTTTACTTA

TTTCCTTTCTCTGTTTCTATATCTACACTACTAAGGCAATCCTTTCACTCATTCTTGCAGCTTGTTTCTT

TCTCATTTACACAATTTATAATTAGTTTCTGAACATTATCATGCATCCAATTCCATATTTCGTCATTTCT

TTCTCCTGTTTTCTGTTCCAGCTCAATTGTTCTTCAATTCTTGACCAGCCCAAACTTCAGAATGAAGTTC

TTTCGACTCCTCTGCCTCCTGTCCATCACATCTCTGCTCGCGGTGAAGTCGAAAACTCCACACGCATTCA

CCCGTTTATTGGACTTGACATTCGACCGGACCGTCTCTTACTGTGACTTGACGATGACTCGACCTTACAC

GATTCTTGGCTGCTTCACTTCTTCATGCTGCTCACCCTTATTTAGATCCTTTACTGTTAGTCAATTAGTT

CACTACCAATCCTCCCGACACTCACTGGCTTACAACGTTACTTCCGTTAATTTAGTTTAGTTTAGTTTCT

CCACATTTCTTGTTACAATCTGCACCATGCCTTCCGCTCCAATTCTCGTCACTCCCGTTCCAAAACCACG

CGCTGTGTCCAAGAATGGACCAACTCTCCTCGTCGCCCGTAACAAGACCGCGCCGCCTAAGAAGGCCAAG

GCGCAAAAACAAAAACAAAAGGGAGGTAAACAGCAGTCGAAAGACCTCGAGGGCAAGATCACTGGACTTT

TGAAGTCACTTTTCAATGATCTTGCTTTCGTCATTGCTTTGGCGATAGCTGTTTACTTCGCTACAACTTA

CGTCTCCACCAAGGATGACAACCCCGTTCTGAAACTCGCCGACCGACTTATTGCAAACGAACGCTTTAAA

ACCGTTGGTGAGTGGATCAAAACGAACGCGATGCGTTTCGTCGGATTATGTATCTTTGCCCCGTTTGTAT

TCTCAATGAAGAGGGACCGCTTGTATGCTCTTGCCGGATCGATAATTTTTGTCGTTTTCATCCCGGAGTA

TACTGTGTGGGAATACGTCGCAATGGCTGTTTTGCTCACTCTCTACATGAGAGCAGAGTTTCGGCAATAC

CGTATTCCAATCATTGGCATATCTTTCTTTCTTTACTATGCAGGGTATTTTATACCCACTTCGACAACTG

CTGCTCCAAGCGCAACGTCGTAAATTTTTTCGCTAAGCATATTTTTCTTTAATTCCATTTTCAATGCTAT

TGGTTAAACAACATTAAATTTTCTTTCCTTTCAATTTTAA

>GATZ02022882.1 TSA: Sminthurus viridis s11732_L_33683_0_a_50_8_l_10627 transcribed RNA sequence/Negevirus

AAGACATGAATAAATCAAATTTAGAAGAAAAAGAAACTTCTCACCGCTGAAATAACGGTTACAATAAAGT

GTATTGAGGGGGAATTACGAAATAGTATTTATACTGCTTACCACTACATGTGGCACTCCACGAAGCGGCT

AGCAAATTGCCATCGAAAAAGAATTCTAATGGACTTTTAAAATTAAACACAAATTCATCCTGGAAAATTT

TTGACTCCCGCAAAGGAGAAAAGGACAAAAGCAGAATAAAGTCATGGGGTGTTTTTGCAAATTTGGAATA

AATAACGGCAAAAGAAGACAAAGTAACACCATTGAGAGTGTATGTAAACGTATAGTAGTCACAGTTAGAT

AAAACATGTGTGCCAGTATAATCATACCTGCGTGCATATTGACTAACACGAACAAAACCATAAGATTGAT

ACAAACCCTCATGCATTCCCTCAGACCTCGCCTTCTGATACAAATTTAGACCCATCATGTGAAGATTTGC

ACTGGTGAACGGTGTATACGCCAAATTAATGTAATTGTTGGGAGAGGATATAGAGTGACAATGAATAATG

TTATCTGTGACTTTGTACCACAGACAACTAGAAAATTCCTTTGTGGACGAAAAAGTTGCTTCCTGAACAT

TTTGAGAAACTAATAATTTTGTGCCGGACCGAATCTTAGCTGTGATATTTAACGCCCGTTTTTTGCGTGG

TTTAATTGTGGAAAAAATCGAAATTTGATGGGGCGATAAATGAGCCTGATCAGAAATTAAAACGTCGCCG

TAGTCCGCTGATTCCGTGGTAGAGTGCCCTACCGCGTAACCCTACCCAACAGAAGATGCAGTGGTCGACT

GTACTGTGGACGAGTGAATAGCCTTCAACCCTTCGAAAGTGGGCAAATGAACCACCCCCGACACGAGCAT

AATCACCCCCACAAAGATAACCATCATCTTCTCAGTTCGATCTTGAACCCTTATGAACAAATAGCCCAAG

GCACTGATGATGGCGTACTGATACAACGTCATCGCCGGCACCAAAAAGATGAAAAATGAAGTACCAGCAA

TCACGAAAGGATACGTACGCACCGCATAAAAGAATGGTAACATAATCACCAAACCAATGAAGTTCTGTTC

GTGTGCCAAGAGCCATTTTATAATTGGATTCAAGTCGGAAACGTCACCAGCCAACATATTGACTGTAGTC

AACAAAATCGACTTACCATTCATGAACGCGACAGAAGTGTACAAAGCAAAGAAAGTAGAGGCCAACATCG

CAGGATTGGCCACAACAGCAGAAAGAGCGGAAAAAATCCCTGCACCCGCCGGCACCACGTTAAGACGCCG

ACGACGAGGCAAAGGACCACGCCTACGAGTATAACCAAACCGATCCATAACGAATGAAAAGTGAAACAAA

CGTTTTAGAAGAGAGACAATACACTATGTTACAGTTGTAACGCTATCAGAAAATTTTTTAAAAGAGTAAT

TTGCAACAACCAAATAAGCGACATTCATGATAGCGACTATGACAAGATCACGATACTGAAAGTAAACAGC

AACAGAAAAGCAAAGCATAATAACGGCATTGGGAAAAGACATAACAACATCATATATAATCTGCACCATC

AAGACAACAAGCTTACGAATAAAAACATTCAAATAAGGCAAAACGACCATCAATTCCTGTTTGATCAAAT

TAACATACGCCACCAAAACATCATAAAGAACATTCATTATCTCACGAAAGAAGTAACCGTAATACGTGAC

ATAAATATGCTTCGCGTGCACTGTGACATTGTGAACAACTTCACCAAACTCAACGTTACGAAAATTAAAA

GATTCACAGTCACGTTTGCGAATTTCAGCAGACACACACGAATCAGAAAAATAATTCTTACAAAATCTGG

TGGAATTGTGCACACAAGAACTATCATCAACATTGTAACTTACAATAGGCAACATTTTAATTAATTCATT

CAAACTGTCATACCATTGAAAACGGCACAAAGTCTTCCTAATCAGCGGATCAAAAACGGAGTAATGGACA

GAATCATCTTTAAGGAAAGGTTTAATGAAATCAGGATAAACATAATCACCATTGCGATCAAGAAATACAG

AATGTTTATAGCACAGATAAGGTAGAAGTGCATACTTGCCGTCTTTATAAAGGTAGTAATATTTGAAATG

TACAAAGGAGTTATATACCTCTCCATCATTAATTCCGTCATAAAAAGTAGGACGTTCATAGTAGTATCTG

TCATCAAATCCACCAGACATGGGAAGAGACATAGAACGCAACTCAGGTAGGGACACATCTCCAGTGAAAC

AATTGTCACTTGTGCAATGCATAACCACATGACCCAATCCAAGACATAATACCACAGACGATCCATACCA

TTTATGAGAAACCAGAAAATGGTGGGGCGTTCCGCATGGACCAGGTAAAGAACAATCAGAAGGCAATAAA

GGTGGAACGGACTGAACGGTAACACATTGGGGTGAAACAGACTGAACGATCGGTACATAATAAAGAGACA

GATTTTTAAAAGTAAAATCCATGAAGAACTGTTCATATTCATACTGATAGCCGGGTGTGAATAGTTCAGA

ATTCGACCGCGCGACTAAACAAATGGTACAACCAACGCTCAAAAGAAACCACATATCCACAGAAAATTGA

CCAATCGTTTCTCATGCACTCTTCTGAACAAATCAAAAAAGGGGAACAAAGAACAATAACGCACAAAATA

AACAAAATAAAACCAACACAAGACATAATAACCTAACAAATGAATGGTTGTTCTCGTACTCCACAGACAA

ACGAGAATAAACAGGTGTTTCGTTAAAAATGGGAATGTCAACGTTCGAATCAAATTTGCACAAATCGGTT

TCAAGATACTGTTCAGAAAGAATGTTATTAAAATAAACCCGTGCTAACTGAGGAAAATCAGTGGGAAAAG

CCATGAAAGAAGGCGAGCGTATAATAAAAAACTGTGCAATTGTACTAAATTTCAAAAAAAGTCCCCGTGG

CATGTGGATCCAATTCGTCACCTTCACGCAAATAAAACAAGGAGGCAAACTTGTCAGCATCCCCAACGAA

ATCCAATAATGCACGAATAGCATTAATAACCATACCTTTGCAACCATAACGTTCCATGAAAGCATCAGCG

TAAACTTCTTGCACAGATTCATCACACAACGGCATTAGCAAATCACTACAACTCCTCCGATACTCTTCAA

CATGTTGCCAATTTTTTAGATCAGACCTTCCCAACTTCGTTACAAACTTGAGGATGTCTGGGACGAAATG

ATATTCACCATCTACATCAAGCAGAAATTTGGAGCAGAAATATTGATTCCTATAATTATAAATTTTCATT

TCCAAATTAAAAAGCAGCGCACAATCAAATGACCTATCTAAACGTTGCACATAAATACTTTCAAACAACA

CAACATTATCATCACCAGTGAAGTACATAAATATAGCAAGTGACATATTATAGAGAAATGCAAAAACACA

CATCACAAACAAAGTGTTCCCAAACCAAGTCGAAGGATCACCACTTTTACGTTGTATGGCTATCATCACC

CATATCCCATGGATAGGATCGTACACAAAAGTGTAAATGTGTGAATTATACCACACATGCACCAAAAACA

ACGGCATTCCCAGATAATAGAACATAAAACACTCAGTCACCAACATTACTTCATCCTGAGATTTATCATA

TTTCCCAATATCATTCTCCAACGGAATCCCGAAACGAATCTTCTGCCTCGCTCCACGAACGTTATTCAAG

TACCTTGTGATTCTTTTGCAAAACTCCTTTGGTGACAACGCCTGGTATAGTTGCACATTATGTTTCAAAA

CAGCTTTCAAACGCAAATTCATTTCGTTGATGACCGGTCCAAACAAAGCGTTCACATTCTTCTTCGAAAA

AACTATCGTCTGCAACGCCGCATATTCAAACGATCCACTCCCATCCAGCTTCGGCTTCGGAATACGTTTG

ATGGCCAACGAAAACCGATTCATGTCAGTCTCCCATATAGAGTATCCATCGTCAACAATCTGGTCAGGTG

TCAAAGTTTTTTGTTGATCAATCCAATCCAAAATCAACTTCTTGTTGAAAGGAATCGGATATTGCCGGTA

GCTCTCATACGTAACCTTATCATTGATGTAACTTTCAACAAAAGCAGCATGTAGTTCCTTAGCCTTGGCA

GTATCATCATTGATATACTTCAAATCCGGTGTGTTGAAATTCCGCTTTACCAATGCCAACAACGTTTCAT

AGTATGTAGTGTCACGCGCTTTCGGCATTGAAGTGCTCAATTTTGGAGTCAACGTATCAAATTTTTTCTG

AAATACATAATCCGTGTTATTACTAGACAAACGTGCGTCCACCGAAACATTCACATCCAAATCGCTAGTG

TGCACGTGATATGCATCGTAATCATAATTAATGTGTCCGTCATTCGGTAGATATTGATCAAAAAATATCT

GCAAGACCCCTTCATCATCACACTTCCCGTAGTCATGCATTAAATCACAATCGTCAACTTCCGGCGTAGT

CTCCACATTCAAACTTACTGGTCTATACAATGATTGTTCACTCACACCTACAGATTCAAGAAATGGTTCA

ACATACTGATTTAGCTTAACAGATTCATCCAAACACGCCCCGACTAATGGATCGTAAATATGAATTTTAT

TCTTTCCGGACAATTCAGAACAAGATTTCACCAATTTATCAACACACATAATACCCCTACGCACCACAGA

CGCATCAATGCACATGTCACCTTCCGTGATCTTGCTACGTAGAAAAGCCCTGAAACAATACTCCGCACCA

AGACCCACATTCTTCTTCAAGATTATACGTTTGTTGTAACCCTTATCTTCCAGGGAATATCTTGGCATGT

ATGCAGGAGCATACGACAATTTAGATGTGTGCAGTACACATTTCAGAGTCCTCGATGGCATGACAGGTTC

TTGATACACCATAATATTATCAAACACATCCGCTTCCTTTGCAACTTGCAAGTCCTCACGCACCGGGAAC

CACATTTCACGATATGCAGGATATACACCACCACTCCTGTTAAAATAATGCTTATCCAGTTCAAAATCTG

ACACATCAATCTTAAAATATTTCTGCAATTCATCGTCAAACAAATTCTTATGGAAATACCACAAATCCTG

ACTACAACGCGACACCGCCACCCAAATGTAATGCGGATCATCGAACGGATATTTACATGATGCCTGCGTA

TTAAGACGCACTATTGCAACCCTCTTCGCTTCATTGCCCTGAAACCTGTGTACTGTTGTGACTTTATCTG

CTGGAAATCCGGGGACCGCTTTCAACATTTGCTGTTCGACGTTTGTGAATGTCAAATACACATCAAAATC

TCGTGGGACATCAATTTCGCTCGAAATCTTGACACTGTGTTCTGCTGTGTGCGATCGACGTACTTTAGAT

GCAGTCATCAATTCCTTACCAAAATGGTGCATGTAACCTTTCCCTTTGAAGAGTTTTTCAACTTTCACAG

CTTCGATCTGTGCAATACGTCGCGACACATTCATAAAGTCTTCGACCTGCGCGAACTTATTCAAATCCCC

GTACTTCATCTCATGATCTGGCAGGTTGTTGACACAAGGTATCTGCAAACTATCACCACAAAAAGTGACG

ACATTAGCTTTGGCTAAATGTGCTGCCAAAACATAAGCGCCAGGATGTTGCATCATCGCTTCATCAACAA

GTATGTTTTCATACATCCTGTCTCCATTTTTCCTCTTTCGCAGAAAACCAGCGGCCGTCATCACGTACGT

TATGTCACGTTTCAACAACGATCGATGAATAGCAATCTTTTCCTGTATGTCATCCGCGTTCGCCGATGTT

GTCGTCAAAATCAACGTATTATCCGGGTCGTGTTCATGAACAATCCTGTATGTTTTTCCCGCACCTGGTG

CGGCTTCACGCAACGTGAGGTTGATCGCCCACTTAAACTCTATGCTGTGCGCTTTGTATTTTTCATACAA

CATTGCGTCAGTGCATCCTTCTGTGGAGTACGTGTTCATCACACAAAATGTATGACTGTCAACGTGTTCC

TGAATTCCATCGCATGAATAGCACTTCAACATCTGACGATAATCAACACGTGGTCTTGATATCCATCGCG

GTTCTTCACCAACATTCTTTATTCCCCAAACAGCAGGGTTCGTCGAGCCCTTAATAGCTAAAACAGCTGG

TATCTCAATGAGGTTCTTACTCTTGTTGTACAGATCCATCAACGCTTGCTCTTCACGTACTGACGCAACC

CTCAACCTTTCGATGTATTCATACGCAGCGTTAATAGCTATATCTCTAGGACACGTTTCAACAATGTGTT

TTGGTATCGGTGCTATAGATCTTGAATTGAGCGCACCTGTGTTCTCAGCACTTTTAAGCAACGGTATTTG

TAGTTCACTAGCATACTCCGCATCAAACCGCAAATTCACTTTCAAAATCCTCAAATAAAAAATTACATCT

GCTGGTACAAAATAGAAAGGTGCTTTCAAAATTGTGGAATTAACATAAACATTCGGGGTTCCTTCTATAA

CTAACCTATGTATAACAATGTCTCGTAGATCTCTCAAACTTTCCACACTCATGACCACACGTGCCGGCTT

ACCGTCACAAGACTTATAATAATCAACATCACATTTGGCGTTCAAAAATTCAATATTGATGTCAGAATCC

TTCACAACTACTGCATACTCATTTGGTTTGCCAAACACGTTATGATAGTCAGTGATGACAGTGGGTTTGA

CAACAGTATCACACTGTTTATCCTCAACTTTCAAAACACTCTCAACTTTCTTGTTCCTATTCTCAACAGA

AGCCATACCAACAACACCGGTGCGATTCTTTCTACGGAACAATTTAGGTACAATTGACTTTTTTGGAGCC

GCATGTTCTTCGAATCCTTCAAGGCAAGCCCACTGATGTAACTCATGATCAAAACTTTCCATCATAATTT

GACACAACATGAATTGCTTGTCAACCATTCTGCAAAATGTGTGCTCACCACGTTCGCCATTGTGGATGAA

AACATTGTATACTTTTGCTCTCAAACTACGTACTCCAGCTCTGATTTCATATTTTTTATAGACAACATTG

TACCTAACCATCATGTCAACCATGCACGGTGTTAATCCAGGAATATTAGAAGGTATAGGGTATTTAACCC

ACAATCGCTCAGGCGTTTCATCCATAGTTGTTAACCTCCTTAACTTATCTTCAATATGTTCACGCGGTAA

TTCACTGACATCCACCACCGACACACTTTCCACACGTAGTTCAAACGGTTTTTTACTCGGACTAAACAAT

CTCATTTCCGGTTCATACGTCTTTCCAAGAATACTGTTCAACTCAGAAACAACTGAACGTTCACTATCCA

CACCAGATGACACTGTTTCAACAATCGAACTGATTTCCGTTACACTTTCATCTTCATCGTCAAAAACAAT

CGACTCAGAGGATAAGAGATGAACCTTATCATCACAATCTTCAACAATCCTTGAACGCGGCCCTTTTCTC

TTCTTCATGCCACCACTGCGTTTACGCACCAGCGTCTTGATGAGTTCAACATTCTCAGCACAAAAAAAAT

CATTCTTTCTACCGTCAGCAACATCCTCAAGGACGCGATAGTGATCAGAGTCAATTAACCCTTGAAACAA

ATGCATGTCCATTGAATTCGCAGGTGTGTACCCACTGCACGCCACAATACAATGATTATTGAACCCCGTG

AGCGAAGGCACCCATGGTATCCACACTTTGTCAAATGGAAGTTCTTTCCCTTTCAAAGTCCGCACGCGCC

AGTAAAAAACTCCTTCTTTATTCAATGCCCCAGCAATCGCTTCAAAGTCAACAGTATCCAAATCAGAATA

AATAACATCATATCTTTCTTTTGCATTCAACTTACATTCCTCAATTGCAACATAATTCAGCATCGTAGGT

GCAACTTTGTTTTTACTCAACTCACCCCAAACGCCATTCAACCTGCCTTCCATCAGTGCACAACACAACA

TGTCAGTAGTATAATGATCCGTGGTATATAGATCCTGTGGTGCACAACAAAAATATTCCGATATTTCTTT

TTTGTAATTTATCCGCGCTTCAAGCAACAAACTGATGGTGTCCAAATCATAGTCACTTTCCTCAATGCGT

TCTCTTGGTGTATTCGCTTCTTCCAGTGCTGTGTAATGCATTGATCGATGTTCAATGTGTATTAACCTGC

CGGTATTCATTATGAAACGTTCGTGTGTTTCATCAACAATCTCACCTTTTCCATCTACCTCAACACTATG

TAAGCACACATTCAAACCATGGGCAGCACACACAACCCACAACATCTCGTTAAGCGCCCACATGTTAGAG

GACGTCAATACATCTTCATATTCCTTTCGCGTTTTCTCCGTAAAGTCAATGTTCTTCATGTATTCCAAAG

TCGTCGCTTTCATTTCTGGAACACTACTTTTAAGCTTTCCCGCCAAGTTAACCGCATGAAACATACAATT

ACCATCTGCTAAAGTGAACATCCTGCGCAGACTGTTGTTACATCTGCTTACACCAACACGCATATACAAT

TTATCAGATCTTGGTTTCTTCTTTTCTTCTGATTCTTCCTTTACAAGAACAACGGTTTTTTCCTTCGAAC

TTTCACTGTCATCCACATCAGAACTATCTTCGTCATCACTGCCTAAATCAACTGTAAAACGGACGGCCGA

TTTCATCAATCTATCACGAACCACAGCTTTCGCTTCTTCGCTAACTATATCAACTATATCATCAGGCAAC

GTATCATAAACATTCATGAAATTGTCTTGACCTTCAAAATACGCACTCGCAACAAATTCACTCAGAGAAA

CTGCTGTCGCCATTTCATGCGCATCTATACCCAGTGCGTTGGTTATATAAAAATATCCAGGCACCTCAGC

CTTATCTTTCAACTCCTCAGTCGCACCCTGTTCCAACACGTCAACAGTATCAATATCCCTGTGTTTCAGT

TCTTTAGCTGTAAATGCCTTTGACAACATTCTATGAAACCAATTTTTTGAGTTGTGCGCTCGTGTGTATT

GTTCTTCTTCAGCAAAAATTTTTGCGGCCTGCGTCACATTAAATCTCTTTGCATAGCACAAACAGTAAAT

GCCAGTGACAGCGGTTGTCAAATCCTCTGCGCTCAAAGTGTAAGGAACACTCACGGAAACACCATTCATG

ATCAAACGTGCATTAATAGAACTACCTGCATTATAAACTACTTCATAACGATAACGCGATTCAGTCAAAC

CCATACACACATTATATAACTTATTGTAAAAGGTAGTAGGGACAACCACTAATGTAGAATAAATGTGCAT

AAGCCTACGCCCACGCTTTGTGAATCTGTTCACAAGGCCAACTGACCTGACAGGTCTGGTACTGTAACCA

ATAGATCTTATAACAGTCCTGTCATGACAATCGGTCGGAACAGCGGTCGTCAAAACACCACTCGGTATAA

CCATTTGAGAAGGGGCTTTCGTGATGGTAAAAAATTGAATTCCCTTGCGATTTTCACACAACTCTTTGAT

GTACAACGCGCCATCAATGACGAAAGACGATTGTACAATCTTTGAGAAATAGGTCTTAACCTCGTGCATG

TAAGACTGACTGGGATCATTGCAGAAATTAAACCTGATCATCAAATTTTTTCCCAGCTTCTCAAACTTAT

ACTCAACCTTAAGGTCGGTGAAATGACCATCCCTTTGAACCAGCATGACAGGTTCATACAAAAAACACCC

CGCCATCTTCTCACTTCCATGAGAATGCATTATGCGACATAAATCTTGAAACGAAATGTCATACGCAGAG

TCAAGAGCGACGCAGAATTGTGATTTTACCTTGCAATCTTGACTACGACGAAAACACACATGCGGTGATT

TTCCTTGATTTTTGTGTGCTCTAAGCAATGATTTTACCATGTCATTGCGCAGACTATTCGTTCGACTTGG

ACGCATCTGTTCCAAAGCAATTCTCCTATCCGTCATCCGTTTCGCATCCTGAGGTCCACCCAACCAAGAG

CAACTGTGTGCATCATAGATACCATCATGTAAATTCTGCAGGGGATTACCACCAACATCAATATACGAAG

CGCTGTTAACGCATCTATGATCGAAAGTCAATCCAAGCTTCTTGAACAGGATTCTTTTACAGCACATCCT

CGTCGCATGCAACATCGCGTGACCTTCAACGGTTCTTTTGTTAGAAACGGCGACTTCAACGTCAGGGAAC

ATCTTGTTAAGTTGCACCAGTTCTGCCGGTGTCAAAAACATGTTCAACCAATACTTCGTTTCACCCTTTG

ATTTAACGAAAGTTTCGGCTGTCGCAGCCGCTGACGCCGAAGCGTAATCAACCAAGAGTTCCATAACCCT

CGGGCTCCTCATCATGTCATTGGTCATTTCGTTGTAATTCTGACCAAGAATCTGTGTCAATACGCCAGAA

GCCAACACACCTTTGTGGTACTTCTCGTATGATTCTGGATCCATCTTTTGAGGTTCCGTGTTTGCATTCG

CAAAGTCGGCAAACCCATTTGGGTTGCCATTAGCAACAGCTAGTTCAGCCATTTTCG

>GAXI02034785.1 TSA: Tetrodontophora bielanensis s11022_L_28654_0_a_7_5_l_1239 transcribed RNA sequence/Negevirus

CAACAATCGTCAAGGATTGGTTTCTGTGCCACACTAACAATAGATTAAATTTCCACGGTTTAGGTATAAG

TTTAAAAACCAAGTTCCAACGCAGAAGCGGTGATGTCATGACGTTCATTGGTAACACTTTGACAGCGATG

TTCGCAATCTCTTACATTTACGATTTGGCCGATGCTATTACCGGAATTTTCGGAGGGGACGATAGTTTGG

TACTTTTGAAGTCAGACTTCCAAATCTTGGATAACGCAGCAGAATTCGCCAACATATTCAATCTCACGGC

AAAAGTGGAAAACTTTCCAGAAGCCATGTACTTTAGTTCTAAATTCCTACTACCTATCGGTAGTTACTGG

CACTTTGTACCTGATCCTTTGAAATGTTTGGTAAAGTTAGGAAGAGACGATATGTATTGCAGAGAACACG

TAGAAGCTTATTACGTTTCTTTCTTGGACAATTATCATATGTACAAATATTCGGAAGTACTAACATCAAC

AACAAAAGCCGTCCACATTCGTTACGCAAGAAACTTTTTATATCCAGTAGATGTAACCCAGAGTATTCAG

TATATTAACACTTTGTTACATTCCAAAGCGGAATTTCTAAAATTATTCACAGCTCCAAAACGAATTTGGG

ACAGAAAACTGCCAACAGCGTTAAAGGATTCCTTCAAAGACAGCACATCGTATGATATCTATCAGTATAT

GTACGAACCCGAATAAATCGAGAACCTACACTCAAAGCAGCTCACATTAAGTACGCGGTTAGGACAAAGT

CCAAGTAACCGGTCCGTTCAGTGTGTAGCTGCTTAGCACGCAAGTCACGACGTAAAGAGAAACAGTACCT

GAAGTTAAACAGTAAATTATTCGAGAACCCACACTCAAAGCAGCTCACATTAAGTACGCGGTTAGGACTA

AGTCCAAGCAACCGGTCCGTTCAGTGTGTAGCTGCTCGATACGCAAGTTACGGCGTAATGGGAAACAGTA

CCTGAAGTTAAACAGTAAATTAACAATCGGACATCCACAGTCCATCAAACAATCAATCAACAGTCAAGGC

ATAGATAATCTATGCAGCAAATCCAACGGTTCAATGTTGGCGGTTAGAAGGGAATGATAAGTTAAGGAAT

TATAAATTTGTTACTGCCCCGGAGGCCAGTACCAAATTTACCTAGCTATCATATCCAGAACTTCGAAACC

GCAAAAACAGTAACTGCCTCCGGGTTGATTGATTACATACAAACACGCG

>JYFJ01081229.1 Catajapyx aquilonaris Contig81229_fixed, whole genome shotgun sequence/Orthomyxovirus PB1

CAACCCTCCAATGCTCTCCCGCCAACTCCAATCGCGGCCGACGACATCAATCATGGATCGAGAACAGAAA

CTTGTCAGCCGCCAAACAGAAACGACTAAGTGACGCCCAGTGAAATTATTGTTCATTACTATGGGTAACA

AATGATGCCTTCTCAAGACACCATTCTCCATACATGGGTATTGGGATGTAGACTCCTACTCATGCAATGA

GACAGAAATAGGGCATCTTGAGAACCTGCTCGAGAGTAATACGACCTTCATCAGAGACACCCAGTGATTT

CCGCCAAGCGACATCTACGGATGCTGCAACTTCCAAGCACATGAGTTTTGTTACCATCATAACAAAATGA

CCCCTCATGATTACATGAAGAAAATTGATGATATAAATCAACAATTTAACAAAGAAACATGGTACCCGTT

CACATGGATCAAAAACAGGAACCAGTGTATAATTATACAATTATCCAACCAAAACAGACGTACCCCATCG

GAAACACAACACTGCAACTTCACTTCGTTGAATCCTGTCGAAGGGTTCCCCATTCCCATGAGAAGATATT

CCAAGTCTCATCAGATGAAATGGGGGTCTTGGGTCTTCTTGGCTTTCGATTTTTGCGATGACTTTAACCG

CCATCGATGGCTACCTATCCTTAGAAAAGTCTCAGATCTCAGTTGCCGACCTTTACAAATGCACAAAAAA

CACAGAAAATCCGTACATCCACCCACTGTATAACTGCAGCGGAAAAATAGGATCAACAAGCCCTATTTTG

CAGCAGCCATGGATGAAGTTGATTGGTGGATACCAAGATCAATGGACATCCACGCTCAATACCTGTCAAG

TACAGGTGGGTATCTGGTCTCTGGACACCTCTGCGTGGAACATAAACGTAACCTACTGTCATGGTGTCGA

GTACACCTCCAAGTATACCTATTGGTTTTTTGGGGGCGCAGACGGAGACCGTGAAGACAACCCCTGTGAC

GGTAGATGCAGTGTACTGTAAAAAAAATACATAAGGGAGCTAGATGAAAAGAGGGAAAATGGATACAAGG

GGAACCAGATTGGAGAATTTGTTCATGCAAAACATGGCTGGACAGGAACATCAACAACAATCTCACGGAT

AATCATGATCTCCAATTTAACGGTAAGTATGGATCATTGGGATCTAAGCATAAAAACTCTGATCCCATTA

TCTGGACATTGTCTCTACAACCAAACTTATTGTTACACTGCGGATCATGGGGTCATGATATGGGAAACTA

TTCCAGATCCCAGTTGGAATAAAGACAAGTGCAATCACAAACTAGATGACGTATTGACATGCCATGTGAG

TCTCTCCAAGAATACCGTGGCCACTGTAATATGTCCCACCCATGACTCCATGTTTCGTGTAAATATGACA

AGTGCACAGTTATTATGCAGTGTTTCTGACAAATCCATACAACAAAACCTCTGGACATCAATGGAGGGAA

AAGTGATATCCTTTATTACCATGAAGGGTACTGGATGTTTTCTTTCTTCATTCAAGGTTCCAGTCAGCGA

GACAGGCTGTTTTGACAAGAAGAAACTCAAGTATGAAGACCCGTTTGGGAATCTGGACCAACAAAACTAC

GTGTACCTCAATATGGCAAAGGTGGATGGAAAGATGCTGGAGCTAATATATCAAACAATCATTATGGTGA

GCAACAAGGCAAATCTGGATGCAAATTCAATAGATTCATCAGGAATTAAGGGACTGGAGATCAGCTGGTT

CAACTCAACACATAGGATGTATAGCAAAAAGACATCTGGAACATTGATCCAAAGATTGTGTACACTGAGT

TATGTGAAATGTCTAAAAATAAAAACGGGATTAGACAATGACAAATGTTTGAGGGCTGAGATAACGGATG

GCACGATAGTGAGGTTTGATCCAACAAACATGATTATCGTGAATGAGGAATACGAGTGCCAGTCATATAA

AGATTCTCTCCTCTTACTTAAAGACAACACGATTATGGACTTAACCACTGGGTCCACTGCAAACGGGGCT

GTTTTGGCAAACCCTCATCTTCCTTCAATGGAGGTCAGCTCACACGCCTCCTTGACAGCATTAGACCTGG

ATCGGATAAGTTCCCTATCTATGGTGTTCGACCAACTTAATGCACAGTCTCGGACCCTTATGGATGAGAC

AAACATGTTCCTCGAGCCGTCATTTGACAATGACAAATACAAAACCTCTACAGACACCTCCTCAGGTCCA

TCATTAGAAGGTTGGAAGAACTGGTTATTAGGCCCACTGTGGTCCATATTTGCCTCCCTCATCATAACTG

TAATCATCATAATCATAACCATTGTCTTACTCAAGTTTGGGACCAAAATGATATTCTGTCCAAAAGACAC

CAGCTCAGAACATGAATACATTTATATGAAACCAAACCCACCCAGCAAGAAAGATTTGAACAGATCACAC

GATGCATTCAGATAATATAACATTTTTTTTAAAAAAAAAGTGTCTTGAGTGGAGTTGCTCAGGCGTTTGT

ACGTATGTTGTTTCTGAGTGCTTATAGATAAACTTTTTGAGTGTAATTGGTTAGGATAGTGTTGTTCAGA

CGGCACAAGTGTTAATAAAATTAGTCCAGCTTTCCTGAACTAATTTTATTATTTTAAGGGGGGAGAGTAT

GTGATGACCCTGGGTCATGACCTGATGATTAGTGTGCCCCCGGGTTAGTCCATATTATTCCTCAAAGTTT

AATTGCTGCTTATCTTTTTCGTTGCTTCATCCCAACCTACATTCCTTTCCTTTTGCGCTGCTCCGGCTGA

TGTAAGGTATTTTGGGCAGCGCGAGGCAGATTGGAATGTTGACCTTTGTACTTGGTGCTGTCTAGTGAAT

TCTTTCTTTTGCTCTTCTCGGGGTCGCTCAGGAGCCAATCGGAGTTAGTAATGTAGAGATGTACTTGATG

CGGTCATCTTTGGCCATCCGGGTTAATTTTGCGTTTAGTTTTTCTACCAATGATTTCATTCTGGTCACTC

CGAAGCTTGAGCGAGACCGAGGAAAGAAGCCCCAACTTGGGGACAGTTGGCGGGCACCTATGTAGTGAGT

AACATGTAATTTCTGACGGACGCTGTGGCCCAGCCCTTAGCGCGGGGTTGGTTGGGCACTGGGCAGTCGA

AATGGGGGTCTTGGGTCTTCTTGGCGTCAATTAGGCGCGTTCCGGTTTTTTTCCGGACTTAAAACTTTTC

TTGTTTGAACTAATGTGAGGCAACCGGCTTGAAATTGTGATAGCCAGGCGTGAGTGGAGTGATGTTTAGT

GATCCCTCCAAATTGTGCGGATTGAGAGAAAAAGTGACGTGCAACTGGAAGATGATGGCCGAAAAGGACG

CTCTGGACAACCTGAGGGAGAAGAGTGGAGCGGGCCAGAAGTCTGGTCTCGTCTCTGATTGGTCGAGACG

ATTGGTCTGAAGCCCCCCGATGGTATCTTCACCTTATCGTGGTGGTGGGGTTTGCGTTTCTCCCGTGACC

CCAAGAGCTATGTCGGCGCGGCGGTAGCTTCGTCTACTGGTAGGGCCACCCAAGCCGGACAGGTCTTTGG

GATAGGAGACAGACTAAGAGAGATGCCAGTCTTTCCTCTCCGTTTCTCTCCATCGGTCACACCTTTGCTT

CTTCTACAGCCAGATAGCCACAGTTGCTTCGGCTTCTTCCTAACAAACCAAACCAATCTGAAGCTGCGCT

GTGATTGGTCAGTTATCCGAGACTGGCTCATGTAAACTCGGACTATCGCTTGGCCTTTGCCCTTATCAGC

AGACCTCTGACCTCGACTAATTGTTGGCACGCGCCAGGGCTCGTGCCTCATCAGTAGAGCTCGGAAAAAA

CCGCAGCATCAGCAGGGGGGCCCCCACAGGACCTGTGACCACCCACTCCTCCCCCACCACCTACCTGGCC

GCTCACCCGCCCAGGTGACACCCCGACTAAATAGGCCCGCACCTTTAGAGCATTGCAGCCAGCCATACGT

CACCTGGACACCCGCACATGGTCCCACGTGACGCCCCCATCCCACCACACCTCCACCTCTTGAGCTAAAA

CAGGGGTTCCCGCTGACTGCTAGAACCGAGGGACTCTGGATTCAGAGTCCAGCGCCATAGCCCCTCGGCC

AGCACCCCTTGGTTGGGGGAAGGTGTGTTGGACCCCGTATGCACATGCCTCGCTGTGACGTAGCACAGGA

GGGGTGGGGCTGTTTGGAGGGGCGGGAGCCATTTTAGTTTTTGGCTGGCTGGCTGGGGCGTTGAATATTC

ACGCAATCTCTGCCTTGGCTTATCAGTCTTGAGATATAACAACTAGAGAGACTGGAGGTGAGAGGTCACC

TAAAAATAACCCCCACCAACTCTATGTAACGGGGGGCATGGGGCAGAGGGGAGGAGGCGACGCCATGTTG

ACAAGGCAGCAGCCATTGTGTATCTTGGGCATCTTTTGCATCTTATGTGTGCACTCTGGGTCACCAGCTA

CTTATGCTGTTATCTTTCCAGATCTCATTCAAGCAGTATTATAAAATAGCATATGATCTGCATGTCCGTG

TCAGGAACCAGCCCATGCTTGTGGCCCAGGTGGGAGACGAGGATCTTCCGGGACCATTATGTTTTGTATA

TATTGTCCCGGAGCTGTGCTGGCCAGTTAGCCATCAGACTGCAGTGGAATCCGGATTACCTAGGGTTATC

ATATACCCAGACACTGCAGACACTGAGGCTACTGGAGCAGGAGGGACATCTCCACACCCTTGGCAGTCCC

CAGTATGTTGGTTAACAGACTTTCATATTCATAAACTCCTGCTTATTTGTTTTTTTCTCCCTAGGAAAGA

GAGCAACAGCAAGACACAGCATAGAAATGTGGTTATTTATTTATTTATTATTATTAAGGTTGTACAATAT

TATTGTGAAATATATCAGATCAACTCTATAATAGTCTGGAACCTTGTCCCAGGTGTTTTTCTCCATAAGG

ACCCATTGTTTAGCCTTACATCGGGCGATTCAGCAGATATGTTATCACTGCACACCACATTATGGAAGAA

TAATGCAGATAACGCCGCTTCCTGGTCTGGTCCACTTCATCGTCGAACACCCACAAGTGGGGGTCCGTGA

CCCATTCCACATGACCCACATCATTGCCCTGCATGAAGGTACTACATAATTATATAGTGTAATAATTCAG

ACGCACACCAAAAGGTCAATGAACACCACATCCTTCGCATTTTTCATGTCTTGTGGTGTGATTATCCTAT

CTGAGGGTTGAGGGAAAGATAGGAGCTGTTGGTTGCTGGGTGATATGGAAATGAGCCAACATCCTCCAAC

TTTTTCCAATCATTCCGAGCCATGGTTGCGTGGCGATCACATCATAAACAGAGATTGACAATTGTGTAGC

AGAGTGATGACAGTGAGGAAGATGAAGAGGTGCAAAGGAGACGGCCAGCCTTGCAGGATCAACTCCGGCG

ACAGAGAGTGAAACAACAACAACGTCGACGCACGTAATGGAGGTTCCAGTCTGCCGATGCCGGTGGACCC

GGTAGCTATATCAGCGATAGAGCGGCGGGCTGGTGTCCTCTATTTGGGGGAAGCTCCATCGGAACATGGC

TGCGAGTAGACATCTGGAAGGTCTTCAGCTTTGGCTGGAGGTGTGGTAGTGGACAATGGACCCGAAGGTG

GATGTCAACCGGCCGGTTTATTTTCGTCCATGATTATTCGCAGCATGTGCCCAACCTCTTCTCTGCTAGT

GCTAAGCTTTGTATACATGGCTAACCTGGCGTTCATATTCGCCCAAACCTCCGTGGCCTGAAACCAACCA

CATTTCTTCCAGACTGGCTGGGCCCCCCTCTTTTTCCGCCATATATGCCCAGTTTTGTTGGCACGATATG

GTAATTGGCACGGTATTGGCACGAGCTCGGTCTGTTACATTGTTTGTTGGCATCTGCGAGTTTTTTCCCT

AAATGTACCTCTTTTGAAACATTGTGCGCTCCGGGGCTTCCAATTATCCGCGATCGCTCTAACTGTTCGA

ACATTAATACACCGTCATCCTGCTGCTCTCGATGGAACTGTTTTTGTGGGAGAGCATGATATGCTGGCCG

CGAGCCGATTGTAAGTAACGACACGTTCGGTCGAGTTGTTTGCATCAGGCGCAATTATATTTTCAGATTC

CTGCGTAGTCGAGCGTTTATTTGGGGATGAGCTGCCCAACCTTTTTGATGAACAGGCCGAATTGGGATAC

TTGCTGGAGGTGTATAATTGCGAGGTGTGGGATGAGGAGGAGGGCGGTGGCGGGGTGGGATGGTATCGCA

TTGTCAGCGACGTTGTCGAGGCGGAATTTGACGGCGGTGAGTTCGTAAATTTATTTAACGAGAGGCCTCT

CCCAAATCTCTACTTGGATTGGGCTCTGGGAGGCGGTCCTTGGCGCTATTACTGTGTCGACGATTTTTAG

GAGGAGGATGATGAATGGGTGGGGGAGGCGCTGGTAAATTTATTCAACGAGGCTCCCCTGGAGGGCTATT

ATTTGGAATTTGTGGAGCCGGATGATCTGAGCGCGTCATACTGGGCTCATGTTCAGAGTCCGAGCCGGTT

GATTGGCATCTTGCGCGACGAGTTGCCTTTTGCATGGAGCAAAGGATACTAGGAAGCTAATTCACGGTCG

CAACCTCCCTCCTTCTTCCCCCCCTCCGATATATGGCACTCCGCACGCCCCGTCGTTTCACTCGAGCCAT

GTCAGTCCAAGTGGTTCTGTTTATTGGGGTCGAACCTCACACGTGTTTTAAACTGCGTCAGAGCGAGCAG

GTGGTGGGGGTCTGGAAAGATGCCCACGAGGTTTTGGCGGATCCGGGGCTCATCCCGGCTGGCCTGGCTC

ACACCTACAGCTATTGCGTGTACCCCTCCTTCACTTGCACTTGTCGGGTGCAGAAGAAATTCCGTTTGGA

TATTAATAGCGGCGTCTCTTGGCTCTCTCACGCCTTGTGCCTGGAATGGTATAAGTGCGGGCGCGGGAGT

CTTTCCCGGATATACTGAATCATTTTTCCCTTCCAGATGCTCGGCTACGAGAAGATGGTTCCTTGTCGAG

CGGTGTACCACATGACCACGGAACTCTGTCAGCTGGCCGTTTTCTGCATGTCCGAATTGGACATGCAGAA

AACGGCCAGCAATTGACATTCCTCCAAAGTAAACTTGCAGCCAACAACTAAATGGACCAGAACCCAACTC

TAAGGAACCAGAATTGAAGCAAACTCCAGCATGACGGAACATCAAAGTGCAATACCCAACATCTATGTAA

TTATTGAGCAAGACCATCTTCATCCGTTGTGGAAAATAAAACGTTGCTCTTATGCCACACTCAGATCCAT

AATTAAGTCTTTTCATACCAAATGACCACTGCATTGGTTACAAAAGTTTCATTTAAAAAAAATATATGCA

AGTAGCAGCAATGGTCTCCGTAATTACGGTTGTAAGATGTGGTTATTAAAAAAGTATCTGTATCCGAATT

TGATGCAGAAGCAGCGACATTTTGTAGTGATGTGACATCAACTTGCGATGCCTTGGGACACTGCGTGTCA

AAACGATCAGTGTGACACAAAAGGTCAAATGTCAGTGACTGAACCCAGTCATGTATATTTAGACATGACC

GGCTTTATGAAAAATTGTCACATATATTTATGTGACAATTGTGAATTCGACATATATGAAGCTGCTGTCT

ATTCCGGAGATTTGCAAAAAGGTTAGTTCTGTTGCCCAGAATGCGTCATGTTAAAGCTTCGTTTTTTACC

TCCCGCATGGTTATTTTCAATGAAACGTTTGCTAGCACAAAAAGCTCTGTCGCGCCAAACAAATAAAACC

ATTACTGCATAAACGTGGAATGAAGCAATTTGCAGCCGCAGTGCTCAGGACATTGCCAGCGCATTCTTAA

CATTTCTCATAGAAATTCGCGATCAGGACCGTGTGATCATACGGCTCGAGAATGGCTCTTCCCAAAATAT

GACACCAAATCTCTCTACAACAAGGAAACAGATCAACGCGAGCTCAAGCACAGAGAAAAAATGGTAATGC

AGACCATAGTCATAACAGGGAAAGACAAATGCTTCAAATACATGATGGACTTAATGAGATCATATGGGTC

TTACATAAAACACGACGAAAGGTCTAAATTGACCAGGAGAGCCATAGCCTCCCCATCAATCATTCAGAGG

ATTTTCCTATACATCGTTGAGGACTTCCATTTGGAACTGGGAAAGGAACTTCCTGACTCAACAATCTCCA

TAGGCGGCGAAGAAGAAGAAAAAAAAGGCTAAAATCACAACCGAATTGACGGCGGGATCCAAGGAAGGAA

TTTTAATGCCAAAAACAGTCCAAGCAACTGAGGACGCAACAAAATGGAATGAATGCTTAAGCGTGGAGTG

TTTCTCTCTCTTCCATGACACAATTTTGGGTGAAGAAGCAAGAAGTTTAACTGGTCTACCAAGACCTAAT

TCAAATGAAAGACTCTTCAACCAAATACTCCAAGCCTCACATTTCAACCTAGCAGTAAAGCGCATTTTCC

TAAATGAAGGCCCCATCTGCATTCAAAAGTCTGAATATAAGCGAGTACCTTATTCCACCCAAAACATAAG

CAGATTCAATGATAATACCAAAGAATGGGTTTCCCAAGCAGAAAAGCTATTCGATGACAACTACCTAACT

GCTAGCCCTGGAATGCTCATGGGAATGCACAACGCACTGTCAACTACCATCAGGCTACTAGCCGAGAACT

TCCTGTTGGACACCACCAAAAGCAATGTTACCACACTCAGGTCGAGTGACGACTCCATGACTGTTTTTAC

AAGAATTGATGATGAATCAATGATAGAGGCCCTACACTCTAACGATTATTGCCTCAAACTCCTAGGAATC

AACATCAGTGACTCCAAATCTTGGCTGTGCAAGGAATCTTTTGGTGAGTTTACGTCATGGTACCAAGACG

GTCAATTCATTGCCCAATTTGGGGTGGAAACCGCCAACATGAGAGCTGGTGGCCAAAACCCAACAGATGA

CTTCTTTCAAATAGCAAAGAGCACTGCAAGCTCTCTATCCCGCTTAGAGATGAACCACTGAGGAGCACAT

TGCAAGCTGCCAATCGGCATTGACAATGTAAGAAGGTTGTGGAGAATTGAACAAACCCTTCACAAAAGGC

AAAATGTGTCTCCCAAAATCCTGGTTCTTGCTGACGGAGGCCATAGGACCCCACTAACGCTCATCTGGAA

GAAACCAGCATTAAATCAATGTGGACAACAACCAAAGAAGAGAAGGAGTACCTATTTAAGATAAGGAATC

CTGAAAACCCTTTCTCTGCAGCTGCCGAGGAAGAAATAAGTTTTCCAAAGGAAACAAACAGCATTTGTTT

GACGCTACTTGAAAATCCTAGGACTGTCTTCAACTTCATGAAGAGAAGCAATAGAACTGGTTTGAATAGG

GGCAAAGACAATCTAGAAAATGAGAAGTCCAACCTGCATGCTCTTCAAATCCCTAACCTTATTGATTGCT

CAACACTTCTAAAGGTGCCTTCACAGACCTCCACTATTGCAAGTCATCTGACCGGGATGTTAGAGACATA

CGCGTCCATATTTAAACTTAGTGACAAAGAGCTTAAATTATTGGCAGACGCCCAAAATAGATTAAGGAAT

GTAGACACTGATGATGAGAGCCAAACTTTAGACTTTTGTTAGAGGACACACACAATGTGTAAATTGAAAA

GAGCCTTGTTTCTACTGGCTGCCATCAACTCGGAACTGATTAGCGCAAACGAAATCATTTTGCGGTACTT

GGTCCCAGGACATACATTCATGTCGGCAGACGGAATTCATGGGAAAATCGGTTCCTAATGGACGCAGTTC

GGATGCCTAACGCTCCACAAATTCAGTTTTACGGAAAATATTGGTTTGTGCAAGTTAAGCTATACAGTAA

CTCACCCTACATCCTCACAAGAGAATATGCCGACCTGGATTTGAATGGGGCAGGCAGCAAGT

>GAUG02039188.1 TSA: Meinertellus cundinamarcensis C284869_a_43_0_l_5753 transcribed RNA sequence/picornavirus

CCTAACTTAAACAATTTTGAAAAACATATTGGAAAACAAAATTATTAATGATTTTATCGCTTTCTCTTTC

CTTCATTAAAGAATAAAATCATTTTAGGGTTTTAAAGCCGTGCATAAAATATAATCCATCACAGCCACTT

GATCAAAAGGACGATTTAACTTCTTTAAAATTTTTATTACCTCTTGAACATGTTTAATTAATTTTGGAAC

ATCAGTAAGCATCTTTGGTACTGACATAACACCCGCCGCCTCAAGGGGTGGAAGAGTAGGTTTTGTAGCT

AAAGAATATTGAATATACTTCGCACTCGCCCATACTGTCATTTGGGCTGATGGTGTATTTTCTGAAATAA

CTATTTGAAGCGGTTTAATAACTACATATCCATTAAAACTATCAATTGCTTGCTTATGTTGCACAACTGG

ATAAGTTTTCATCCAAGGTATTGGAAAAATTGTCTCACTCCCTCTAAAAGAATGTTCAACATGAGGATAT

TGAGCCAATTCTCCTGGAGTTAATTGACCTGGATCAGCTATACCTGGAGGTACTGCTGCTATTAAAACTT

TACCATTTGCATAAGCATTTGCACTTGACGTAATTTTAAATTCTCCATACAAATTTTGTCCTTTTACAAC

TGTTGGTCCACTAAAAATAAACCTTGAAGCTTCTAAATTAGTTTGAGAAAAGGTTCCAGCAACATCTGTC

CACTCAAAAGGATTTACTCGAAAAACATACGATTTATCATCTGCATCTTCATATGTTATAGTAGTTAAAG

GAACAAATACTTGATGAGCAACATCAACATTAACTTGATTATGCCCAGTTGTATGATCCATCGAAGTTCC

TCCCTGATTTCTAGGGGATATATTGACTTTAGAATTTGAAACTTCATCTTGATCAGATGATCTTGCCCCC

GTTTGAGTTTCTGCACTAACCAAACCTCGACCAAAAATCTTGTTTTTGAGCGCCTCTCCAAAAGAAATTT

TCTTTCCTTTAATATCCGATTGTCTAGTAAAATAACTTCCATAAGGTGACACAGTTAAAGCATCTGTCTG

AAAATCTCCTGATGCTGCGTCAACAACAAGATAAGTTCTTACTTCTGTAGGTTTATCTGGATTCACAGCT

GTAACATATTTTGCAACTTCTAATTCCAAAGCCTCAACATCTGGATCTTGTTCCATACTATGAATAAGCC

ACATTTGCCGTTCTCCATCGTCAAACTGAACAGGAACTGGTGTAGCCGAATGATCAACTCGATACGGAAA

GGAATCATAAACAGGTAAAAGTGTATTAGAATAATAATCATTATATAATGAAACATTATTACCTTCAGGT

GGTGTTGTTACTACCGTTCCATTAAATATATGATATTGAGGTTGAGGAGCTACAGGAGGTGTTTTAGTAA

ATTGTGATAAATCCGAAGTTGTTATAGAAGCTTGTAAATCTGCTTCAACATCATCAGACAATTTTGGAAT

TAACGTCCTAAATACATAGGAACCATCTCGTATCATTAAACCAATAGTTGCTTTATAACAATACCATTCT

CCAGGTTCATCAATAACTCTTAAAAACCAAACATCTCTTTGAACTGAATTAATAGTTACCTGACCGGCAA

CTGACTCTAACTTGTTAGGTGTTGAACTTGCAATTATATTACTATAATAAAAATCTACTGTTTGATCAGT

AGCTGTTCCTCCTAAATCATAAATAATTGGTTGTTCTCCTGTAGACACATTAGAATTTACTATTACTGTC

AAGGATTCTTCAGGATGAGCTTGTGCATATGCTATATCTTCTTCTAATGTATACTCAGGTTGAGTATTCA

TTATAACTCGTGTTACCGCTAAATTATTTCTCAAAACACTCCCAAACGTTACTTTGGTAACTGAATAAGT

ATTACCAATACCCAATTCCAACACTCTAACTGGTTTCAAGTCTGATCCAATCATAACTTGTTCAACCACT

GAAGATATTATATCAAAATGTGCTGGTCCTTCTGTTGACACTGATTCATTAATAACACTAAGTCGAACAC

CTAATTTATGAGAATTTTGTCGAACAGTACCCACTGGTTCAACTCCTATAGGTGGAATTGGTGAAATTTG

TATTTTAGAACCATAATCTGAATCCAATTCAGCAGAAATTAAATCTTGATCAGGATCTTGAAGAGAAACA

TGATCTATGATTGGTCTTAACTCTATTTCTCCTGCAACCGTTACAAATGGATTTAAAACTGTCCTTATAA

ACCGAAAAGTCACGCCTTCCTCTTCCTCATCTGTTACACCCACTTCTAATTCACGAAATGGCCGTATTTC

ACCATCCTCACATTCTAATTGTACTATACGAGATCCCTTAAGAAAATTAAATCTCTTTAAATTATTAGAA

ACTTCTCCAAATGGATTAACTACTGATGTTGGATATTGAGGCGAAGCAGCATTTTGAGGAACAATATAAT

ATGTTTCTTGTCGAGGTGAACCATCATCTGAAACATGCTTCTGAATCATTACTTGACTCTCTACTGGTTG

AACAGCATAATTCATTCGAGCTGGTACTGGAACACCAGCCTCCACACTTCGTTCTAATTCAATAAAAGCC

AAAGAAGCTTGTTCTGCATCAAAATTTGTAGCCCAATCGGGTCGAATAAAAGGTACATAAACATAAATAT

CAGAACAAACAGCAGGATTCCAAGTAAATCCACGTTTGCTAAGAATGGTATTTTGAGGAGCATTCAACAA

TTCCCCTGGTTCATACAAACCAAACTTCAAACGAGAAGATATACCCAATGGATACATAACATTAAATCTA

ACTATAAAATCTGTTCTAATCTTTTGAAAAGCATGTCGTAAAATATTCAATCGCTTGTTATCTGATGGCC

GAAAATTTATTCGATGAAACTCTCCTTTAGAAATACGAATTTCATCCTGCCATTCCATAATATTAAAAGG

TTTAAAAGCCTTAAAACTAAAATCTGCCGTTTTAATCTGATTGGAACATGGAAACTGTTTTTCTAATACT

ATTACTGGTTTTAAACCATCACAAGTTTGCATTTCACGAATAATAACACCATCATCTAAAGTAACACCAT

CAATATACATTTGTGCCTCTGGTGGAGGGGCATCTGTTTGTTTTACTGATTTATTTTCATTTCTTCGTTC

TGTCACGTCTTGGATTTTGTCCGACACATTCTGCAACTTCTCAGCAGGTTGTGTTTTAACAACATCTGAA

GTCGTTTGTTTTGGTTCCTCCAAGGGTCGATCCCCTATTTCGGTAACCACTGAAGTTTTCTTTAAATCAG

ATGAAGATCGAAAAAGAATCTGAAGACGATCTACAAATGTTTGAATTTTAATGATCATATGAACTTGATC

AACAAAATGGTAATTACTTTTAGAAAAACCTTTTTGTAATTCTTGGCGAATTATACGAAAATCTGTTTCC

CAATTGAATCCTTCAAAAACTAAACATGAAGCACTATAATATCCTTTTGTAACATTATAGGCAACGTTTC

CACTCATAGAGATTTTTAAATTTGAGATCAATTCCCGAAAGAATCGATCTGCTTTAACTATATGATCATT

TTGATTACACATCTCTGTCTCCATTTCTTGTATTAAATCTAAAAATTTAAATTTATAATGACTCAAATGA

TTTAAAAATCTATTTAAAAATGATTTTGTATATTGCCACAAACCACAACCTAAAGCCGGTATATAAAATA

CTGCCTGAGATCGTAAATTTAATATTTCCTCTTTTTCACTATCTAAAAATTTTAAATAAGGAGGAAATTT

TTCACACGTTAAAAATAAATGTAAATCGGAAAAACACTTTTCAGGACGTGTTCGAATATTAATAACTTTC

GTACCGTTCGACAATGTCGTTAAAAATATCGTAGCTTTACTCTGAGATTTTTCAACAAAATCTGGACAAT

TATCTGCTATAGCTCTAGCAAAACCAGCAAGATTTGATGTCTTATTCCAATGTAGGAAATGCAAAATAGG

TCTACCTTTAGTATTTGTAAAAACACTATAAATCTCAGGTGAACTACAATTAGAACAACGTTCTATCGAC

GCACAATTAACCTCTGTTAAATCCGGGTCACGATTCTGATTACTTTCAGCAATTTCTACAACAGGGGCCT

GTCCAAAATAACGAGCAACAAACTCTTTCTTAATATCTTTGAAAGTCGGTGATAAAGTTAACCGCAATTG

ATTCATAAATAATTGCTGAAATTGTTTATCAAATTTTGAAATCTTAACATACAACTGATTTTTAAAATAA

TTAAAATATTCTTGTCCATGTAACATGGCTTCAAATAAAGTCTCGCCAATTGTGACACACCATGTTGTAT

TATCATTCTCTTCGATTTGGGAATAATTAAATTTTGATTCAATTGATTCCTTACGAAGCGGAGCCACTAC

CATAGTTTCTAATACTTTGAAACGTCTTTGTAAAAAGGTTGCTTCTGATAATGTTTTAAAACCTGATGCA

GTTGAAATTTTATCCCCAGGTGTTATTACTTGACCAAGATCTTCATTCATTATTTTACGAATACTTTCAA

AATTAAATTTTTCAACAATAGAGGGATGGATGGATAAAATTGCATCATCTCCAAAGAAATAACAAACTAC

ATTTTCACGAAACTCTTCAATAGATCGCATTCCAAAAATTTTACAATAAACATAATAATGATAAATAAAA

TTTGCCCAACAATTCATCACAGTTGTCATTACGGCTCCAGATGGATTACCATTCTTTTTCATAATTATAG

TTTCTCCAGAAACACAAAGTGTTGATATATTTTCTTCCCACAATATTTTTCTAATTTGAAAATTCTCTTC

AGAATCTCCTTGTGTAGCATAAATTGTATAATTTATTATATATCCTACCATCTGCATTATAACTTTAGGA

ATTCGTTTATCAAAATTTTCAAAATCTTCATCTAAAATATGATGATATTTAGATAAACTCTGATATAATA

TACGCCAATCAGTCGAATGAGGATTAATAGCCACAGCATGATTAAATTCAGTGCCCTTATCTGTCCAAAC

TGCCTTATATTTACCAAAAGCAATACGATTAGCTATTGTTGTTTCTCTTGGACTAGAAACAAACATACGT

GTTTTAGGTTTCTTAATTTTAGAAATAGGACGGCATTCAGACTTCAAACAATTTTTCCAAAATGAGACTA

CTCGTTTACCCTTAGCTGCCATTCGTAACTTAAATTGAACCCGATCTTTAATTTTACGATATATACCTTT

ATTAACTTTCTCTATTCTGCCCTCTTGAGAAATTTTAACAAAATCCGTTCCAACAGGAGCTCCCATCTGA

CACCAAGGGAAACCATTTGCTGATTTAAGATTCATAGGTTCAAAATGTTCTGAATTATTTTTATCACCTA

AAATCGCTTGTTCATACGTAGCCATACCTATATCTTCTCCATCCATTACTCGAATTGAATAATCAAGACA

TTCAAACATTACATGTTCTAAAATATCAGAATCTAATTTTGATGGTACATCTTGATATTTATCTGTATTT

GTTACTAATAATGAATAATTTCCAGCCGAATCTTTTGATAAATGTGAAAAATCTTCTATACGAGGATCAG

CAGTATCTAATACTGAAGGCTGAGTACGCTCTTCAAAAGCTCCAAAGAAAGGAGATCTACGGATTACTTC

ATTTGCTCGAGAAGAAACCGGATTTAAATATACTAATTTTCCATTATCATCTTGCAAAGAACCAACATAA

TCCACAAAACCAC

>GAXJ02019692.1 TSA: Occasjapyx japonicus C98453_a_21_0_l_7941 transcribed RNA sequence/picornavirus

GCAGGTAGGAGGAGTCATGGATAAAAAGAAAGTTTTGCAAAGAAAAACGGAAAAGCTAAGCAAAAAGAAA

ATTTTAAGCGCCTACAAACTAATTTTCGATTCCACAGCAGGCTTGCCCAGATTCAACGAGCGCCGGAGTA

GCAGCACGCTCGCGTTCCCGCACAGCTTCTTCTTCCTGTTGACTCTGGCAACTTCGCGCCTCACCACCAC

TGGATTGTGGAGCAAGGGTATGATAGTTGCCGGCAGGAAGCAATTCGGCTGGCGAAAAGCCCCAAAACAT

TCTGTGAACGTCCGAGAAACTCACCGGGAGAGGTTCAAGGCCCACGTCACATAGTGCATGGTATATCTGG

GTTTTAACCCTGTCATAGTATCTTCGGCCGTGGTGAACACATTCCCTAAGGACTGTGTCCGCGTTTTGTC

GGGTGGATTCTCCGGCGGAGATGCCGGGTGAAGTTCGTATCCAATTGAGGGTCTCTAGCATCGGCCCCTC

TTCCATCGGTGCGTAGACGACGTTCCCATCGGGACGGAAATTCCGTTTGAGAAACGTGCATTCGGATGGA

GAGAGGAAGGGACGTACAATCTCTCTTTTCTGGGGGTCGGTGAATCCCATACCCCGCGACTCAAACCATT

CCTTGAGGGTCAGCGTATTAAACCAAGAGGCGCACGCATTGCGGACGGAGAAGATGAGATCATCCCCGTA

CGTCAAAGCACGCACATTGGCATCATAGTGCTCAAGGGTGGCGTAGTCAACGGGTTCGGGTTCGCCTACC

TTCCATTCCTCAAGCGCGCGGCGATGTTCTTCGCGCGCCATCGAAAGAAAAGCGAGGGCGCAGTACCAAT

GGCCAATTTCGCTATTGAAGCTGGCGGTGGACGGGGATCCGGATGGTATTCCCTTGTTCATTCGATACAC

CGAGGAGGTTTTTCCTCCCGGCACAACGAAATGAGCGTAGGAATGATCCTGGGCAAGCGCTTGAAAGCAC

TTGTCATGTCCTCGAAGCGAGTTCCCATGGAACTCGTACCAGCGTCGGGCCACATCAAACAGCTTAAAGT

TGAGGTAGGGGTCAACTTTGCCATCAAAGTTCTCGTGGTCTCCAGCGATCCAGTTATCTCCGTACTCCTT

CAGCCAGGTGTACCAAAGCGCCCAATCATGGCCGTGCGGGTTTGTACCCACACCATGGCCAATCTTAAAG

CGATGTTGGGACAGCGAGGCTTGGAAGTCCATGAGATGCCTGCGATGGTGGAGCAAGAATGGCTTGGGGC

CAGCTGCGAAGATGCGGCACTTTAGCACTTTGGCTAGAGGCCTTCGTTCATCCTTGGTGGAAGCGGTGTA

GAGGGAAGGATGGTAAACCCCTACACGGGCATCCTCGCAGAGTTGGAGCACCTGGGAACGGGTGTCCTCA

TCTACGAGGGTTAGACGGTCGTCTTCCTGCTCGAACAACCACAGAGATCCAGAGGTGCCCGGAGGGCGCC

GGAGGACCAGAGGGTAGCCTGGACTGGTATGGGGATCAATCCGGGCGTAGCCAGGAAGTGGGGGGGGGTC

GACCGCCTCATCAACTGTCAGCTGGTGATCAAGATTGCGGATTGGAGAGCAGGCCAGGTACTTCGTGAGA

TAGTACTCGGCAACTGCATCCACGTCCTGATGGCTGAAGTTGGGAGGGGCTCCGTTGAACTTCTTGAGTC

CAACACGGAGGGGGGAGTGTCCTGACGGACACCTCGGATCCTTGGGGGTAAGAACTGACGGGGCCGTAGC

CGGGATTGTCAGCTTTCCCGCAATGAGGGATGGAGCGAGGGTCGTCTTTTCGGGGGCGCGTACACCCGGC

ACTTCACCTTGGTAGGCTGGGTGCCGTTGCAGCGGGTCTTCCTCCAACAGGAACGCCTGAGCTTCGGGCT

CTAGCGCACAGTCAACACCCAGGAAGTTGGAGGATTCCTGGAGTCGGTGGGGGTAGGGGTCGAACTTGTC

GAGTTGCTCCTTTAGGAACTCAACTAACCCACGGTGGATAATGAGGGACCAGCCTTGGCCATTCATTCCA

GCCGCGTGAAGGCCTACAATGACGCCTGTCATTTTGGAGTTGTCCACAAGAAGCGGAGCTCCACATAAGC

CCTTGAATTCTGAGCATCCGTATTGGAACGACTCGAGAAGTTCGTAGGTAGAATCCCCAGTTGTCACCAC

TGTCGTGGGACGACGCGAACTCCGCCACTGCACCACTTGTTGGTAGTAGTGGGCGACAGTATCAGATGGC

TCCTGGATGAGATGGAGCAACTGTCCTGTGCCAGTGGCGAGGTAGGCGACGTCTTCGGCATCGGCAACGT

GGGGGTTCTTCGGAAAGAGGGGGAGGTGGGGGGGAAGCCGGCAGATAGCGAGGTCCTTATCCTGGAGCTC

CAAAAACACACACTCAGAAGGCTTGACAAGCAAGTTCTGCTGACTGCGGTTGAATTGGAACTCGGCGCAT

TCATCGAGAATACGCGCACAGTGGCGGTTAAGGAGGAAGTCGCTTCCGCCGACGCAGAGGGCGTGCATCT

TGATCCCTTTGGCTTGGATCAAGGCCACGTGCTTGTGGAGGACAGCACGCGCTACGTCTTCGTTGGATTG

GGCCTGGGGCAGAGGCTCTACTCGGGCTGCAACCTTGTTGATTGCATACCGAGTTCGAGAGTCTCCGGAG

CCGCCGGATCCCAGCTGTGCAACAGCTGAGACCTGCCGCCAGGCTGTGTAGATCTTGTAAGCTGTGAGAC

CGAAAAGGGAAACCGCAGCCATCGTCGCCATTATTGGGTGTCGTTTCAGCCAAGAGGTGCATGAGTATGC

ACCCTCGACCAATTTGTCCCAACATTTGGTGATTTCGGGATGGCGGTCGCGGAATACCTTGTTCTTTCGT

CGCAGCCACTGCCGGAAGGATGACTCAGTCACCTGCTTAAGCTCTTCTACATGGTCAGAGTGCACATTGC

TGAGCACCGACCATTCGAAGCCAGTAAACAGGGAGGGAGGGACTTGGGCATTTTCGATCGCACGGGAAGG

GGCATGAAGGGAGAACCACTCGTCGTTTTCAACGGCGCGTTCACCGATATGGAGCACTATTCGGTCGATG

TTACCGTCAGCAAAAGCTACACCCAGAGCCTTAAGGGCTTCTGAGAGAGGACCGAGATCTGAAGGATCCC

AGTTACTATGCCAGAGTTGAAAAATGAACTCTGGTAGTGGGTCGCAGCTTCTGTATCGGGTACCACCGAT

GTCTAGAGTCCACTCGGTGGAGTCTTCTTCTTCTTCTACAGGATGATCAGCAGCCATTTGCTGCATCCTG

CGCATCGTTGCATCGGGGTTCTCGGGGGTGACGTCTTGATCGGGATCGTCCATTTGCAGGTCCTCCAGCT

TAATTTGCGCTTCAGGTGGTCGCGCATAATTGTGCTGGGTTGCGAGGAACTGCCAAAGACGGTGGGGAGT

GTAGTCCGGGTCGAAGTCAGGGTCAGGGGTCCCAGATTGGGCCGTGGGCGCTGGGGAAGCAGCGCGAGGA

CGGCCACGTCCAAGGGTCTGGGGGGGAGCGGGTTCGGGAGGAACAACGGAGGGATGGGGTTTGCGTTGGG

CCTTCACAGCATTCTGAAGGAATATCTGTGAGTCGCGGTACTTGCGGGAACAAGCGGCCACGTCAGATTC

AAACTCAGCCAAGGTCATCACTTTGATGACCTTGAGTGTGATTGGATCATTTTTCACCCATTCAAGATGA

GCAAAGTTCTCCATTTGTTCCTTGGTCTGGGCCGCAATCTTGGCATGGTCTACGAGGAGTCCTCCGCGAC

CATCTGCGATCTTGAAGCTCGATTTGAGGCGAGCATTCACGACGTAGTCGCGTCGTCTAAGCATTGCTTC

GGGGCACTTTTGCCCAGTCATGGCTGGCTTAGAGACGTTACTAGTGCAGATGATAGCTTTGGGCGCCCAC

CGCAGACCTTTGCGATTGATTTCGGCGAAGGGGGGGTAGAAGCCGTTGGGGTTCTTAGCGTCGAACATGG

TGGTGAGCTGGGGTTCTGAAACAGCGGAAGCAACTTGATTGAAGTCGTCGAGTCCGCATATCGGAGTGGA

AAGATCGAAAAGTCCACTTAGATATTGGTCAGTAGCAGATACCCAGAAAATGGGAGATTTGTCCTCATCC

CATTTTTCACCGAGAGCATCGAGAAGGCACCAGGCCATCCGTTCAGCAATCAGTGACTTACCAACACCAG

GTTCGTCGGCCCAGATGTAGAGGCAGAAGGGCTCGGGGAGTTGGCCTTGGAGGTGGGGGTACTGTTTGAG

GGTGAGGAACGCAAGTCCAACCTGTTTTTTGAGGCGGGCTAGGGTGGACTTGAGTTCACGGGGGATGTCG

TCGCAGTCGAGGTCCAGGGGCATGAGAATGTTGTTGGCGAGGATGACATCCTCTCTAACTCGTTTGTCGT

GTTTGAGATAGAAGAGGTTGTCACGGGCTGTGATGAATTCTGTGCGGGTGATGAAAGCGGTAGTGATTTC

CTTGCTCTGAGTAAGGTAGCGCAGGGATCGAACATGAGGGTCCAATCCGAACAAGTAATCACCAGCTTTC

TTGAAGAGTTTCCAGAAAAATTCGATGAATTTTCCTGCACTCTCCACACCACGCCACAGAAATGAAAGGT

CGCGTCCAATGTCTGATGTGCGTTTCAGGAAAGCCATGAATCCATTGCTGGATCCATGCTTCACGCCGGA

GATGTCTTTTGCGAGTCTGAAAATCTTTTGGGCTGTTGCTGTGTTCACCTCGGTGTCGCCACCTTGGCTT

ACCACCTCCTCTGAGTTTTCTTCGCGTAGAGGAGAGACGATATCTTGGAGTTTGATTATCAGATCGCTGG

CGTTGAGTTTGAATTCCTGGAATGAGTCGAGAATGGTGTTGGCGATGAGATGGACAACAGACCAGGCTGA

GGCATGAGCGATGATGTTGATGAGGTCGAGGGCTCGCTTTATGTAAAAGCTATCGGTGAAGGCGGAAGCT

CGGAGATGATCCGATACCACGCCGAACACGGACTTGCTTTCACCTAACGCGGCCTTAAGAAAATCCTGAG

CAGTTGTAGATGTTGAGGCAACTGCTGAGGAAATCTCGTTGATCTTGGTGGTGGTTTGGGCCATGCAGGT

TTCGGCGAGGTGGAGGGTTTGGCCGAGTCGAGAGATGGAGCGTTTGTACTCCTGCACGGCGGCATATCCT

ACAGCGGCAACTGCAGCTCCTGCCACTATAGGGGTGATTATGCCTTGAGAGTGAGGGGGGGGGGCAGCCA

AGCAAGTTCCGAGAGCGGAACCCGCAGTGATCATTGCCATGTGGATAGGTCCGGGATTGAGCTGGACATC

ACCGTCTTGGGTGACATCGTGCACAGGTCCGCGTCGGGCGAACAGGTGCATCACGCGGGTGGGCACTGGT

CGAGGGATCACCGGCTCGAACAGTGTAGTGTCTGGGAGCGGGGATGAAGCGGTACTGATTGGAGTGTCGG

GGATGAAAATGGCTTGGGGATCTTCAAAGCCTGCGAGTCCTGAAGGGAAGGTGGTCAAATCGGGGTAGTG

AATCTCATCGTAGTTGTTGGAAGCAACGACGCTGAGAACTCCTTTTTCAACAAAAAGGGGTTGTTTCCTG

AAGGGAAGGTCGGTCTGAGGGACCCAGGAGGAGTAGGGGAGGAGGCCAGTGTAGAGGTGAAAGCGGAAGT

CATCTCCTGCACGTTGGAAGACCTGGAGGTTATTGCTGTGAATGTTGGCAACCTTGTTGAGTTCCGATCC

AGACTTGTACGGGACAGGAACGGCGGCAAAGTCACAATAGACGCGGAGAACGCCATTTGCGACAGTCTCG

CCGGCTGCATCCAGGGTTCCTGCTTTGCGAAGACAGGTGCGGAGCATGGTGTACTGGGAATAGAAGGGGA

CAGTGACGGTGAGGAGATCGTTGACGGTTCCAGATGCAAGAAGGTAACCGCCGCTAGTGCAGGGAAGCTG

CATCTTCTGGGAGGGATCGGATTTAAAGAAAGCCGGGGAGGGGAAGATCTGCTTCTTACCATAAGAAGCA

GCTGCGGTGGGGAAATACACTACAACAACTCTAACGTTGCCGTAGGCACTCACTGCTCTGAAGGAGTAAG

TGAGTGACCCAGTCCAGAATTTGAAGAACTGGGCGCAAGCAGCAAGGGGGGTGTTGATTCCGGGGTAAGC

AGGGCACACAGGGAGGTCAAGCAGGGGCTGAAGGCGGTGTAGGGTTATTTGGGAGTAGAACGGAGGAGGA

TCAATGTCGAGAAGCGGCACTGATCGTTTGAGGATGTTCCCTAGTGAAGCGGGAGCATCCCCAAACCACA

AGTGGGCAGCAGCGCGAGCTGGAGGACTAACCGTCAGAGAAGCTGACGATCCAATATCGTGGGGAGTGAT

GTCTTTGGGAGGGCCAGCATCTTGAGGGGCGGACTCATCAGTTCCAACCTGGGCAGTGGGTTGTTGGTAG

GACTGTTCAGGGACGAAAGTAGGATGGAAGGGGACAGACATGGGACCGGCATTGACTCTACCGATACCAC

CAGGCAATTCCCAATTCTCCAAGTCTGAACTTGTTTCAGTCTGGAAGAAGAGGGAGGCGGAGGGCATGTC

AGGAACGGCCACTTCGAGGTCTTCGAGATGGAAGCTAACCAAGTAGTCAACTTCTTGGGCGACGGCGCTA

GGACAGACAAGTGGGTTTTGGACACTGATGATAAACTTGGCGTAGGGGGAATCTGGTTGTTCGGTTGATG

TGTCGAGACGGATGCCGAAAATTGCATTCGATTCAGCTGATCCATATCCGTATGCCAAGTTCTGGGTGAG

GATGGATTGGCGGCGGGTTGGAGCGAATGGGACAGGAAAGCGGAAGGGAATCCGAATGGTGAAAGATGAA

GTTTCACCAGACTTGTAGCTGAGCAGGGTCATCTGAGTTGGGTCAACCTTGTTGTCACCGTAAACGGCGC

CAGCAAGGAGACGAAACTGGTGAAGGAAGGTGGGGAAGAACTCTACTGTGTATACGAGAGTTGCGCGGAC

AAAGCGGTTAAGGACAAGCGGAAGCAAGAAGGGAGCGGTGTCTGGGTCGAGGTCAGCCCAGCGGAAGGTG

TGAGTGTAAATCACATCATCCACTTTGGCGGTTGTCTTGAGGCTGGAGGCGGCGATGATAACGGGGCGGG

CAGCGAGAAAGCCGAAGCTCATCTCGTCATCAAATGGCACTCGATTGTTCACATTCGAAAATGTGACGAA

ATCGAGAGCGATCTGGTCGGTGGGACGGGCGCCGCAAGCACGCTGGGGCTCAGGATTCTGATTGGGTTGC

ATGGGGACTGGGGGAAGGTGGGACTCAGGAGCATCAAGACCTGACAAGCTGGCAGATGGATTGCCAGACG

AGTCACCTCCTTTCACGGAGGCACCACTGGAGTCGCCGTAGAAGTTGGCTACAGTGGAGGAGACGTTTCC

TTGGGCAAGGGGTTGGCGGAGGGAAACCTGGAAGTCGTTGAAGTGGGTCCATACGGTGTATGGAACCTCT

TGAACGCCATCAGGAGGGCAGCGCAAGGGGACTCGGGTCCACACCATGAAGTATGAATCAAGGTGAGGCA

TGATGGTTTGGGGGGTCATGAGAGGGTTGGTGTACATGAGGACCATCTGGGTGAAAGGGATGTCGATTTC

GAGGGTGGTGCCACTGGTGAATGAAGCTGTGACACCTCGCATCTGGGAGAGTTGATCGAGTTCGAGAGTG

TAGTTGTCGGGTGTTGAATCTTCTGGAAGCCAGCAGAGGCCATAAGATCCTGTTTGAAACTGATTGGTGG

AGACCTGAACACGGATAGTGATTGAAGACCACTTCCAATAGAGATATTCAGGGACATGTTTGGCAAACAG

GTTAATGACGTCTGAGAGCTTCCAGGTGTTGTAACCACCTGGGGTATCAGTTGTTTTGAAGCTTTTGACC

GCGAGAATCTGGGGGCGGGCCATAGCTTTTG

>GAUE02014165.1 TSA: Anurida maritima s1072_L_1184_1_a_103_1_l_8391 transcribed RNA sequence/picornavirus

CTGATTATGAACTGTTTCATAGATTAGTTGAAGGAAGATATAAATCCCTTGATGAGCGGAATTCTTTCCT

TGCTAAGCAATTTAAGGGTTATGAGAAGGAGATTCTTCATAAGTTAGCTTTTGTGAATGATCATATAAGT

CACTTTAAGAAGTATGGATGTTGTGTAGATATCTTTAATAAATACATTACTAGTTTTGATAGGAAGGTTG

GCGAAAAGTTTTCCCATAGAGAAATGCGCGGTATTCGGTTTTTATTTAGTCATCTTATCGACTGTGAAAT

TAATAAGGAAAAATATTATCCTTGGTTTTGCGAAGGTGATATTGAACCGCAAATGAATGAAGAGAAACCT

TCATCTGGAGGTACAAAGACTTCTGATCAAACTAAAGAAGGTAATGTTGTTTTAACATCTGAAACAGCCG

TTGAAGTAGAAAAACCTATAGAGACTGTTGCCAAAGATTTTTGGAGTACTCGTAGTACTAATGATTGTGT

TGACAATTGCGATTTTACCACCAATCGTTGGATAACTATGGCTCAGTTTGATTGGACTAAGGATCATGCT

GTTGATACTGTTTTATTGAAAAAGTCTCTTCCGGTTGATTTTCTTAGTGCTAATGAGAATGCTGCTTCGG

CTATGATGTTTAAGCAACACGCCCTTTGGAAAGGTGATATAGAGGTTAGAGTACATGTTAATTCTGTCCC

CTTTCAAACTGGTATGTTAATAGCTAGTTGGTATTATGGTAGTTCTTTTGATAAATATACTGATAATAGG

GCTAATTTTGCTTCTATGGTTCAATTGCCTCATGTTTGATCCAGGCATCTACTGGAAATGAGGTAGAGTT

TGATATACCATTTAGGAGTTATAAGAGTTTCCTTAGTAATGTTAAGAGAGCGAATGATTCCGCTAATTTA

GATATGGGCAGATTACAGATTTCTGTTGCGAATGTGCTTAAAAATCCGGCGAACAAGGATGATGTAGTAT

CTGTAGTTGTATATATTAGATTTAAGAATAATTCTTTCACTGGTCTTATGCCCAGGAATTTAGTTGAAGC

TCAAATGCTTCCAATTAAGAAAGTAGTTAATTTAGCTAGTGATACTTTAGATATGTTGTATGATGATCCC

AATAGGGATAATCCGCCAATAGTTGCAGGTCCATCATGGATAACTCCTCAACCTGCCCAGAGTTGGGCTA

CTGGTACTGGAGATGTTGAGAATTTGGCTGTTATGAGATTGTGTCCAGCTGGACAAACACCTCACCCGCC

TGGTTCATCTACTAGATCTAATGAAACCAATATTAAGAGCTTATGTACTAAGTGGGGTTTGATAGAAACG

TTGAATTGGTCCAAAGATCAAGCTGTGGGGACAAAATTAAAATCTTATGAGGCGACTCCAATGATGTCTT

TTTCAACTTATAAAACTGTTAAGATCACCGATGAAGGGAAAGAACATACCTTGAGTATTTTACCTCCTGT

CGCAGTGGTTTCTAATATGTTTGCGTATTGGAGAGGACCGTTAGAAATTAGATTCGATTTTATCGCATCT

AAGTTTCAGTCTGGAGCACTCCTCGTTGCGTATGTCCCACGTAAGATGTTGGCCGATACTGACATTACTA

TTGAGAATTTAAAATCATGTTCTCATGCTTTGATTAATGTCAAATCCGATCATAGATATGTGTTCGAAAT

TCCATTTATTGCTGATAAGCCTTTTTGGCCTCGTCGGGAATATTTTGAAGGTCAGTCAACGCTTCTTAAT

GATCCTCCAGGCCATATTTATATATTGGTTTTAAATAAGTTGATAGCTAACGATAGTGTCCCCAATAATT

TGGACATTAATGTTTATGTTAGAGGTACTAATGACTTTGAGGTTGCAATTCCATGCATGCCGATGATAAG

TACATATAATGAATTACCAAATCCGGATGATGTCACTAATTTTATTAGACCACGAACTGGATATGGTTAT

TCAATGTATGTTGGTACGTGGCACTATGCCTATAAGGATAATAAGTATTATAACGTAGTTAGGTATGATA

CAGGATCTGATCACATAACCCAATTTGTTGGTAAAGTGGGAAGAAATGTATATAAGCCGGCTGATGGTTC

GTCTAAGATTTATGGATATATTAAGGACAACAATACTACTCCTGCAGAAATGAAATATTTCTGTAGATTA

ATAGTTAATGGTGATGGTAATACCTATTTTTATATGGCTGCGTTTTCCACATTGGTAGCTGCTGCAACTT

ACTGCAGAGAATGGAGAAGGAAAGGGTCGCGGTATGCTGATAGTCCATTACAATTCTTAGAAGAAAATGG

AGGATCTGGAGGTCCTTATGTTAAAAAACCCGAGACCATTAAATTTGAATATGTTGAGGTTGAAGGTTTG

CCAAAAGATGCGGAGACTGGTCCAACCTCGTTGGAAGGACAAATGGATAATGAAAATGAGACCCGAGTTA

AGGTTGCATGTGGATCATGTTTGGATACTAAGGAGGGTTTGGTTACCTTTGGTGAGAGATTTACAGATTT

AAAAGATCTTATGCGTAGGTATCAGACATATGCAGTTGAATCTCATCAATTTAGCAAAACAGATTTGCTA

AATGTTTGTACTTATCAAATGGTTGTCCCAATACTTCCTATAGGTCCAACGACTAAAGATGGAACTAATT

TAAACAAAAATACGAAAGGTGGTCTTTTGCCGCTTTTGTTAAGTGGGTTTAGATTTTTTAGAGGTGGCCT

TAGATTTAGAATCGTTATTTTAAGTGCTAATCCCATTCAGGGGTTTATACAGCATCGACCTGATTATCAA

ATTAAGGACCGAGCAGTAGAATATCCGAGAGCAACTTCAGCTGATCCTAAGCATCTAGTTTCATCTGGAT

ATGCAAAGCATTTCTTTAATACAGCAATTAATACTGTTGTAACTATTGAGATTCCTTTTTATAATCCAAC

TAATTTAGGTTTGCTCCAGGCTCCAAATTTTGCTAACAAGTCCGAGGTTGTTCAGTACGGTCTTGGTATA

TTGGGTTTCAATTTTCTCACCACTGTCAATTCTAACGTTACTGTTATAATGGACTATTGTGTTGCTGATG

ATTTCAAAGCGAGTTGCTTTGTTGGTTTTTCACCAGTTAAATCAATAAAAGCTTCATCGACCTTTGAACA

GATTGGACCTGTTCGCCGAGATTCTGCAGAATATGAGGTTATAGAACCTCAAGTTGGAGAAAGAGTTTGG

ACAAAGATTAAGGAGTCCACCAGAGGTGGTGTTAGTGAAGTCGTCGAAGTTGAACGGCCTAAATTTGCAA

AAATTGTCACTGATGCATTGCACCAATATGCTGGGGATTGTTCTGCTGAAGTTAAGCAAGTTCTTATGTC

TTTGTTTGGTCAGTTAATTCAAGTCGTTGCTAATCCTGATTGGACTACTATAGTTATAGCATTAATTACT

ATTTTTTGTCAAATAGGTATTTTTAGTTGGGATCACTTTGCTAAGTTAAAAGAAGCCTTTTGTAAGGTTT

ATAGTTATTTTTTTAAGAAAAATCGTGTTGAAGAAGTAATACAAGCTCAAGCCTCTTGCACGGAGGAAAG

GGAAGGAGTGCAGGAAGGGATTGAAGCTCAGATGTCTATATCTGAGATTCCTGGTTTTCTATCGACTGTA

GTTGGTGCAGTCTGTGGAGGCATGGGCTTGCGTTATAGTGTGAAGAAGTTTGGAGAAATTCCGAATTTCG

GTAAGACTATCTTTGATACGACGAGAGGAATAAACGTGTTACACACGTTTTTTAATAATAATATAAAGCT

AATTGAAAAAGCCTATAGTTGGGTCTGTAATAAGGTTATGGGAGATAACCTTTTTGAGAATGTTCTGGCA

ACCGAGGGTGAGAAGATTCGCCAGTGGTATGATGATTGTGTTGAAATCCTAGATCCTAATAATGAGGAAA

GACTCAAGACTGATCTGGCGTTGATTTGTAAAGTTTATTGTTGCGCAATTATTGCGATTAGCATAAAGAA

GAAGTATGCTACTGGAACAACGTCAAAAGAGGAACAACAATCTAAGTTTGCTCTGTTCCATATGTGCAAT

AAAATTATTGAGAAGCGAGACCAAATTGTTTATAACAAATTGGCTCCGCCAGTCAGCTTTGAACCGTTTG

TCATTTGTCAATATGGAGATCCTGGAATAGGTAAAACTCATATTGGCGAAGCTGTTGCTCTGAAATGTTT

AGAAGAAATAAATTATCAGACAACAGGTGAACTGATATTTACCAAAACACCTGGTAATCAATATTGGAAT

GGTTGTCGAAATCAACCCATTTGTATGTTTGATGACTTTTTGGCTATTAGAACAGCTCCATTTTCCATCA

CTGATATATCAGAATTGATGCTTTTGAAATCTAAGGCTGTGTTTAATCCGGCTCAAGCATCAATTGAGGA

GAAGTATATTAGATATAATCCTCTTATTGTGATGATGAATATGAATCAGGCTTTTCCAAATATTGACGGA

TTACTTGATCAGCGTGCTTGGATGAGAAGGAGAGACCTTTTAGTTCATGTCAAGTCTCAGTATGGGCGAA

TTGCTAACGTTCCTCAGGAGTTGTTAGCTGATAATGCCCATTTGAGATTCGACATTTATGTAGACTCTTC

TAATCCTAGGGCTCACCCTATACAACAAGATTTGACGTATGGGGAGTATATTGAGTTAGTTCGAGAATCT

TTTTCTGCTTATTACAGTAACGAAGCCGAGCAATATTTGCAACGTATTCAGCGATTTGAACAATTTCGTC

CTAAAATGGATGCATCGATAGGTGATACGTTTGCTTATTATGTTGACTTTGTTAATAAGTTAAATTTAGA

ACAATTAACTGTAGAAGAACAAGCAGAATTAGCTAGGTTTAAGGTTGAAGCTACTAGATTAGGTGATCCA

GAACATCCAACTGTTAGTAAGATCAAGAAAACAGTTGAGGCTTTCCTGAGTTGCGCTCATACATCTGAGG

CATCATTGTTGGCTAGTAATGTTGGAGCCAATCCTCAAAATACCACTCATGATATGGCTAAGAGAGTTGA

ACCCCAAGGTATAGGAGCGTGCATATATGCACCTAGTGGTCGTAAAGTTCCTTGGAATGAGTTTGTTGTT

CGGCGAGCTAGAGAACAAGTTAATAGGAAATTGAGAGACCGTAATGATGGTGTCCTCCCAACTGTAACTC

TTACTCCAGCAGTTAGCGCTACTTGTGTCCATCAGTTCTTTAAACCGAGCCTCGTTTATACATCAAAGCC

GGCAATTACAGCATCTCAGTTGGGGATGGTTGATGCGCCGCAATATGCAGACAAACCAGCTGGTTTTTAT

AACTTTGCTGAAGTTATGTTTGAAGATGAGGCTTTAGTTTATATAAAAGACGATTTTTGTGGCCCTTTAA

AAGCAGATTGTGTTTGGTCACACCGAGCATATTACGAACGAGATATGGAAAATTGGTTGGCCGAAGATGG

TTTGTTGAGAGAGTCCGAAGATTTGCCGAGGTATTTTAAGAAACACCGAGGAAAGAAAAATGAGAAACAA

TACCTCAAGGACGTTAAGAAGGCGTTGACTAAGGAAGTTGAGGAAGGCATTATTAAGAAGAGCCTTGGCG

TGGTTGGAGCGACAATTCGAAAGTTTTTTCAAGTTATTAAACCTTTTTTGAAGTTTGTGGGTTGTTTACT

AGGTCTTTTTGGACTTGGTTGGGCAGCCAAGAAAGGCTATGGAAAAGCTAAAGATTTTGTTGATACGTCT

ATTCAACCAAGATACAATGAATGGCGTCAGGATGTTGATGAGAAGGGTGGTTTTAGAGCCTGGGCAAAGG

ACAAGGTTCGAGCTCCCGAGTGGTTTAGGAAGAAGGAACCAGTTGACAGTGAGATGGCTTATGATGAGGC

AGTTCCCAAATTGAAAGAACATCGAATGCTTCGACCAAGAGGTAGAGATGAAGTTAAGGCGCAAATGGCT

GCTGAACAACAGAGCGTTATTATAACCAAGGTTAAGGAAAACACTGGTTTTATTATTTGTCGCAATAGAA

TAACTAAACAGGTAATGAATTTTTGCCGAATTTTAGGAATTTGTGGCAGATATGCATTAATGATTGACCA

TTATAGTCATTATTTTGCTCATAACGCTAAAGATATAACTCTTGAATTAGTTCATAAAGGAGTTATTCAG

CCATTTACAGTTGGAGAGCTGAAGATCCAGAGGTTTGAGGAAAGTGCCTTATTTGTAGTTAGATTTCCTC

CACGTTTTGCGATGTTTAAAGACATTCGCCACTTTTTTATGACTCAGCAGCAGTCTTTAAATGTTCCAAC

AGAAGCATTTTTGCTGGAGTTTGAGTGTCCTGATAATGTTATAGAAGATCTTAAATGCGTTATTCACCGG

GAAGATATCACTGTTCGTGATTATATTACTATTCCTAGTGATTACGCGGAGTTTTCAGACACCACTGTTG

CTAAAGTGGCGGCTTATCGCACTAATGCGAGAGGTATGTGTGGTTCCGTTCTTGTAGCCGATGTTAATAC

ACCGAGTCCTATTATTGGTTTACATGTCGCTGGTGTTAAAGACGGCAACAAAGGATTTGCCGAGCTATTC

CTGCAAGAGACAATTAGAAATGTTACTCAAGATACTATTGTTAGGGAGGCTGAACCGATTACGCCTCAAT

GCAAAGATCCTGAGTTAGCATCTTTGAGTTTACCAGCTAATGTGATCAATGTTTGTGCCGTTGAGAAGGA

AAGATCACATCGTGTTCCTGGCACAACAAAAATTCAGAAATCTGTTTGTCACAACGAGATTTTTGACGCT

ACTCATGAACCTCCTGTTCTCCATGGAAAGGATAAGCGTTTGAAGGAAGCCGGATTAAAGTTCTCTCCGA

TTATAGAGAGTTGTAAGTACCACGGCGAACTCTTTGGAGTTATGCCGTTGGAGGTTGTCCGAGAAGCTGT

TCACGACTATAAGTCGTTGATGCTATCGGCAAGACCTGTGAGAACTAATCCTGGTGTTCTTTCCATAGAA

CAAGGAATTATGGGTATTAAGGACCTAGAAGGCTATGAGATGATGGAGATGGATACCTCCGAGGGATTTG

GCTTCAGACATTTAAGGCCAACTGGAGCTATGGATAAGAGATGGCTTTTTGAGATTGAACAGACACCATC

TAGTATGGAGTTAAAGTCGGTTAATGAAGGTCTCTTGAAAGAAATGGAGAGAAAGAACNTATTGTTCCAC

ACACTGTCTTTATTGACTGTTTGAAAGATCAAAAACTTCCAAAAGAAAAAGTTTTAAAGCCTGGAAAAAC

AAGGGTGTTTTCAATGAGCCCTGTCGATTTTACAATTCAATTTCGACAATATTTTCTGGACTTTTTAGCT

GCTTTTAATAAAGGTAGACTTGATCTTGAGCACACTCTTGGCATAAATGCGGAGAGTCCTGAGTGGCACG

ATCTTGCGTTGCGTCTTTTGGATATGTCACCATGTATTGTTACCGGTGACTATTCAAAGTTTGGACCGAC

GCTACTCGCTCAGTTGATTGTCGAGTGTGGTCAGATAATGATTGACTGGTACGAAAAGTATTCCGATCAA

ACACCAGAGGAAAAACGTATTCGAAAGATCATTATAGAGGAGATCATCCATAGCGAACATCTTATGGTTG

ATCTCATTTATGAGACTTTGTGTGGAGCACCTTCCGGAACAGCAATGACGGCTGCTCTCAATTCTATGAT

TAATAGCCTGTACATTCGTTGTGCTTGGTTGTTGATCTTTGAGATGGGTTTGCGCCGTATACCCGGAATT

GAAGAATACCTTGGCATGTCTGCATTCCATCGATATGTCAGGATGTTTTCGAATGGAGATGATCTAATTA

TGGCAGTTAAACGGATCATCGTTGATTATTTTAATGCTGCTACTATTGGTGAGGTATTAGCTACTGTTAA

CATTGTGTTCACGGATGCTAAGAAGAGTGGCACTGTCATCAATTATGCCACACTTTTCTCTGAAGATACA

ACTTATCTGAAGAGGAATTTTATTATACATCCTCACCGGCATTTAATAATTGCCAAATTAGATCAGCGAG

CGGTCCAAGAGACATGTAACTGGGTTATGAAGAGTAGAATCCCAGTGGAGATGGCTCAACAAGCTTGTCT

TGCCATGATGCTCAATGGTTTTGGCCTTGGAGAGGAATGGTATGAGAAAACTCGAGCCCGAGTTCAGGAA

TTTTGGAATGAAAGAAGAGTCCATCTTGTAATCCCGAGCTGGTTCGAAGAAGACCTGAGACTTTACGGAA

GTGGAGTTGCCCTACATTAATGCGTTTTAGCATTCCGGAGGGACACAGGCGGCCCGGCGCCCAAATTGCT

TGTGGTTGTTGCTCAGAAAGTGAGCACCCACTTGCGCTAGGTTAGTATAAGCAATCCAAAA

>GAXI02037733.1 TSA: Tetrodontophora bielanensis s2383_L_2112_0_a_74_4_l_5471 transcribed RNA sequence/picornavirus

AGAAATTAAATCTGGTATTTTTTCGCATGTTTATGAAATGATAAAAGATTTTGCATCTGATAAAAAACAA

GTAATTTTAAATGCTATTACTAATTTAATACATGTTATTTTAAATCCTAATATGAAAACTTTCTGTGTTA

GTTTAGTTTCATTTTTATCTTCTTTAGGGTTAATCTGTTATAAAATGGTTGATAAGATGGTTACTTCTTG

CACCAATTTTTTCCCTGCATTGTTCCGGGCCTCGGCGAGTGAAACCGAAGGAGCCGCTAGAATTCAGCCT

GAAGGCGACGACGATCCGTTGCTCAATGAAAAGTCTGCATTCTTGTCCACGCTTATAAGTGGAGTGGCCG

GAGCTTTGCAATTAAAAGTTGCGTGGGGTAAAGTAAAATCTATTCCCGATTTTTGTAGTGGATTACTCTC

TGGTATTAAAAATTTTTCTATGTGTGCTAATTCTTTATGTCAGTTTTTTAAAAATAATCTCTTAATGGTT

CAACGAATGTTTAATTGGGTGTTAAGGTATTTTGGAAAAGGTAATGCTATAGATAGCATGATAGCTTGTG

ATCAAGTTATGTTTTCAAAGTGGGTTGATGACTGTCAAACGTTGTTGAACCCATCTTTTAAAGAACGAAT

GAAAACGGATAGTGGTATGATCATGAAGGTTTACTGTTGTGCAGTTTTAGCACAAGCTATTATGCGTAAA

TTTGTATTGGCAGAAAATTTAGATCCAAAAATGACTAAATCTAAATATATGACGTTGAACTTGTGTAGAG

ATATTATTAAGAAAAGAGATGAATTAATACAAAATAAGTATTCACCTCCTGTGTCTTTTGAACCTTTTGT

TGTATGTCAAGTTGGGGAAGGAGGAATAGGTAAAACTCATATCACTAATGAAACTTCTACGAAGATGCTA

AAGTTTATTAATTATAAGTGTGATGGAGAGGTTATGTTTACAAGAACTCCTAATAATCCATATTGGAATG

GCTGTTTGGCCCAACCTATTTGTGTTTATGATGATTTCTTGCAGATTGAAGCTCCTCCAGCTTCTCATAA

TGATATTGGAGAGTTAATGATATTGAAATCTAAAGCTGTATTCAACCCTCCCCAAGCTGCAATTTTGGAT

AAATGTGTTAGATATAATCCTTTAATAGTATTTTTAAATATGAATAAGGCTTTTCCTAAAATAGATGGCT

TAGCGAATGAAGAGTCGTGGATGCGTAGAAGAGATGTATTAGTGTCTGTGAGAGTTAGAGATCAGTATCA

AGGAATGACTGCTAATGAGTTACCTGATGACATTAAATTGAATAATAAACATTTGTTGTTTAATATCCAT

AAATATTCCAGGAAGGAAACTGCTGATATGGTGCATGTAAATCTTAGCTTTCCGCAGTACATAGATTACC

TGAATGTACAATTTGCGAAGTATTATAGCGTTGAAAGTAAGATGTATTTGCAACGTTGTGCAGAATTAGC

TCAATTTTCATTTGATGAGACTGAGGATTTGTCTGTTATTTTAAAAGAGTATAGTGATTATATAGATAAG

GTAAATTTTAGACAGTTGAATGCACAAGATAAAGAGAGTATAAATGGTTTCTATTCTAATTCTAAATCTG

CTATAAAAGAAATTATGAAAGGTGTAGTTGATAGTTGTCATAGTGTTTCTGATAGATTTACTAAGTTCTG

TATGTGGGATAAAGATAGTTCTTCAAATATTACTAGACAAATGAAAGCCTTTTCAGTTCAAGCTGAAGGA

GGTTATAGTTTGGCTACCCCTTATAAGACTGTTAGAGAGGAATTTAGAGATATTGAAAATTGTGCTCTAT

TTGATATGAAGGATCATTATTTTGACTATATAGCGGCCCAGGATCATGTTGACATATCTAATAAATCTTG

CAAAAAGAGATTACCACCTTTTAGATGTCATGTGGTTATGGTAGATAAAGTTCAGTCTGTTTTATTGTTG

TGTATGCATCATTCTACAAAGAGGAAAGTTCCTTATACTTTATTTTTTTCTAAGAAAACTAACGTATATA

GAGCGGAAGATAGCAATGAGAATCGAGATGAAGGTGAATCTTTTACTCAATTTGAAGAAGTTAAAGGTTT

TCCAGTTCGTGATAATGTTGATCCCCCTTCATCTGATGAAAGTTCTAAATCTGATAGCGAAATTGAGAGT

GATGAATTAAAACAATCTGATGAATGTTTACATAAAAGATTTGATCCTGAAATGTCTTATCATGTCAAAT

TGAGAGAGTTTTTGATTGCTTGTAGTTCTAATAAAGATGCTCCCTCTTTGTTACCCCCTGAAGAGTTTTC

TGGCTTTATTTTGGCTGATAATATAGATGAAGATACTAGCTTGTATATACCTTTGACTGAATGTGATGCT

CCTACTTGTGTGTGGAAAAGAAAATTTAAAGATGTTAAAAAAGGAACCTATACTACTCAGAAGGACATAG

ATATGGCCAAATGGCGAATTGCAAATATGCATTTTTTTACGTCTAAGAAAATGGATTGGACAGCTGTTCC

AAGCTATTTTAAGACTGATGTTAAAAATATTAAATTGATTAATAAGGCAGTTCTAGATTTGTGTACTCCT

GAGGCTAGAAATGAAGCTGAAGAAAGTTGGTTTGGTAAAATCGGGAAATGGTTGAAATGGATTTTAGGAA

AAGCTTTATGGATAGTCGGTAGTATTATAGGTACTTATTTGGTTACCGTTGGGTTCTTTAAATTGGATGA

ATACATGTTTGAAAAGCATAGTAAGTATAGTTATACGCAGAGTGCCGGTGAAGCTGTTGCTCGCATTCAA

AATCCAAAAAGTGTTGCAGAAATGGCTTATGATCAAGCTGTTCCCAGACAACATAGAAGAGCTATCTTGC

CTATGAAAGTCGTTAAAAATCTAGCTATTCAAAACGAAATGGCAACAGAGCAAGAAAATGTTATTATAAC

AAAGCTCAAGAGAAATACTTTTTGGATTTCGAGTGTTACGAGTGAAGGTAAAATAAGGAGTATGCGTTGT

TTAGGATTGCGAGGTCACTATTTTATTATGCTTGATCATTATCACCAATCTAATCTTTTGGTTCTTAAAA

ATTCTACTTTCTTTTTTCAAGCTAAGAATGCTAGATATCAGTTTCATTATGATGAGATTAAGTTTAAAGC

TGTTTCAGATAGTACTATATTGATTTGTAAATTGCCTCCTCAAATGTCTTTGTTTAAAGATATTGTTAGT

TTGTTTATGCGTCAGGAACAGTCTGCAGTGATACCAGCTCGAGGATTTTTATACGAAATTCTACCGCCTG

CAGATTTGTACGATGAAGTTTCTGTTGTTAAACACGTTTTAAATCTAAGTGTTAGAGACAATTTACAAAT

TCCCTCTGAATTTGAACAGTTTGAACCTACAATTATAGAGAAAGTGATAACTTATGGTGAGTCTAGAAAA

GGTTTGTGTGGTTCTGTTTTAGTTGGAGATACTAATTCTCCTAATCCTATATTAGGTATACATGTAGCTG

GTTTGAAAGGTGGGGGTTGTGGCTTCGCGGAGGCGATTATCCAGGAGACTTTAGATCCTTATACCACAAG

TTTTGTTTCTGCTGGCGATAATATTTGGCCGGAGAGTGGTTCTGTTAAAGATGCTAAATTAATGATCTAT

TCTATGGTAGATCCTCTTTTCACTGTGCCGAATAGACTTGCCCATCAGTCTCCTGGAAAATCTAAACTCG

AAAAGAGTGAATGTTATGAGTTAATGGGTGAAAGTAATTTTGATATACCTGTTTTGCATAAGAATGATCC

TAGAATAAAAGATAATCCTTTTTCTCCTATAGCTGTTGGTTGTTCGTATCATGGTCATATTCCTACTCCT

TTTCCTGATTCTATTTTTAAGCCTGCTTTGGAAGATGTTCGCGAAATGATTTTTAGCACTGTAAAACCCG

TGCGTGCTAATGTTGGTGTTTTATCTGAAGAGGCGGCCGTATGTGGCTTAGAAATAGAAGGCTATGATGC

GTTAGACTTTTCTAAATCTGAAGGCTTTCCATATATAGCAAGTAGACCTAAGAATGCAAGTAATAAAAGG

TGGTTGTTTGATTTGGATGAAAAGTGTGGCGTAAAATTGAATAATCTCCATCCTATGCTAAGGAAAGTTA

TGGATGATAAGCACGAAAAGCGTTTGAATGGGGAGATACCATTGACTATTTTTACTAATTGTTTAAAGGA

CCAAAAATTAAAAAAGAGTAAAGTTCTTAAACCGGGAAAAACTCGGATATTTGGTATTTGTCCTGTAGAC

TATTCTATACAATCTCGTCAATATTATTTAGATTTTTTGGTTTCTTATCAGTCTGCTAGGTTTTCAGCCG

AACATGCGATAGGAATTAATAAAGATAGTATAGAATGGACTCAAATGATTAGAGAATTGTTAAACGTATC

TCCGTGTATAGTCACTGGAGATTATTCAAAATTTGGACCTACACTTATGAGTAAATGCGTTAATGCTGCT

TTTGATATTATGATTGACTGGTATGATTTGCATGGTGATAAAAGTTTAGTTAATAAAAAAGTTAGAGAGT

GTATGAAGTATGAACATATGCATGCACATCATTTAATGTTAAATTTAGTATATCAAACTTTTTGTGGTCA

ACCGTGTGGGACAACTTTTACGTCCATTTTGAATGATTTAGTTAACAAAATGTATATTAGGTGTGCATGG

CAGTATATTTTCAATCCTAAGAGAATTAGCGGTAAAATTGATGGCGAATTTATTTCTATGGCTTCATTTA

GATCAAGAGTTAAATTAATAACATATGGTGATGACTTGATTATGGCTGTAGATGGTAAAATTATAAAAGT

TTTTAATGCTAAGACTATTGGTGAATTTCTTAGTATGTTTAATATTGTTTTTACCGATAGTTTAAAGAGT

GAAAATATAGTGCCATATGCCAGTATTTTTAGTGAGTCGACCACGTTTTTAAAGTCGACGACGAAGTTAC

ATCCTTCGCGAAAAGGTGTATTTATTCATCAATTAGATCCGATGGTTGTGAATGAAACTTGTAATTGGAT

AATGAAATCACGTAACTCTGTAGAGATGTCTATTCAGAGTTGTCAGGCGATGATGCTTAATGCGTTTGGT

CTTGGCAAGGATGAATATACGGAGATGAGAGGAAAAGTGTTAAAATTTTGGAGTGATAGAAATGTAAGTA

TTGTCATTCCTAGTTGGTTAGATGAAGATGAACGAATGTTTTATGATGGTGTGAATGATTATGATAAGCC

GTTCTTTAGTTCGCTTCTTAATTATAGTAGTAATTTGGTGAAGGCTGCGAAAGCAATTAATTTAGATAAT

TAGTTATGATCCGCATCTTTCTCAAAACTCCTTTGGTTTTACCCACGTTAGAAATCCATCTGTTTCTCAA

TGAGTAGCGCAAAATGCTCAGAGAGGTAACACGGCGTGGGGTTTATTGGATCCAAAAAAAAAAAAAAAAA

AAAAAAAAAAA

>GAYN02051149.1 TSA: Campodea augens C259544_a_54_0_l_2753 transcribed RNA sequence/picornavirus

GATATCACCAAACACTTTATTGTGGATGAAGATCTTCAGTTTTTGGCAAATACTGAGGCTACTCTCTGCT

CTATTTCTGTGTCTGTTGATACATACTTTTATCAGTGTCTTCCTGTGCGTTCAGTTTTGATGTCTGATCT

CCGCTATAGGCATGATGGTGAAACTATTCATGTCGCTAGAGGTTGGCAGTATTCTGCGAACACACAGCGA

GGGCACTGTGGATCTATTCTTTTAGCTTGTAATCCTGCTCTTCCTCGTAAAATTCTTGGTTTTCATATAG

CTGGCGCTCCTTCACAGAGTGTTGGTTATAGTACTTTGTGTACACAAACTGATTTATTACATCTTAATGA

TACAGTGGTGGCTCCTGAAATTCCAACTGTTGTTGATACCGATGTGCCTCCCCACATTGAAGCTCAAGGG

TTTTTAACACCCGAAGGCCGTGTGGAGAAACACAAAGGGTTCTTTTCACCAACGAAGACCACTATCTGTC

CCTCTCCTTTATTTGATCGCTTCTTTGCTCATACTTCTCTCCCTGCTAATTTGGCTGGTACTCTTCAATT

CGTGCCTCTCCAAAAAGGTTTGGAGAAATTTGGCAATCCTGGTAAAGCTTTTCCTATTTCTCAACTCTAT

TGTATAACAGACTATCTCACCTCTTTATTTGCGGCATTCCCTGGTACGGGGGTTTTAACGGAAACCGAAG

CTATCAATGGTTCTCCTTCAGTTCCTGATATGCAAGGTATTCCTATGCAAACCTCTCCCGGTTGGCCATT

CTCATTAGATGTACCTCCCGGGGCCCACGGAAAGGCGTTTTTATTTGAGGAAAAGGATGGTGTGTTTACT

GTTTCTTCTCCTGTGTTGCGCAAAGCTTTAGATGATCGTCTTGAAAAAGCTCTTCAGGGTGAACGCGTGT

TTAGTGTGTGGTCTTGTTGCACAAAAGATGAACGTCGCCCAATTGAAAAGGTCAATAAAGTTCGCGTATT

TGAAATTCCTCCTGTTGATTTTACTATTCTCTTTAAGTGTTATTTCGGTGCATTTTCTGATATGTTTTTG

AGAACGAGATTGCAGACATTTTCAGCTGTCGGCATTAATGTGGAAAGTGGTGAATGGAACCGTCTTTATA

CTCAACTCAGACAGAAAGGTGATCATGGCTTTGACGTTGACTTCCATTGTTTCGATGGTATAATAAACGC

CTCTGTCATGAACACAGTTCTGTATGTTATTGAAGATTGGTACCAATACAACGATCCCTCTTGTACTTCT

GAACATACTAGAATTCGTGCTGTTCTTTTTGATGAACTTGTACATACAATTCTTCTTGCCGGGGATGATG

TTTATTGGAAACATACTGGTAATCCTTCTGGCAACCCTTTTACTACTATAATTAATACTATTGCTCAAAT

GGTTCTATGGCTTAATGCCTGGTTGTCTCTCGCGACCAGAGAAGATCCCTGCCTAGCCTATTGTCCCATG

TTTTTTGAACACGTGGGTTGTTATTTATACGGCGATGACGGGATTTTCACTGTTAGTGTGCGGACTCCCT

GGTTTAATGGCCAGAACTGTTGTAATTACTTTACTTCTTTTGGTATAACTGTAACACCAGGTGATAAGAC

GGGAGAGTTTTCTCTTATTCCTTTAAATGATTTAAAATTTTTAAAATTAACTTTTGTTCCTTATGGTGCT

ATGATGCTTGGAGCTTTGGATAAATCAGTTATCTACGAACTTTTTAACTGGGTGCGAAAGTCTTACGACG

CACCATCCCTAGCGGCCGATAACATAGTTACCGCTTTACGAAGATCATGGTATTGGGGCCAAGAATTCTA

CGACGACATGAGACCAAGATGTTCTGCTGCTTTGCGGGCTTTACGAGTCTTTCGCTATCTGATGACTTAT

GAAGAACAAGCTCGCTCACTGTACGATGCTGTGCAGTGTCAAGGCGGAACTGCGGTGGTCTTGTACCGTC

CGTGTGTTCACGGCTCCTTCTGTGATTCTTTTACTAATCATGCTGATGCTTTAGGTCTTAGCCGGCCAGA

AGCTAGGCAATATCCTTCTATTTTTAGTTTAATGAATGTTTTTGACTGCACTGTTGATGAAGTGCCTAGT

ATCTTGTTATTCAAGCTTCGCGAAGTTGGTATGTCGAAGAATCCTGTATGGGATCATGACGAGATGGAAT

CGGTTCATAACGCCGTTGTCCACGTGAATTGTGTTAGTGCTAATCTTAGTTTCGTCCATCCTTTGGTCGC

TTTAAGCGCTTTTCATACCTTGCCTCGGTCTCCAGCAGAGGCTCGGTTTCTTACACCAAAAGACGTTTTT

AGTGTGCCATGGGTGATGAATGCTACAGCTTTTTGTTCACTTACGCATCCCATGTGTACGGCGGGCAGGT

TTTTAGTAACTGATTTCGCCAAACTCATGATACATCCCATATATTTTTCTTCTCCAAACTTGCGTCGCGG

GCTTGACTTTTGGCGTGTTACACCTAACCATTATCAAGTTGCTCTCGCGCAGACTTCTACTGCAGTCTTA

CAGTTTTTAAAATCTTTCAGAGAAGCAGTTGGTTACCCGACTGATATCTCTGCTTCTGTAGATTAATTTC

TTTTAATTTTTCTCTCCACCTTTCACCTCTAGTTGATGTTTTAGTATCTTCTTATTTTCGGGTATTTACG

TCGGACGGGTTAGCAACCCTGTCGTTCCTCTTCGTGTTCTTTTCTTATTGGACTCCGGCGTAAATATCTT

TCTTTCGCTTTTGCATTAATTAA

>GAYJ02032904.1 TSA: Atelura formicaria C263383_a_42_0_l_2190 transcribed RNA sequence/tombus-like virus

GGGCTTCTGGCTCAATAACTTGGCCCTCCACGGGCGCATTGGCGGCCATTTTGGCGAATAAAATTCACAA

TATTATCTCTCTCTTTATTCACTCAAATTCTCTCTTGAGATGCAGCAGGGCGGGATTTCATTAGGCTCCC

GCCTACCCCAATTATAGATTTATGAGCGGTTCCGCAGGGTGCTGTGCTTTTGCGGATACCGTGCCCCCTC

ACCAAGGGAGAATGTTCCATTTACATTGCCAAAGTCGTCTTCCTTTCATGATTTACAAGTACATATCGAG

TGGCCACAAGATGAGCTGGGGGATTCTGTGTACTGATCGGGATTTGTACATCTAGTATCTTCCGGATCCT

TCTTACCATTTCTTCACCACAATCAAGGGCAGAGTCATAATTTGGGTAAGCTTGATACACAGACATGGTG

GGTTCCCCACTAGCTATGTTCCATTTTCCATCGCTCAGGTCATGTTTAAGTGCTGCTGTTGTGTATTCAT

CTTCACAACCTATCTCGCCGTTTACTATCTTTGAGAAGGTTTCTTCTGCATTGAACTTCGCTATAGTTCG

CTCAAAGATACCTTTCCACCACGCACCAAGAGGATTTTTCCTATTCCAGGCTATATTACATATAATTACA

TTCTTCAAATACTCTTGGTACGGAATGGAGGTTTGATACGTCATTCCAAACATGTCTACACTGCGTTGGG

GGTCAAATAGCAGTGTGGTTTCACCCTCAGCAGTCACAATCAATTTTGTTCGACAATATTCAACCGTATG

TATATTCTGAGTGGACGGTTTCAATTTGGACTCCTGGTTAAACATCTTCATTAGTCGTTCGCACTGCTGA

ATGGGGATTGGTACATTAGTAAAAATTATGCTATCATCGCCATTGACTATAACATCTCCCTGGATGCCTA

ACATTTCCAACACCTGACGGATTATAGCATAATTTAGTATACTGTTCCCCAAACTGGTATCAACATCACC

GGACATGCGTGTTCCACGTATCGTATGTCTACCTCCATCTCTGAGATAGCAACGGTTTCTTATGGTTTTC

CTACTGAGTGTTCGCAGCCGTCGATCATGGTTGTAACAAGATTGGTAAAACTTGTGAGAAACACGCAACA

TCTCCACAGTAACATGCGCATCCCACGAAGTATGATCAAGCTCAGTGAAGTAGCGATACTTTTTACGGAG

CTTGTTAATATTGGCAGAAACTTGATCGACTGTTCCTTTGCCAAAATGGTACCCCTCTCTATGGCTGTGA

AAGAGGAGCATTTCAAGTGGTTTAAGGTATCTCCCATACTCAATGTTAAATGATGGATGTCTGGCCTGTA

TGCATCTGGGAGCTTTATACTTGGACATGTTAAACTTCTCCAGCTTTGTAAAAGGCTTGATTGTGCTAGA

GATTTTCCCAGTCTCTTCCATGCGTTCAAGGACATCCCGGTAGAATTGTCTTTTGCTGCTCGTAGTATGT

GAAGAGCAGTACTCATCCCGGGTCCATACTCTTGGGTGACATAAGAATCGTCGTATATGTCGGTAGATGC

CTGGAATGAGACGGGGCTGATATTCAGGCAATATGACGGGCTCGTGACGGTTGGCGTATGAGGCAATTGT

TGTGTCAGTGCCGCTGGGGTAATAGTAGTGCAGCTTCGGCAAAACAGGGCCGAAATGAGGTGTTGTGCTG

CAAGGGCGCCAGCCCCAATCGCGATACCGTACCCTAAGGAAGTCCAAATACTGCAGGACGATCGTTTTGG

TGAGGGGGCGTTGTAGTTTTCCTGCTGTTCTAACGTAACTGGACTTCCTAACTTCCAGGGTGTCCACCAT

TGCCAAGTGTAGTCTCGTCCTTGGTTGGCATCATTGTGCTCCCAGGCACGGCGGATGATCTGCTCATTCC

AGAAGTCTGGTTCCTGCAACTTCTGGTTACGGTGTTGCAACAAAACATCATTCATCATTTCATACCTTAT

TTTGTAGTCACGAATGGCATGTGCATCACACCACTCGTTAAACAGGGTGGTTATGTATGTGACATAATTC

TTGTCCATACGCTTCCCTCGACACCGGACATTCAGGTAGGCTACTGCATCTTCACGTTGGTTCATGTACA

ATTCAGCCAATCGAGCAGCATTCTGTATAATTCGTTCTTGCTCTTCTATTTGTGCTGCTGCTGGTGGCTG

TTCTTCTTCATTTCCGCCCA

>GASO02037726.1 TSA: Tricholepidion gertschi C202207_a_9_0_l_1773 transcribed RNA sequence/tombus-like virus

GGTCTAAGGGGCTACAAGGTTTTCTAAACCTCCCCTGCCTTTCCCCAAAGCACCGTAACCTGCGCTGGCG

GGCGGAATGCCATTCTGGCCCCGGAAATGGCAAAATTTCCGGGGACCAGCACCGTGGCTGGGCCGGGGGT

GCTGGCGCTCCAACCGCCGCAGGCTATGAAGCTTGGTGTGTTTGTTGTGATGGATACCGACAACGGTGTC

CACCCTATTTCTGTCAATGTTAAAGAGTCTGTTACCCTTTGCCACCTTAGGCTGTCGGGAAGGCCACGGA

GAATGCGCCCGGGAAGGGGGGCATTCCTGGGTGCCTTCGGCTGAACAGGCCGGGGGCGGGGGTTGCTGCT

CTGGAGGGCAGACCTCACCTCTCTCCGAACAGTGGGTGTCAGGAGAGAAGCGAGGCGGGCAACAGGAGTC

TTGGAGTTCATATCTATCAAAGTGATCAATCATATGCCCCAGGTAGTCTTCTATAAGTCTGCGGAGGACT

GGATGATCAAGTCGCTGTGTTGGTGAGTAAATCGCAAGGCCAAGCAAATACCTTTCGCAAGAGATTTGCT

CATGAATTGGGATCCCAGTAGCGGCTTGAAAATCAGCCCTGCCAGTCTCAGTTGGCTCGGAAAAATCAAT

AGAGTTGAGTTGCTGACCCATAATTTTTCGTTGAACGTCCGGATGCCACTGGCTATAGTCTTTTCTAGGA

GCGCCTGCTGCGCGCACCCACTGTTTGCACAGTGCGCCAACAACTGGTACGTTCCCGTGAAAAATTGCTG

CGCATCTAGCGACCTGTGCGCCTCGTTCTCTAGCCAAGTGTGGCTTAAGAGCCTCAAGGGCATAGGGAGC

TGTGGCAAGCATCTTTCCGGGGTTACGGATGTGTCTGACGCCTGAGTTGGTGTGGACGAATATACCAGAG

CAGTAACGGAGTTCGTTATAGTGGTAAGCAATTCCTTCGATTTTTGTTTTAAGTCCGAGCAGCTTGTAAA

AGCCTGGGGCAGAGTCGGAAAGGATGGAAATGAAGTGAGGTTTGACAAAAATGAGGGCGTCGTCGCCGTC

GAGATAAATTCTGTATTGTGAGATGTGCAAGGACTGCAAAACCCAGATTGTTGAGACAAGTTGGACAACG

GTGTTTCCCAAAGAGGTGTTGACATCGCCTGAGGCCCGAGTTCCCTTGGTAGAAATTTTTCCCCCACATT

TGGTGTAGATGGAAGACCGATGCTGGTTGGAGAGGATCTCAGTGAAATCACACTGGACATTGGACTGGCC

CAGCATTCGTCTGTAGAAGGGTATCTCCGTGGCGTTGAGCAGCTCTGCTGATTCTGTGGCATCGAATCGT

GAGTGGTCGACAGAGACTGCTGCATAGCCAGCTTCGAAGTATTTCTTGACAATTGCTCCTTTCTTATATG

AGTCGTGTTTCTTTGAACAACATGGAAGAGTGAAGAATTGTTTTTCAAGTTTGTGGATGAGTGGGCCCCA

GGCAACTTTGGCATACACATTGCGACTGGAGATGAGATGGGTTGGTTTGACAGTGTCACTGGTGTGTTCC

CGTTTGAGGAATTGGTCAATCCGCGCGTAGTGGACCTCAGAACAGGGGAGTAATTCACAGATGAGCAGTT

TCTCTCTGGCTCGCTCAAGAAGGCCTCGCTGGCCAGGAGAGAATCGCGAGACCCACTGTGAAAATCCTCC

TGGGAGATGTCTGGTTCGACGGAAGATTCCAAGGTAGGGAATAATTTGAAGACGGTGTCAACGGCAGTAT

TAAGTGTTTTTATACTACTCTCG

>GFPE01052446.1 TSA: Holacanthella duospinosa c41094_g1_i1 transcribed RNA sequence (7680 bp) benyi-like virus

AATAATAATAATAATAATAATACTGATAATTACAATAATACTTGTGATAATACTAATACTGAATTATTCG

AATGAGTCAAAGGTTTTAAATGAAGTAAGATAAAATGAAAGTACAACTAAAGGTTTAAAACTTTCCTTTG

AACTACTTATTTCAGTGAAATATTTGACCATTGTCGCCACCTTGTGGAAATGATGCTTCTTCTGTTCTGT

CTTCTATTATATTATCGTATTGTTCTTTGTCTATGTGTGAAAAGGATACTACTGATTCCCACATACCTGT

CACATCGGACTCTGGTAATCTGTACATTAACATGTTAGCAGCCGTTGTTTCTTGTACACCAATCTTCTCG

ACTAACTTTACCCAGTCCCTCAATGCGTTTTGGTATTCACAAAAGTGTTCGTAGGTTTTGTAACGGTGTC

CGATAATTTTATTTAATTTTCTTTTAATTGATGGGAAACATCCAGACCTTGAACAAACTAGTCCACAGAA

TTCTACTGCTTGTCCAAGATGTATTTTGATTTTCATATCACATGCCTCTTGTAACTTTTGCAGTCTTTCT

TGTACTAACTCTAGGTTCAATTGTCTCTTTGCAAAATCATCTCCTTTTGATGCTACAGCAACAGGCCCCA

TACCTCTTAAAACATAATTCCCAAAAGTTTTTGATATGTTTTCATTATTACGTAGTGTACCGGGTTCGCC

AGAAGGTTTCTTCCAGACCACTTTTCCATACAATACCCCAGCTATTATCGTGTATTCATGTCGGTATTTG

TAATAGTGTTCGATCATTTGCTCTGACATACCATAACCTTTCCACATCAATTTTTCTATTTGTTGTGTGA

ATTCATTTTGACCAGCGTCACATTCTACAAAATCAAGATAACCCCAATTTGCTGTGGTTGGAACTTTCAT

AAATTGTTTTCTAAATGCTTCTATCATTTCTAATTCTGAGACATATGCGTCTGACACGACTTGGTTTGAT

TCCATATTTGATATAGCTTTGCCTAGTATTCGCATTACAGGTGCAAACATTGTGTTTAGCTCCACTGACC

AGGCTGATATTCCTTGACCTGCTTTTGTTCTGTCAAGAATCCCACTGTCTTTGGGTTTAAATATGTTCTT

AAGAAAGAACCTCACTTTGTCATAATCTGGGTCGTCTAACCCGTGAAATCTTTCTAAATAATTTCTTGTG

GTTGCGTCTTTAAGAAATCGCTCTACAGCATCATTCATTTCACATTCTGCTTGTAAAGATAGCGATTTGT

CGACATGTTCATCAAACCATAAATCTACAACTTCTTTTGCAAGTTGTTGTTGCGCGAAACCATATTCTTT

GGTCGGCGCTTTGTTTAGATATCTTGCTTCGCAGACTCTTGCGGTTTGCATTGGTTTTCTAGCTGTAAAA

TGATTCCCATATCCTGGCCCGAATGCTTGATATTTACATTCCAATGCCATTGGTCTGTGCCTTGCGTTTA

ACGGATTTGAAAAATCAACATCAAAGGTGGTCTTAACAAACTTTCCTTCACACATTTGTGATTCGAGTTC

GTTTAATGCGGTTGTCTCAGGTTCAAATGCTTCTGGAGGTAAGAACTCGTCTGTCAACAAGTAAGCGTCC

ATTCCTGGATGCTCTTGTTTTTCACGTGCTGGTGTTGTATCAATGCGTGAGTCCCACTTTGTTGTGTAAG

TCTTTACGCTTGGTAAATGTGTATACATCAGCATATCAAATTCAAAATTCTCTTCGTAACGCTTTCTTAT

GTTGTCTGAACGAGTTGGGTCTTGATCAACGAGAAATATTTGATCATCCTTTTCGTGCGTCTTTGCTGTT

CGACTTTTGAATTTTTGCACAAGATTTTCTATCGCGCTTTTCCCTTGTTTCGATTTTCCGCCACCAAATC

CTTTCTTCCTGTTTGGGTTTTTATCTTCTCTTGTTGGTCGTGAAAATTTTGCTCCCATTCTTAGAATGTG

ATTATCATATAAATTACATGTTTCTAACCAAGACGGTATTTTTGCCTGTTTTCGTCCTGTTGTACATACT

ACAAGTCTCGGTTGTATTCTCGAAAATATGCACGCCGCAAGGTGTGTTGATAATTCTGTTGCATTCGAAA

TGTGTACTACCTCGCGCCCTTCTGCTTGGTTAATAAATGTATACACATCGCGAAATCTTTGTCCTGGGGC

CGATGCAGCGTTTAAGTGTGGTACACCGATATCGACATGGTTTATAGTATCAACAAATACAATATCCATA

GTTAGTGTCGTTTTGCGTAGTCCACTATGTACTGTTGCCACTTCTGGATTGTTCATGCTTTTAGGTAGGT

TCCATTTTTCTCGTGTTGGGTCTATATTGTAAAAATAGCGCACAGAGCTGTTGTTTGGACATTCAAAAAT

ATCTTTTGGGCATTCGCAGTCTGGTAAAAGCAACTCGGCAACGTCATAATGTGCACCGTAATAAACTAAT

GCTACATTAGCAGTTACTGGATAAACTCCGACTGTTCTTGTTGTGTAAGATTTGTCAGCCGTATTAACAT

GGAATACAACGATTTGTTGTTTTATTATCTTTGACACTACCGAAATTGAAGCGTCTCCACCCTGCGATTC

GCAGTCCAAAAAGTATTCTTGATTGCACCATTCATCTAATACTATGTCTTCGTCTTTGGATGTTGTAATG

TACTCTGCGCATGCTAATCTTATGTTCATGAATTCTTCTCCTCGCATTCCGGATACAATCGCTAATGCGC

GATAGCAACACTGGGCTTCTGCCCCAGAAATTGGCGTTATTGCACCCAGTCGATACGTTTCGTGCACCGG

TCGGTTCACAATATTCGTGCGGGCCACAAATGCCGAATCTGTCCTTGTACGTTTTATTGAATTTTCTGAT

TCGTTTGTTGGTATGCCATATGAGGTTGCTGGCGGTTGTCCTTCGTTACTCTCGATATCTTCTTGTTTGT

GTCTGAAACTCTTTTCTGAGCTTGTTTTGGGTGTCAAATCTCTTAGTATGCTTCGTACACTTTGAATATA

TGTCGAGAACAACTTGTAGGTTGCGAATTCTTTTAATATACTTGTCAAATTGTCAAGCATTTGTTCACGA

CTGAATAATGCGTCTTCTGTCGCTCCTTCCGTCATTTCTTTATCGTACAATATAATCTGGTTTATCTGGT

TGTCTATTGCGATTAAGTCGTTTATGCGACTTTCTTGTCTTTCCCTACTCAGGTACTGTATTTTAGAGAG

CTCCTTTAACAACTCTTGTTGTTCTTTTGTGTCTTCTGGTGAAAATTTTTGAAATAAACGTTCAATGACC

CGAAATACATCTGACAGCCGTAGAATGTCTTCTGGACTTTCCACGGTTATTTCGAGAAAGTTAAATCTTC

TTCTAAACATTCTTCTTATTATCACAGCCTTTGGGCACAATTGTGATAGTTTGTTGTAGTCATCTTGTAT

AGGTGGTCTTTCTATTGCTTTTAGTATTATTTCGTGTACTTGTTCTAATACCTCCGGTTTTGACAGCATC

TCATTTAGTTCAATAACAACTGGTTTTGTCAGATTCTCTTGATTTACGGTCCATCTATTTGCTATTGTCA

TGTTTCTCTGTGTTAAGATCGCGGTGTTAAAGTTTTGTTTCACTTCATCTTCTTCATTCTTGTATTCCGG

CCATTGTTTGTTTACGTCTGGTTCTGTATTTATTTTCAGGCTCATTGGTTTTACGCTGACGTTTTCTGTC

GCTTCTTCCATCAGTCCTGCAGGTAAAACTGCTCGTGCACTATTTCCTTCAAACGTTTTGAAATACTTTG

ATAGATTGTAGCGTTTGCACATTTTCTGTCTCTCTTCTAAGAGCTTTTCTATCTTCACCTTGTACTTCAT

ACTTCTTTTGCTACTTGATGCTATTTGTTCGTAGCCTCTTTCAGCAATACTGTTTATTCGGTGCAAATAG

TAGTATTGACTGTCGCACAATGTATTCACTGCTTTTGAGTATGATTCAATGAATGGTTTACATGTTTCTA

TTTCGTATGGATCAAATAGAAATGTTCTTGTTGGGACGTAGTTTGTGCCCTGCATGAATTCTTGCATACG

TGCAGTCCCATCGAAACCTTTTGCACAGACATATTTTTCTACTGCGCTTTGATTACTACAATATGGTTTT

ATTAATTTTACTTCTCGAAATGTGTTTGTTAATAAATATAGTATACTCATCACAATCGGGTCAGTCAAAT

CAAATAACTTCATCACAAATGTTCCACCTGGCGCTAGAATGGCTTCCATTGTCGCTTGCTGTCCTATGTA

TATTTCCAGCATTTGTTCGACGGTTCCGGAAGCCCCATCAGCGACGACAACATCAACACCAGATTTAAAA

ATTCCTCTGACGTTTTGTACGAACAAGTTTCTATTCCTTTTATCAGTTATCCCTCCTTCTGTGACTCCAT

AAAAGGCTAAAAACTGCTCGTGTTCAATTTTATATGGGTATTCCGGAAGCGTGAAACCAAAACCTGACAT

ACCCTTTACCTTTACTAGATACTCTGCAAAACCGCCTGGACCGCCGCATAAATCCACAAACGTCGTCTTC

CCGTTTGTCACATTTAATGCGTTGTCTATTGCACAGATTTTTGTAGCTCCTCTTGACACAAACTCTTTTG

CCACAGGCATTACAGCCGGGAGTGCATTTGTGTATGCAGTCCGAAACACTTGATCATTTAATGTTGCAAC

CTTTTCCTTCATTGCTTGTAGTGCTGCTCCATTACCAGTGTTTATTATTAATTTTTCGGGTTCATCGAAC

ATGAACATTTGACCGATTTTGGTTTTGTTCTTCTCCAATTTAAACCAGACGTCTTCGACCGGGGGAGACC

ATGCTATCCCTCTATTCAGTATCGCGTCCGGTAACGCAACATCTTCTATTTTTCTGGATTTTGGTGGTGC

TGTTAGGTGGTCGATTATTGTTGTTATTGAATCGAAGCTACCCATCACTGTCTTGTTATCAATTCTGTGT

AGTTTTATATACTTTGTCATTTCGTTTAAAAATTGTTGGTTGTAACCTAGTCTAGCGACCCATTTTGCCG

CCTCTGGGCTGTTGTCGATTACCACATTTAGATTTTCTTTGTGTCTACTAAGTGCAACAATATTTAACTC

TTCTGAGTCTAGCAGTGCGCTGTCTTGGCCTGATACATATAAAGTCACATTCTCATGTGCGGTTCCTTGT

TGGCTACGCACTGTTGCCTTTTCGTCCTCTATGTACAGTTTTGCTGCCGCATGGCTGTATGCCATGTGTA

TTGCTGCTTTAGTATGATCTATTTCGTTGACGTTCGTCATAAAGATTGACGGTTTTCCATTGTATGGTTC

TTTTGGTTCCATGTCATAGTCGTACAACATATTCAATAATGCAACTGTGTCATACGGATTTCGAAAGTTG

ACTATTAGACGATGTCTTGGTAAATCTTCAATTTTAACGTGGTTTCCTATGTACATCCCTTCTGCGGGTT

CCTGTACCTTTGTTTGCCTGTGGTCTCCAACTAAATAAACCTTTCTTGTTCCGTTGTTGTGTGCTGCTAT

CATAAGATATTCCATTGGCATTGATGGCCATTCGTCAACAAATATGACACTATGTCCGCGTGTTTCCAAT

GCACGGTGTTGTGTTTTGAATGTTAGGTCATATGTTCTTCCGTCCTTTGTTATATTTTTGTAGTCTGGCA

TCAGTTTTGTGAATGGCGCCATAATTAAGTCGTGTTCTGTTGCAAGTTCACGTATCACATGTGATTTGCC

GGTGCCTGGACCTCCTTCTATATATTCAACTTCTAGTTCCAAGCTAAATTCGTCCGGAGGCAAATGTGTC

GCTGCTCTCTCTTTTACATTTTTCAGCCCGACTGGGTCCATGTCATTGTTTATTAAACTTGTCTTCAGTT

TTTCAATTTGTTCTCTAGTCATTCTGAAAGTTACTGTCGGATTTCCGGGATGACACGTGACCTTTTGCTT

CCCAAGGTGTGGTATTATGCGTGCACAAAAGTCACATGAAAGTGTCTCTTCTATTTCAAAATTCATGTAT

ACAGGAATTGTCGTTGCCAGTACCGTTCTGTCGGCTTTTGTCGTAACCATTTGTTGTTGGAATTTTGATG

GGTCACTTACAATGAATAGTTCGTCGGCAAGATGTTGCGAGAGAATCCAATCGTATAACAGAGTTATTGG

GAAGAACACTGCTCTACTTATCTTTTTGAACATCGCGATAAATTTCTCCATAACACTTGTTGGGGCGAGA

TTTTCTATGACGCGTGCCACGTCTTCTGACATTATCATTGCATGTATAGTCAACACAACTGCTATTTTAG

GCACATCTAGTTGTTCTATCCGCATAGGCATTATTATATCAATGTCATTTCCTGCTTTTACACCACCTAT

TCTTCTTCTTATGTACAGCATTGTGTTTTGGAATGTAAGAGAGGCTCGTGGTATAGCCAAAAGATAGTTC

AGTACTTCACACACTTCGCGTTCAGGTGCTGAAAAATGGTGAAGTGGTGACCTAACACGACCTGTCACTC

TATTTACTGATTTGTCTATGTCCAGCAATCTTACATAACGCTCATACGGTCTCAATTCTATTGTGCGTGT

ACAAACGCCTGGATTATTTGTGCGCATCATTTTGAATGCTGCCATTGGTCCAGCACGTGCCACAATCTCC

GTCATCACGTAGACTCCTTCATAAGCCAGCACTTGTTTTCTTAACAATGTTGACCAACTGTTCCAGTTGT

GTGTATAGCCTGTACTGTAACCTCCTCTGTATGTTAATGACGCGTCCTTCCCTTCACGTTTGTAGAAGTA

CAGTGGGTTGTTTGGTAGATCCGTGAATATTAACTCCATTGGGAATATTGCGTATCCGAAACCGAACATT

GCGTTCGTTTCTATAAAGGCTTTCACTATTTCCTTTTCTGATAAATTATATATTGAGTCTTCAAAACAGA

TTGTGTTGCACCTTATTGGTTTCATGTGAATTCGATCTGGACATTCGTTGATTTCACAATATGCTCGGAA

AAGATTTTCAACCTCAACAATTTTTGCCACTGGCGCTGAATGTGCTGTTCCACCCAACTGCCTTGATACT

CTTATGTCATTTCTTGAGGCTCTTTTCCGTGCACGGTCCAATATTGAATTTAGTGCCGGTCTTATGACTC

TATCAACGTCTTTTCCTTCTCCATTGTACACATAAAAATGACAATTGGTCTGTGTCCAATACATTGATAT

TTCCCTTGCACCTGCACCCCAGAATAGAACACGTTCCTTAGCATTTCTTATCTTAAATGTTTGGTCGTAA

ATATTACGTATCAGGATGCGACAACAGGCGATCACTGGGTGGTCATTTGTTATCGTTTCCCCACCAACAA

ATAGTATAGGGAAATCTAACAACGCTTCTAACATTCCCCTCTCTTGGGGTTTTAATTGCACTGCTAGTTT

TTGAGAGCATTTTACACTTTCCTGAAAACTTTGACTTGCTACTGCACTTCCTAGATTTACTGCTTGCGCA

GTTCCTGCTATATCTACTATCTTCTGTACCGACATCACCTTTCCCCCTCAGATCACTCGTTAAAAGGACC

TAAAATGCAGTTCACGAAAACTCAACACTTTGTTTTTCCTAAGTCTTGGGCAAAGAATTAATATTCAAGA

CGAAAGATTGACAAGTATCTCGTGAAAAATGAACTGCCTACCGTTAAACG

>GAUE02013860.1 TSA: Anurida maritima C98610_a_10_0_l_4248 transcribed RNA sequence REO VP1

TGGGGGATGGCTAGGGCTAGTAAACCCTATTACGCGAACGGTTAAGTTGGATCTCTAGTCGATGACTCGG

AATTAATGTGGTTCTGGATCCACCATAATCAGTTGGAAACATCAATAGTAATCCTATCCCTATTGTTTCT

AAATACTTTTTAACAGACTCACTTCCTCTATTAACGTAAAAGAAATGGCGGCTAACCGATAACGAAGTAT

CAAAGAAAAAGATTTGTCTAGGATTTAAAGCAAACCTAACTGAGGTAGTAGCCCAACTGCCATTAACAAC

TAGGTTTTCTAGATCATTCATCATTTCCATCGAAAACCCGAGAGATTCCCGCAACAACTCGAATGCAGCC

TTTCCACGACCAAGAGCCTGTCTTCCTAATTTTATGTACATCTTCTCGTCCCCAGGCAGTCTATTATCAG

AGAACAAACGCTCCTGTATAGCTAATTTACTAAAGCCGTGAATTCTAGGTAATCCAAATGCACCAATCAT

GTAAGCAGATAAGCTGCGAGGAGGGACACAAGCGCCAAAACCAACATCCTCAATATTAGTGCCTAATCCT

TTAGTTAACTTTGTTCCTCGTATTATTGAAAAACTTCCTATAGCGATACGATCATAATGTTTCCCAGCAG

AATTTCGGAAACGAAGTAACCTATCAATGATATTTACAACTTCTCGTAAAGACTCACCACCAACGCGACG

TACCTCAGTCATTGCCTGATCTATCTTACTACCGCCCCTATATGCATAACTAGCTGACTCAGGAACCATA

ATTCCATAACTAAGACGCAAATCATATGTGGCCCTTTCCGACATCCCGCGTTTTCGAGGATCAGACAAAG

AGTTAGCAGTCCGTTTCCACATAGTTAATAAAGGATCCTCGTACCGACTTCGTTCCATTTCAATTGTTTT

AAATAAATCAGGCAGCTCCCAACCGACACCAAGTCCACAATTCCATAACAGCTCAGTGTCAAATTCCATT

TCTAACGGCAGTCCAATCTCATGGGCATAAACCATGAAAGCATTAGACAAATGATCTTCATTGATCCCTG

AAAACTTTGGCATTGATTTAAGCACACGTAACACTTTATTACGAAACGATTGATAACTCTGATTAACACT

AGTAAATGCCTCTTGAAACCGTATCCGCTGTTCAACATTAGATCTTGAACATAATGCTAAAAATTGATAT

GTACAAGCTGAACTTGGAATCGTAAAACACGTCGAACCCGATCGAGTTATTCCATCAAGTCCTTTGATTG

CTGGAAACGGAAGACCAATCGAATCAACTTGAGCCCATAACCCTTTTGGACAAAAATGAACAAATGTTTC

AATTTCATTCGCCCATGAGACACTAAAGAAATTAGTCGTAAACTCATCTTTCTTTAACAAACGATCTACC

CTACCTTTAACGGAAATGCGACTCTCACGAACACGTACCTTTTGTCTTCCACCTCGATTAATTAGTCCAC

CACTGCGACCATATCTAATAACCGTACCACTACCACACCATTGTACTAACCACATGTAACCAACCCAAGC

CTCAGGAAAAGGACATCTTGCCATCTTTTCTCTTATCATATCCCACATGACACCACATCTAGCTGGAAAT

ATTGTACCCGCAACGTCTCCACGTTCAGCAGTAAATAATGCCAATCTTGAAGGAAACAACTCAGGTGCAC

CGCATACTCCAAGCTGTTTTAAAAATTCAGCGGTTGTGTCACTACTAACGGTCTCACATTGAAATCCTAA

CGCTTCATAATAATGACGTATTAATGTTGCTGCTTCTATACTAATTTCCCGAACTTCATCTGAATCCATA

ACACCGACAGTTTCAGTCTTGCTAGTAAGATCATCTCCTGCAACTCCGCCACGTAAACGAATCTTTTTGC

AATATTTAACTAATAAAGGATATAATAACTCAAATTCTTTCTTATTATAATATCCGCTCGCTAATCGCTT

GGCTATAGTTTTTAATATGTTCGTTCCTATCATTGTATGTTGATCGGAAGTTGGGAGCCATCCTGATCTA

AATGCAGTACGTGAACAATTGACGTATTGTTGAAAGAAACCATCAGAAAACAGAGTTTCAGCGGTCTGAT

CACGATTCATTATGGCTATCACCAACTGAGGGAAAGTTATATGAACGTCCTTCTCACTTAGAATTTTGCC

GTTCTTATCTCGTCTTCTTTCATGAACAACATGATAATTATCTGCACCAACTTGAGATCCTAAGAATAAC

GACGGCCCTCCTATAGCATCATACCTGCACCGATGATATACTGCCGCACATGTAAAATTCACTTGAGCTT

TCTTTGTTGAAGAATCCATACCTTTGACGTCCATCGAATTATTAACGCCATGTTTCCCAGACATCGATAA

CACCAAATTAAGGTCACGAACGTCACCAGTATTCTTCCCAGAAACACTATAATCGGTACTCTTATAAGCA

GGTTTCATAACTTGGTGCATAACAAATCCTGGTAATTGAGCTGCACTTCCAACCATTTGTATAAATCTTG

GTCGTCGATTGATTTGATGTCGTTGACCAGCTTTCTTTATTGATTGAATAGCCCTTCTAAAACCTTCAAT

TGTTGTTAAATCATGTATGATCGTCTCTGTTGCATCCATCACTCTGACATTCCTAACTTTTGCAAGAAGC

TGTCCTAACTCGCCTTTAACATTATTGACTATCTCATCCCTTAACTCTTTTAACGTTTCATCAGTATTAC

CAGAACTGTTTGTTGTTTGTACATCAAAGAAGTCAGATCCCCAATTGTTTTTCGAAGTAATTTCTACACT

GTCAACCGTCTCAAAAGAGCTTAATCGTAATGCCTCTAATATCTCGTCTTCATACCACCAATCAACCTCA

ACCCACTCTCCGTCTAAACATACTTCAGTACGAGGACTACTTTCTACTTGTTTAAAACCAGGAGTAGCCT

GAGCTGACTGATCTAATGAGAGACTAGTTGCAGATTTGACATAAACAATTGCACCACCAAACAATTTTAT

AGCACTGGCAAACCATCGACCGTCACTATCGCTCATCTCAGGAACATTCCTAATGATATCGCTCATACCA

GGACAATCACGTTCTAAATTAAGGTACGCATCATCAGGAACAACAACTTTTGGAACGTTCCTAGATATCA

CATTTCGAATGGACATATTATTAACTATAAAATTATTTAAATTCGTCCTTGACAATAGAAATTCTCTAAT

AACATCGTAATGACCTTTACGTTCAAACATTTGCCCAAACATTCCTATTCTACTCCTATCACACCATAAA

TTAGCTAATGCTGTTTCTATTTCCCCAGTAATGTTGCAATCGCTAACCAAACAAGATTGTTCTAGTAATT

GGAAACATAATGTTGGTAGTTGACTACTAATCGCTTTAGATGTACGCAAGAATTGGACTCCTGTTCTAGT

ATAAAAGAAAGGCATATCAACGTAAGGCTCAAATAATTCAGCCATAGCACGAAATGAGGATGAATAAGCA

ATACATAAATGATCTGAATCAACTTCTTTCATAACCCCAAAAGTCGGATCACTCTGGCTTCGCTTTTCTG

CACATACCCCACAAGTATAACCAGCCATTCCAATCATGACGCATTCATTCATCGTTGCACCTCGTAACTT

GAAAGATTGATCCAACATACGCTGTAATCCGTAAACAATTCGATCGTGCTCACTCGCTCCATAACTGATT

GAACCTTTCATGCTTAATAATGTCTCAGTCCGAAAATTACATAAATATAACGCATTCTTATTCTTTGCAA

CAATATTTTTAGAACTTGGATCTAGTTTGTAATCAAACGTATTGACTACTGGTGGATTAATCAACTCTCG

AGGAACAGGCTTATATTTTACTGATATACCTAATCGTTTATCTCGCTCGTTCCTAACATTATCAACAAAT

GGGATTGATTTAACCATCGCACATAGTATTGCTCCATGCAATATACCGGTTTTACCAAATAGTCTAATAA

TCTCATTCCAACTTTCCATTTTTGATATATCATGAACTTTCCTACGAAGTATTTCATCTCTCATATTATT

CCATCCTTCAATCATTGATAAAGCTAACGCAACCCCATCATTAAATGGTAATCGATTCGGTATATCACTC

AGGGAATCGATACCCAATTGTAAGCGATCATCAGTTGTGTTATTCGTAAGGTCCTTATTTTTAAAATTTT

TACGGAGAATATTGATGCGTGTTATAAAGTTACTAGCCCATAATTTCG

>gb|GAUE02012122.1| TSA: Anurida maritima C91402_a_6_0_l_1853 transcribed RNA sequence REO VP2

CATAGTTGTACATCGGGAATTTTGAATAGATTGATATATTCTTTAGTTAGTTAAATTTATTAGAATGAGT

AAGAATGACCGTTATAGAACGAAGGATACAGCATTATCAGATCTACCAAGTAAGGATGAGGGAGAGGAAG

GTAAGAAAGATAGTAAGGATAAAGTTTCTAAAGAAGAGAAGGATGCGCCGAGCACGAGTATTCCAAAGAA

AGTTGATGATCCGAACATGGTTTTAATGGGTAAGATGATGGAGATGTTTTCAAAATTGATGCAAGGTACT

GGAGATTCACAAGATAAGAAGGTTGTGACTGAACCTGGTGGAATTCATCTTACACGAGAAATACAGAATG

CTGGTAATTTGAGCGAGATGGTGAAAGGCTTAAGCGTTAATTCGGTCTCGATCATTGGCAGCATGAGAGG

AGTGGATTCTCAGTTCCCTGTTGACGAATATAGTACAGGCGGTTTGGATATGCCAAATAAGATGCCTCAT

TTACTACGGCTCGTTCCTATGTCTGGTTCGATGGGTGCTGAATATTTGAATCCTTCAAGTAGATTAATAA

ATATGGATGATTTTGTATTTATTGAGGGAGTTCGGAAAGCTTTTTGTCATCCGTTAGATGCAGTATATTT

GAAAGTTGAAACTAGTCAAATGGGTTTCATTGCAGAGGTTGATCCGTTTGATCTTTCTAATATACGTATT

TCTGTTCCTAATACCGATGATTCTGGAATAAAGAATATTGTTGGAAATGAGCGTGGTGATCCTTTGGTTA

TTAATGGAACGCAAGGTAATGTTGCGGGTGTTATGCAAATAGTATTGTCTTTGGTTGCTGCTGCTTCTAG

GATTGGACGATTTATACTAGTAGGCACTAAAGACGGCAAAGATTGGGCTACGAACGATCGGAACGTTGGC

GCCAGAGCACAAGAGTATGAGCGAATGCGCGGATATATTCCAAATGATGATTATGAAAGGTATCCTCATG

TTCCTGAGGTTTGGTCTAATTGTCATGATTTACCTTGGTTTGCTGAAGAAGTGCGTCCTCAAGCATCTAG

ATTGAGATTTGGAACTATGAGTACTGGAGTTGGTACGCGTCATCAACGCAAATTCTTTGAATGGATGTAT

GCCAATGTCGATGTTCCAACCATGGAGTGTAATGCGAATGTTATTGAAGAAATTCGTGACGAGAGGATTG

ATTTAAGCGCTTATAGCGAGTTAATCAATGGATGTATTCTTGGTTATGGAGAATATGCCGGGATTGTTGA

TGACATGAAATTAGAAGGGTATCATGTTCAAATAATGGATCCAGAATTAGAGAGGCATTCTTTATTGTCA

TTCCCAAACGGTGTGCGATATAGAGATGGGTTTCGATTGGCAGAATTAATCTCGGATGAATTTAGAATGT

GGAAATTTCACAAACTTCGTGAGATGTTTGACGAGAAATTGAGAATGGTAGATCAATTGTCGCTTCCTGA

AGGAATGGCTGGTGTAACTAGTGCTAAAAATCCCGCTTTATTGTCCTCGATGTCAACCGGTGTGATTGTT

AGAGAATGGGGTGATCTAATGGAAACAACAAATTTTGGTCAATTGGCGCACGTAGCTGTTCAAGATATGT

GTCCGGAATATGTTAATGGAATACTTAGGTCAAACAATGTTGGTGAGCGTACATCTATGAAGTTCATGTT

TATGCTTCTTGGAACAAAGATTGCTTTCTTGATGACTCCAAACCTCTACGATCACAACAAACATATTAAA

GCGTATATGATTCACACATTCCTCAGGACGTTTTTTGAAGTTGAATATTTGGCATGGCAAGCTAATTCTG

GTTATGCTGTTGGAGATAATGGAGAGAGAATAG

>gb|GAUE02012094.1| TSA: Anurida maritima C91298_a_4_0_l_1840 transcribed RNA sequence REO VP2, second part

gcatccgacaacgtacttatcgtgcgccaggaaggtcgtatgttccgagtccgggagttgag

actgcattcagacctgcaactgttcaattgagacaagtgttagctattgctaaagagtat

aatgagaaattctatggtccaggtgaacgtactcagacttcaaatgaaaggtcggtttct

aacataattcgaatattgatggatagatatgctgttggttttggagagtggttccattgt

atatattgtccagcttatcatatggttgcattacatccattactgcataatgatgttaat

gatcctggattaggatggcgaaatatggttggtgttatggaggatgcagaagaattagct

cgattgaacatgccgatgtctccgtttagtcaaccacatccagctacttttcctattcat

tcaactttcggtccaatacatccaactgaagcattatatatattcaccacagcccatgtt

ccaagcgggtactataaaggtggagtagttaatgagaatagccaaatggattttgattgg

tggaatactttttggaattcaaataatggtcaagatatggctaccacttggaaacgatca

cttgaaataggtcatgggatggttgaggttatgatggaagttggagagttgtcaagaggt

ggactgattgaaaacgatatgttgcgtcagtttagtgtcgaagcaatgactagtggagct

ataacggcagctggatcagcaatgatgatgagatttcttggacggtcaagtaagggtgat

actgcggcgatggcaaattctatgagtgcaattgataagattcatcaagatccaagaatc

tatgctccattattcgttactattaacagacacatggggattgtcgaatcgcatcgcggt

ggtggtgatttaattcgtccgtcgattcgaaagaatctcaaaatcttgaccgaattaaca

ttcgacccgaggattggaataatgaaattatctcgcgggttagtgttttctgaaagattt

ttgagaggcgttagatataacacgactcatgtggttagcaatgtaagtgtatttatgagt

aatagagttgaccacatcagaaatccgatcactcagagtgttgctaaaacttataatgcc

atggttgtttggagatctcgagttggtgaagctgttgtacgacgcgttacaattcatttc

caatctatcgttgaactgttggatttatgttctgacgcagttgaggaaaaagttgaattt

gtcacttttatgattcccgatgttaccagattagttcatgatatttatgattttattgtt

cgatgtgtgaatttacggcttggagttgctgttttcgaaaatcctttcattatcttaacg

tatgggatttctcatagcggtactctccaatcgatggttcaaacccaagaaagggctgat

attttagctttacctactaggaatcacagagctataactgagattaaatgctattcaacc

ggagttatgcgaggtggtcttgatgcagaacaaatagttaagccgttacttccaagaact

ccctcagagcctcctttatttactactgaagttaatactgtagtccgaagagagagggga

ttggttgctagaatgaatgagtactttcctattttaagtgatgtcgaggacattcgacca

tttgtattggatcctgtgactaacagaaatgttagagcttatactttatttccgtttggg

cttaggactagggttcctttgtataaaaatccaatggtggtctcaccaacaataactacc

caaacgagacaacttccgttctagttgcatgtccccca

>GAUE02011164.1 TSA: Anurida maritima C87378_a_10_0_l_1411 transcribed RNA sequence, REO VP3

CCCGCGTGGGATCCTATGTACTATTCGACACCAGGTAGCAGACGACAGCGGAAGTAACGTCGTAAAGCAT

CGATGCCTCAATAGTAGGAATAGTTCCTCGGATAACATTAGCTCTTACGATATCGACATAACTAGGAATT

AAACGTTCCTGACTTTCAACCAAGCTTATTTCAGTTAGCCTTGGAACGAATCCACTCAATGGATACTGCG

AACTTAAAACTTTACCTCCATCTAATAATAATTGTTGCCTTAAAGATCTTGGCATCCTAGATTCTGCGTC

ATTTCCTGAGTCACCACCTAAGCCATATGTCCTAATGAAAGTAGCGCTTTCTGATTCCCCGGTTTCTTTT

AAGAACTTTAAAGTCTGTTCGGCTGTAAAACCAAAATTTATAATTACGTTACTAAAACAGAAAGCATATT

CACGTAACTGAGAATCATCACTCATACCAGCTAATCTTGCTAACCTACGCATATTCGAAAGAACATTTTC

TTTTGGTTCCCATTTTGCGTACGTGACGTTTTTAGTATATAAATATGTGACTCTGGCTCCCCATGCACCA

CCATTAGGATCACAACAGTAATATCGAATATTGCCAGGAACCCATCCACTTAATTCTCCACCACGTCCTC

CAACGTCTATCAAATGTCTAACGTTCTCGAGGTTAACTAACCATCCATATATCATCGGTCCTGTTACAGC

TTGATTATATTCTAACGGTCCAACAGAAGTATAGTGGTGTCTACTCCCTAACATAGGATCTGATGATAAA

GCGAGGTCATTATTGATCTTTCCCCAATCAACTTCCCCAACGGTAACAGTCTTTCTTGCAGATTTGTCTA

TATTCCTTAATGCCTCATTAATCTTTGTCACACCAGACATAACGTAATTTGTAACTCCATTTTGATATTG

CGTTTTAGTAATATCACATGTACCGCACACACTCCTAAAAACACTAGCCAGTTTTCGACCTGCATTTCCA

GTACCACTTCCAGATAATAACACAGGTACAGGAAGACTTACTATGGGTCCTTGCATGTGCGATTTCCAAG

ATAATACATCCTGAGGACTTGAATCAACAGAATAAGGAAATATCGATTTAATTGAATGCGTTACGCCGAA

CCAATCAGATGCAGGGTGATTTTCCCAATAACGGTAAGGTTCATGAGGATTTTTATAGAATACAAGATAA

GATTCAACATCAGATGTTGACATGCCCATCTTCATAACATTCTTTTTATATCCTTCTGGTATAGCATCCC

AACCCATCGAAATTATAGCACCACTTGGACGCTCAATTCTAATCATCGAAACAACACTATTCTGACATTT

TGCAACTAATGCTTCTAATAAATCACCTTCATCCACAACATCAGTCATTCCCATTTCATATTCACAAATG

ACAATAACCCA

>gb|GAUE02012784.1| TSA: Anurida maritima C94214_a_4_0_l_2312 transcribed RNA sequence REO VP4

GCGACCAGAGGCGAACCCACTCATCGACCAAGTAGGGATGCTTCAAGATCTGCAAAGTGTTTTAAACTTG

CTCCAATTGATTGACGTACCCCAATCGATTCGGATGAATATGGCATTGCAGCGATCCCTTCTCGTGCAGC

AGCAAGTAGCGCATATCCTACCATTTTCCGATGTGCTGGATCAACTATTTGTGATAAAGGACTGCTCCTT

CCATAATGCATCCAATTCGGCAATTCTAGCCATGCTTCGGCAACCCATATACACGCACCACGTTGTGTCA

AAGGCATTCCATTCCGCTCTTTTATTCCAACCTTTTCTAACATTTTGAAATATCGTATTTCACTTAAAGG

AAAATGTTTCTCTTGAGCAAACCGCTGGGCTAATAACGTAACATAAGGCATCATCGGAGCTACAAAATTT

GGCCATGCTTGTGAAACGTCTCGAGGCGAATCACTAGTAAATGTTAGAGCATCAACCTCACCGATAAAAT

CAAGAATACTTTTATCAGTAACTCGTTTTCTGTTGAATATTAAATATTTGTCGATACGTTTAGGAGATAT

CACAGAATGTCCTTTATCTAAACCAAGTTGAACACTAGTTACCGCATTTGCGTTCTCAGGAGTATCAGTT

GCGACAACAAATGCAAACGTACCGTCATATGGACCATTTCCTCCCGACGTTAACCCATTACGTAAAAAAT

CGGTAACTGTGGCTGCTGCTCTAAAAACGAACACACCATTACCTTCTTTGTGTAATTCTTGTGAATTAGG

TACTCGTGCAGGCAATAAAACCAAACTACCATTTCTGGATAATAACATACCAGTCCAATATTTTGCATTC

GGTGCTCCTTTTACAGCCAACGCCAGCCAAGCTTCAGTGGTAACATTATGATCGCTTTTCAATTCATCAT

CTCCAAAATAAATAGGACTTACGTTATATGAGTAATATCTTCCAGTAAATACCGTTACTTGAGGACCACC

TACCATCTCAACCCTGTAATTGTTAACAAATTTTCCATTAATCACTCCATCAGCAGTGAATGTAGCATCG

TTCGCAACCATTCCATCTTTTATTGGCATTATGTCAAACGCATCTACCGCGGAGACAACGTATGAAGTCG

AGGGCATGAAATGGTCTAACGGTCCCATTTCTAAAGGTTTTAATACAGCCAAATCAGTGTCAACAACCTT

CATAACTTTTGATACCTTCTTTGTCGTATCTCTAACTTTGGCAACACTGCCCATTGATGTTTTAGTCATA

TCAACCATTCCTTGGACAACTTGAGGACTTACTTCTGGCATTCCATCATTCCGTCCGTCTGATACACTTA

TTCCGACACTTGCACCTCCCAATCTGATATGGTCAACAGCCTGTGGAACTTTATGTTCAGGGATATATCG

AGCAAGAGTTCGAACATGTTCTTCAGTTGGTACAGCCACCAGTCTACTACCCTTAACAACGGCCATCGAT

ACATCAGCAGTAGCTTCACTCTCAATTTTCTGTGATAATTGCATAGCTCCCATCCCGTGTCCAAAAGCTT

TCTTCACAGAACCAACCGTTTGCTGCATCCATCCAGAAGCGACAGCGTCACTTTTTGATATTGGTTGACC

ACTTCCAGCAGTAGCTAATGCAGGCCTTAGTTTACCGACTAGCATATTTTTCAATTTAACACCCCGTTCC

TTTAGTGATCGTAAAAATTCGAACTGATCTGGACTTATCTCAGATTCACTTTCGTCTAACTCACCATCTT

CCTGATAATGAACAAATTGTTGGAATACCCGTAAATAATGTGCTGTCATCTCATCGTCTAATTCACGCGT

TGCATATTGAACTAAATCATCCAAATCTGAATCAAATGTTTCCATACTTCCATCGAAATACCGAGAATAA

TCATCATCATATATCGATGGTACTGCATTGCTAGTTCCAAACGCCTCTTGTTCTCTCGTTTCACTCTCCA

TCATACTATTCCTATCACCATCACCATTCATGTTATTGTTTCCTCCAGAATAAGTTCCATGCATAATCGC

TCCTAATCCAACTAATGATGTATCCATCAACCCAGACATTTCGTCAATTATCGCACTTCCAGCAGTCAAA

CCACTATTTGCAACATGACTAACAATCATTGCAGCTTTTCTCGCTTTTTCGATTCCAGTTTGAGTTGCAC

TAACAAAACCCGACACCTTATCTAACGCACCTCTAAACTTCTTGATCAAGCCAAATATCTCAGCATTATC

TTCTTCTTCATCGACTATTATACCCAAAAATCGCCAATATTCATCCATCGTTGAAAAAGAGACAAATGCT

GG

>gb|GAUE02013011.1| TSA: Anurida maritima C95222_a_45_0_l_2548 transcribed RNA sequence REO VP5

GTCGTGCTGTAATCGTAATCCTACCAAGCAAAATAAAGAGGTGTCTGTAATTTCATTGTGTGTCATATTA

ACCGAATATATACAAATTTTCGAGCTTATTTACTTGTAGTATATTCTGATAATATAACGTTGTCCTAATT

GTGTTACTAGTTATAATAGTTTCTCTCTTCTTTCACCTTTACATAATACTCAAGAAAATGGGATTGACTA

TCATAGTCATCGAGGATGATCAGGAGACGGGTAGTGTTAAGGTTGATGCTGGGAATGGGCGTGTGTATGG

GTATTCCGATGGAAATACTTGTCTTGATGTGGTCGGGAAGAATGGGAATGGATTATATATTCCTACTACA

AAGAAGGATTATTTAGATAGTGTTAGGGATATTCTAGTGTATGGCTTAGATTATGCTGGTGCTGAATGTG

GTATGTCTTATTTAAGTGGAATGGCTGAGGGTGGTTCGGTTGAATTTGGTCTTGCGTTGAATTCAATGAT

ACCGACTACTTTGCCTTGTACTTTTTTGAGTGAATGTTTGAATGTGCAAGGTACACAGCTTAATGCTGCA

TTGAGTTATCTTCATGAAATTCGCGATGGAGTGGCGAATGTGCATAGTGATGTCCTTGCTGGGAATGAAG

CGACAATTGAAAAGATAACTGAAATGGAAGGGAATGTATTATCAGCGTTGGGTGCGTATGGATCAGTACA

AAACGAGCGTGATAATACTCGAGCAAAAGCTGAGTTGTATGAAAGTGAAATGCGTGATATTTTAGTTAAG

AATGCTGTTGCATCGATTCGTGATGAAGTGAAGGATGGGTTGCAGTCTATTCGCGACGCGCTTGTTGAAA

AGATTGGTAAACGCGGGGATGTTGGTGTTATCGATGGAACTTCTTGGGCTGAAGAAGTTGAGAATGAATA

TTTTAGTGATAAGAAGGATGCGCTTGCTGTAGTTGCTGCACCGTCAACTTCAACTGCCATAGTACGACAT

GATTATCCTGCTGCTGCACAACAGAGTTTATTTGTCCTAAAAACTGACGAAATGGAGTGCGCTATATCAT

CATTCATGTCACAAGCTTTTTGTTTTGGACCAACTCTTCATTTTACTCTCACCGATAAGACGATAAGTGC

AGGTGCTGAGCGTGATCGTTTCTTGTCTCGAGAGTTTATTACTGCAGCTTATTACATCGGAGCTGTTGAA

TTAGGTGGTCCGGGACGAGCTGAAGATTCTGGATTTGGTGGATCATGTTTTGGTGATATTTTAATTAAGA

AGGGCGGGTGCCTGTTTCATGTTGAGTTGGCTTTAGTTAGTAATGGTGGTGGTCATTTCCTGTTGTTAGG

TAGATCTGGGGTTCAGGGGTTTCGGTGTATATTCGGGAATGATATGATTGAAGAATTTGATGGACACCGT

GTTTGTTTGGTAGCGTGTGGTCGGGATCAAGTGATGACTTGGGATGGACATAGAGACTCAAAAATCGATA

ATGTTCGTCCAATTGGAGCAGATGAAGTTGGGGAAGCTGTTGCGGATGTTCTTGTTCGAATTGGTGAGAA

TGAAGTCGAACACATTTTATCAGTTGGAGGTGATGGACGTGGTAATTTGAGCGTGATAGATTTGTGTGAT

CGAATGACTAACATAGATCCATCAATGCGTATGGTTAATACAAATAGTCCAAAATTGACTAGATGTCCAA

CTACGATGTACACAAATTTGCTTAACCATGGTGAGAGGAAGTGGGCTGTAGCGTTGGATTTGATTTTGAT

GGTCTTTCAATCATATAACGCATGTTATGTGAAAGGTGATTGTCATCGTCAACTCTACGAGGATTGTATC

ACCAGAGCGACGATGTCAAATTGCCTATCTGAAATGAAAGGGCTGCTCGAATGGTCAACAACATCGCTCG

GAACGATTAGTAAGTATTGGATTCGAACTGTTAGTGGAATAAACACATTTAACCTAAAGGGTGCATGGTC

AGGAGCTCAGGCTATTTCGATCATAGCCCATGAAGGTAAAGAGTACTTTCCCTGCCCTCAATGCGGTTTG

TGTTATTCAACTAAATTCGGTAGGCAGATGTGCTCAACATTAGATATTGGAATGATGAAATGTGTTCGAA

TGGGATTGGGTCATGGCAGAGTGACAACTGTGTTAGCAAGTCCGAACACCGACAAAACAGAGCTAACTCA

ACGTGAGATTGATGAAAGATCTTGGGATGCTAAAAGAGTGAGTGCTTTGCATGGAAAATATCTCAGGGTT

TTAACATCTCAACCCAGTGAGATGCGAATGGCTCGACCGACAACTAGAAAGGCGCAATATCAGAAGGAGA

CTTTAGATATTGGAATGATGAAGAACCAAATTCCAATGGCGTCTTTGATGAAGAAGTTGTCGGCTAGTAA

TGTTAAGAAATGAAAACATAAAACCAAAAACATAATAATAATCAAAAATCACTAAAAATATGAAAATTAA

AATCAAAAAACATTTAAAAATAAGAAATCAAAGAAAACATTAGTTAGTCACTGAGGTCCTGCTATGTCTG

GTAGTTGATTCCCACGACCATGGTGCGG

>gb|GAUE02012054.1| TSA: Anurida maritima C91152_a_9_0_l_1820 transcribed RNA sequence REO VP10

GAAGAATATACTCTTATTACCCACTGGAAGAAAATATGGAAAATTATATCTAAAACATTTAAAATTGTTA

AAAATTGCTGACTCGTTATGGAGAACCCTTTCCCTGGTGGAAGCCCGCATTAAGAGAAACATTAATAAAG

GACCTGGTTGGGAAGAACGATTGAAAGGAAGAAATGGCAAAAGTTCTGGTTTATTAGTTATTGCAAGAGG

TGAAATGGCGACTCATGAAATTTTATTAGAATATCGTCGTTTGGGAGGAAGTCGAGATTTTAAAACATAC

AATGGTGATTTCCCTAACTTCCATGAATCTAAGTTGTCGAAAGATGTTAGAATGAGGATGACAATTGCAA

ATGGAGGAAATGTAGTGCTTCGAGATACCACTGTTGAATTGAGAGACATAATATCATATGACGCAATATT

CATCACTAAGTTGGACGAAGTTAAAATGATTATTATGGGTGAAGCACATGTTGAAGATTTTTTGAAGATA

GGTGAGTGTTGTGCTGTACCATTTGATAGTCAAGGAATGTCTATATCTAAAGATGACATATGGATTTTAA

CATTACAGGGTGGATATCGATATAGTTACGGACGTAACAGGTCATGGGAAGAAGGATCACGTGAGGATTT

CTTTGGTGATTCAGAAATACAATGCTGTGTTAAGAAAATGATTGGTTGTGAGCTTCTCGATGATCCAAGT

CGTGCTATCAATAAATACTTGATTGAGTTGCCTGAAGATTTATTGACTGATCGAGAGAGACATGTCCAAA

TTAACGCGAAAAAGATTAGTAATAAAAGAAGGAATTATACTTCTGGTTTCCAAATGGTGAACATCGAATT

GGTGAGTAAATCAGTTCATGAGCAAGATAAGTTAATGCAAAAACTTGGTGTTCGGATAGGAATGAAAGGG

TTGTTTATTATCCCGTTTGACAGGGCTCCTGTAACATTAGAATTGTTATCTGGTGAAGAATGTTTTTCTC

GTTACTTAAAGTATGTCGTTGAATCAATAGATGAAAATGCGCGTTTATTGTTGTCGATTGCCACAAAGAT

GGAATGCACACTGAGAGTTCATGCTGGTGTCAACATTGTGAACAGTTACTACACTTTAAATCGTGTTTTG

ATTTCCGATTTACTTAAGATGGAAAGACGTTTGTACTTTTCAGCTAATAAAGGAAGTGGGAAAACTGTTA

TTGCTCGAGCAGTTGCGGAATTAGGATATAACGTATTCGATTCTGATAGTTACGGTCGTGTATTATGGTT

GATGTTTCAACAACCTGAGAATCGACGAGATTTGGTTACTTCTTCTTTGAGTTATGTAAAAATGGAATTA

GATGCAAAGAATAAAGTTCCGTCAATATTTGAAGAAGAGATGACTGCATCGTTGGAGAAGTTTGGTGTTG

ATAAATTTCCGAGTGACAGACGACGTCGGAAAGACGCGATTGAGGAATTTGCTGTATTTTATAACATGTG

TCTGTTAAAACATCGAGCGACAACGGTTCAGACTAGTTTAGCCAATGGTCTATTTGAGGAGAATGGATAT

GATGATTTAGGTAATCGGTGTTTTGCTAAGACTGTAGAAGAGTACAAGATATGTTATTTGGTTCATACTT

ATCCTGAATTAGTTGAGGCTATGGATGGTGTATTAGCTAGGTTGTTTACATGTGGACATTCAAAATTAGG

GATGATGTTGCGAGGACAAGGGTCTAATGTTATCGCAGAAATGCTACTGTATGAATACTATGACTCAGTC

GACCAACAACATGTACCTGGACTGCCGCTTTCGCTACTGTTATGGGTGCTAGAGCATTATTCGACCATGG

>QVQU01083516.1 Machilis hrabei contig_83516, whole genome shotgun sequence//Mono-Chu clade

TGTCAGTTTGATTTGTTAAGAAAGTTTAGATGTTTACAATTCAATTTTAATGAAGGCTGGATCTACAATC

AGCTTATAAACTTTGTTTTAAATATAATGACAAGAAAACTGTTGAGCACATTAACAGGAAATGGGTGGTT

GCCTAGCAGCTATCAAGTTCATAAAGTACATTTTATTTCATAAATAAAAACAATTTTGGACTAGAGGCCT

TTCATCCAGCTCCTTACTGAAGTTTATCATTATTCTACGATAAGTGAATATACAACCTAATCTGTAAGCC

GCTACTGACACAGCTATGGAAATGGAGCACTTAATCTGATTTGCCAACATGAAACACAGATGAAAAGTAG

CTTACATAAAGTTGCAATCATGGACTTCTGCAAAGACGTACACTCATTTTGTCCCTGGGCTAGTCATCAG

GGATGTCAAACGGAGTATGAAATTGAGTACACCTCTACTTGTCAATATGGAAACCTTCTGGGGTTCAAGC

CAGAACAAAACACATTTCCAGGAACTGTTCATTGAGTGGCTTGTAAGTAAGCGAAATGACGGCAAACCTT

TGTATTTGGGAGGAGGCCACAAAGATAATCCAAAGATGTGCTTGAAACTAATGGAAGGTTCTGTAAGTTT

TGCACCATCACTTCATTGTGAATGTGAGGAAGCTGATTACAGGATCATGAAACACATAGAAAATACTGTG

TCCACAGACTCTGCTGAAAAAGTCATGATTGCTTCTGCTGACACAGATGTGATGGTCACTGATCTATACC

ACTTCAAAGAGAGCTTTCAAAAAGGTGGCCTTAAAGAACTGTGGATAATCAAAGACACTCGGTCATCTGT

AAGGTACATACCTATTCATAATATGATGAAAATCATGGACAACCTCCTTGTATCTATTCTACCTGCTGTG

CATAGCTTAACTGGTTCTGATACGACAAGCAAGATTGGAACAAAACTTATGGCACTAAAAACTGCTGTTT

CATTAGGAAAACTTTTGAAAGATTTTGGTGTTACTCCTCTTGACGACCATATGATAAAACATGCTGATGA

ATTTATTGTCAAAGTACTCGGTTCATAAAAAAAAACTACGACGAAGTGTGATTTGAGCATCACCATCAAG

CTAAAACCCTATATTTTTCATAACTGTCCCCCCCTTCTTCAACAATGCATACCTTCAGTGCTCCATATGA

ATAAATGTGCTGAAAAAAGCAACAACACTAAACCCTTTACAATATGGCTACACCGTAGACGAAGGAACAT

TTAAGCCTGTTATAACAGCAGAACAAGTGAGACCTCCTGAATTTGTACCTCCGTGTACTTGTCAAAAATG

TGCACGAGCTACAGTGTGCATTTGTTGAGTTGAAAACATGCCTTGCTGCCAATTTTGTAAATGTAAAGCA

AAGATCTGTGTTTAAATGTTTAAGAACAAGTGAAAAAAAAACACAAAACGCTATACAACATTATGTTGTT

ATTTTCTGTTGCCTGTTAATTACAGTATAGCTACATTTGCTTGACAATTTATATAAAAAAGTTAAAATCA

TGTTTACTATTAATTATAGACCCATCCCATTTTGTGTGATCAGCGTACTTGTAAACATATAAAATGACAT

ACAACATCCCTATACTGCGCATAAATAAATTATCAAAAATATTTTTGGAAAAATTCAACTCACGGAGCTG

TTAAATGACAAGTCAGTGGCTCCGCCACGAAGCAAATTTGATGTTATATACGACACGGATCTCTTGGGAT

ACCGACCGACGCCTCTACAACCCAGTGAAGACAGAAGACTTCTAGTCAACTTGTTGAATCGGTCTTCTTT

AAACCCAGAGGATATATTGGAGAAAATAACAACCAGACAGGTTCCAGACAATTGGAAATGCGTAGGTATC

CACCCTAAGGAGAGGGAAGAAAAGAGCGGAAAACCTAGGTTATTCGCTCGTCACCCCAGAGATTAGGTAT

TATTTCTGTGCAACCTAGAAGAACATAGCCAGCTAGTTGTTTGAATATGTAGAACACCAAACAATGACAG

AATCGGAAAATTCATTGTTGCACAGAACACTGGATACCACGCAGGAACACTCTACACCAATAAAGATTTT

TGTCTCCTTAGATTTTCAACGGTGGAATCATGCCTGGGACTTCTCTGGGACATGTGCAACTTTCGAGATG

GTAGATGATACATTTGGTACACCTGGACTCTACACCTACACATGAATTTTTCGCAGTTTGTCTGTGTTAC

TTATCATCTCGATTGAACCCACCACCAGGACTGTCAAAAGACCATGCAGGGAATCCCCCAGACGTCCCAG

GCCACCTCTGGTATAATCATAAAGGAGGGTTTGAAGGCCAGCGCCAAAAGTTGTGGACCTTCTTAACTAA

CGGGTTGGTGCTATCGGTTGCCATTGAGCTGAAGCTTAAATGTCTCCTGAATAGGTGTGGGGACAATCAG

CTAGCGACGATCCTTATCCCCAATAGTACAACAGACAGGACTCCCCAAGAGGATCTTTCATGTAGAAGGG

AAGAAATCAATCTATTATGTGAGTGAATAATACAACTCCTCCAGGAGAGGGCAGCTGGTACAGGTCAAAC

CTTGAAGGCCGAGGAGACATTTTTAAGTGACACTGTGTATATATACGCCAAGGAAGTAGTCATTAGAGGT

ACAGTCGCCCCCTCCACATTGAGAAAGGCATCACGAATCATGGAAGACACCAGCGGCACTGTTAAAAGTT

TTGACAACATAGTGGCAACTATCCACACTGGTGGCCACACTGCTGCACTGAAAGGTCACTCTTGGATGGT

GGCTTATTTCCACTCGGTTATCGAAACCTTTCGAGCCTTATACCATGACATCATGCCATCTCGCGGGAAA

GTCATGGGTCTTACACTCAAGACTCAACAAGAAGTCCTAGAGGTGGTAAAAATCCTTTGTCAGTTACCAT

CCTGTTTTGGAGGCTTACCATCTCTCCCCTGGACGGATTATGTTGTCAGGGGGCATCCAGATCCTCTGAC

CAGTGGGATTTTAGCTCTCACCTTGATTGACCATAGGATATCTCGATTGATCCTGAACTACATCTATAGC

CTGCTATTAGAGAAGAGAAATCCGAGGTCTTTGCCTCTAAATTTGGTCGAGGACCCAGCCTCTCTGGATA

TACCCGGAAAAGGTAAGACAGCGTCGGTTGTTCGACAACAACTCGAAAAACACCTTCCTTCGTACACTAG

GAACCCTGATCTCAAACAGCTCTTCAAATCTGCACCCTTGTCTGAGGATCGACAATGGAAACCTCGGGTG

TTGAATGCTATGTACAAATCCTCGCCGCACGGGGCCCGTAACAACTTCGTGGCCACCTTTAATAATCTGA

ATATGGTAGACAAAGTAATGAAGACCTGTGAGGCAGGACCCATATACTCCATGGTTAAGGAAGAAGAAAG

GAGACGTAGAGTTAACCTAGGAGTCATAGTGCAGCGCATTTTCAAAGCTCCAAAGCTACCTGGTCATGCT

TGTGGTAGGATATTCGCTGTTGGTCTCAGGTTGCACCCAAGTGGACTCCATGAGCCGATCCTTGGGGTGA

CGGTTCCTCACCCATCGGAGGTTTTGTGTAGGTCTGATCCAGAGTATTTCGAGTTCATCTTACTGATGTG

CCACGGCAGCCAACACAAGGACACAGACCTGAATATTCCATTACCATTCCTGAACCGGGGGCCGTTTAAA

CCTGAATTGGGATCCGCAACTAGGGAGAAGAGTTCGTCAGAGCTAACAAATATGGAAAAAGGTGATCGCC

CCTTAATAGAGGCCTTTCGCTTATCAAAGATCAAAGATTGGGTCTCTGCCCCCAATTCTTATGTCAACAA

GTGCATTGAATATCTTCAGAGGTTACTGACATCTGCCGATTCAGTGGTCATAAGCTTATTAGCTAAGAAA

ATTGTGGGAGGCAGCGTTCTCCATAGGTTCATGGATGTGACAACTAGCCATTCTGCAGTCATCAATTCAA

ATCCCTCACTCCAAACCAATTTGACCATTCATACTGACTGCTTCTCTCCATACAGTCAGTCGGAAGTGGA

CTACAATATCCATTTCCAGGGGCTTGTACTCCATGCAGCTGGGTTAGTTAGGATAATGGGTCTACATGGC

TATGACATGAGCCTGAACTGTTTCACCATATCATGCAGTAGTTGCATCACAGAGCTGCCAGAGGTGAAAC

TCAACTCTTCCTGTCAAATCCCTGCTATGAGGATTATTAAGAATCCTCTATTTTACAGCACTGTGGAACA

AATGGGGAATCAAGACACGATCTCGTTCTTGTCTAAGAGGTCTCCTTTTAGTCAACTTCGCTGGGAGGAT

CAATATGCTTGTGGCCATGCCATAGGGGCAAGGGTGTTTCAGCCATTATCCTCCGCAGTGCGACATGAGT

ACCAAGGTGTTGAGTATAGCTCAAAGTCTTTAAATATCAGGATACAAGATGTTACACATGCTGGACTAGA

GTCAATATTGTTGGGCTTCTTGGACGCATTCATATATAGGTATTATATCCAGATTTTAAGAATTGCAAGA

GATTCTGGAAACCCATCTCTATCCAGCACAATTGTAGCCATTCTACAAACCAATAAAACAACAATATGGA

TGAGACATCTTGGCAAATTTCTCCCTCTCTTAGCGAAGGCTTCTCAGATCCCAGAGCCATGTCTGAAAAT

CATGCGAGAAGACTCTTCCTCAAGTATTGCCATTGAGCGGTCCACAGAGTCCATCACTACTTATTTATGC

GAAAACATTGACAAGTTTTTGAAAACAATCCTATCCATGAATAGATCCTTTGTGACATATTTGGAGTCAG

TGCATGACGGAGATGCCTTTTGCCGTTTTATGGTGGTAGACCACCTATTGAAACTCACCAAGAAGGATAC

CATTTACCCGGATAAAGCATTGAAGATTTGCAGAACAGCATTGACCATCTCTCCAAACAATTGGCTGGCC

ACTTATTCCAATCTCGTACACTCTTTTGGATGACCAGAGGGAACATTAGTGGTAGAGAAAACACCAGCAG

AAGCTGTGATGAGTTCCAGGGGGGCTCAGTTGTACACCCACCTGTTGCATCCGTTGAACAAGTGCAAGAA

CCTGGAAGACTTCCAATACTTTATGTAGATCAATATGACACATCTGGTATACGGGTTGAACTTCATGGGA

AATCCGCTACAATCCTAACTTAGTTAGGAGGGAGGAAAAGAGAATCTTCTCCAACTAGAAAAGATCACAT

GTACAGGTTGTCTTCTTGCAGCTCAACCTCCTTCTCTTAACTCCTCCATGTTTTAAGCTCTGAGGGGTTA

ATGCATATGGATCCCCCTGAATCAATTCTATGCACTGCGGAAGGCAACGGGTCTATGATGGCTACCCTTA

TCAAGTTATGGACTCCCAAAGTTGCCCACTGTAATAGACTTGTCAGTGACTTGCACATTAACCCAGAAAG

ACTAACGTCATTTATTCCACCTTGCTGCCTGGGCCTTCAGCAAACCACAGACATTCAAGTCCTCCCGGGC

CTAACCTACGGTGGGGACCTTCTACACAGAAAGCTGATAAATGAGTTGTGTGATTTGTCCCCTCATTTTT

GTATCACTTGTGATATCACTATCTCCTCAAGTGATCCAACTACAAAGGTGGTAACTTTAATGACACACTT

GGCTGTTTTGTGTAGCCGTACTCTAGCTGATGATGGCTTTGCAGTTTTTAAAGCTTATGCACGGTCTGCT

TTGGCTCTCTCAATATCTGTAAATATCTTGAAGAAAGCATTAAGGGTTGTACATATTGTTGTTCCAGAAA

TGGCCTCTTACGAGAGCAGTGAAGTTTATATTATAGCTCGATCACCTATACGCAGAGATAACCCGTATTT

GAGTGTTGAATTTCGTGACACTCCAAAGTCGTTGAGGGAGTTCTGTCGTAGAAGAGTTTCTCATACGGAG

CCCTTCCAAATGAATCATCCTACCGCATTGTTTATCATGAAAGCCTCTAGACGTCTACTGGACTCTAATC

TACCACACATCATGAGTGTTATGTGGGTCAAACATCTCTTTGGACTCGGGGGACCTGGAGGCTAGAAGAG

AGGAAGTTTTCAAGGTCTTGGAAACACGACACAAGCTGATGGGCAAAAGACTATATGCAACCCCCCTGAG

TTACCTGGAGAAAGTAGAACTGACTAGGGAAATAGGAGAAAAGTTGTGAACATCAAGCCCGATTGATTAG

CTGATTGGGATTGCAATGTGCCTGAAAGCCCTACTACTTTGGGTTTAGGAAGTGGGAGAATATCAGAGAC

TTGATGATTAAGGTGCAAGATGGGCATAGTAAGTGTTTTGTCATGACTATATCAGGAATTGACACCCTTT

TAATTGCAGGCAAAGTGGTCTGGTTTCAAGAAAACAATTGTTTGATGGATTATGATCAAATATTAATGCT

AATAGAACTTTGTACTCCAGATTTCAATCTCTAACTCACATTTTGTTATATAAGGGCTGTCACGATGTAA

CCTCTCTCCCTTAAGACGTTTTGGTGCAGGTGTATCAATGGGGTGATAGACTCCTGAGAGATCATAACAA

CACTGCATATGACACATTCAAGATGTTTGAAGCTCTGATTACATCTGTTATAATTGCTAGGAACAGCACC

TTGCCCATTTCTTATCGGTTTCGGGACTCCATGGTTCAAGAAGTGGTCACTCTCCATGAAGCTTGGCCAG

AGCACCTTCAGATTATAGAAATTTTTTGATGGACTTAGATCAGACTTATGGCTGGTAGAACTTCAAAGCA

TTTTTAGGCATTGGGGACACCCAACTGTCGATGAAGTGGGAGATGTCAAGTCCGTTGTGTAGATGGGTCA

TACTCGCAAACCAGCAAACCTCCCAGATGTATTAAAAATCCATGCTTCCTGCAGTAGAATTTTGTGTCTT

GCTTACATAAGCAAATATGCCAGAAGGTTCAGTCTTGAAATCTTGAATTGATCAAGCCATAGTAAACATT

GATGAGTATGCGGACGGATACGAGTGGGAGGACTTTTCGTATGTTTAACATTGCAAATGGCTAGAATTCG

ATCCGACTCTCGACTACAAGTCAGTGGCTCCGCCACGAAGCAAATTTGATGTTATATACGACATGGATCT

CTTGGGATACCGACCGACACCTCTACTACCCAGTGAAGACAGAAGACTACTAGTCAACTTGTTGAATCGG

TCTTCTTTAAACCCAGAGGATATATTGGAGAAAATAACAACCAGACAGGTTCCAGACAATTGGAAATGCG

TAAGTATAAACCCTAAGGAGAGGGAAGAAAAGAGCGGAAAACCTAGGTTATTCGCTCGTCACCCCAGAGA

TTAGGTATTATTTCTGTGCAACCGAGAAGAACATAGCCAGCNAGTTGTTTGAATATGTAGAACACCAAAC

AATGACAGAATCGGAAAATTCATTGTTGCACAGAACACTGGATACCACGCAGGAACACTCTACACCAGGG

GCGCCAATAAAGATTTTTGTCTCCTTAGAATTTCAACGGCGGAATCTTGCCTGGAACTTCTCTGGGACAT

ACGCAACTTTTGAGATGGTAGATGATATATTTTTGGTACACCTGGACTGTACACCTACACATGAATTTTT

CGCAGTTTGTCTGTGTTACTTATCATCTCGATTGAACCCACCACTAAATGTTAAATATACTTCCACTTTT

TTTAGGTTAGAAAAGCATTAGCTTCATTTGATTTCATTGGCGAATTCAAAATATACAACATAGGGTTAAA

TATGATTTGAATGAGTGATGACAAAAGACCAGTTATTTATTTAGATGAAACATGGACTCCTAAAAACTAT

AACGTGCAAAAGTATATAATATAATAATAAAGGTGCTACTGTCAGTGGTGTTAATAAAGGTGCTACTGTC

AGTGGTGTTAATAAAGGTGCTACTGTCAGTGGTGTTAATAAAGGTG

>QVQU01249695.1 Machilis hrabei contig_249695, whole genome shotgun sequence//Mono-Chu clade

CGAACTCGGGAAAATCTACATCTTTTACGGAACACAACGCAAAACAGTCTAATTCTACTAATATTCGATT

ACATAAATTAATCGTGCATGTTAATCGTGCATGTGAATAGATCATGGTATTAAATCAATTAATCAAGCAA

GTAGGTAATTTGATCGGCACCTGGTTCATGTCTCGTGGCCGATGTGAACTAGGAACGCCATGCATCACGT

TATAGGTGCACTAAATTTCACCACTTGAATGATAGTTTTTTTTACAACAACACTTAAGAGCAAAACACAT

ACACACAAGCTGTTAAGATAACAAATACTGGCGCACTGTTCCATCTTTCAGAAAACTCCTGGAAGCGTTC

TTGGTATGTATCGCTAACACTCTAAACTCACCTGTATATTTCATCAACTTGTTTTGACTCCATGCAACTG

AAGCATGGAAGCTATTCTACATACATAGCATACTTTCTGGACCATGCTTTGGTGCCTGACCTACGACTCC

ATGGTTCGACTCTGCATTTATTCTATGGAATTTCAAGCAGCTGGAACTGCTATAAAACGTCGTAATATGT

GAGTTATGAGTGCGAAAAACTTTAGTTTTGCAAACTGATCAGTGAACATTAAATGTTTCAGTTTTTTTTT

CAAAAGCAAGGACAGTGTTTACCAACCAAGTGTATATATAACGTAGGGTGTGGCAGCATATATTTTTTCA

AATTGTTATTGCAGACTTAAATATTTTTTTCTTTTCAATTAATGCTTTCTTAACCTGCAATTTTCAATTT

TGTTCTATATTTTGATTTGCCAACCCTAACGAACAATTATGAACAATTATCTACGAACAAAACAATGTGT

ATTTCGGAAAAAAAATATTTTGTTCATGAAACAATCTACAATCGATTTATGTACAATTTTGAGGTACCAC

GCTCTACTGTAAGTATGTTTTCTTTTATGTTTGAATATGTTTTTTTGGACTCCTAGTTGCCTACAAGCTA

ACCAACATACTTTGCACTCGCGTCTGTATATAAAAGTAATTTTTATAGGTCCTGTAAGTGGGGGAATGAT

GTAAGAGTGTGAAATTGTCTAATTAATTACGTACTTGGGGAATCATATTTTATATCTAGTATTCACTGTC

TGTGCGAGTATGCACTTTATTAATTGTTGAAATGACAACGGAAGTCCGGCTATTGTTGAGTCGTAGGATT

AGCAACTCAGTGAGTTGGGGAATTTGGTTAATAAGCAGTTACGGAGCCTCGATCAGAATATTGAAGTATG

TCAGTTTATTGCACACAAGGGCATTATTCTTTGAATAAGTAATGGGGTGAAAGGCAGGTTAGGAACTTCT

AGTTTAAGACTTGTGACTGACGGTTTCGTCTATTGACGGACGGAGTCGTAAAGTAAAATCTGCTGCTAAT

CATTACCAGTGCCTCGGATGCGTAAGGTGTAGTATGTACTTGACTAAGTATATCTGCTATTAATTATTGA

AATTATTTGGAGCTTATTATGCTCAAAGTTTAAACGAGGCCTGTTTTCTTATTCTAGTTATTGACACTTT

CTGTTAATGTGAGTTGCCTTTTGTTATAACTTATTATCCGTGACTTTTGTTGACATTTCATATCGGAAGT

ATTATTATTCTTGAATACAAGACATTAATATAAAATATTGGTTATGCTTATAGGTCAAGTCATTTAAAGT

GCTGTTCAGTTTCTTGTACAGAGCGTGGCTTTTGTCTGAAAAGAAGCAAATAATATGTAACCATTTTTAA

TCCGTTAACTGTTGCCTTGTTCTTATATTACCTTTCCGCGCATGGAATGTGCATAGTAATCAGTATTGCT

ATTACTGAGATAAGCTCAGTTAACATATGTTCGTTGAACTAAAACCTTGAACTACAGAGCGTGTGACTTG

CTAGATGTCGTGTTGAGTTCCAATCCACGTAAATGAATAAAACTATAATTATTTAAGTTTTGAACTAAGA

GTCATCATTGTGTGCCAATTGTCATAACTTATGTTATGAGTGGAGTATCTTTACATAGATTGATATAACC

AGTGTGTTGATGCAAGTAATTATCTGTTTGCTCCGACTCGTAAACTAACTAATATATTTACGTTCATGAT

TCGTTAGTATATTGGACTCAAGTAAGGTTAAAGCGAGGCACACTTGATTATATGACATAGTGTCATCTAG

CATTTATCAGCAAACATATACTATCATATCAAACAAACTTTATGTGGTGTTCTATTGGCCTAAGACCCTG

AGATTAAGAAGTCTTGTACAGTACTCTAAGAGCCGAAGGTGAGGACAACACATTTACACGATGTATAATA

AATTTAAAATCCAATTAACTTAAATTCAAACCTTTATATTAATTTTCAGAAGCCAATTTGGCTTTTTTTT

AAAGTAATTTCAAATAAAGGTGATAAAATGGGAACATCAAACACTAACTTTAGTTAGGAAATATCCTATG

CCATTACTCCGAAATTTGAATTTTGTTTTGCAACATAATTCCCAAAAACGAAAGAGAGGCAGGCTGTTAG

GAGAACGGCTAGGCCTCTTTTTAGATATTTAAATATGTGTTTAGGTTTTTAGTCACTCACCTTGAGTGAG

AACTATTTTATTGATAAGCATCTATTTAGCTTACTACTAATTGCTAGCTTGTAAGACTGTGAAAGAACTT

TGCAAACGATCCATTATTGTCAGCATTACGAAATGCATATAAACTTGACAGCGGATGGAGAAAGGGCTGG

TTGGTTAGTTAAGGTGTGAGAATGTATCACCATTGTTTTGTCTCGTGTGTGGTTTGAGCAGCACGTTTGC

AGGTAATTATATTTGTTGTATTATTGTTTGTTTATTGTTGGATATTTAAGTATTTGTTGTTTAGCACAAA

TACTGGATTGTAACTTTGATGTATTGATATTCGTTTGACGCATTATGCGTAATATATTGAATTAACTAAA

ATCCATTGAACTATTAATTTCATCTCCTATATCTTTGTCTGGCCTAGGAATAGCTTAGTGCCATAATATA

ATATATACCAGTTCTAAGTACAGATTCCTAACAATTTTTTATCAAACACAAGGTTTAGCCTCCAGTCTTC

TTAGACCCTGGCACCTCTCCTGGACTTACCTGGTGTATTTTGCATAATAAATGGATCTCTGACAGAAGTT

ATCCGATCCATAAGTATGAAAGTCTCTCAGTTATCAACTTTGAAACAGTCTCCCTTCAGCCTTGCTCCCA

CTTTGACTATGTTGATAGTCAGATGCTTTTGCTAAAGGACACAGCTTTGGCTTTCACAATAAGTATATTA

GAAAACCCTGAGCTTCATGCCCGACCCTCATTGAAAGAAAGACGGACACTTCTCTTTTTCTTGATGACTC

AAAACCCTCCAGCTGAGGTTAGACGATATATGGACCAGTTTGTGGAGAAAGATCCATTAGCATTAGATTA

TTTTGTTATTAAGTTAACACAGAAAGCAAGAGATCTCAAGGTTGAAGGAAAGCTGTTTGGGCAGAGCCCA

TATTATGAAAGAGCCAAGAGATGTGTAGCAGAGAAGAATGTATCCAGGTTGATGAAGCGTTACAACTCGT

GTCAAGCAATGACTCTAACTGAACTTCAGAAACAAAAGAAAATGTATATCATGTCTCAGTTAGCTAATAA

GATGAAGGATAGTTATGTAGTCCACTTCTCATTAGATGCTGAGGGCTTTAACAATTGTTTCAGACGAGTT

TTATGTGAACCTATAGGTCGAGAATTTTTCGATAGGCTTTTTGGTGTGAATCATTATGCGCGAGTGATGG

ATATTTTTTAGCAGTCTCTCTTCTGCTGTGATGAGTTTTTCAGATATAATTGGACGGGTCAAAAAGGAGG

GATTGAGGGACTCGCCCAAAAGTTCTGGACCTGGATCTATGAAGCCGTTGCCAGTCGGATCGCGTCACTG

ACAGGAAACAAGTTTTTTGTTATGGTGAATGGAGACGATTTGAGAGTAACTCTCATTATTCCAAAAGAGA

CTGTATCCTTAGCCCAACTTTCAGACCTCATAACAGAGATCGCTCACGCATTCCAAGCTGAATATGAGTC

CTTCGTATTCAACGTCAAATTAGAAGAAACATATGCAAGTTCTGTTCTCCTTGGGTTCGGGAAAGTTTAT

TTTGTTAACTCAGTGTGCTCCTCAACGACATTGAAAATGGGATGTAAGATGCATGGCTTAGCAAACCTCA

TAGGCGACCTTCCGGTGGAATATATCAAAGGTGTCATGTCGGAAACTATGGCAACCATAGGTGTCAGCAG

CAATCATCGATTCATCTACATATTATGGCTTGTGGTGGTACAGTTCTACTTGGAGAACTCACTTGGTTCG

TTGTTTCAAAAAATGTCGAAAGATCAAATGGTACTGATGCTTCTCTTTCCATCAAACTTTGGAGGAATTC

CGGTTCTTCCCTACATTCGGTTCCTGGCCAAAGGAGATAGCGACCTAGAAACTGTCTGGATTTCTCTTTA

CAAGCACATTCAGACATGTGATCTCCCCATCTTTCACAGATTAGTCTATTTAATGTCTGTGTGCTTGAAG

TGTTCAACACAATATCAAGGCTTGGCATCAAATCCGTTTTCCCTTCCGCTGTCTACACCATCTGATGGAA

TTGCTGTCCTTGAAGGTGCAATCAAGAGTGTGTTACCTGGACTCGTGGAGAACATTGAATTTGCCAAAAT

CATTCACGCAGCGAACCTGTGCGCACAGCTTTATTTATTCAAGCACTATTCTCATCACATATCATTGAAG

CCAAGGCGTTCAGTGTTCTTTTCGAGACAAGTCCACCTGGGCAGGTCATAGAGTTAGCAAGCAAGTTTAC

AGGAACCCGGTCAGTATTCAACCTTCTACAAGTCAAAGGAAGACGTCATCGTGGTCTCAGAGTCCTTAAA

CGAGCTAGAAAGGAGGATCTTGCGAAGCTCTCATATTCAGCCCGGATAATCACAGGGGAACTGAGAAATT

TAGTAGAAAACAGACAGATTACCATCATTCTGTCAAGAGATCGGTGTCCAACGTTGATTTCAGAACAACT

AAGAGACTTCCTATGGCAAAAAAGAGTCAAAGGGTTTACTTATCCTTGCTTTCTTGACCAGTTCAATTTT

AGAAATTGTAGAGACATGTCATTCACTGGAAAATCAGGAGGAGGATTCAACCACAGCACAGTTTTAGTTG

CGGCAAGAAAACCAGGAGAGATCCCTTGTTATAGTCACGGTCCCTTCCCTCAGTACCCAGGATCCCACAC

TGACATGAAATTAATAAAATCCGCTGTTGAAATTCATGGATCGTCACCAGGGGTGGGGAGAGTTACCAAA

ATTCTCCAAGTCTACCCATTCATGGTAAGGTTCGGAGAAAGGGCACTGGTATACCTCAATAGGAGTTTGG

AAGACATCACAGGAATTAAAGCAGAGTTATTGACAGCAGCAAGTTACAGAAGTTCATCAGGCTCAGTAGC

TCATAGGGTGCCCTTAAACCACTGGTCATCAATGGTGGGTCCGAATAAGCTACCCAATAAGACAACCTAT

GTAAAAGTGATAATGACGACTGACAGGAAGTTGAAGACAATAATTGCTGATTATCCGTTGAACTTTAATT

ACATTAAAACACTTTTCATGGCTCTTGGAATGTACCACACAGAGTTTGGTTCTCATCATCTAATGGATAC

AGAGAAGATCTATTGGCCCGAGCTTCATTATGACTGCGAGTCCTGCATGCAGGAAATTGTTGAAGGAGAT

ATTGACTGGTGCTTGGACCCCCCAGATGTCACATTCGGTCATTGTCCAAGATCATCAGCAGTGGCTAGAA

GACTTTCACGAACAGATTCAGATTCTAGATCAGGCTTTGGAGATGGAAGGAAATGAACCTGATCTAGAAC

CTAATGACGGACACGGTCACTTGTTCGGCTCTGAAAGTAGGGTGGAGGATGTCTCACTGTTGGAGTGGGA

TGATCACCATGGCGAAAACTAGACTGAGGCGACCGCAGGGGCGGGATTCAAATATTTTTATTTTCCATCA

ACAATTTTATATACATTTTTTTCTTTTTTTTTCAAAATTACAAAATGTAAAAATTCAAAATCCAAAGATA

AACAATATTGGTACACATTTGGTTCATAACACACCTTTTTAAGAATTACAAAATGTAATTGTTTTTTGTT

TTTTGTTTTTTTAAGTCACATCATGACGTAACGACAATTATTCAGTCAGCATTTTAATAAAATCGTGTTC

TTAATTATAGACATAAACAACTTCATTAAAAAACTCTGTTAAATATGCTACTGACAATTAATCTTTCATG

AACCAAACGTTTTTTATGCTACTTGAGAGTTTGAAAACTCAGTTACCCAATATTTTCATTTTGAATTTAC

GTAATGAAACATATCGCGGACAACAAACATTTTTTTAATTAGACACTTTGTACCCTACATTAAAATAAAC

AAGTAAAGAGTAGAAATCTACTCATAACGAATATCAATCGCTTTAATTAAAGCTATTATCTAAGATTTTT

TAATTTCATTTTCATCGCATGACGTACCATAAATTCAAAAGAAACGTTTCTGGTCAACGCCAGTAACGAA

ACATTTGGAGGTGACATACCTTGGTTTTATCATGGCAGTATACCATAATAAATGACTCTTAGAATTTATT

TTATTTTTAATTAGAAACAAAGAATTTTCAAAATTATTAGGGACCAAGGGTTGCCGTCATATAACAAATG

TAGCGCGTTTTCTTTTCTCTATTTTCTAAAACGAGACTCAACATTGTTCTTCAACTTCGAGGATTCCTTT

GACAAGTAAATAAATTTAATATTGTGAAGTCAGTTGACATAATAAATTAAATTAGTACTTGTAACTTACT

AAATCAGTAAACCGACGTTTCGGTAATTGCAATTAAGTTAATAGATATTTAGAGTCAAAGGAACACAAAA

ATGTAGAGATATTAGCTAGTATTGAGCAAATTACTGGCACTCTTTTCTATTTCATATAACGCCGTGAAGC

GTCTTGGTATACACCACTGGCACTGCGTTATGACGGAAAGACTTCATACCATAAAGTTGCAAATGGATCC

ACTTATGATACACAAAATGACGTCATTGAGAATTACGTATGTACAAAATACAACATTCAATAACAAACGC

CGTTTTTTTCATAAAAGTGCAGTGCATATACTCTAATTTCTTACCTAAGCAGTGAACATTTAAATGCCTC

AGTGTTTAAAATTAATCTCAATGAAGTGAATAGCAAGTGAACTTTATTTAATATAACGTTAATTAGATAG

GACAATCAAATTTCCAAAATTTTAATGTCAATAACAAGACCATTACAGACATGTATCTACGTTTTCAATC

AAAAATGTAAAGCAAGTTCGTAACTCATAAATCAAAATGTGATTGTAACAACATTACTACATGATTAGAC

ATCCTGTGAGGTGTCATTTTGTATATTTTAGTGGCTCCACACCAAAATTTTCTTTGAAATCGCACATTGT

TGTCATGTTTCTCTTTTTTCTCACATTTTATCGTTTGTTTTTAGTGCGATTAATATGTTGGCAATCCTCA

TACCTGCTAGCTAACCAGATGCCATTGGCATTATGATATCAATCCTAAAGAAGGGGAGTGGTGTAGTGAA

ATGTGACATAATTTAATATAAATTAAATAACATTTTACTGCACTGTATGTCAATGATTAAAAACAGCAGA

AGTCCGTCTCCCCACATGATAAAGTCGGCCAAAGTACACCTTTAGCTCGGTGCCCTGAGTATTGTAATCA

CATATCAAACAGTGGAAGTTTTATAGGCGCTGAATAGACAGTTTGAAGACGATCCACTAAACCGACAAAC

GATAAGACAGGAGTCGGGAAATGACTCATGCGTGCGTGTGGAAACACGTTTGAAAACACCATCCTTGGAA

AAGATAGTTAAGCTTTGAGCTTGAGTGAAGGAAGTCGTTCCTGGTGGCCTCGGATGAGTGTATGGTAATG

TATGTCATAAGACAGGTTTTGATTTCTGTGATCTTTCCATAAAAGTCAAATTTGTACGGCACGAGTGGTA

TCGCAGTCAATCAGTTTTTCTGAATTTTATTGTATCGATATGTGATTTCAATCAGGAAGCGAGTGTGACG

TATACTTCAGTAAATAACAAATGTGCTAGACGGTAAAATTTAATAGGCTATGATGGTCATAAGCCATTTT

TATAAGTTTGTACGGTGTGGAGTATGTGTTGAAAACATACAGTAAAATTTACTAACTCAATCGGTGGTTT

TAATACAATAATTTTCCCTCCTTGGTAATAGCGTGTGTGACTGCACTCTGTAGTCGGGTTGAATTTCGCT

GACCAGGCAAGGTCAGCGGATGACGAACAGCTGGTTACTGCCTCTTGTTAAATAAAGCCCAGAACGAATC

TTCAACCATTAAAAAATTCAATAAGTAAGGTTGTTTGAACACAGTTCTGAATCTAACCTTCAGAGTAATT

ATTAGGCTAACAAATAGTTGCCGCCTGTGTACTCGAAGTGTGGATTATTGGTTTTATAACACTTGGACAG

CTGACAGCTAGAGAGGTTCGTTTTAAAG

>GASN02036638.1 TSA: Thermobia domestica C218246_a_5_0_l_601 transcribed RNA sequence//partitivirus

TGCCAACTTCTCTAGGATCTATGCCTATCTCCCTAAGATACGTTAACGTATCGTAGTTACTACGTAAGGC

TGTCATCACATCGACCTTCTCTAAATTCTTTCTAGCCATGTAAGCCATAAATACTCCTGCTTTCCCAGGG

TCAAGAAATGCAAACAACTGACCTACTGTGCAAATTGCCATTCTGGTCGACTCAGTCCTTGACTCGCACT

CCGGGCGTATCCACGAAACAACACCACTTGCCTGGTTCTTCCACGACGCTCCTTGCCTATAGTGAAAGCC

TAAAAAATTGACACAATTTGGTCTGCGAGTCACTACACTCATCTTATCTGGCATTATCATCGAGAAGAGG

CTTTCGGCTATCTGTGAGTTCTTCCGAATATAATATGGTTTATTTGTTACAACAACAACAAAAGTCATCA

CCCAGATATAAATAAGCATCAGTCAAACGACTTGTTATCAGAAGCACACACGTACGTGTTACGATCATAC

TTACGATTGAGTCCACAATACTTGTGTAATAAGAGCCTGAGGGCACGACCCGTGTCTTTTTAAATCTCTC

GCCAGTGGATAGCTGAACGACTGTCTCAATAAAAATAACGC

>QVQU01337473.1 Machilis hrabei contig_337473, whole genome shotgun sequence//partitivirus

TGCCTGTGGCTGGTTCGTTTTCACACTTTCTCGTGGACCGGTGCCATTTTCAAAACTGTCCGAAATGCTA

TTGCAAACTGCTCGCTGTACATACAAAGTCGCATTGTCTTCTTCAAGCAATGCCATTTTTATAGCGTCAT

CCAAAGTTTTGGGTTCTTTTGTCAAAACAAACGGGCGAATGTGTCTTACCACTCTCGTTAACGCTCTACC

TCTGGTGGCTCTTGTTTCCTGTAGTCGGGGAAGCTACCTGTGTACTTTTCTTCGTCCAACTCTTTAAACT

CTTCGAGTAATTTTCTCATGTTCCACTTCTTTTTCACAGGACATCTCTTTTCTGGTCTTTGAGGAGGCTC

TACCTCCAATAACTTCCATCCAGGTGTCTTCGGTATATACAGTTTTCCTGGGTCTAGTCCAGCCAGTCTT

ATTTACCTAAGTGATCCTGGATTCTCTTTCATGAATGTGTCGAACCAATCATGAGCGAATTCTAACTCAT

GTGCCATATCATGAATGATGTCCAACCACCTTGCTGCTGACATTGCATTCAACGTACTCCACATTTGTCC

GGCTGCTCGCACTGCAGTGAAAATGGGGTCGAATGTCTGGTGGTATCTTTCTGGATACATAAAGGAAGCG

ATTAGGAAATCCTGTTCTTTAACGGGAAATCCGTGATGATTGTAAAATCCAAGGAAGTATATATTACTAG

GATTCGTAGTGATGTAGCTCTTAGCTTCATTCACCTCCATCCCAAACTTCCTCATCGCTAACCGGGAGAT

GTCTTCAAGGTTCAAATTCCCTGAGCAGATGATTAATCCGTCATCCCCCATGTACATATCCTGTTCAGGT

AGAAATCCGCTTGTTTGATATATCAAATATCTAGTCACGATACAGTTGACAATTGTATCTATTATATTCG

TAAAACATGACCCCGACGATACTCCTCCCTCCTTAAAGAATCTTTCTCCATTCGGAAGAGTGAAAGGCGT

ATTGATAAAATACGACACTAATTTTCGCCATCGGCGGCCAGTCTTTTGCGGATCTACGGGCCATACTTTT

CCCTCAGAATCGAAAACTTCGTTCATTTTGAAGCTCTCAAGCATGATTCCGAAAGCATCTCTAATCAGCC

AGGCGGAGATAGACTTGTCAAACTTCCTCCAGTCTATCATCAAGCACTTNGCTCCTGGTCCTGCCTGCTG

AAACATTTGATCTATGTAACCCATTCCTCCATTCGCCATCTCGATTCCGTACGCGACGGGAACACTAGTA

TTAGTGTGGCGTATCCAATCGAGGTGTGGGTACACGAAGCGCGCCTCTTCAAGTATCAGACTTAGGGGAT

ATCCCCAAGTACTTCTGATCTTGTTTGTATCCGGACTACAGATCTGTGCTCTAAAATAGACCATAGCATC

AGGAAGCTGCACCCGTGAACCTCTGCCCACGAGAGCCCATTCCATATGGATCTCAGACACAGCATCATGG

TCCTCTAGGACACTCTTCTTTGTTGTATAGCCCCTCTCTTTCCATGGCATACCAGGTGATTTCTTGGTAG

GTAAATTTGGTAAGTTTTGCACTCTTCCCAATGTCCATGGAGTAATGGATCCCTTAGCGGAGAAATCTTG

CCTCACGCTTTCTAGTACAGCAGAGTAGATAGAGTCCACGACTCTCGGGGAATACTCTCGATCGTAATCG

AAGACGTCATTCCTAATGGCGTCTAGAGAAACCCGAGCTCTATGGAACTTCTTTTCTACTCTTTTAGCGA

CTCCTTCTCCAAACCACTTATCTACGAATCTTCTTGCATAAGGATCGGGCTGGGAGGGTCTAAGGTCTAG

TGGGTATCCTTTTGTTTTGCTTCTTGCGTTGAGTGGCATTATTATATATATGTGCGAGTACTACTCTTAA

ACTCATCTAAAATTTTTTGTGTTGACATTGAAAAAATGTTAGACGCCATCTCACAGCCTCTCGCAAACTG

GCCAACAAGTCNAGAATATTAAATANANCAGTCCAGTTTTACCTTAGAAAGAAAATGTGGTTTTAGGACA

GGAGAATAAAGATAGTCGGAGAATTTTGGGAGCGACCCGTGCAGTTTTAAGACAAAAACGTAGGACTTTC

TTTCCTTTGAAGGATTACGACAGATCAACCGTTTTGCGAGGCCTATAAAACAGACAAATGTGAAAATTGT

CAATTTTCACATTCGTCTGTTTTATAGGCGATACATAGTATTTGTTGAGAGCTTGTGGATATTGTGGCTT

AAGCTCAGGTAACGTGATTTGCACATTCTACCTTCATAACGTAGCGACCATAGGGAACATGATCCTGTAT

CGTGGTGAAAACGCTTGAATTACCATCTCCTGTATATACTTTGTAAAGAACACCATGCTTTGCCTCACTT

TGTTGGACTCCTTCAACAATAATAGACTGTTTCATGCCAGTTGCAGGACCCCTATATTTTTTGTAGCATA

AATGGTCCTTTTGATCTGCATTGTCTCCAGATTTGGTATGTGCACATGAATAACAAAATTATTTTTTTAT

CTCTAAGTATAGTACTTTTTTGTTACTGCTCCAATTATGGCTCCAACACCTGAGAGTGAATTATACCCAT

GACCATATGTGTGCTTTGCCCATCCTCCATCGCCTATGAAAGATATATATGGAATCTCGTTTAAGAAGTT

ACCCTTTTCTTTGGTGATTTTTCGCTCTTCTTTCCCAGCAGATTTCGTTTTCAGAGATAGATACTCAATA

TCCTGTTCAGCTTTGCGGAAGGATTTNGATGACATAATGGGAACATCAACAATCCCCATCAACTCTTCCA

CCTGTGAATATCCTGTGCCTGTTGATAGAGCACCCCAAACCAAGCACTTTTTTACATCTAAAATATTGTT

AGTTTTATCACACCCCCTCCCTATTATTCCCCTACTATTATTATATGTGTGTCCCCAACTGAGTTGGCAA

CCACACATCACCCACAAAAATCAAAATTCTAAAATCACACATCAACAATTACAACTTAAATAAAAAAAAT

TTCCACGGCTAGGATTCGCGGTGATCAGACGCAGTACCCAATTTTTAAAGCAAACACAACAGCTTAAATT

CAGAGAAACGAACATTTTATTTGTCATATGCCTGACGGCTTACAACACGTTCGAACCCACCCACATAAAA

GTACGTAGCCCTCCCTCACACTTAATTCGTTTATTATTAAATTCAGTTCTAGTCGACATAGCGGTTGAAA

TGGAAATGTTCGTGCATGTTTATACGGAAGTTGAACTCGAAGTTAGAATGACGGAACAAAAGTTAATATG

GGAGATTTCCCTTCCGAACCCGGGGATAGGTATAAAATGGAGTAATCTTACCAATTTGGCGGCCGAAGTC

AGTTCTGAAGTGGTTACGTTGATATGCGCAAGTAATT

-------

Incomplete sequences and fragments of viral RdRPs in basal hexapods

Flavivirus NS5

>GATZ02003749.1 TSA: Sminthurus viridis C114795_a_4_0_l_726 transcribed RNA sequence

Length=726

Score = 231 bits (589), Expect = 2e-69, Method: Compositional matrix adjust.

Identities = 126/247 (51%), Positives = 161/247 (65%), Gaps = 13/247 (5%)

Frame = -3

Query 49 DTCPVEQTKYEERIFKIYKGLGMWFKGCGYKHRELTWEEVIMQANKVGAPGVIDHDVENV 108

DT PVE + Y ++ ++Y+GL W G+ ++ EE++ QANK GAP +DH +NV

Sbjct 724 DTEPVENSPYHTKMREVYEGLADWMSSRGFCNQPFNDEEILRQANKQGAPTRLDH-WKNV 548

Query 109 GAFLRKPTAEAEVEAIRMSFLSGKPRGAIFNTMGKREKKESQSGQKGSRMVAFLPIGTRI 168

G FL + E+ I +P AIF+TMGKREKK + G KGSRMVA+LPI TR+

Sbjct 547 GEFLADSEWKNELGKIEKGLTQNQPTRAIFSTMGKREKK--RGGDKGSRMVAYLPIPTRM 374

Query 169 LELKCLGMLINLTKPAINRFGVGGLGLHDLGMRLNQVWRGFGVSTDIAGFDTRVGLFIQS 228

+ELK G LI TKPAINRFGVGG+GLHD+GMR ++++ + S DIAGFDTRVGL + S

Sbjct 373 VELKYFGRLIECTKPAINRFGVGGIGLHDMGMRAKEIFKTWACSDDIAGFDTRVGLHVLS 194

Query 229 CE--SWL-----VQYLGGNATCEALYRCYAYPHILIPMTGEHVRSELLKGRGQRMRGSNP 281

E S++ QY + E LYR YAYPH+LIP RSEL+ GRGQRM G+ P

Sbjct 193 MEYHSFIKRLTPKQY---HPVIEGLYRIYAYPHLLIPTLSSFSRSELVAGRGQRMSGTAP 23

Query 282 TYAMNTI 288

TY MNTI

Sbjct 22 TYTMNTI 2

>GATZ02000981.1 TSA: Sminthurus viridis C103834_a_5_0_l_500 transcribed RNA sequence

Length=500

Score = 126 bits (317), Expect = 4e-31, Method: Compositional matrix adjust.

Identities = 65/140 (46%), Positives = 87/140 (62%), Gaps = 3/140 (2%)

Frame = +3

Query 398 VERYMPTRSVAEILAKSCLRVGGPDQDLSNLAWISAQGNMLLTYYAHLRTVRLVGLCYKA 457

VERY PTRS EI K + + G D LS AW SAQ N LL Y H+R R VGL ++

Sbjct 6 VERYQPTRSYGEIFGKLGIWIAGNDLQLSQDAWASAQANNLLVNYHHMRDCRRVGLMLRS 185

Query 458 LAPENLVMT-AKGGEFFPRPWMRPGDILEVMNAVLFGESTAYPVPDFALRSFRHVGYLKP 516

+ +N+++ + F PRPW++ GD L+++N LFG STAYPV +F +R +H+GYL

Sbjct 186 IVLDNIILLPMRKAGFLPRPWLQNGDTLDIINECLFGGSTAYPVQNFRVRQMQHLGYLMH 365

Query 517 QREQIYDPETFTMERVR-WR 535

RE IY+P F E +R WR

Sbjct 366 GRELIYEPR-FESEAMRTWR 422

TSA: Sminthurus viridis C114795_a_4_0_l_726 transcribed RNA sequence

Sequence ID: GATZ02003749.1Length: 726Number of Matches: 1

Related Information

Range 1: 2 to 724GenBankGraphicsNext MatchPrevious Match

Alignment statistics for match #1

Score Expect Method Identities Positives Gaps

Frame

231 bits(589) 6e-67 Compositional matrix adjust. 129/244(53%) 171/244(70%) 7/244(2%)

-3

Query 390 DTAPKENHKYEQHLVQIYEGLAHHFLKMGFSFRELDWTEVMHQANKQGAPGIIDTQFENV 449

DT P EN Y + ++YEGLA GF + + E++ QANKQGAP +D ++NV

Sbjct 724 DTEPVENSPYHTKMREVYEGLADWMSSRGFCNQPFNDEEILRQANKQGAPTRLD-HWKNV 548

Query 450 GQFLSQPNWVKKVIYTRQALEKGRPVGGVFNTIGKREKKLSPHEMKGSRMVAYLPIATRl 509

G+FL+ W ++ + L + +P +F+T+GKREKK KGSRMVAYLPI TR+

Sbjct 547 GEFLADSEWKNELGKIEKGLTQNQPTRAIFSTMGKREKKRGGD--KGSRMVAYLPIPTRM 374

Query 510 lelklfgkllelTKPAINHFGVGGLGLHDLGMRVEEIWQGHAVSDDIAGFDTRIGLFFLS 569

+ELK FG+L+E TKPAIN FGVGG+GLHD+GMR +EI++ A SDDIAGFDTR+GL LS

Sbjct 373 VELKYFGRLIECTKPAINRFGVGGIGLHDMGMRAKEIFKTWACSDDIAGFDTRVGLHVLS 194

Query 570 LENH-FIRLLGGNETHTL---MYRLYAYPHILVPLASEFTRSQLLKGRGQRMSGTNVTYS 625

+E H FI+ L + H + +YR+YAYPH+L+P S F+RS+L+ GRGQRMSGT TY+

Sbjct 193 MEYHSFIKRLTPKQYHPVIEGLYRIYAYPHLLIPTLSSFSRSELVAGRGQRMSGTAPTYT 14

Query 626 MNTI 629

MNTI

Sbjct 13 MNTI 2

DownloadGenBankGraphics NextPreviousDescriptions

TSA: Sminthurus viridis C103834_a_5_0_l_500 transcribed RNA sequence

Sequence ID: GATZ02000981.1Length: 500Number of Matches: 1

Related Information

Range 1: 3 to 428GenBankGraphicsNext MatchPrevious Match

Alignment statistics for match #1

Score Expect Method Identities Positives Gaps

Frame

124 bits(311) 8e-30 Compositional matrix adjust. 65/143(45%) 82/143(57%) 3/143(2%)

+3

Query 737 TTHRYMPTRDLTQIIAKSTIKIGGKDASLDDMAWLSAQGNNLLVNYAHLRTARAVGFGYK 796

T RY PTR +I K I I G D L AW SAQ NNLLVNY H+R R VG +

Sbjct 3 TVERYQPTRSYGEIFGKLGIWIAGNDLQLSQDAWASAQANNLLVNYHHMRDCRRVGLMLR 182

Query 797 AIVNPNALLC--DTGGFLRPTPWMQPGDILNVTNKILFGESTHYPVEGFRVHSWKHVGFL 854

+IV N +L GFL P PW+Q GD L++ N+ LFG ST YPV+ FRV +H+G+L

Sbjct 183 SIVLDNIILLPMRKAGFL-PRPWLQNGDTLDIINECLFGGSTAYPVQNFRVRQMQHLGYL 359

Query 855 KPKREMVYDQDTFSAGRAYWRSK 877

RE++Y+ S WR+

Sbjct 360 MHGRELIYEPRFESEAMRTWRAN 428

DownloadGenBankGraphics NextPreviousDescriptions

TSA: Sminthurus viridis C109709_a_3_0_l_603 transcribed RNA sequence

Sequence ID: GATZ02002474.1Length: 603Number of Matches: 1

Related Information

Range 1: 2 to 601GenBankGraphicsNext MatchPrevious Match

Alignment statistics for match #1

Score Expect Method Identities Positives Gaps

Frame

112 bits(280) 3e-25 Compositional matrix adjust. 73/201(36%) 104/201(51%) 7/201(3%)

+2

Query 125 THITSVTLGPGPGHEGHEAFTKIHFPGREKIRVLYGDARQFPI---ASHDTLLFDGGESH 181

T++ VT G GHE FT F G+EK+ V+ D R+F D +LFDGGE

Sbjct 2 TNVWGVTFGKLQATPGHEDFTDRPFKGKEKVNVINMDVRKFVAEVDVEADWVLFDGGEQR 181

Query 182 SDADIEEARFYSLFYNVVMRQINSQTKHFVLKVLTPTSPRIQKLLEEIQRMTGRGAFYRS 241

S+ D E ++F +L + I +TK F+ K+LTP I + LE IQ+MTG G F +

Sbjct 182 SNYDEEASKFRALVLPC-LEAIGPKTKGFIFKILTPFDDAIIRKLEHIQQMTGMGNFILN 358

Query 242 CHSRLSTMELYFVSTG-IAPVKGRAYSLLQSVMIQGRENKV--LKPRNYDLGFTFSREKI 298

H+R S +ELYFVST + ++ A L++ + + L P R I

Sbjct 359 THTRQSNLELYFVSTKPVMNLRKSARQLIEYKLSRAEIESAHKLNPIRMANADYLGRTPI 538

Query 299 EPKTIPLLKPLDLTDSINELG 319

+PLL P+D+ SI+ELG

Sbjct 539 NDPVVPLLMPVDMNRSISELG 601

Flavivirus NS3

TSA: Sminthurus viridis C106664_a_3_0_l_547 transcribed RNA sequence

Sequence ID: GATZ02001699.1Length: 547Number of Matches: 1

Related Information

Range 1: 29 to 535GenBankGraphicsNext MatchPrevious Match

Alignment statistics for match #1

Score Expect Method Identities Positives Gaps

Frame

81.6 bits(200) 9e-15 Compositional matrix adjust. 55/173(32%) 86/173(49%) 12/173(6%)

+2

Query 207 GFAALGCVLIFVFFQTMLDHPSVYVASAASTPVRDGIYMVRTYWLGIPLSKGVGVATSGV 266

GF + +Q M+D + +TP+++G Y++ G SK +GV+ +GV

Sbjct 29 GFVMFLSAVFIYTWQDMVD--KYQLMGTENTPIQEGSYLITRELFGHVFSKCMGVSYAGV 202

Query 267 LYIPYHVVTSMPVWIDNRCLLPSYVDVKRDLVTFGGTPSLVAPLDQEELVVS----LERD 322

L+ PYH + I R + P +V V DL+T+GG PS + +VV+ +ER

Sbjct 203 LHAPYHGAHRNDIIIGGRLVKPYFVSVDCDLITWGGMPSFATLTADDRVVVNHEDEIERR 382

Query 323 DGRFCYRTVANVDEFGFTFVGKSSPGESGSPVYVLRGEKP----TLVGFCGRW 371

Y+ +D F++ GKS PG+SGS V+ + E +LVG GRW

Sbjct 383 SMLVDYKYDDEIDS--FSWAGKSKPGQSGSGVWKVTEEGDIQIISLVGLVGRW 535

Gap 40 bp

TSA: Sminthurus viridis C120865_a_7_0_l_931 transcribed RNA sequence

Sequence ID: GATZ02005284.1Length: 931Number of Matches: 1

Related Information

Range 1: 63 to 929GenBankGraphicsNext MatchPrevious Match

Alignment statistics for match #1

Score Expect Method Identities Positives Gaps

Frame

176 bits(447) 1e-46 Compositional matrix adjust. 116/300(39%) 176/300(58%) 12/300(4%)

-3

Query 405 TDEVIRVCKHPGYGKTRRIIPELIATHSTQVKNPKVIITGPTVVVCEELHKSLLANCALD 464

+D + R+ HPG GKT ++IP ++ + ++ + KV+I GPT VVC+EL+ SL+

Sbjct 929 SDPLKRIIMHPGAGKTFKVIPSVVHKYLSEGRKGKVMIVGPTRVVCQELYNSLVRKFPC- 753

Query 465 VGLCVRDRKRYRVKRAPVQVMAHATLWRMIETDAFEVRNPTMLIIDEAHADMESTKVLLK 524

VGL +++ RYR A +Q+ H T RMI+ + EV N +LI+DEAH + +T++ +K

Sbjct 752 VGLSMKNNTRYRKPTARIQITTHHTFLRMIQNASIEVANLGLLILDEAHVEGVATRLCVK 573

Query 525 YGEQLAKSGGKVVLLSATFSNDLYDDGSNYDIKDVDRVDDYVSNGITVLLEDLTHHVNLD 584

Y E L+KSGG+ VLLSATF +D + +GSN+ I D ++++G + + + VN

Sbjct 572 YAENLSKSGGRAVLLSATF-DDTHSEGSNFTI-----TDKHINSGDEISI--VKSKVNDG 417

Query 585 QKVLVFCPGVMGSEGVNALRDKVKALHPEKRVLTLHRDVMAHRWNSLKTSNYDVILATNI 644

++VL F PG G G + D++ + + + + R + L + V++ATNI

Sbjct 416 KRVLWFVPGYHGKNGAKEMADRL--MRDGIQAIAVGRPSYTDYASRLNDTTVRVVVATNI 243

Query 645 AEMGMNIDVDVVLDLCKRFTYLG-DTYVVGSTLSINLssrvqrrgrvgrIKPGYYYYYGK 703

AE GMNID DVV++ F Y D V G T +I SS +QRRGR GR K G +YY K

Sbjct 242 AECGMNIDCDVVVNTATEFDYFSFDNIVTGETRTIGHSSWIQRRGRCGRTKVGEHYYTAK 63

Partitivirus

>GASN02036638.1 TSA: Thermobia domestica C218246_a_5_0_l_601 transcribed RNA

sequence

Length=601

Zygentoma

Sort alignments for this subject sequence by:

E value Score Percent identity

Query start position Subject start position

Score = 63.3 bits (138), Expect(2) = 1e-16, Method: Compositional matrix adjust.

Identities = 33/67 (49%), Positives = 40/67 (60%), Gaps = 0/67 (0%)

Frame = -3

Query 294 FINTPIQLSSGERFMKYGGVPSGSCFTNVVDGIVNALATRYLVYHMTGSLPLDDLYLGDD 353

FI T +QLS+GERF K VPSGS +T +VD IV + TR V +T L LYLGDD

Sbjct 593 FIETVVQLSTGERFKKTRVVPSGSYYTSIVDSIVSMIVTRTCVLLITSRLTDAYLYLGDD 414

Query 354 SIVITDK 360

+K

Sbjct 413 FCCCCNK 393

Score = 51.7 bits (111), Expect(2) = 1e-16, Method: Compositional matrix adjust.

Identities = 39/140 (28%), Positives = 63/140 (45%), Gaps = 10/140 (7%)

Frame = -1

Query 355 IVITDKPLNMDVFSEKADEWFSLIYNVDKSYQTANPQNVHFLGYYNMTGVPFKPVDTTIA 414

+V+T+KP + S+ A+ FS+I S T P V FLG++ G K + +

Sbjct 406 VVVTNKPYYIRKNSQIAESLFSMIMPDKMSVVTRRPNCVNFLGFHYRQGASWKNQASGVV 227

Query 415 SSVYPERMPRNK-FETAVRLVGQAYSCFEPTDAKNFFRAAKILVNEMEGANLDMIKEFTA 473

S + PE R + A+ VGQ ++ +P A F + M NL+ + TA

Sbjct 226 SWIRPECESRTESTRMAICTVGQLFAFLDPGKAGVF-------MAYMARKNLEKVDVMTA 68

Query 474 DHPEF--FKYLQTIGVSTKE 491

+ YL+ IG+ +E

Sbjct 67 LRSNYDTLTYLREIGIDPRE 8

FIETVVQLSTGERFKKTRVVPSGSYYTSIVDSIVSMIVTRTCVLLITSRLTDAYLYLGDD

FVVVTNKPYYIRKNSQIAESLFSMIMPDKMSVVTRRPNCVNFLGFHYRQGASWKNQASGV

VSWIRPECESRTESTRMAICTVGQLFAFLDPGKAGVFMAYMARKNLEKVDVMTALRSNYD

TLTYLREIGIDPRE

nyamivirus

Catajapyx aquilonaris contig_2370, whole genome shotgun sequence

Sequence ID: JYFJ02002370.1Length: 58159Number of Matches: 1

Range 1: 10635 to 10901GenBankGraphicsNext MatchPrevious Match

Alignment statistics for match #1

Score Expect Method Identities Positives Gaps

Frame

52.1 bits(112) 5e-06 Compositional matrix adjust. 29/89(33%) 48/89(53%) 6/89(6%)

-3

Query 141 MTGAGDNQVVSVKLVKTPAL------QSLvkkvkvkLAEGFAEVGLKVKLAETWHSSILL 194

+ G GDN+V+ + + A Q+ V LA+ + + +K E+W S+ILL

Sbjct 10901 IPGQGDNRVILLNIPDEIAPGPSEGNQAYVDGFLRSLAQWSKALKVPIKPEESWKSTILL 10722

Query 195 AYQRRYFLRGIPVPNAIKQGTRAFSGMSD 223

Y RRY+L+G+ VPN +K+ +R + D

Sbjct 10721 EYARRYYLKGVEVPNDLKKASRLPAQAND 10635

KYVSKFQSMTLGESWLSKRLLKMTRTMISHVSLILAAAFDVGALISIPGQGDNRVILLNM

PDEIAPGPSEGNQAYVDGFLRSLAQWSKALKVPIKPEESWKSTILLEYASSYYLKGVEVP

NDLKKASSLPAQANDPFSWLLGDLYGIFTSGCTAAHEDLEEAKATMAFWL

chuvirus

Catajapyx aquilonaris contig_6454, whole genome shotgun sequence

Sequence ID: JYFJ02006454.1Length: 19481Number of Matches: 1

Range 1: 1642 to 1947GenBankGraphicsNext MatchPrevious Match

Alignment statistics for match #1

Score Expect Method Identities Positives Gaps

Frame

76.3 bits(168) 2e-12 Composition-based stats. 36/104(35%) 55/104(52%) 4/104(3%)

-3

Query 550 NVDSSSWNNMFRHEALAPVIAATLDKYYNCSIFSKTQLAFQNTFIYLPDVEETYSWNGQK 609

+D +WNN FR E PV D+ ++ F F + ++L D YSW+GQ+

Sbjct 1947 SIDIDAWNNCFRNELCEPVGRLIFDRIFDQ*QFQHIMHVFNRSLVFLSDFSSMYSWDGQQ 1768

Query 610 GGIEGLNQ--YTWVTAYIHQMKVCMRNFAFPYHILCKGDDLRIC 651

GGIEGLNQ +TW+ + + VC + + ++ GDDL+ C

Sbjct 1767 GGIEGLNQKFWTWIYESVARY-VCDQAGVTAF-LMANGDDLKTC 1642

partitivirus

Machilis hrabei contig_337473, whole genome shotgun sequence

Sequence ID: QVQU01337473.1Length: 3397Number of Matches: 1

Related Information

Range 1: 720 to 1466GenBankGraphicsNext MatchPrevious Match

Alignment statistics for match #1

Score Expect Method Identities Positives Gaps

Frame

62.4 bits(150) 2e-08 Compositional matrix adjust. 70/276(25%) 111/276(40%) 61/276(22%)

-3

Query 162 DVAKQIHKE--LPKQNQLFAIP----YHRAQISGYVGKNGKLRPKATKHKDRLVWCVDAA 215

D +IH E L + +P Y RAQI +K R W +

Sbjct 1466 DAVSEIHMEWALVGRGSRVQLPDAMVYFRAQIC-----------SPDTNKIRSTWGYPLS 1320

Query 216 TVLIESLYARPFID---------KVLTHIQNYAGGKDDNYISNLLI-AWNVDNWISIDYS 265

+L E+ + P +D V I+ GG YI + A + ID+

Sbjct 1319 LILEEARFVYPHLDWIRHTNTSVPVAYGIEMANGGM--GYIDQMFQQAGPGAKCLMIDWR 1146

Query 266 KYDSTVQAWLIKDVFDIIRSFFSKRDV-----KVL---------QW--IENQFIHTRILD 309

K+D ++ AWLI+D F I+ F +V KV +W + + FI+T

Sbjct 1145 KFDKSISAWLIRDAFGIMLESFKMNEVFDSEGKVWPVDPQKTGRRWRKLVSYFINTPFTL 966

Query 310 FDGNISTKHRGIPSGSYFTQIVGSLVNALVI--LTYEFARFRGNEAKVWEDMRYGDWLRF 367

+G K G+ SGS FT I+ ++VN +V L Y+ + F +

Sbjct 965 PNGERFFKEGGVSSGSCFTNIIDTIVNCIVTRYLIYQTSGFLPEQD-------------- 828

Query 368 MTMGDDNIVFTRNKISLDDLASYVEHNFGLVINVEK 403

M MGDD ++ ++L+D++ FG+ +N K

Sbjct 827 MYMGDDGLIICSGNLNLEDISRLAMRKFGMEVNEAK 720

Machilis hrabei contig_73480, whole genome shotgun sequence

Sequence ID: QVQU01073480.1Length: 12352Number of Matches: 2

Related Information

Range 1: 287 to 1099GenBankGraphicsNext MatchPrevious Match

Alignment statistics for match #1

Score Expect Method Identities Positives Gaps

Frame

437 bits(1125) 4e-136 Compositional matrix adjust. 213/271(79%) 233/271(85%) 1/271(0%)

+2

Query 50 VSLDAIRNDVFDYDREYSPRVVDSIYSAVLESVRQDFSAKGSITPWTLGRVQNLPNLPTK 109

VS +AIRNDVFDYDR+YSPRVVD++YSAVLE+VR+DFSA GSITPWTLGRV+N PNLPTK

Sbjct 287 VSHNAIRNDVFDYDRQYSPRVVDAVYSAVLETVRRDFSAHGSITPWTLGRVKNSPNLPTK 466

Query 110 KSPGMPWKERGYTTKKSVLEDHDAVSEIHMEWALVGRGSRVQLPDAMVYFRAQICSPDTN 169

KSPGMPWKE+GY TK+SVLEDH AVSEIH EWALVG+G RVQL D+MVYFRAQICSPDTN

Sbjct 467 KSPGMPWKEKGYATKQSVLEDHTAVSEIHREWALVGKGLRVQLLDSMVYFRAQICSPDTN 646

Query 170 KIRSTWGYPLSLILEEARFVYPHLDWIRHTNTSVPVAYGIEMANGGMGYIDQMFQQAGPG 229

K RSTWGYPL LILE+ARFVYP+LDWI HT TSVPVAYGI+MANGGMGYIDQMF+QAGP

Sbjct 647 KXRSTWGYPLGLILEKARFVYPYLDWIHHTQTSVPVAYGIQMANGGMGYIDQMFKQAGPE 826

Query 230 XKCLM-IDWRKFDKSISAWLIRDAFGIMLESFKMNEVFDSEGKVWPVDPQKTGRRWRKLV 288

S+ LIRDAF IMLESFK+NEV+DSEGK WPVDPQKTGRRW+KLV

Sbjct 827 QNA*W*TGENLISLSLLG*LIRDAFNIMLESFKLNEVYDSEGKTWPVDPQKTGRRWKKLV 1006

Query 289 SYFINTPFTLPNGERFFKEGGVSSGSCFTNI 319

YFINTPF LPNGERF KEGGV SGSCF +

Sbjct 1007 QYFINTPFVLPNGERFLKEGGVPSGSCFQTL 1099

Range 2: 808 to 1458GenBankGraphicsNext MatchPrevious MatchFirst Match

Alignment statistics for match #2

Score Expect Method Identities Positives Gaps

Frame

235 bits(600) 5e-66 Compositional matrix adjust. 129/243(53%) 148/243(60%) 53/243(21%)

+1

Query 224 QQAGPGXKCLMIDWRKFDKSISAWLIRDAFGIMLESFKMNEVFDSEGKVWPVDPQKTGRR 283

Q +G KCLM+DWRKF D W +D ++

Sbjct 808 QTSGARAKCLMVDWRKF--------------------------DKSIPAWLID*RRL*HN 909

Query 284 WRKL--------------------------VSYFINTPFTLPNGERFFKEGGVSSG-SCF 316

RKL VS + + R +GG S+

Sbjct 910 ARKL*AKRSV*F*RQDMAR*STEDWSQMEEVSTIFHKHTLCTS*WRKILKGGRSTIWIML 1089

Query 317 TNIIDTIVNCIVTRYLIYQTSGFLPEQDMYMGDDGLIICSGNLNLEDISRLAMRKFGMEV 376

NIIDTIVNCIVTRY++ QTSGF+PEQDMYMGDDGLII SG +NL DIS+LAM KFGMEV

Sbjct 1090 PNIIDTIVNCIVTRYVLLQTSGFIPEQDMYMGDDGLIIISGKINLSDISKLAMTKFGMEV 1269

Query 377 NEAKSYITTNPSNIYFLGFYNHHGFPVKEQDFLIASFMYPERYHQTFDPIFTAVRAAGQM 436

NEAKSYIT+NP+NIYFLGFYNHHGFPV+EQDFLIASFMYPER+H+ FDPIFTAVRAAGQM

Sbjct 1270 NEAKSYITSNPTNIYFLGFYNHHGFPVREQDFLIASFMYPERFHKIFDPIFTAVRAAGQM 1449

Query 437 WST 439

WST

Sbjct 1450 WST 1458

permutotetra

TSA: Megajapyx sp. UVienna-2012 DMPC16734223 mRNA sequence

Sequence ID: JT051866.1Length: 427Number of Matches: 1

Range 1: 4 to 390GenBankGraphicsNext MatchPrevious Match

Alignment statistics for match #1

Score Expect Method Identities Positives Gaps

Frame

158 bits(399) 7e-42 Compositional matrix adjust. 75/129(58%) 95/129(73%) 4/129(3%)

+1

Query 528 MYRGGPTRTEPVPYLPDDEWTNLIMVPRDDPENFRRGRPKDSQLTVSRRWYDRARGYMVT 587

+Y GP + + VPYLP +EW +L++ PRDDPENFRRGRPK SQ+T++RRWYDR RGYM+T

Sbjct 4 IYEQGPNKIQLVPYLPYEEWVDLLLSPRDDPENFRRGRPKTSQVTIARRWYDRIRGYMIT 183

Query 588 GAFSNPLIRDWIHGFVNGLDPVSIVMNVQEGDGR----GAPPDSAFLEDFQYPDSAGFPT 643

GAFSN IR W+ G VN +D V IVM+VQ G G+ G P E+F+YPDS+GFP+

Sbjct 184 GAFSNESIRLWLQGVVNDMDSVPIVMSVQAGGGKGEVPGIPGFFEEHEEFEYPDSSGFPS 363

Query 644 EEWCENLYF 652

WC+NLY

Sbjct 364 VPWCQNLYL 390

The only case of HVT in basal hexapods

>Holacanthella duospinosa (GFPE01073448, 340 bp) Bromoviridae; Alfamovirus

ASFHFKEIDFSKFDKSQNELHHLIQERLLKYLGIPNEFLTLWFNAHRKSRISDSKNGVFF

NVDFQRRTGDALTYLGNTIVTLACLCHVYNLMDPNVKFVVASGDDSLIGTVEE

P2 protein [Alfalfa mosaic virus]

Sequence ID: ATS17298.1Length: 790Number of Matches: 1

Range 1: 522 to 634GenPeptGraphicsNext MatchPrevious Match

Alignment statistics for match #1

Score Expect Method Identities Positives Gaps

236 bits(603) 2e-71 Compositional matrix adjust. 112/113(99%) 112/113(99%) 0/113(0%)

Query 1 ASFHFKEIDFSKFDKSQNELHHLIQERLLKYLGIPNEFLTLWFNAHRKSRISDSKNGVFF 60

AS HFKEIDFSKFDKSQNELHHLIQERLLKYLGIPNEFLTLWFNAHRKSRISDSKNGVFF

Sbjct 522 ASSHFKEIDFSKFDKSQNELHHLIQERLLKYLGIPNEFLTLWFNAHRKSRISDSKNGVFF 581

Query 61 NVDFQRRTGDALTYLGNTIVTLACLCHVYNLMDPNVKFVVASGDDSLIGTVEE 113

NVDFQRRTGDALTYLGNTIVTLACLCHVYNLMDPNVKFVVASGDDSLIGTVEE

Sbjct 582 NVDFQRRTGDALTYLGNTIVTLACLCHVYNLMDPNVKFVVASGDDSLIGTVEE 634

Sobemo-like viruses

>Pogonognathellus sp. AD-2013 capsid of sobemovirus (GATD02018541, 390 bp) Collembola

RSNAALRKIVQDVTNNMSQMNLKTKSKKVKRKRKSGKKVEGSGAQQGSIRLRQSEIWTGV

TSTASKTQTTKTLPCVPGGSGIKYLDQMARLFDRFIFHSATVSWKPYCSASTNGGMLFGI

DWEVGSDIF


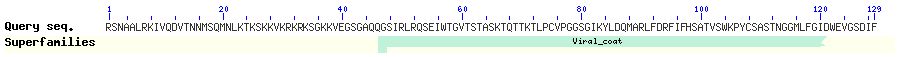


>GFPE01061060.1 TSA: Holacanthella duospinosa c72747_g1_i1 transcribed RNA sequence

Length=273

Score = 55.1 bits (119), Expect = 5e-06, Method: Composition-based stats.

Identities = 29/77 (38%), Positives = 47/77 (61%), Gaps = 3/77 (4%)

Frame = -1

Query 175 AYYRFEQLYLKCVFITSGGHLLRQLRPGALKSGCVNTIADNSLMQYLLHLRVVFQLGYSP 234

A +R+ LYL +F TS GH RQ+ PG +KSG + TI+DNS+ +L + + ++ P

Sbjct 270 AAWRYNCLYLNPLFQTSSGHQFRQMLPGIMKSGGLLTISDNSIGVAVLEV-LTSEMTSEP 94

Query 235 --GIIMSMGDDTLQQEP 249

+ +MGDD+ + +P

Sbjct 93 IDKSVKAMGDDSTRSKP 43

LAAWRYNCLYLNPLFQTSSGHQFRQMLPGIMKSGGLLTISDNSIGVAVLEVLTSEMTSEP

IDKSVKAMGDDSTRSKPMSPAKLVAQSKLGM


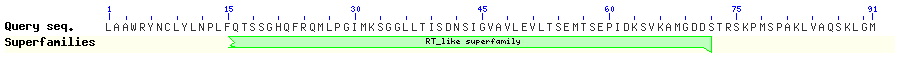


>Holacanthella duospinosa sobemo-like capsid (GFPE01060684, 469 bp)

VLRDMFQQLIDELGVRDPTPKYREFRDKFFSIPEAGLKEWTPDLLLVLTMMIPSVVFWLM

VMARFVGLLVALARWTFMIDGMKYIPEAIIEGSTFLAGEPPKFQARIRATELMGRLVGNA

VRMNNYLVVPTHVLQAASGASVTVEASKSKLILPKT

Endogenous qinvirus

>QVQU01284830.1 Machilis hrabei contig_284830, whole genome shotgun sequence

Length=997

Sort alignments for this subject sequence by:

E value Score Percent identity

Query start position Subject start position

Score = 176 bits (400), Expect(2) = 8e-50, Method: Compositional matrix adjust.

Identities = 105/300 (35%), Positives = 158/300 (53%), Gaps = 20/300 (7%)

Frame = +1

Query 339 MKHGLAILQNNFHSHAEKLEMGLGNRNDMLWKEQKDIADLKIEWYSFLSDLDIPERDKVN 398

MK +A+LQN+FH H+E LEMG R L + ++I + W+ + + +R +++

Sbjct 1 MK*SVALLQNDFHEHSEVLEMGTKTRSTALKRIIREIHPMXTFWHEVVRANKLTDRARLD 180

Query 399 LAYLYHILPSPDAPPKEVYERVVTQLNDSNKEDVEEFATFLNYCKATDVSMFVTKSHGKV 458

LA +Y+ L +PD + + R + + ++ C+ FV K

Sbjct 181 LANMYYGLTAPDDDEQNLRSRATEYMATAKRKT----------CR------FVRKFRRYP 312

Query 459 KLHCKDGYDPSVKKWYKSC--AQGKLMLPPKHEWGLCWIKNVVPVNLLIHTWYLNSADVT 516

+ ++GY+P W SC + +PP+ EWG CWI+ VP N W +++DVT

Sbjct 313 RYSIEEGYNPEGSAWADSCLSPEKLFKMPPEEEWGKCWIEGEVPYNDTTSQWPWDASDVT 492

Query 517 RVVPDISKYDSLVEKSSQVPMEV-NELLYTLMHAPDIEPGWSPELVLECVRNGSLNLPKI 575

VV D Y S V K ++ V NELLY L+HAP + W P L+ C++ S ++

Sbjct 493 HVVADTKIY-SXVGKMNELKRYVHNELLYALVHAPYLSFKWDPSLLRFCMQCDSDIWDRL 669

Query 576 ADMSVKSENTKYGPKMRETWSADAITRELLTDYDHSLIGLSHMVDGVTSRKSNVHVDGIF 635

A ++ KSENTK G K+RETWS D +TREL + YD S I L+ + GV RKS VD +F

Sbjct 670 ATIAAKSENTKPGMKVRETWSGDDVTRELTSAYDSSAIPLASLYSGVV*RKSQGKVDEVF 849

Score = 47.8 bits (102), Expect(2) = 8e-50, Method: Composition-based stats.

Identities = 20/31 (65%), Positives = 23/31 (74%), Gaps = 1/31 (3%)

Frame = +3

Query 649 IIISNDTSGWSPSAPREAWAQHHDYKVSLTS 679

+II ND SGWSP R AWA+HHDY V+ TS

Sbjct 900 LIIFNDVSGWSPLGDRIAWAEHHDY-VARTS 989

>QVQU01284831.1 Machilis hrabei contig_284831, whole genome shotgun sequence

Length=2829

Score = 50.8 bits (109), Expect = 3e-04, Method: Compositional matrix adjust.

Identities = 55/213 (26%), Positives = 85/213 (40%), Gaps = 53/213 (25%)

Frame = +3

Query 794 LSESKTIYSSIKFIYLNRFFCEGSEVLVDMKTFSKVDRDYNRRLSTFFQASDTVMGGYRS 853

L E KT+YSSIK ++LN CEGSE++ MK F++ D++ RR Y S

Sbjct 6 LDEVKTMYSSIKSLFLN*MHCEGSELVFPMKVFARADKEKSRR--------------YAS 143

Query 854 SAMKGSDPMLCYYWYFFRSFDLMIQSNAIIKEIIPNLGYLVTAAFAPRSLNGLGMPHIVA 913

+G DP++ Y + DL+ + K G+

Sbjct 144 E--RGDDPLVXYGTAIEKVCDLVWRPSGEXK------GF--------------------- 236

Query 914 FMTKEKVDALAMFFGVHVRLKNLISNVHIRNSLIYSFSAYVNQKLEPPTATVVFKDPMAV 973

F+ ++ V L L+ L S R I S +NQ + K P ++

Sbjct 237 FLGQQDVCIL---------LETLASPGD-RELCINILSHTLNQTPAVVDIDALLKSPTSI 386

Query 974 YAADRISPDDNVYRTLKARMRLKCKSPAISEAF 1006

A + PD V R +++ + KSP +AF

Sbjct 387 RAINIPDPDSAVMRRVRSALLKFAKSPVFLQAF 485

>QVQU01284830.1 Machilis hrabei contig_284830, whole genome shotgun sequence

Length=997

Sort alignments for this subject sequence by:

E value Score Percent identity

Query start position Subject start position

Score = 255 bits (584), Expect(2) = 2e-78, Method: Compositional matrix adjust.

Identities = 138/313 (44%), Positives = 182/313 (58%), Gaps = 22/313 (7%)

Frame = +1

Query 333 MKQSVALMQNELHKHSEHLETNTEQKSANLREAIAGILPLDSYWHDHLSNAGISDRAKMD 392

MK SVAL+QN+ H+HSE LE T+ +S L+ I I P+ ++WH+ + ++DRA++D

Sbjct 1 MK*SVALLQNDFHEHSEVLEMGTKTRSTALKRIIREIHPMXTFWHEVVRANKLTDRARLD 180

Query 393 LANLYYNLPSPDADLESLWKKGAEIMCNASTADPEIWKLFMNYSKALDFCKLTAMIKEVP 452

LAN+YY L +PD D + L + E M A C+ + P

Sbjct 181 LANMYYGLTAPDDDEQNLRSRATEYMATAKRKT----------------CRFVRKFRRYP 312

Query 453 KHRKKEGYEFEESQWFKSCLKGK--MRLPPDEEMGNVWIYHHFEFQNTLSEWYWEAGDVT 510

++ +EGY E S W SCL + ++PP+EE G WI + +T S+W W+A DVT

Sbjct 313 RYSIEEGYNPEGSAWADSCLSPEKLFKMPPEEEWGKCWIEGEVPYNDTTSQWPWDASDVT 492

Query 511 HVHADLANYTDQLKATSLTREDCNELLYAMDYAPLLSKKYSPSEVLDRVCT--GKKCWDS 568

HV AD Y+ K L R NELLYA+ +AP LS K+ PS L R C WD

Sbjct 493 HVVADTKIYSXVGKMNELKRYVHNELLYALVHAPYLSFKWDPS--LLRFCMQCDSDIWDR 666

Query 569 IALMAAKSENTKPGAKVRETWSGDDVTRELTSCYDRQAIPLGSMYRGMVSRKPPVKVDAM 628

+A +AAKSENTKPG KVRETWSGDDVTRELTS YD AIPL S+Y G+V RK KVD +

Sbjct 667 LATIAAKSENTKPGMKVRETWSGDDVTRELTSAYDSSAIPLASLYSGVV*RKSQGKVDEV 846

Query 629 FDRIADLTTKPRE 641

F I TT+ ++

Sbjct 847 FQLITSRTTQDKK 885

Score = 64.2 bits (140), Expect(2) = 2e-78, Method: Composition-based stats.

Identities = 24/31 (77%), Positives = 26/31 (84%), Gaps = 0/31 (0%)

Frame = +3

Query 645 IIISNDVSGWSPQGDRKAWAEHHDYVVHTSD 675

+II NDVSGWSP GDR AWAEHHDYV TS+

Sbjct 900 LIIFNDVSGWSPLGDRIAWAEHHDYVARTSN 992

>QVQU01284831.1 Machilis hrabei contig_284831, whole genome shotgun sequence

Length=2829

Score = 98.3 bits (219), Expect = 2e-18, Method: Compositional matrix adjust.

Identities = 67/221 (30%), Positives = 101/221 (46%), Gaps = 59/221 (27%)

Frame = +3

Query 789 EIDQVKTLYSSIKVIFLNRLYCEGAEVLTPMKVYARVDRELTRRFSTVYEQVDTILGGFR 848

E+D+VKT+YSSIK +FLN ++CEG+E++ PMKV+AR D+E +RR+

Sbjct 3 ELDEVKTMYSSIKSLFLN*MHCEGSELVFPMKVFARADKEKSRRY--------------- 137

Query 849 SASERGADPMVCYIMAIYRSLDLIIQSS---RGCIHGNLDVMEIVNAAFAPRGLGGWGLP 905

ASERG DP+V Y AI + DL+ + S +G G DV ++ +P

Sbjct 138 -ASERGDDPLVXYGTAIEKVCDLVWRPSGEXKGFFLGQQDVCILLETLASP--------- 287

Query 906 HMTGWLTQESQDKLTAYLGVIFSLNEYMMESGTVTRLSSYIYKTLNQTLAEATVEGILDS 965

G + TLNQT A ++ +L S

Sbjct 288 -------------------------------GDRELCINILSHTLNQTPAVVDIDALLKS 374

Query 966 PRDVRVGSLVGLSGAVLEKVKREMAQRAKSPIFRAALNSNS 1006

P +R + AV+ +V+ + + AKSP+F A + S

Sbjct 375 PTSIRAINIPDPDSAVMRRVRSALLKFAKSPVFLQAFATTS 497

Endogenous tombus-like viruses

Machilis hrabei contig_261130, whole genome shotgun sequence

Sequence ID: QVQU01261130.1Length: 2308Number of Matches: 5

Related Information

Range 1: 1722 to 2057GenBankGraphicsNext MatchPrevious Match

Alignment statistics for match #1

Score Expect Method Identities Positives Gaps

Frame

130 bits(327) 3e-36 Compositional matrix adjust. 64/113(57%) 82/113(72%) 2/113(1%)

+3

Query 658 RGT-RMSGDVDTSLGNSILNYAIIRQVLEMLGIQGDVIVNGDDSIIFTNVPIPIQQCERL 716

RG+ RMS DV TS GNS++NYAII++ L + GI GDVIVNGDDSIIFTN P+P +

Sbjct 1722 RGSPRMSADVHTSFGNSLINYAIIKKALRIHGIDGDVIVNGDDSIIFTNEPLP-SSLQDT 1898

Query 717 MKMFNQESKLKPSTQNIHTVEYCRTKLIVTAEGETTLLFDPQRSVDMFGMTYQ 769

++FN E+KLKPS +NIH VE+CR K++V G+ L+ DP R +FGMTY

Sbjct 1899 FRVFNMEAKLKPSLRNIHQVEFCRCKVVVNTLGQWMLMMDPGRHERVFGMTYH 2057

Range 2: 1541 to 1726GenBankGraphicsNext MatchPrevious MatchFirst Match

Alignment statistics for match #2

Score Expect Method Identities Positives Gaps

Frame

47.4 bits(111) 3e-36 Compositional matrix adjust. 25/63(40%) 43/63(68%) 2/63(3%)

+2

Query 598 YRYFTELDHTSWDAHVTVEMLRVSHKFYQSCY-NHDRRLRTLSRKTIRNRCYLRDGGRHT 656

++Y++EL H++++ +VT EML ++HKFY++CY ++ L+ LS +TI NR R G

Sbjct 1541 WKYYSELXHSNFEKNVTEEMLLLTHKFYRACYPDNVEELKKLSARTI-NRFITRTG*EME 1717

Query 657 IRG 659

+ G

Sbjct 1718 MEG 1726

Range 3: 1100 to 1405GenBankGraphicsNext MatchPrevious MatchFirst Match

Alignment statistics for match #3

Score Expect Method Identities Positives Gaps

Frame

90.1 bits(222) 7e-36 Compositional matrix adjust. 51/103(50%) 63/103(61%) 6/103(5%)

+2

Query 612 HVTVEMLR-----VSHKFYQSCYNHDRRLRTLSRKTIRNRCYLRDGGRHTIRGTRMSGDV 666

H T+ ML+ + CY + LS +TI NRC R G + RG+RMS DV

Sbjct 1100 HSTLTMLKS*RSCLRGLLTSVCYPDNVEELKLSVRTI-NRCITRTGEKWKWRGSRMSADV 1276

Query 667 DTSLGNSILNYAIIRQVLEMLGIQGDVIVNGDDSIIFTNVPIP 709

DTS GNS++NYAII++ GI GDVIVNGDD IIFTN P+P

Sbjct 1277 DTSFGNSLINYAIIKKAYRXHGIDGDVIVNGDDCIIFTNEPLP 1405

Range 4: 864 to 1055GenBankGraphicsNext MatchPrevious MatchFirst Match

Alignment statistics for match #4

Score Expect Method Identities Positives Gaps

Frame

68.2 bits(165) 7e-36 Compositional matrix adjust. 31/65(48%) 46/65(70%) 1/65(1%)

+3

Query 547 IQARHPSFNIEYGRYLKPLEMLLFHSHREGYHFGKGTVDQVSANINKLRKKYRYFTELDH 606

+ ARHPSFNI YG+Y+KPLE + H + ++FGKG ++ I L KY+Y++ELDH

Sbjct 864 LDARHPSFNIAYGKYIKPLEYKVCHG-KHKHNFGKGNYLEMGERIRVLPMKYKYYSELDH 1040

Query 607 TSWDA 611

+++DA

Sbjct 1041 SNFDA 1055

Range 5: 1413 to 1553GenBankGraphicsNext MatchPrevious MatchFirst Match

Alignment statistics for match #5

Score Expect Method Identities Positives Gaps

Frame

38.1 bits(87) 7e-36 Compositional matrix adjust. 19/48(40%) 30/48(62%) 1/48(2%)

+3

Query 714 ERLMKMFNQESKLKPSTQNIHTVEYCRTKLIVTAEGETTLLFDPQRSV 761

R ++N E+KLKPS +NIH VE+ R ++V + TL+ DP ++

Sbjct 1413 SRTRSVYNTEAKLKPSLRNIHQVEFFRCTVVVNTLVQWTLM-DPGSTI 1553

DownloadGenBankGraphics Sort by: NextPreviousDescriptions

Machilis hrabei contig_111567, whole genome shotgun sequence

Sequence ID: QVQU01111567.1Length: 6307Number of Matches: 2

Related Information

Range 1: 1982 to 2419GenBankGraphicsNext MatchPrevious Match

Alignment statistics for match #1

Score Expect Method Identities Positives Gaps

Frame

123 bits(308) 2e-28 Compositional matrix adjust. 63/147(43%) 88/147(59%) 1/147(0%)

-1

Query 445 YYYPSGTDTTIASYANRHEPVILPEYQPRLIPGIYRHIRRFLCHPRVWTRDEYCSSHTTS 504

YYYP+ T +T+A A RH L EY P + +RH+R+F+ +T +Y S TS

Sbjct 2419 YYYPASTISTVAGVAKRHCVDPLLEYNPSSVRRCFRHLRKFITRYNSFTP*QYVESMPTS 2240

Query 505 SKRQFYRDVLERMEETGKISSTIKPFTKLEKFNMSKYKAPRCIQARHPSFNIEYGRYLKP 564

R YR VL+ + G + +++ PFTK EK + + YK PR IQARHPSFNI YGR++KP

Sbjct 2239 RARNHYRGVLDHLNNGGSVRTSVTPFTKFEKMSGTHYKPPRLIQARHPSFNIAYGRFIKP 2060

Query 565 LEMLLFHSHREGYHFGKGTVDQVSANI 591

LE + H R ++FGKG ++ I

Sbjct 2059 LEHKVCHG-RYKFNFGKGNYLEMGTRI 1982

Range 2: 1918 to 1974GenBankGraphicsNext MatchPrevious MatchFirst Match

Alignment statistics for match #2

Score Expect Method Identities Positives Gaps

Frame

27.7 bits(60) 2e-28 Compositional matrix adjust. 10/19(53%) 17/19(89%) 0/19(0%)

-2

Query 594 LRKKYRYFTELDHTSWDAH 612

L +KY+Y+TELDH++++ H

Sbjct 1974 LARKYKYYTELDHSNFE*H 1918

DownloadGenBankGraphics Sort by: NextPreviousDescriptions

Machilis hrabei contig_29330, whole genome shotgun sequence

Sequence ID: QVQU01029330.1Length: 6886Number of Matches: 2

Related Information

Range 1: 5680 to 5991GenBankGraphicsNext MatchPrevious Match

Alignment statistics for match #1

Score Expect Method Identities Positives Gaps

Frame

84.7 bits(208) 7e-15 Compositional matrix adjust. 46/104(44%) 62/104(59%) 0/104(0%)

-2

Query 444 HYYYPSGTDTTIASYANRHEPVILPEYQPRLIPGIYRHIRRFLCHPRVWTRDEYCSSHTT 503

+YYP+ + +TIA+ A RH PEY RL +RH+RRF +TR+E S T

Sbjct 5991 QFYYPTCSASTIAAVARRHCIDNCPEYDKRLTRAAFRHLRRFXVRCTPFTREE*FESMPT 5812

Query 504 SSKRQFYRDVLERMEETGKISSTIKPFTKLEKFNMSKYKAPRCI 547

S R YR+VL +E G++ S ++PFTKLEKF+ YK PR I

Sbjct 5811 SRARSTYRNVLADLEVGGRVRSRVEPFTKLEKFSGRTYKPPRLI 5680

Range 2: 6372 to 6506GenBankGraphicsNext MatchPrevious MatchFirst Match

Alignment statistics for match #2

Score Expect Method Identities Positives Gaps

Frame

37.7 bits(86) 1.4 Compositional matrix adjust. 17/45(38%) 28/45(62%) 0/45(0%)

-3

Query 34 QREDAVAYLNVRCRGKRMDKNYVTYITTLFNEWCDAHAIRDYKIR 78

Q++DA+A+L V G++MD+NY Y T + E+ A+ D I+

Sbjct 6506 QQQDALAWLRVTTAGRQMDRNYEAYCTAILREYFVDRAMVDANIQ 6372

DownloadGenBankGraphics NextPreviousDescriptions

Machilis hrabei contig_29331, whole genome shotgun sequence

Sequence ID: QVQU01029331.1Length: 1282Number of Matches: 1

Related Information

Range 1: 868 to 1179GenBankGraphicsNext MatchPrevious Match

Alignment statistics for match #1

Score Expect Method Identities Positives Gaps

Frame

82.0 bits(201) 1e-14 Compositional matrix adjust. 44/104(42%) 61/104(58%) 0/104(0%)

-2

Query 444 HYYYPSGTDTTIASYANRHEPVILPEYQPRLIPGIYRHIRRFLCHPRVWTRDEYCSSHTT 503

+YYP+ + +TIA+ + RH PEY RL + H+RRF+ +TR EY S T

Sbjct 1179 QFYYPTCSASTIAAVSRRHCIDNCPEYDKRLTRAAFXHLRRFIVRCTPFTRVEYVKSMPT 1000

Query 504 SSKRQFYRDVLERMEETGKISSTIKPFTKLEKFNMSKYKAPRCI 547

S R YR+VL +E G++ S ++PFTKLEK + YK PR I

Sbjct 999 SRARSTYRNVLADLEVGGRVRSRVEPFTKLEKLSG*TYKPPRLI 868

DownloadGenBankGraphics NextPreviousDescriptions

Machilis hrabei contig_276258, whole genome shotgun sequence

Sequence ID: QVQU01276258.1Length: 4248Number of Matches: 1

Related Information

Range 1: 3518 to 3805GenBankGraphicsNext MatchPrevious Match

Alignment statistics for match #1

Score Expect Method Identities Positives Gaps

Frame

73.2 bits(178) 2e-11 Compositional matrix adjust. 36/96(38%) 60/96(62%) 0/96(0%)

-3

Query 33 NQREDAVAYLNVRCRGKRMDKNYVTYITTLFNEWCDAHAIRDYKIRYEMMNDVLLQHRNQ 92

N+R+DA+A+L VR GK MD+NY +Y+ L E+ I +Y+++ E + ++ +H ++

Sbjct 3805 NERQDALAFLYVRTLGKVMDENYESYLRALMTEYFSHRKIVNYRVQREYLQKIIPEHISE 3626

Query 93 KLQEPDFWNEQIIRRAWEHNDANQGRDYTWQWWTPW 128

KL EP + Q+IRRA ++N QG + WW P+

Sbjct 3625 KLTEPVIFTPQMIRRANDYNKELQGINTKSVWWWPF 3518

Endogenous totivirus

>QVQU01134226.1 Machilis hrabei contig_134226, whole genome shotgun sequence

Length=4728

Score = 58.2 bits (201), Expect = 8e-07, Method: Compositional matrix adjust.

Identities = 96/388 (25%), Positives = 184/388 (47%), Gaps = 34/388 (9%)

Frame = -1

Query 513 PNRGRWTRAQYLEDFDFALEQGYKQMFMKPKPVRVDSFVEFWKLRRQWVAKGSTVLNKIP 572

P+R +W ++E++ L YKQ+ PK+ + + +F K R++WV+ GS +K+

Sbjct 1386 PSR-QWVVQTFFEELSAELKLIYKQLV--PKAPAIPNWADFIKRRQNWVSAGSGGGHKV- 1219

Query 573 PEMLTYVVQFGDKLSQQIQMRHNKKSLFESHQVIDLLNETAESWNTTKVVPKLNETGKKR 632

+ + +R +K LFE + +++ E + V + E GK R

Sbjct 1218 -------------MVEGESIRIKKPVLFEELETSEMVTWLDEEPKIIAVGSEKYEMGKGR 1078

Query 633 ELLPGTLMHYLVFSYVLYVAEKQ-APVGSTRLNVNDDDNI-KYYDRKMME---GIHHMLY 687

+ +Y + SYVLY E + V++ + D D++ R + I+ +

Sbjct 1077 SIYGTKPKDYTIMSYVLYAIEPHLNRVDGIESGLVDLDQVFSVLKRSRLARESRIEGTMI 898

Query 688 DWANFNAQHSTEDMAKVISYLA---RIPNTPADYGHFCMAIAESFSQMWVMDPAG-GKHK 743

D A+FN QH+ E + V+ L + ++ AD + C A+++ + WV P + +

Sbjct 897 DYADFNYQHTLEAQSEVFIALRERLKTVSANADLIRACDWCAQALLNQWVHFPMEKSALR 718

Query 744 IEKGLFSGWRGTTWINTVLNYVYVSIGVECCKRIYN-DFQPTYFDHGGDDL---DAGFLM 799

+G+FSG RGT INT+LN Y ++ + + + + Y H GDD+ +++ L

Sbjct 717 STQGMFSGVRGTNCINTLLNLAYFRVAERQVSKCFGLHARKLYHIHQGDDVWITNGSRLW 538

Query 800 PHDCYRLMEVMDKIGYEATAIKQMIGYD-AEFYRNTINERGVFASPSRALANFVSGNWES 858

+ Y M M G+ + KQM++ + + F R + G + RA++ + G+ +S

Sbjct 537 AITLYNTMIAM---GFVFQSSKQMFDTERGKFLRVNYDRSGCRGYLMRAVPTMIVGQMQS 367

Query 859 GGAKTLSEKTTSVLDQVSKLKRRGVKEV 886

+ + ++S+ Q++ L RRG+ +

Sbjct 366 VEEHSPAAMAASLNSQINLLHRRGMSTM 283

>QVQU01024963.1 Machilis hrabei contig_24963, whole genome shotgun sequence

Length=11753

Score = 55.3 bits (190), Expect = 7e-06, Method: Compositional matrix adjust.

Identities = 99/405 (24%), Positives = 197/405 (49%), Gaps = 41/405 (10%)

Frame = -1

Query 517 RWTRAQYLEDFDFALEQGYKQMFMKPKPVRVDSFVEFWKLRRQWVAKGSTVLNKIPPEML 576

RW R +L + + ++ ++ + PK+ +V + +F K R++WV+ GS K+

Sbjct 4439 RWIR--FL*GVE---GRAETHLQLVPKAPHVPTWPDFIKRRQNWVSAGSDGGLKV----- 4290

Query 577 TYVVQFGDKLSQQIQMRHNKKSLFESHQVIDLLNETAESWNTTKVVPKLNETGKKRELLP 636

+ + +R +K LFE + +++ + + V + E GK R

Sbjct 4289 ---------MVEGESIRIKKPVLFEELETKEMVTWIDDEPKIVAVGSEKYEMGKGRSTYG 4137

Query 637 GTLMHYLVFSYVLYVAEKQ----APVGSTRLNVNDDDNIKYYDRKMMEG-IHHMLYDWAN 691

+Y + SYVLY E + + + S +++++ +I R E+ I+ + D A+

Sbjct 4136 TKPKDYTIMSYVLYAIEPHLYRMSGIESGLVDLDQVFSILKRSRLQKEARIEGTMIDYAD 3957

Query 692 FNAQHSTEDMAKVISYLA---RIPNTPADYGHFCMAIAESFSQMWVMDPAGGKH-KIEKG 747

FN+ H+ + + V+ L R ++ D + C +A+++ + WV P + +G

Sbjct 3956 FNSPHTLQA*SEVFIALRERLRTLSANVDLIRACDWFAQALLNQWVHFPMEKEALQSTQG 3777

Query 748 LFSGWRGTTWINTVLNYVYVSIGVECCKRIYNDFQPT--YFDHGGDDL---DAGFLMPHD 802

+FSG RGT INT+LN Y ++ R + ++++T Y H GDD+ +++ L +

Sbjct 3776 MFSGVRGTNFINTILNLAYFRVAEKQVSRCF-GLHSTKLYHIHQGDDVWITNGSRLWAIT 3600

Query 803 CYRLMEVMDKIGYEATAIKQMIGYD-AEFYRNTINERGVFASPSRALANFVSGNWESGGA 861

Y + M +G+ + KQM++ + +EF R ++ G + R L+ + G+ +S

Sbjct 3599 LY---NTMISMGFVFQGSKQMFDTERGEFLRVNYDHTGCRGYLMRVLPTMIVGQMQSIEE 3429

Query 862 KTLSEKTTSVLDQVSKLKRRGVKEVFCDKLIKMTLNHWLKIKIEE 906

+ ++S+ Q++ L RRG++ C L MT++H L+ I+

Sbjct 3428 HS---PAASLNSQINLLHRRGMNTRTCKSLWDMTVKHYLRSVIDG 3303

Machilis hrabei endogenised Tenuivirus/Phlebovirus nucleocapsid protein (Bunya) (QVQU01294448, 2913 bp) 20-30% id with phleboviruses

MCDYPQESPFELIDHLAGESXEDDVSECLXGLTNDVSNGDWYDSWXXHVFLPSHQ

GFDPFIIYNELSKKEPDEDVLQKDLLYLAFTYGVRGTNLIKMKKNSTPEFVSEIQRLALK

YSIEEITPSDDKTITMARIASTVPHLVCMCMKFFEDDALLVHPQQLPSNYPRVVMNAQFA

RLIPRNLPGITLNLVKAHLLYLIHLDQVVNKGKTIIRGMVEFQVSAMQWPMFSDDDRINF

CVQNGIIINTDAGLALNENLVTAATVINANNTTANILYKTLLYDLADDDDIDKVVRQQLE

ELVLDAGK*


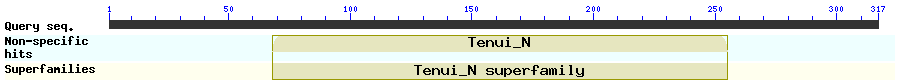


endogenised chuviruses

Machilis hrabei contig_249695, whole genome shotgun sequence//

Sequence ID: QVQU01249695.1Length: 8568Number of Matches: 1

Range 1: 3042 to 4721GenBankGraphicsNext MatchPrevious Match

Alignment statistics for match #1

Score Expect Method Identities Positives Gaps

Frame

205 bits(522) 5e-51 Compositional matrix adjust. 159/578(28%) 268/578(46%) 26/578(4%)

+3

Query 405 NFYNKHKN-YPIFEHIPDDPALKHILFSPLAYKDHS-NYHIWKSISLKTWNQCEFGKNAE 462

F+ KHK P+F P L + D S H ++S+S+ + +

Sbjct 3042 QFFIKHKV*PPVFLDPGTSPGLTWCILHNKWISDRSYPIHKYESLSVINFETVSLQPCSH 3221

Query 463 FDFIDHGMLLLKDRAACRTRASFLAEIKGESRLAVEETKVLLQYLMSDDNSG-TREYIER 521

FD++D MLLLKD A T S L + +R +++E + LL +LM+ + R Y+++

Sbjct 3222 FDYVDSQMLLLKDTALAFT-ISILENPELHARPSLKERRTLLFFLMTQNPPAEVRRYMDQ 3398

Query 522 YMEDDWGDEVLNYLVIKLTAKELEFKYKGRFFGASPIYERNRRIIQESNITRLMAHYIPD 581

++E D L+Y VIKLT K + K +G+ FG SP YER +R + E N++RLM Y

Sbjct 3399 FVEKD--PLALDYFVIKLTQKARDLKVEGKLFGQSPYYERAKRCVAEKNVSRLMKRYNSC 3572

Query 582 QLLTPPELSIIKKLMHYRTMRTLYPDSVVLNISFDFSSWNNRMRSGTVD-VAANVLDGWF 640

Q +T EL KK+ + DS V++ S D +NN R + + D F

Sbjct 3573 QAMTLTELQKQKKMYIMSQLANKMKDSYVVHFSLDAEGFNNCFRRVLCEPIGREFFDRLF 3752

Query 641 GVKLYGKTMKAFQHALVIYKDGKFVRQWIGQEGGIEGLNQATWSWVFIGGMRHALEKTGF 700

GV Y + M F +L D F W GQ+GGIEGL Q W+W++ TG

Sbjct 3753 GVNHYARVMDIF*QSLFCC-DEFFRYNWTGQKGGIEGLAQKFWTWIYEAVASRIASLTGN 3929

Query 701 KYQITVKGDDVRAALVVPKNTLAIRSMDNIKSEIMSNIQELCAHVGWKLNPNECYISRTL 760

K+ + V GDD+R L++PK T+++ + ++ +EI Q + + E Y S L

Sbjct 3930 KFFVMVNGDDLRVTLIIPKETVSLAQLSDLITEIAHAFQAEYESFVFNVKLEETYASSVL 4109

Query 761 MSTSKSYFVNDVHLPNATKKFMRSEGNTN----IPFPTLEDTVGTCFSIAHSVCYNSTSI 816

+ K YFVN V K + G N +P ++ + + V N I

Sbjct 4110 LGFGKVYFVNSVCSSTTLKMGCKMHGLANLIGDLPVEYIKGVMSETMATI-GVSSNHRFI 4286

Query 817 FPAYLGALLQATKAIHASYKNKPEFNSWKTSRCVVKDFLVVMLCWPQVIGGPGSLPLQTF 876

+ +L + + S K + KD +V+ML +P GG LP F

Sbjct 4287 YILWLVVVQFYLENSLGSLFQK-----------MSKDQMVLMLLFPSNFGGIPVLPYIRF 4433

Query 877 LVRGENDMLSVSVSLMRYILFRGSQNELQSVVRILNQKIDDRQNFDTMLLGDPYAIPLST 936

L +G++D+ +V +SL ++I +V +++ + + L +P+++PLST

Sbjct 4434 LAKGDSDLETVWISLYKHIQ-TCDLPIFHRLVYLMSVCLKCSTQYQG-LASNPFSLPLST 4607

Query 937 PPRPQSLLKQMMRKAMRRITKQTDVKQLLDKSISVSEL 974

P ++L+ ++ + + + + +++ + ++L

Sbjct 4608 PSDGIAVLEGAIKSVLPGLVENIEFAKIIHAANLCAQL 4721

Machilis hrabei contig_249695, whole genome shotgun sequence

Sequence ID: QVQU01249695.1Length: 8568Number of Matches: 2

Range 1: 3042 to 4706GenBankGraphicsNext MatchPrevious Match

Alignment statistics for match #1

Score Expect Method Identities Positives Gaps

Frame

182 bits(463) 7e-54 Compositional matrix adjust. 137/579(24%) 263/579(45%) 43/579(7%)

+3

Query 381 HYYKRHRKWPLCDMSPETPVRVARIILHN--LDPFSLETMRRYGGIPLEAYDYIQFRKLR 438

++ +H+ P + P T + ILHN + S + +Y + + ++ + +

Sbjct 3042 QFFIKHKV*PPVFLDPGTSPGLTWCILHNKWISDRSYP-IHKYESLSVINFETVSLQPCS 3218

Query 439 EFEWLENFIPYIKDR----TISALRSDIMRVYFSETGETGRIPWQDTRLLLFYLMSPMSR 494

F+++++ + +KD TIS L + + R ++ R LLF+LM+

Sbjct 3219 HFDYVDSQMLLLKDTALAFTISILENPELH---------ARPSLKERRTLLFFLMTQNPP 3371

Query 495 LNHVKYMHAYVAGQWEMIQDYLIIRIVPKEKEHKVEARGFGCKPYEDRARTIVQEYNTAG 554

+YM +V + DY +I++ K ++ KVE + FG PY +RA+ V E N +

Sbjct 3372 AEVRRYMDQFVEKD-PLALDYFVIKLTQKARDLKVEGKLFGQSPYYERAKRCVAEKNVSR 3548

Query 555 ILHDYSSEHVMTLDELGLAKKLLAFRSMAKAYRGYRMLILSVDASAWNNAFRGEAIHPIM 614

++ Y+S MTL EL KK+ +A + ++ S+DA +NN FR PI

Sbjct 3549 LMKRYNSCQAMTLTELQKQKKMYIMSQLANKMKDSYVVHFSLDAEGFNNCFRRVLCEPIG 3728

Query 615 EETLDRFYDVDLWSKTQTAYERSFIYVPDVERMYSWDGQAGGIEGLNQ--YTWVYAYIHQ 672

E DR + V+ +++ + +S + R Y+W GQ GGIEGL Q +TW+Y +

Sbjct 3729 REFFDRLFGVNHYARVMDIF*QSLFCCDEFFR-YNWTGQKGGIEGLAQKFWTWIYEAVAS 3905

Query 673 MKVCLRDQPYPYYILCKGDDLRVAVLVAPDYLEAISIDALKVELLESVASIGRKFGHSIK 732

L ++++ GDDLRV +++ + + + L E+ + + F ++K

Sbjct 3906 RIASLTGN--KFFVMVNGDDLRVTLIIPKETVSLAQLSDLITEIAHAFQAEYESFVFNVK 4079

Query 733 VEDSYASESYFAFSKDAYVEGAEQSQAMRKVQKCYGANNAFINILDEYVASAFSNAHSAS 792

+E++YAS F K +V S ++ K +G N ++ EY+ S +

Sbjct 4080 LEETYASSVLLGFGKVYFVNSVCSSTTLKMGCKMHGLANLIGDLPVEYIKGVMSETMATI 4259

Query 793 KVAPSPVATYCVGVWWALVALLMDKR----YKELADWELVACMLVPNILGGFPIIYLHNM 848

V+ + Y +W +V ++ +++++ ++V +L P+ GG P++

Sbjct 4260 GVSSNHRFIYI--LWLVVVQFYLENSLGSLFQKMSKDQMVLMLLFPSNFGGIPVLPYIRF 4433

Query 849 FTRAESDLLPPFLDLCRYAQ-------EHVPHLATILLRAWRQKLAPVHRCLSGLMMDIY 901

+ +SDL ++ L ++ Q + +L ++ L+ Q GL + +

Sbjct 4434 LAKGDSDLETVWISLYKHIQTCDLPIFHRLVYLMSVCLKCSTQ--------YQGLASNPF 4589

Query 902 SLPITKPSSATTILRREMSHMLQDRTQNEALQALFRAAS 940

SLP++ PS +L + +L +N + AA+

Sbjct 4590 SLPLSTPSDGIAVLEGAIKSVLPGLVENIEFAKIIHAAN 4706

Range 2: 4724 to 5626GenBankGraphicsNext MatchPrevious MatchFirst Match

Alignment statistics for match #2

Score Expect Method Identities Positives Gaps

Frame

54.7 bits(130) 7e-54 Compositional matrix adjust. 75/328(23%) 133/328(40%) 50/328(15%)

+2

Query 947 LLLAYQEANVYNVKLMSALFDCLPEAIIRELVRKFESGKSIYLALH----RGRGFRRAQS 1002

+ A +++ K S LF+ P + EL KF +S++ L R RG R

Sbjct 4724 FIQALFSSHIIEAKAFSVLFETSPPGQVIELASKFTGTRSVFNLLQVKGRRHRGLR---- 4891

Query 1003 IVRQAYKADARMHQFRIELLT---RGVLKAVELLPADWAQRCPGEVCAEIRSQLWEKPII 1059

++++A K D + ++T R +++ ++ RCP + ++R LW+K +

Sbjct 4892 VLKRARKEDLAKLSYSARIITGELRNLVENRQITIILSRDRCPTLISEQLRDFLWQKRVK 5071

Query 1060 GVTQPPPQHQIYGGWVDSIEPTYYTLRNHFELWHTHPSGD------------RPHLLSVG 1107

G T P ++D + RN ++ T SG +P +

Sbjct 5072 GFTYP--------CFLDQ-----FNFRNCRDMSFTGKSGGGFNHSTVLVAARKPGEIPCY 5212

Query 1108 EYTPF---VGSITGRGLSKPHVELKTQNIVSMKIHTLLDVYQWSRVCKSFEAHDMVGNLW 1164

+ PF GS T L K VE+ + ++ +L VY + F +V

Sbjct 5213 SHGPFPQYPGSHTDMKLIKSAVEIHGSSPGVGRVTKILQVYPF---MVRFGERALV---- 5371

Query 1165 QICESLIEDYTGRSIKA-FLPYAGDTFINKTIQHHLRAHNYRASIVPNTLMNIYTTMKGN 1223

+ED TG IKA L A + ++ H + +++ + + PN L N T +K

Sbjct 5372 -YLNRSLEDITG--IKAELLTAASYRSSSGSVAHRVPLNHWSSMVGPNKLPNKTTYVKVI 5542

Query 1224 IYAHRIFKTSVDHYKMNYLQIMCHMISL 1251

+ R KT + Y +N+ I ++L

Sbjct 5543 MTTDRKLKTIIADYPLNFNYIKTLFMAL 5626

endogenous nyamivirus glycoproteins in Machilis

Machilis hrabei contig_332204, whole genome shotgun sequence

Sequence ID: QVQU01332204.1Length: 9701Number of Matches: 4

Range 1: 2124 to 2891GenBankGraphicsNext MatchPrevious Match

Alignment statistics for match #1

Score Expect Method Identities Positives Gaps

Frame

124 bits(312) 9e-40 Compositional matrix adjust. 78/270(29%) 136/270(50%) 21/270(7%)

+3

Query 370 QSPGYTATVRGEVIHVGKCQPVHVNYTSSIDKCYNELPVTYDGNLAFMLPRTRILSKIGT 429

++PGYTA GEV+H+ KC P V + +CY+E+PV Y F+ P++R + K GT

Sbjct 2124 ETPGYTAITLGEVVHLAKCAPEEV-IMRNT*QCYHEIPVLYQNQSVFLTPKSRPIQKHGT 2300

Query 430 EVDCSGLINIMYKLTDSWYSVSRDLIHTHK-PEIISITPNDI--WEFKMISGLAESGIYS 486

++DC L+N ++L WY++ H P I +TP+ + W+ + LA GIY+

Sbjct 2301 QIDCDNLVNAQFRLDGQWYAIGNS---KHPVPPPIELTPDPV*HWKGYSFAELARYGIYT 2471

Query 487 QRDLDQVQKILMNPVEKEILSSRILRTLDGASSLPTGYSLYNTFTPQDLERLTKNTVSTF 546

+ +++++ +MNP E+ +++ + R + GA G SL + E + K+

Sbjct 2472 FDEPERLREWIMNPYERGAIANILTRGVTGAHV*TQGISLSSLVD----EHMIKSMGERI 2639

Query 547 FMVFYGKMTTIGNFFSFLLALFMILRFIKFILNSIINWTYLYRTVGLSWKLIFCWWENLV 606

+ GN S + L+ ILR +KF+ +++ + +Y G S L+ W+ +

Sbjct 2640 M*KIRSWTSVFGNISSGFIGLYFILRLVKFLXDTLFHCRAIYEVYGFSAALLGRAWDKVT 2819

Query 607 HHWVRDSKTQSTKQTD----QELVHIEVPI 632

+ QS K D +E +IE+PI

Sbjct 2820 ------TCIQSRKSRDCTPVKEKPYIELPI 2891

Range 2: 1864 to 2127GenBankGraphicsNext MatchPrevious MatchFirst Match

Alignment statistics for match #2

Score Expect Method Identities Positives Gaps

Frame

63.9 bits(154) 9e-40 Compositional matrix adjust. 34/90(38%) 53/90(58%) 2/90(2%)

+1

Query 281 TEHPKLSIIEEQQNLGFNLKPDKTLFNKEVNLMTYFNSKLLYIMKHTKDQVDSLYQKISH 340

TEHPKL II + GF +L ++LM Y N+K +YI K +S+YQ+++

Sbjct 1864 TEHPKLVIIPDA-GTGFYFT-RHSLHPGTMDLMAYVNAKFVYIEKRRSRSAESMYQELAT 2037

Query 341 DRCNSETRIVNSMMTLALISPLEFAYEYFQ 370

RC E R + +++++A +SP EFAY Y +

Sbjct 2038 QRCMIERRSLMNLLSMASVSPTEFAYIYMK 2127

Range 3: 3921 to 4724GenBankGraphicsNext MatchPrevious MatchFirst Match

Alignment statistics for match #3

Score Expect Method Identities Positives Gaps

Frame

152 bits(383) 2e-36 Compositional matrix adjust. 86/275(31%) 150/275(54%) 11/275(4%)

+3

Query 228 QEYTVLFEGACTKIIETKD-GFSITSYLMNIDDYDFQITRRDKQIRLCGQLGWATEHPKL 286

+++ V +EG K ++ + S T Y+++ + F + R + C TEHPKL

Sbjct 3921 RKHLV*YEGVAQKFLQLDEYNVSTTVYMVDSGERVFGL-RTTGYSKGCMFKALYTEHPKL 4097

Query 287 SIIEEQQNLGFNLKPDKTLFNKEVNLMTYFNSKLLYIMKHTKDQVDSLYQKISHDRCNSE 346

II + + +L ++LM Y N+ +YI K +S+YQ+++ RC E

Sbjct 4098 VIIPNAGTXFYFTR--YSLHPGTMDLMAYVNATFVYIEKRRSRSAESMYQELATQRCMIE 4271

Query 347 TRIVNSMMTLALISPLEFAYEYFQSPGYTATVRGEVIHVGKCQPVHVNYTSSIDKCYNEL 406

R + +++++A +SP EFAY Y +P YTA GEV+H+ KC PV V + ++CY+E+

Sbjct 4272 RRSLMNLLSMASVSPTEFAYTYMNAPAYTAITLGEVLHLAKCAPVDV-IMHNTEQCYHEI 4448

Query 407 PVTYDGNLAFMLPRTRILSKIGTEVDCSGLINIMYKLTDSWYSVSRDLIHTHK-PEIISI 465

V Y F+ P++R++ K GT++DC L+N Y+L WY++ H P I +

Sbjct 4449 SVMYQNQPVFLTPKSRLIQKHGTQIDCDNLVNAQYRLDGQWYAIGNS---KHPVPPPIEL 4619

Query 466 TPNDI--WEFKMISGLAESGIYSQRDLDQVQKILM 498

TP+ + W+ + LA SGIY+ + +++++ +M

Sbjct 4620 TPDPVRHWKGYSFAELARSGIYTFDETERLREQIM 4724

Range 4: 3310 to 3555GenBankGraphicsNext MatchPrevious MatchFirst Match

Alignment statistics for match #4

Score Expect Method Identities Positives Gaps

Frame

35.8 bits(81) 4.5 Compositional matrix adjust. 28/85(33%) 40/85(47%) 5/85(5%)

+1

Query 3 ILKLLFNYILFVHYSYALIAFDCESKISERRTFSLVETNPCIPIVHNITTSIEKIQVVQP 62

+LK L I VH+ Y+L+ FDC + T +L + PC EK+Q+VQ

Sbjct 3310 MLKFLV-LICLVHHGYSLLGFDCFHPMVNVSTXALDKVPPCHVEDPMSQAKSEKVQLVQ- 3483

Query 63 RVFDK--LEYIQCMITISHQIFRCG 85

+ DK + QC IS + CG

Sbjct 3484 -LADKYPVHVYQCKTVISRIVTNCG 3555

DownloadGenBankGraphics Sort by: NextPreviousDescriptions

Machilis hrabei contig_21209, whole genome shotgun sequence

Sequence ID: QVQU01021209.1Length: 2112Number of Matches: 2

Range 1: 34 to 1188GenBankGraphicsNext MatchPrevious Match

Alignment statistics for match #1

Score Expect Method Identities Positives Gaps

Frame

124 bits(310) 9e-28 Compositional matrix adjust. 113/411(27%) 179/411(43%) 40/411(9%)

-1

Query 11 ILFVHYSYALIAFDC------ESKISERRTFSLVETNPCIPIVHNITTSIEKIQVVQPRV 64

I VHYS LI FDC S I+ R S +P P E+IQ+VQ

Sbjct 1188 IGLVHYSQCLIGFDCFHPMVNVSTIAHDRVPSCHLDDPPSP------AKCERIQLVQLAD 1027

Query 65 FDKLEYIQCMITISHQIFRCGKTIDTFQAGGIYSEVVE------VSRSQCEDLHKLRTFN 118

+ QC I +S + CG + ++ + VS E LH++

Sbjct 1026 NYPVHVYQCKIVVSRLVTHCGMHSHSSAVDWWVHDIYQAH*QERVS*YARETLHEI---- 859

Query 119 YFGVQIKLEKGNSVTKLSTETFGSIDSDGSCTPGNGQLHANN-RVYSRAVRTSNIEITLI 177

G+ N+ + G+I G CT G +++ + V +I TL

Sbjct 858 LLGI-------NTSRSVPMTLGGNISISGGCT---GTSYSDVFGSWEYVVVQGSITKTLK 709

Query 178 KSLGTIDIDEKKFILEDTTKCRYEDFECFSVNNGYSYWEEANDKIHCPESQEYTVLFEGA 237

D D L C +C Y+ WE +++ C +++++ VL+EG

Sbjct 708 DYTALADTDRDIINLXSGVTCPASRGDCMDTEVXYTMWE-SSEVTRC-DARKHVVLYEGV 535

Query 238 CTKIIETKD-GFSITSYLMNIDDYDFQITRRDKQIRLCGQLGWATEHPKLSIIEEQQNLG 296

K + + S T Y+++ + F + R ++C TEHPKL II + G

Sbjct 534 SQKFNQLDEYNVSTTVYMVDSGERVFGL-RTTGYYKICMFKALHTEHPKLVIIPDA-GTG 361

Query 297 FNLKPDKTLFNKEVNLMTYFNSKLLYIMKHTKDQVDSLYQKISHDRCNSETRIVNSMMTL 356

F +L + LM Y N+K +Y+ K +Y++++ RC E R + +++T+

Sbjct 360 FYFT-RHSLHPGTMXLMAYVNAKFVYVEKRRARTTKLMYRELATQRCMIERRSLMNLLTM 184

Query 357 ALISPLEFAYEYFQSPGYTATVRGEVIHVGKCQPVHVNYTSSIDKCYNELP 407

A ISP EF Y Y +PGYTA GEV+++ KC V V S+ +CY+E+P

Sbjct 183 ASISPTEFPYTYMNAPGYTAVTLGEVVNLAKCTSVDVQ-VSNTGQCYHEIP 34

Range 2: 1671 to 1943GenBankGraphicsNext MatchPrevious MatchFirst Match

Alignment statistics for match #2

Score Expect Method Identities Positives Gaps

Frame

37.7 bits(86) 0.88 Compositional matrix adjust. 28/94(30%) 47/94(50%) 6/94(6%)

-2

Query 436 LINIMYKLTDSWYSVSRDLIHTHK-PEIISITPNDIWEFKMIS--GLAESGIYSQRDLDQ 492

L+N ++L WY V H P I +TP+ + K S LA SGIY+ + ++

Sbjct 1943 LVNAQFRLEGQWYDVGXS---KHPVPAPIELTPDPVRV*KGYSFAELARSGIYTFDETER 1773

Query 493 VQKILMNPVEKEILSSRILRTLDGASSLPTGYSL 526

+++ MNP E+ +++ + R + A G SL

Sbjct 1772 LRERFMNPYERGTIANILTRGVT*AHVQIPGISL 1671

DownloadGenBankGraphics Sort by: NextPreviousDescriptions

Machilis hrabei contig_107066, whole genome shotgun sequence

Sequence ID: QVQU01107066.1Length: 2757Number of Matches: 2

Range 1: 1351 to 1941GenBankGraphicsNext MatchPrevious Match

Alignment statistics for match #1

Score Expect Method Identities Positives Gaps

Frame

93.6 bits(231) 5e-18 Compositional matrix adjust. 63/211(30%) 101/211(47%) 15/211(7%)

+1

Query 198 CRYEDFECFSVNNGYSYWEEANDKIHCPESQEYTVLFEGACTKIIET-KDGFSITSYLMN 256

C +C GY+ WE + + C + +++ VL+EG + ++ S T Y+++

Sbjct 1351 CPASRGDCMDTEVGYTMWE-STEMTRC-DQRKHMVLYEGVAQMFAQLDENNVSSTVYMVD 1524

Query 257 IDDYDFQITRRDKQIRLCGQLGWATEHPKLSIIEEQQNLGFNLKPDKTLFNKEVNLMTYF 316

++ F + R + C W TEHPKL II + GF + +L LM Y

Sbjct 1525 SEEMVF*L-RTTGYYKGCMFKAWHTEHPKLVIIPDA-GTGFYFR-RHSLHPGTXXLMAYV 1695

Query 317 NSKLLYIMKHTKDQVDSLYQKISHDRCNSETRIVNSMMTLALISPLEFAYEYFQSPGYTA 376

N+K +Y+ K ++ + C E R + +++T+ ISP EFAY Y +PGYTA

Sbjct 1696 NAKFVYVEKRSR---------TTDLMCMIERRSLMNLLTMPSISPTEFAYMYMNAPGYTA 1848

Query 377 TVRGEVIHVGKCQPVHVNYTSSIDKCYNELP 407

GE +H+ KC PV V + + Y P

Sbjct 1849 ITLGEGVHLAKCAPVVVQVNNQLYILYTGCP 1941

Range 2: 794 to 1075GenBankGraphicsNext MatchPrevious MatchFirst Match

Alignment statistics for match #2

Score Expect Method Identities Positives Gaps

Frame

35.4 bits(80) 5.0 Compositional matrix adjust. 27/99(27%) 45/99(45%) 11/99(11%)

+2

Query 14 VHYSYALIAFDCESKISERRTFSLVETNPCIPIVHNITTSI-----EKIQVVQPRVFDKL 68

VHY++ LI FDC + T +L PC ++ I EKIQ+VQ +

Sbjct 794 VHYAHCLIGFDCFHPMVNVSTIALDRVPPC-----HVDEPIGPAKSEKIQLVQLADKYPV 958

Query 69 EYIQCMITISHQIFRCG-KTIDTFQAGGIYSEVVEVSRS 106

QC +S + +CG + + GG + + +S++

Sbjct 959 HVYQCKFVVSRIVTQCGMHSHSSAVTGGYMTYIRPISKT 1075

endogenous mononegaviruses in Machilis

Machilis hrabei contig_67087, whole genome shotgun sequence

Sequence ID: QVQU01067087.1Length: 8449Number of Matches: 1

Range 1: 2674 to 3882GenBankGraphicsNext MatchPrevious Match

Alignment statistics for match #1

Score Expect Method Identities Positives Gaps

Frame

159 bits(401) 6e-37 Compositional matrix adjust. 113/406(28%) 191/406(47%) 12/406(2%)

-2

Query 582 INLDLSKWNSSYRHALVTRFGKTLDQLFGLKNFYEYNHIWFLKANVFTNSRLHPPDYDIF 641

IN+D KWN +R+ + LD LFG KN Y+ H NVF N + PP+ D

Sbjct 3882 INMDFKKWNMHFRNKACQPTFQCLDDLFGFKNVYQATHQLLTDCNVFLNCQTCPPETDRE 3703

Query 642 TKLPIPGDYYYNNHKGGMEGMRQKLWTIITIAIIKYSAETLNLRITVIGQGDNQVVLIKY 701

P+ GDY++ N GG EG+R K W+++T I + L +++G GDNQVV ++

Sbjct 3702 GN-PLRGDYFHRNQLGGFEGLRHKGWSLVTTLTIMITLRQLCYLGSMMGAGDNQVVCLRI 3526

Query 702 ----REDQIDKKSELRN---RFLQLLKTNFLAVNLKLKLSETWISKNLFEYGKVRYYKGE 754

+ +KK +R+ +FL L+ +F + LK E+W S L Y K +

Sbjct 3525 PIPDGLSEEEKKQLVRDTVDKFLTKLQEDFRLMGHTLKTQESWASSCLIAYNKKFFLNAT 3346

Query 755 AISQTTKKISRLIPDINDGISSFMSSLSTINTITESAAKMDHCPDSCFLINSISILNYLM 814

+ K+ R+ PD+ND + + ++ + T E+ AK D S F + + ++NY

Sbjct 3345 PQCTSYKRACRISPDLNDSFPTVNAEITCVATAAEATAKEDFNQISAFSLFCLELVNYFF 3166

Query 815 RRKIIHQDTPSPVCFMYLC-YPSDFGGISLSHYFSHYVRGHEDKVTMWLAYYNHLRLYYP 873

RR I + S LC + FGG+ ++ + +RGH D++T L H++ P

Sbjct 3165 RRDIFQIASRSEAEATALCLWNKTFGGLPITSLLNCTIRGHPDRLTQQLGLLFHIQRQDP 2986

Query 874 MNFEYLAHIINLIPSGKKNINRLIEDIYCLDVITLPSIEALFKEKALDYLKSDEVTNPE- 932

F+ L + + + L++ + ++++ E+L +K + + +VT E

Sbjct 2985 EVFDILKRLCSYKTPRIPSYRALVQYPHSINIMVPRDPESLVNDKI--HERMIQVTKNEY 2812

Query 933 IKKLFDSNQCISQQELIDQLKTMKPMFLPLAHEILRHSNAGILIAF 978

IK L S LI+ + +P + E+ SN GI + +

Sbjct 2811 IKTLIASASEQQVNTLIEDIIRTRPYHARMGCELFGLSNPGIFLEY 2674

DownloadGenBankGraphics Sort by: NextPreviousDescriptions

Machilis hrabei contig_38110, whole genome shotgun sequence

Sequence ID: QVQU01038110.1Length: 10736Number of Matches: 2

Range 1: 3562 to 5118GenBankGraphicsNext MatchPrevious Match

Alignment statistics for match #1

Score Expect Method Identities Positives Gaps

Frame

140 bits(353) 3e-31 Compositional matrix adjust. 144/531(27%) 227/531(42%) 54/531(10%)

+1

Query 371 RDFCQNYFKKHRRWP--NMKSYPAD--FGNFISKNLVMPKSFSN--RWNLWSKIKFDKCF 424

R C Y K RWP + P + ++I + V ++ +W ++ IK K

Sbjct 3562 RILCLAYISKPGRWPPCDTSKMPDNSVLKSWIDHSNVNIDEYAEGYQWEDFAYIKHMKWL 3741

Query 425 EYDYSVDTTELLKDTASAPPFSEWFLAYDHCAFKHlhnknkpflpknkkptplRIIS--- 481

E+D +VD TELL D ++ P S++ + YD + +P + L S

Sbjct 3742 EFDATVDYTELLNDKSATPTRSKFDVVYDSDLLGYQPTALRPSEDRRLLVNLLNRSSFNP 3921

Query 482 -RFLQGIPNEVEKKVFECTELYWHIDDSTAVVCLKEREIKND-GRLFVKQTYEQRLGQVS 539

LQ I K ++C ++ KERE K + RLF T E +

Sbjct 3922 EETLQKIQTRQVPKEWKCVGVH-----------PKEREEKGEKPRLFALLTPEIQYYFCV 4068

Query 540 SEMNIANTIFRYIPDQTMTDSEVILAQKISSAVKNQNQDYE----LINLDLSKWNSSYRH 595

+E NIA +F + QTMT SE L + + ++LD +WN ++

Sbjct 4069 TEKNIAQQLFE*VEHQTMT*SESSLLHRTLDTTQEHATPGAPIKIFVSLDFQRWNLTWDF 4248

Query 596 ALVTRFGKTLDQLFGLKNFYEYNHIWFLKANVFTNSRLHPPDYDIFTKL----PIPGDYY 651

+ + +D +FG Y Y H +F + + +SRL+PP L IPG +

Sbjct 4249 SGTFATFEMVDDIFGTPGLYTYTHEFFSECLCYLSSRLNPPPGLAKGHLGDPPEIPGYLW 4428

Query 652 YNNHKGGMEGMRQKLWTIITIAIIKYSAETLNLRITVIGQGDNQVVLI------KYREDQ 705

YN+H GG EG QKLWT +T ++ A L LR + GDNQ+ I K R Q

Sbjct 4429 YNHH-GGFEGQHQKLWTFLTNGLVLSVAVELKLRCILNSCGDNQLAKILIPNSTKDRTPQ 4605

Query 706 ID------KKSELRNRFLQLLKTNFLAVNLKLKLSETWISKNLFEYGKVRYYKGEAISQT 759

D K ++L R + LL+ + LK T++S ++ Y K +G T

Sbjct 4606 EDISCRREKINQLCKRIIHLLQERAAGIEQTLKAEATFLSDTVYIYEKEVVIRGAVAPST 4785

Query 760 TKKISRLIPDINDGISSFMSSLSTINTITESAAKMDHCPDSCFLINSISILNYL------ 813

+K SR++ D + I SF + ++TI+T +AA + +L ++I L

Sbjct 4786 LRKASRIMEDTSGTIKSFDNIVATIHTGGHAAALKGNS*VVAYLHSAIETFRTLYHDIMT 4965

Query 814 MRRKII-----HQDTPSPVCFMYLCYPSDFGGISLSHYFSHYVRGHEDKVT 859

K+I +Q V + PS FGG+ + + RGH + +T

Sbjct 4966 THGKVIGLTLKNQQEVVDVVKILCLLPSCFGGVPSLPWTDYVFRGHPEPLT 5118

Range 2: 6045 to 6236GenBankGraphicsNext MatchPrevious MatchFirst Match

Alignment statistics for match #2

Score Expect Method Identities Positives Gaps

Frame

40.0 bits(92) 0.78 Compositional matrix adjust. 22/64(34%) 34/64(53%) 0/64(0%)

+3

Query 1184 GGNMVHRFRAAIERNSAVINSLPTTGSHFRQTTNMLSAITKGGRDWTIHFQLVFLFNVSV 1243

GG+++HRF +SAVINS PT ++ T+ S ++ + IHFQ + L V

Sbjct 6045 GGSVLHRFMDVTTSHSAVINSNPTLHTNLNIHTDCFSPYSQSEVHYNIHFQGLVLQAAGV 6224

Query 1244 ISRL 1247

+ L

Sbjct 6225 VRAL 6236

DownloadGenBankGraphics NextPreviousDescriptions

Machilis hrabei contig_83516, whole genome shotgun sequence

Sequence ID: QVQU01083516.1Length: 7956Number of Matches: 1

Range 1: 2211 to 4205GenBankGraphicsNext MatchPrevious Match

Alignment statistics for match #1

Score Expect Method Identities Positives Gaps

Frame

128 bits(322) 1e-27 Compositional matrix adjust. 165/701(24%) 288/701(41%) 78/701(11%)

+3

Query 619 HIWFLKANVFTNSRLHPP---DYDIFTKLP-IPGDYYYNNHKGGMEGMRQKLWTIITIAI 674

H +F + +SRL+PP D P +PG +YN HKGG EG RQKLWT +T +

Sbjct 2211 HEFFAVCLCYLSSRLNPPPGLSKDHAGNPPDVPGHLWYN-HKGGFEGQRQKLWTFLTNGL 2387

Query 675 IKYSAETLNLRITVIGQGDNQVVLIKYREDQIDK--KSELRNR----------FLQLLKT 722

+ A L L+ + GDNQ+ I D+ + +L R +QLL+

Sbjct 2388 VLSVAIELKLKCLLNRCGDNQLATILIPNSTTDRTPQEDLSCRREEINLLCE*IIQLLQE 2567

Query 723 NFLAVNLKLKLSETWISKNLFEYGKVRYYKGEAISQTTKKISRLIPDINDGISSFMSSLS 782

LK ET++S ++ Y K +G T +K SR++ D + + SF + ++

Sbjct 2568 RAAGTGQTLKAEETFLSDTVYIYAKEVVIRGTVAPSTLRKASRIMEDTSGTVKSFDNIVA 2747

Query 783 TINTITESAAKMDHCPDSCFLINSISILNYLMRRKIIHQDTPSPVCFM------------ 830

TI+T +AA H + + I R + H PS M

Sbjct 2748 TIHTGGHTAALKGHSWMVAYFHSVIETF-----RALYHDIMPSRGKVMGLTLKTQQEVLE 2912

Query 831 ---YLC-YPSDFGGISLSHYFSHYVRGHEDKVT---MWLAYYNHLRLYYPMNFEYLAHII 883

LC PS FGG+ + + VRGH D +T + L +H +N+ Y +

Sbjct 2913 VVKILCQLPSCFGGLPSLPWTDYVVRGHPDPLTSGILALTLIDHRISRLILNYIYSLLLE 3092

Query 884 NLIPSGKKNINRLIEDIYCLDVITLPSIEALFKEKALDYLKSDEVTNPEIKKLFDSNQCI 943

P +N L+ED LD+ ++ +++ +L S NP++K+LF S

Sbjct 3093 KRNPRSLP-LN-LVEDPASLDIPGKGKTASVVRQQLEKHLPS-YTRNPDLKQLFKSAP-- 3257

Query 944 SQQELIDQLKTMKPMFLPLAHEILRHSNAGILIAFRNKLSNIATINKIIQSSEENSYLEL 1003

+ + + KP L + + + S G F +N+ ++K++++ E +

Sbjct 3258 -----LSEDRQWKPRVL---NAMYKSSPHGARNNFVATFNNLNMVDKVMKTCEAGPIYSM 3413

Query 1004 MAVNNDAVREILISKARSRKRYSLRDSLIKENCPTQLAINIRNEHWNL--DLLGASKPVP 1061

+ + R ++ +R L C A+ +R L +LG + P P

Sbjct 3414 V---KEEERRRRVNLGVIVQRIFKAPKLPGHACGRIFAVGLRLHPSGLHEPILGVTVPHP 3584

Query 1062 HHQFTIKPLDECTQEEINMSILINTSREFAQSDLG---AYNQLGPFPIFHGAATKEKINK 1118

+ L E +L+ + +DL + GPF G+AT+EK +

Sbjct 3585 S-----EVLCRSDPEYFEFILLMCHGSQHKDTDLNIPLPFLNRGPFKPELGSATREKSSS 3749

Query 1119 PKMEMFTKSSYTKSLQQLFTIGTWIQKIQG--NNLMQLIENLILEKSSHIPEEFLDQELD 1176

M +L I W+ N ++ ++ L+ S + L +++

Sbjct 3750 ELTNMEKGDRPLIEAFRLSKIKDWVSAPNSYVNKCIEYLQRLLTSADS-VVISLLAKKI- 3923

Query 1177 DWCVSTYGGNMVHRFRAAIERNSAVINSLPTTGSHFRQTTNMLSAITKGGRDWTIHFQLV 1236

GG+++HRF +SAVINS P+ ++ T+ S ++ D+ IHFQ +

Sbjct 3924 ------VGGSVLHRFMDVTTSHSAVINSNPSLQTNLTIHTDCFSPYSQSEVDYNIHFQGL 4085

Query 1237 FLFNVSVISRLKRSIPLLYTQYAAYLSCNTCTQEVSNIVMD 1277

L ++ R+ + +SC++C E+ + ++

Sbjct 4086 VLHAAGLV-RIMGLHGYDMSLNCFTISCSSCITELPEVKLN 4205

endogenous chuvirus glycoprotein

Catajapyx aquilonaris contig_4110, whole genome shotgun sequence

Sequence ID: JYFJ02004110.1Length: 34860Number of Matches: 1

Range 1: 9458 to 10387GenBankGraphicsNext MatchPrevious Match

Alignment statistics for match #1

Score Expect Method Identities Positives Gaps

Frame

108 bits(269) 5e-23 Compositional matrix adjust. 90/321(28%) 151/321(47%) 15/321(4%)

-3

Query 102 EVSRSQCEDLHKLRTFNYFGVQIKLEKG-NSVTKLSTETFGSIDS-DGSCTPGNGQLHAN 159

E +R +CE + + Y ++ N T S G DS G C+ G+ + +

Sbjct 10387 EHTRHECEMFTRTGVYRY*TSLRPIDSPLNITTHASLVIVGHTDSASGECSGGDYNI--D 10214

Query 160 NRVYSRAVRTSNIEITLIKSLGTIDIDEKKFILEDTTKCRYEDFECFSVNNGYSYWEEAN 219

VY V + I L TID + + +L +C Y+ C G ++W A

Sbjct 10213 GYVYKNVVVEVYLTIRLSDYWITIDRKKNEAVLPSGARCEYKKENCMDDFLG*TFWT-AQ 10037

Query 220 DKIHCPESQEYTVLFEGACTKIIETKDGFSITSYLMNIDDYDFQITRRDKQIRLCGQLGW 279

C + Q VL+EG ++I+ + G + + D F + D + +C Q +

Sbjct 10036 APTLC-DGQSIDVLYEGE-VRVIDPEKGRKVA--IGQDDGTAFAVEMGDGDV-MCNQHVF 9872

Query 280 ATEHPKLSIIEEQQNLGFNLKPDKTLFNKEVNLMTYF-NSKLLYIMKHTKDQVDSLYQKI 338

EHP+L +I E N GF K +++ VN+ Y+ NSK +++ +H ++ +Y +

Sbjct 9871 RMEHPQLVLIPEGTN-GFIFK-KQSILTLNVNMAAYYYNSKFVFLEQHLGSEIGRMYGIM 9698

Query 339 SHDRCNSETRIVNSMMTLALISPLEFAYEYFQS-PGYTATVRGEVIHVGKCQPVHVNYTS 397

+C + + ++ +LA I+P EFAY + PGYTA RGEV ++ KC+PV V+

Sbjct 9697 KKQQCQDRQQTLRTLQSLAYINPDEFAYALMSNEPGYTAFTRGEVCYIIKCKPVTVSLLK 9518

Query 398 SIDKCYNELPVTYDGNLAFML 418

S + CY++L V N + L

Sbjct 9517 S-NSCYSDLQVRAADNSTWFL 9458
